# [Pd(NHC)(μ-Cl)Cl]_2_: Versatile and Highly Reactive Complexes for Cross-Coupling Reactions that Avoid Formation of Inactive Pd(I) Off-Cycle Products

**DOI:** 10.1016/j.isci.2020.101377

**Published:** 2020-07-16

**Authors:** Tongliang Zhou, Siyue Ma, Fady Nahra, Alan M.C. Obled, Albert Poater, Luigi Cavallo, Catherine S.J. Cazin, Steven P. Nolan, Michal Szostak

**Affiliations:** 1 Department of Chemistry, Rutgers University, 73 Warren Street, Newark, NJ 07102, USA; 2 Department of Chemistry and Center for Sustainable Chemistry, Ghent University, Krijgslaan 281, S-3, 9000 Ghent, Belgium; 3 Separation and Conversion Technology Unit, VITO (Flemish Institute for Technological Research), Boeretang 200, 2400 Mol, Belgium; 4 EaStCHEM School of Chemistry, University of St Andrews, St Andrews, KY16 9ST, UK; 5 Institut de Química Computacional i Catàlisi and Departament de Química, Universitat de Girona, c/ Maria Aurèlia Capmany 69, Campus Montilivi, 17003 Girona, Catalonia, Spain; 6 King Abdullah University of Science & Technology, KAUST Catalysis Center (KCC), 23955-6900 Thuwal, Saudi Arabia

**Keywords:** Chemistry, Inorganic Chemistry, Catalysis, Organic Synthesis

## Abstract

The development of more reactive, general, easily accessible, and readily available Pd(II)–NHC precatalysts remains a key challenge in homogeneous catalysis. In this study, we establish air-stable NHC–Pd(II) chloro-dimers, [Pd(NHC)(μ-Cl)Cl]_2_, as the most reactive Pd(II)–NHC catalysts developed to date. Most crucially, compared with [Pd(NHC)(allyl)Cl] complexes, replacement of the allyl throw-away ligand with chloride allows for a more facile activation step, while effectively preventing the formation of off-cycle [Pd_2_(μ-allyl)(μ-Cl)(NHC)_2_] products. The utility is demonstrated via broad compatibility with amide cross-coupling, Suzuki cross-coupling, and the direct, late-stage functionalization of pharmaceuticals. Computational studies provide key insight into the NHC–Pd(II) chloro-dimer activation pathway. A facile synthesis of NHC–Pd(II) chloro-dimers in one-pot from NHC salts is reported. Considering the tremendous utility of Pd-catalyzed cross-coupling reactions and the overwhelming success of [Pd(NHC)(allyl)Cl] precatalysts, we believe that NHC–Pd(II) chloro-dimers, [Pd(NHC)(μ-Cl)Cl]_2_, should be considered as go-to precatalysts of choice in cross-coupling processes.

## Highlights

• Highly reactive, air-stable Pd^II^–NHC chloro-dimer catalysts for cross-coupling reactions
• Broad substrate scope, excellent functional group tolerance, and chemoselectivity
• Rapid one-step catalyst synthesis and facile catalyst activation
• DFT studies provide key insights into Pd^II^–NHC chloro-dimer activation pathway

## Graphical Abstract

**Figure:**
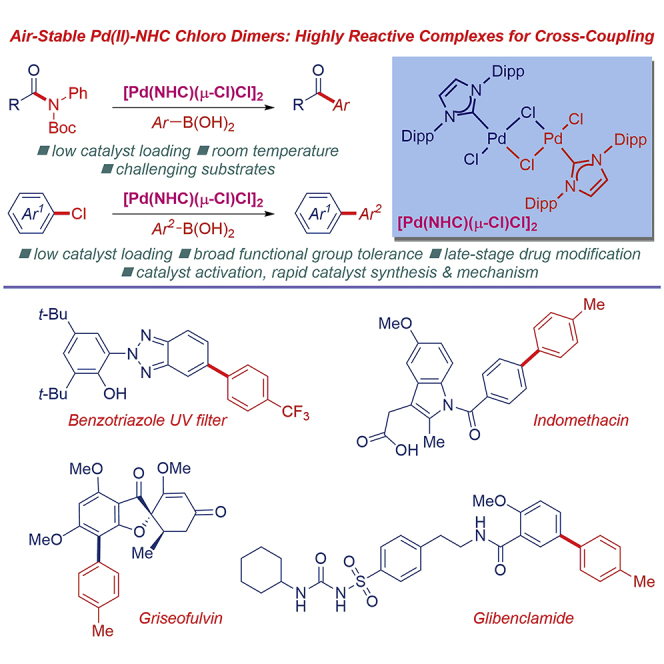

## Introduction

**Figure 1:**
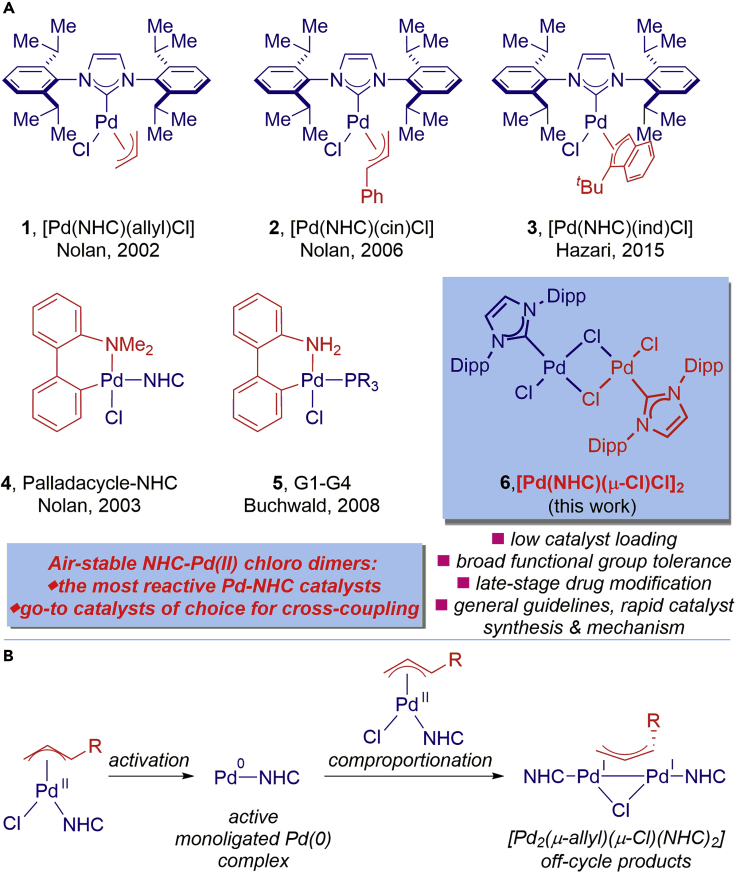
Pd–NHC Complexes in Cross-Coupling (A) Structures of well-defined Pd(II) precatalysts. (B) Comproportionation mechanism.

Palladium-catalyzed cross-coupling reactions are among the most powerful molecular assembly tools in chemistry by enabling facile construction of C–C and C–heteroatom bonds (Molander et al., 2013; Colacot, 2015; Diez-Gonzalez et al., 2009). Tremendous advances have been achieved through the discovery of tailor-made ligands that facilitate challenging oxidative addition and reductive elimination elementary steps (Fortman and Nolan, 2011; Martin and Buchwald, 2008). The Pd-catalyzed Suzuki-Miyaura reaction now ranks as the most frequently executed catalytic transformation in production of pharmaceuticals, with numerous commercial syntheses of drugs singularly relying on this bond forming technology (Blakemore et al., 2018). Mechanistically, it is now established that achieving high activity of Pd catalysts involves the formation of monoligated Pd(0) species (Christmann and Vilar, 2005). As a result, the development of well-defined Pd(0) and Pd(II) precatalysts, wherein Pd and ligand are in a 1:1 ratio, represents a major direction in catalyst design (Molander et al., 2013; Colacot, 2015; Diez-Gonzalez et al., 2009; Fortman and Nolan, 2011; Martin and Buchwald, 2008). In this context, commercially available [Pd(NHC)(allyl)Cl] (NHC = N-heterocyclic carbene) complexes developed by one of us (S.P.N.) are among the most powerful and widely used Pd catalysts for various cross-coupling reactions worldwide (Marion et al., 2006; Hopkinson et al., 2014; Nolan and Cazin, 2017); however, their reactivity is limited by the formation of off-cycle Pd(I) allyl products (Figures 1A and 1B) (Hruszkewycz et al., 2014; Melvin et al., 2015; Johansson Seechurn et al., 2017).

**Chart 1:**
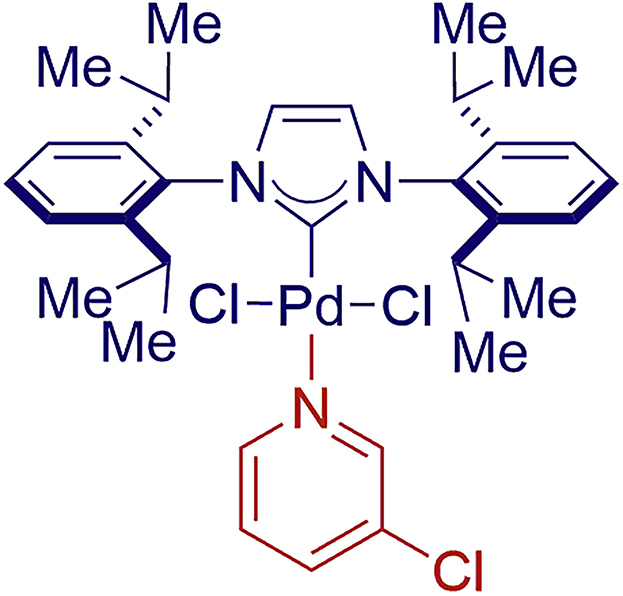
Structure of Pd-PEPPSI-IPr (**10**)

The [Pd(NHC)(allyl)Cl] complexes were first introduced in 2002 (Marion et al., 2006; Viciu et al., 2002a, 2002b). The proposed activation pathway involved a nucleophilic addition to the allyl or the halide displacement with an alkoxide and reductive elimination to give the active NHC–Pd(0) species. In 2006, it was established that addition of bulky substituents at the 1-position of the allyl ligands, such as cinnamyl or prenyl, resulted in a dramatic increase of catalyst efficiency (Marion et al., 2006). In the meantime, [Pd(NHC)(cin)Cl] (cin = cinnamyl) complexes complexes have become a commercially available class of Pd catalysts of choice for cross-coupling reactions. The use of NHC ancillary ligands expedites the reaction development owing to the strong σ-donating properties of NHC ligands cf. phosphines (Martin and Buchwald, 2008; Marion et al., 2006; Hopkinson et al., 2014; Nolan and Cazin, 2017). These [Pd(NHC)(allyl)Cl] catalysts are now available in several forms from various suppliers, facilitating challenging C–C and C–heteroatom cross-couplings worldwide. It should also be noted that, in addition to Pd(II)–NHC precatalysts bearing highly effective allyl-type or palladacycle-type throw-away ligands (Figure 1A), heteroatom donors, including the PEPPSI-class of catalysts, have attracted considerable attention (Chart 1) (Nolan and Cazin, 2017; O'Brien et al., 2006).

In 2014, it was identified that the formation of inactive [Pd_2_(μ-allyl)(μ-Cl)(NHC)_2_] dimers during the activation of [Pd(NHC)(allyl)Cl] complexes takes place (Figure 1B) (Hruszkewycz et al., 2014). It was established that the monoligated NHC–Pd(0) species undergoes comproportionation with [Pd(NHC)(allyl)Cl] monomers to give the inactive allyl-bridged Pd(I) dimers, [Pd_2_(μ-allyl)(μ-Cl)(NHC)_2_]. The extent of formation of this inactive dipalladium complex is dependent on the presence of substituents at the allylic terminal position. Thus, allyl-type complexes bearing sterically bulky *t*-Bu-indenyl ligand, [Pd(NHC)(1-*t*-Bu-ind)Cl], showed high reactivity by suppressing the formation of the inactive Pd(I) allyl products (Melvin et al., 2015). However, this class of catalysts still relies on catalyst activation by allyl displacement (cf. dissociation), multi-step synthesis, and the introduction of waste-generating throw-away allyl ligand, which is less than desirable from the activation-, reactivity-, atom-, step-, and cost-economy perspective.

Over the past years, we have introduced Pd–NHC complexes for the cross-coupling of amides through oxidative addition of N–C(O) bonds, which is also instrumental for the cross-coupling of bench-stable esters via acyl-metals from common amides and esters (Shi et al., 2018). In the context, we have studied Pd–NHC complexes with various throw-away ligands (M.S.) (Lei et al., 2017).

In this study, we establish air-stable NHC–Pd(II) chloro dimers, [Pd(NHC)(μ-Cl)Cl]_2_, as the most reactive Pd(II)–NHC catalysts developed to date. Most crucially, compared with [Pd(NHC)(allyl)Cl] complexes, replacement of the allyl throw-away ligand with chloride allows for a more facile activation step, while effectively preventing the formation of off-cycle [Pd_2_(μ-allyl)(μ-Cl)(NHC)_2_] products. These catalysts are highly reactive, easy to prepare, readily activated to Pd(0)–NHC by dimer dissociation (cf. allyl displacement), and avoid cost- and waste-generating allyl ligands. The utility of this class of catalysts is demonstrated via broad compatibility with privileged biaryls and the direct, late-stage functionalization of common pharmaceuticals. Extensive computational studies provide key insight into the NHC–Pd(II) chloro dimer activation pathway. With the goal of providing increasingly practical technologies, a facile synthesis of NHC–Pd(II) chloro dimers in one-pot from NHC salts is reported. Considering the tremendous utility of Pd-catalyzed cross-coupling reactions in chemical synthesis and the overwhelming success of [Pd(NHC)(allyl)Cl] precatalysts, we believe that NHC–Pd(II) chloro dimers, [Pd(NHC)(μ-Cl)Cl]_2_, should be considered as go-to precatalysts of choice in cross-coupling processes.

### Results and Discussion

#### Catalytic Studies

Our investigation of the reactivity of NHC–Pd(II) chloro dimers, [Pd(NHC)(μ-Cl)Cl]_2_, was initiated by evaluating the reactivity of a model IPr-based catalyst (IPr = 1,3-bis(2,6-diisopropylphenyl)imidazol-2-ylidene) in the cross-coupling of amide **7** with boronic acids. Somewhat ironically, it is worth noting that the [Pd(IPr)(μ-Cl)Cl]_2_ catalyst was first reported by one of us (S.P.N.) in 2002; however, at that point the focus was aimed at the seemingly more reactive [Pd(NHC)(allyl)Cl] complexes (Viciu et al., 2002a, 2002b; Navarro et al., 2003). Now, after nearly 20 years in catalyst development (Marion et al., 2006; Shi et al., 2018), we hypothesized that [Pd(NHC)(μ-Cl)Cl]_2_ complexes might be of great benefit in cross-coupling reactions owing to facile activation and elimination of the off-cycle products in the absence of problematic allyl throw-away ligands.

Selected optimization results are summarized in Table 1. Full optimization results are presented in the Supplemental Information. After preliminary experiments, we found that the desired cross-coupling occurred in >98% yield at 0.25 mol% catalyst loading under very mild room temperature conditions (Table 1, entry 3). Furthermore, the reaction could be successfully performed at 0.050–0.025 mol% catalyst loading (>95% conversion) by increasing the temperature to 40°C (Table 1, entries 7 and 8).

**Table 1:** Optimization of Pd-Catalyzed Suzuki-Miyaura Cross-Coupling of Amides

Entry	Catalyst (mol%)	Boronic Acid (equiv)	Base (equiv)	H_2_O (equiv)	Yield^a^ (%)
1	1.5	1.2	2.0	0	56
2	1.5	1.2	2.0	5	>98
3	0.25	1.05	1.1	5	>98
4	0.05	1.05	1.1	5	32
5^b^	0.05	2.0	1.1	5	74
6^b^^,^^c^	0.05	2.0	3.0	5	85
7^b^^,^^d^	0.05	2.0	3.0	5	>98
8^b^^,^^d^	0.025	2.0	3.0	5	96

Conditions: amide **7a**, PhC(O)–NPh/Boc, (1.0 equiv), catalyst ([Pd(IPr)(μ-Cl)Cl]_2_) (x mol%), 4-Tol-B(OH)_2_ (1.05–2.0 equiv), K_2_CO_3_ (1.1–3.0 equiv), H_2_O (0–5 equiv), THF (0.25 M), 23°C, 12 h.

a GC/^1^H NMR yields.

b 0.50 M.

c Toluene.

d 40°C. See Transparent Methods for full details. IPr, 1,3-bis(2,6-diisopropylphenyl)imidazol-2-ylidene.

**Figure 2:**
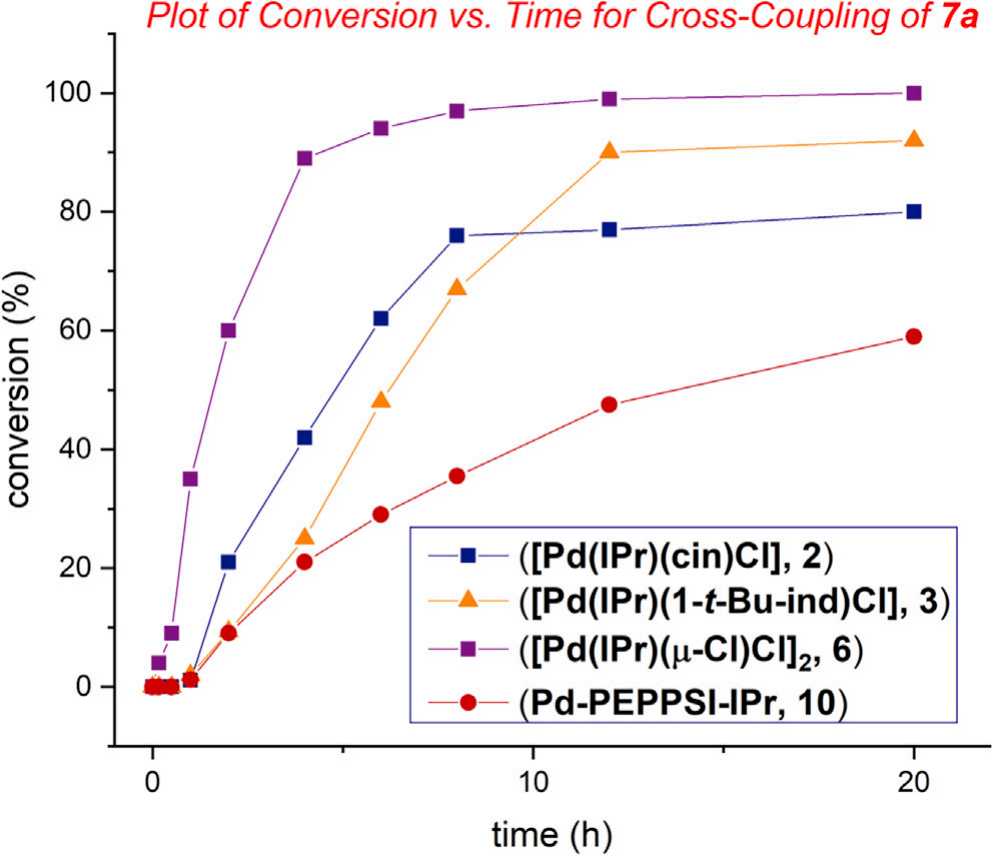
Kinetic Profile of Suzuki-Miyaura Cross-Coupling of Amides Conditions: **7a** (PhCONBocPh), 4-Tol-B(OH)_2_ (2.0 equiv), catalyst ([Pd(IPr)(μ-Cl)Cl]_2_, 0.05 mol%; other catalysts, 0.10 mol%), K_2_CO_3_ (3.0 equiv), H_2_O (5 equiv), toluene (0.50 M), 23°C, 0–20 h.

At this point, kinetic profiling studies were conducted to gain insight into the reaction and compare the reactivity of [Pd(IPr)(μ-Cl)Cl]_2_ with other classes of Pd(II)–NHC catalysts (Figure 2). Crucially, in kinetic profiling studies, we found that [Pd(IPr)(μ-Cl)Cl]_2_ (**6**) was a superior catalyst to [Pd(IPr)(cin)Cl] and [Pd(IPr)(1-*t*-Bu-ind)Cl] (Marion et al., 2006; Melvin et al., 2015), whereas the heterocycle-based Pd-PEPPSI-IPr (**10**) (O'Brien et al., 2006) (Chart 1) showed the lowest reactivity. It is well known that activation of Pd-PEPPSI-type catalysts is slow (Hopkinson et al., 2014). However, it should also be noted that, in specific cases, the rate of catalyst activation might differ between substrates, including cases when substrate activation by nucleophilic addition takes place (Shi et al., 2018). The reaction of amide **7** gave 89% conversion after 4 h using **6** as catalyst, which can be compared with 42% and 25% conversion when using [Pd(IPr)(cin)Cl] and [Pd(IPr)(1-*t*-Bu-ind)Cl] catalysts. Crucially, initial rates revealed that the NHC–Pd(II) chloro dimer [Pd(IPr)(μ-Cl)Cl]_2_ catalyst gives 3.1 and 4.2 times faster reaction than the cinnamyl- and *t*-Bu-indenyl-based catalysts.

Our preliminary studies indicate that sterically hindered imidazolylidene and saturated imidazolinylidene ligands perform well as ancillary ligands in [Pd(NHC)(μ-Cl)Cl]_2_ complexes. As such, two other chloro dimers [Pd(NHC)(μ-Cl)Cl]_2_ based on SIPr and IPr∗ NHC ancillary ligands were prepared and evaluated in the cross-coupling of amide **7** with 4-Tol-B(OH)_2_ (see Scheme S1). The reactivity of saturated imidazolinylidene-based catalyst SIPr (SIPr = 1,3-bis(2,6-diisopropylphenyl)imidazolidin-2-ylidene) (74% yield) and sterically hindered IPr∗ (IPr∗ = 1,3-bis(2,6- bis(diphenylmethyl)4-methylphenyl)imidazol-2-ylidene) (Izquierdo et al., 2014) (24% yield) at 0.050 mol% loading was identified as promising but provided lower yields than **6**. Our ongoing studies are focused on the development of NHC ligands that can be broadly utilized as supporting ligands in cross-coupling reactions.

**Scheme 1:**
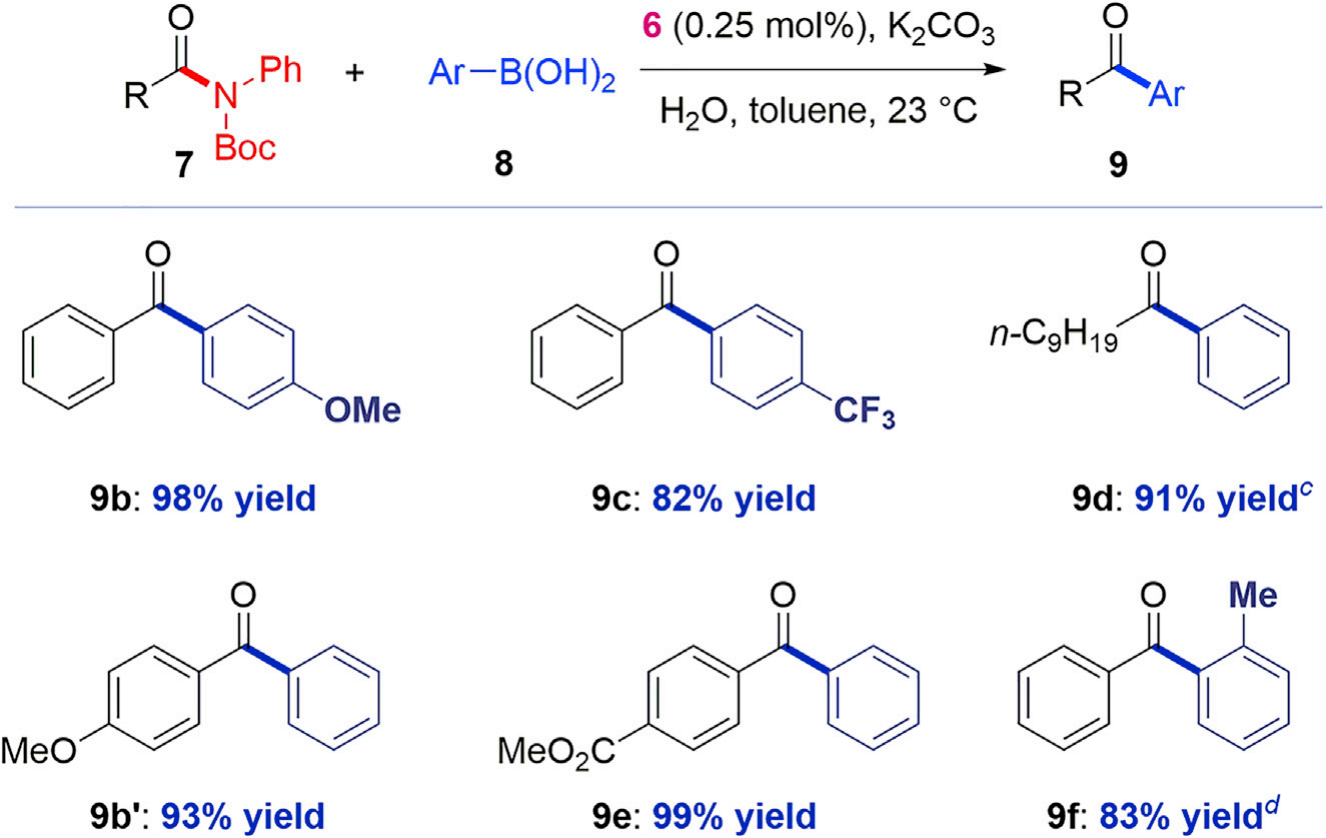
Scope of Amide Suzuki-Miyaura Cross-Coupling Conditions: amide (1.0 equiv), Ar-B(OH)_2_ (2.0 equiv), [Pd(IPr)(μ-Cl)Cl]_2_, 0.25 mol%, K_2_CO_3_ (3.0 equiv), H_2_O (5 equiv), toluene (1.0 M), 23°C, 12 h. ^a^Isolated yields. ^b^0.50 mol%. ^d^[Pd(IPr∗)(μ-Cl)Cl]_2_, 0.25 mol%. See Transparent Methods for details.

The substrate scope of amide bond cross-coupling using the NHC–Pd(II) chloro dimer [Pd(IPr)(μ-Cl)Cl]_2_ **6** was briefly investigated (Scheme 1). As such, the cross-coupling of electronically varied amides and boronic acids, including electrophilic functional groups (**9e**), alkyl amides (**9d**), and deactivated substrates (**9c, 9b′**), could be achieved at room temperature at low catalyst loading in excellent yields. Furthermore, a turnover number (TON) of 14,800 was calculated for the cross-coupling of amide **7a** ([Pd(IPr)(μ-Cl)Cl]_2_ (**6**), 25 ppm, 4-Tol-B(OH)_2_, 120°C, 2-MeTHF). The use of 2-MeTHF is preferred for TON determination owing to much better solubility of the base in this solvent (see Scheme S3).

At this stage, we turned our attention to the more synthetically significant biaryl Suzuki-Miayura cross-coupling. Beyond doubt, the biaryl Suzuki-Miyaura synthesis ranks as the most important and powerful C–C bond forming cross-coupling reaction discovered to date (Fyfe and Watson, 2017). The impact of the biaryl Suzuki-Miyaura cross-coupling is clearly illustrated by the change of the shape of bioactive pharmacophores that are now prepared as medicines and scaffolds in drug discovery enabled by the emergence of this cross-coupling technology (Yet, 2018).

Our initial optimization focused on two standard conditions that are routinely applied in the development of Suzuki-Miyaura cross-coupling, namely, the much preferred conditions using weak base (K_2_CO_3_) and the alternative conditions using strong base (KO*t*-Bu) (see Table S1). Crucially, the NHC–Pd(II) chloro dimer [Pd(IPr)(μ-Cl)Cl]_2_ **6** promoted the model cross-coupling of 4-chlorotoluene with Ph–B(OH)_2_ in quantitative yield under both conditions in EtOH as a solvent.

**Figure 3:**
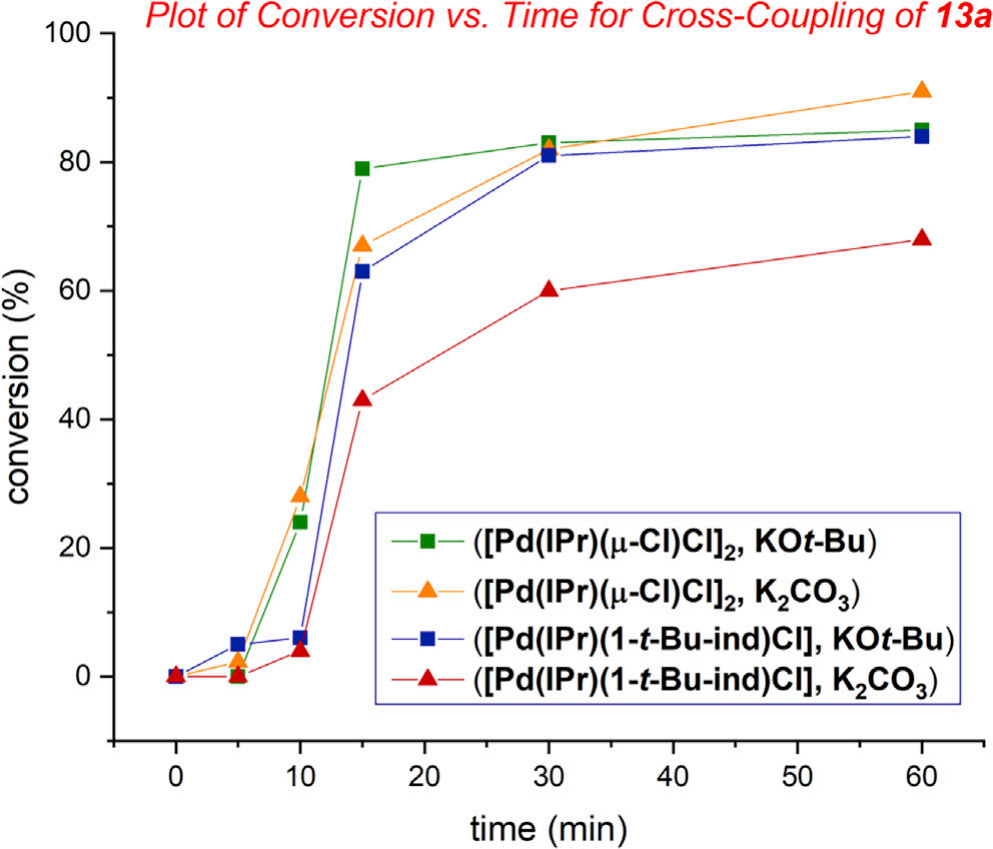
Kinetic Profile of Biaryl Suzuki-Miyaura Cross-Coupling of Aryl Chlorides Conditions: **13a** (4-Tol-Cl), Ph-B(OH)_2_ (1.05 equiv), catalyst ([Pd(IPr)(μ-Cl)Cl]_2_, 0.50 mol%; [Pd(IPr)(1-*t*-Bu-ind)Cl], 1.0 mol%), KO*t-*Bu (1.1 equiv)/K_2_CO_3_ (2.2 equiv), EtOH (0.50 M), 23°C, 0–1 h.

Next, kinetic profiling studies revealed the NHC–Pd(II) chloro dimer [Pd(IPr)(μ-Cl)Cl]_2_ **6** is a superior catalyst to [Pd(IPr)(1-*t*-Bu-ind)Cl] under the much preferred mild base conditions using K_2_CO_3_ (orange triangles versus red triangles, Figure 3) consistent with facile activation by dimer dissociation. Interestingly, the reactivity of **6** is similar to [Pd(IPr)(1-*t*-Bu-ind)Cl] under KO*t*-Bu conditions (green squares versus blue squares, Figure 3). It is also worth noting that K_2_CO_3_ is the preferred base in case of selected substrates (see Schemes S5 and S6). We have further evaluated the comparative reactivity of the NHC–Pd(II) chloro dimer [Pd(IPr)(μ-Cl)Cl]_2_ **6** and [Pd(IPr)(1-*t*-Bu-ind)Cl] in the cross-coupling of electron-rich and sterically hindered substrates, wherein **6** also showed better reactivity. Our preliminary studies indicate that [Pd(NHC)(μ-Cl)Cl]_2_ catalysts are efficient in cross-coupling of sterically hindered 2,6-di-substituted aryl chlorides (see Scheme S6). Our future studies will focus on expanding the scope of reactions enabled by [Pd(NHC)(μ-Cl)Cl]_2_ catalysts.

**Scheme 2:**
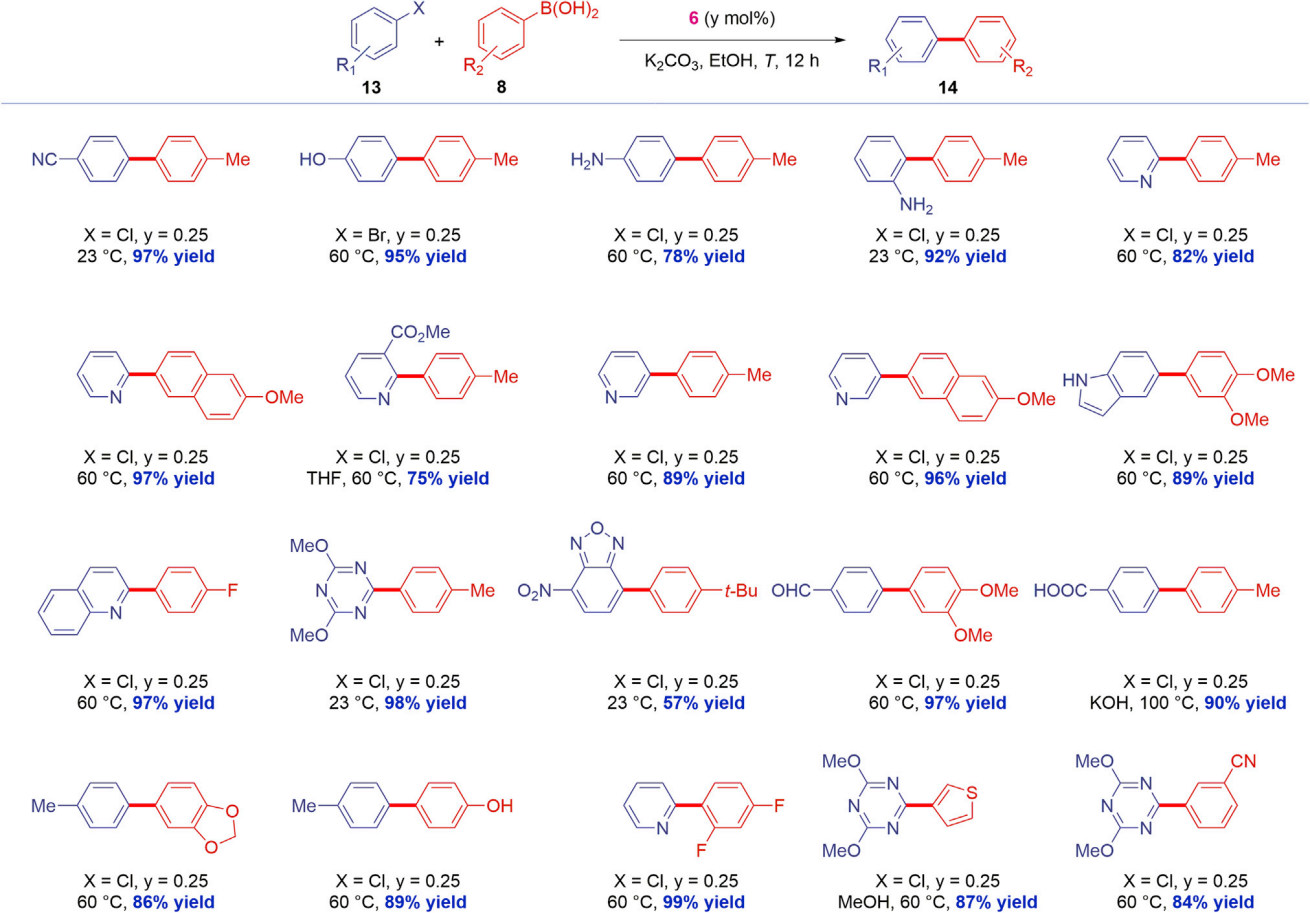
Scope of Pd-Catalyzed Biaryl Suzuki-Miyaura Cross-Coupling Conditions: Ar-X (1.0 equiv), Ar-B(OH)_2_ (2.0 equiv), K_2_CO_3_ (3.0 equiv), [Pd(IPr)(μ-Cl)Cl]_2_ (**6**) (y mol %), EtOH (0.50 M), 12 h. Isolated yields. See Transparent Methods for details.

**Scheme 3:**
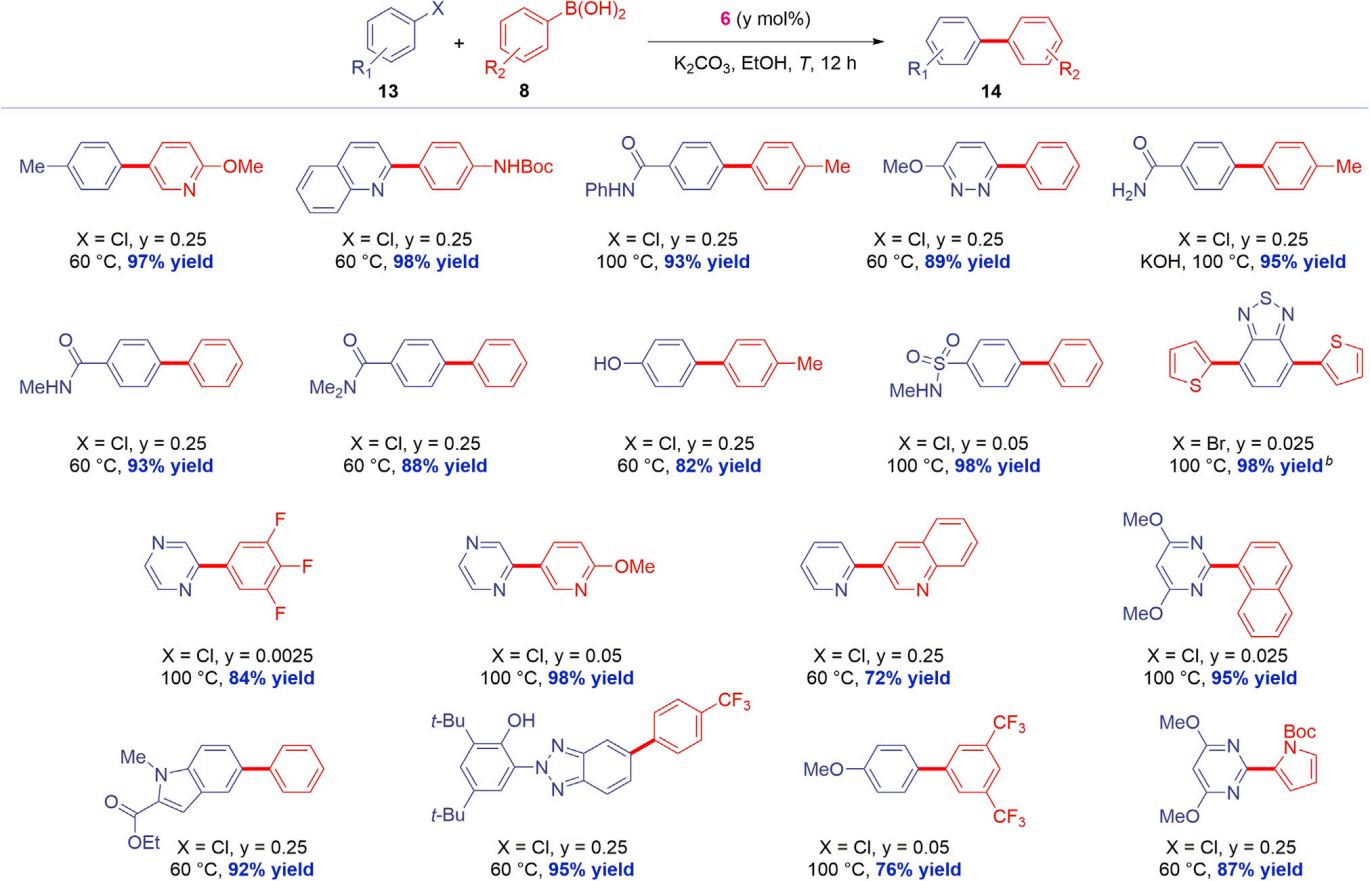
Scope of Pd-Catalyzed Biaryl Suzuki-Miyaura Cross-Coupling Conditions: Ar-X (1.0 equiv), Ar-B(OH)_2_ (2.0 equiv), K_2_CO_3_ (3.0 equiv), [Pd(IPr)(μ-Cl)Cl]_2_ (**6**) (y mol %), EtOH (0.50 M), 12 h. Isolated yields. ^a^Ar-B(OH)_2_ (3.0 equiv). See Transparent Methods for details.

**Scheme 4:**
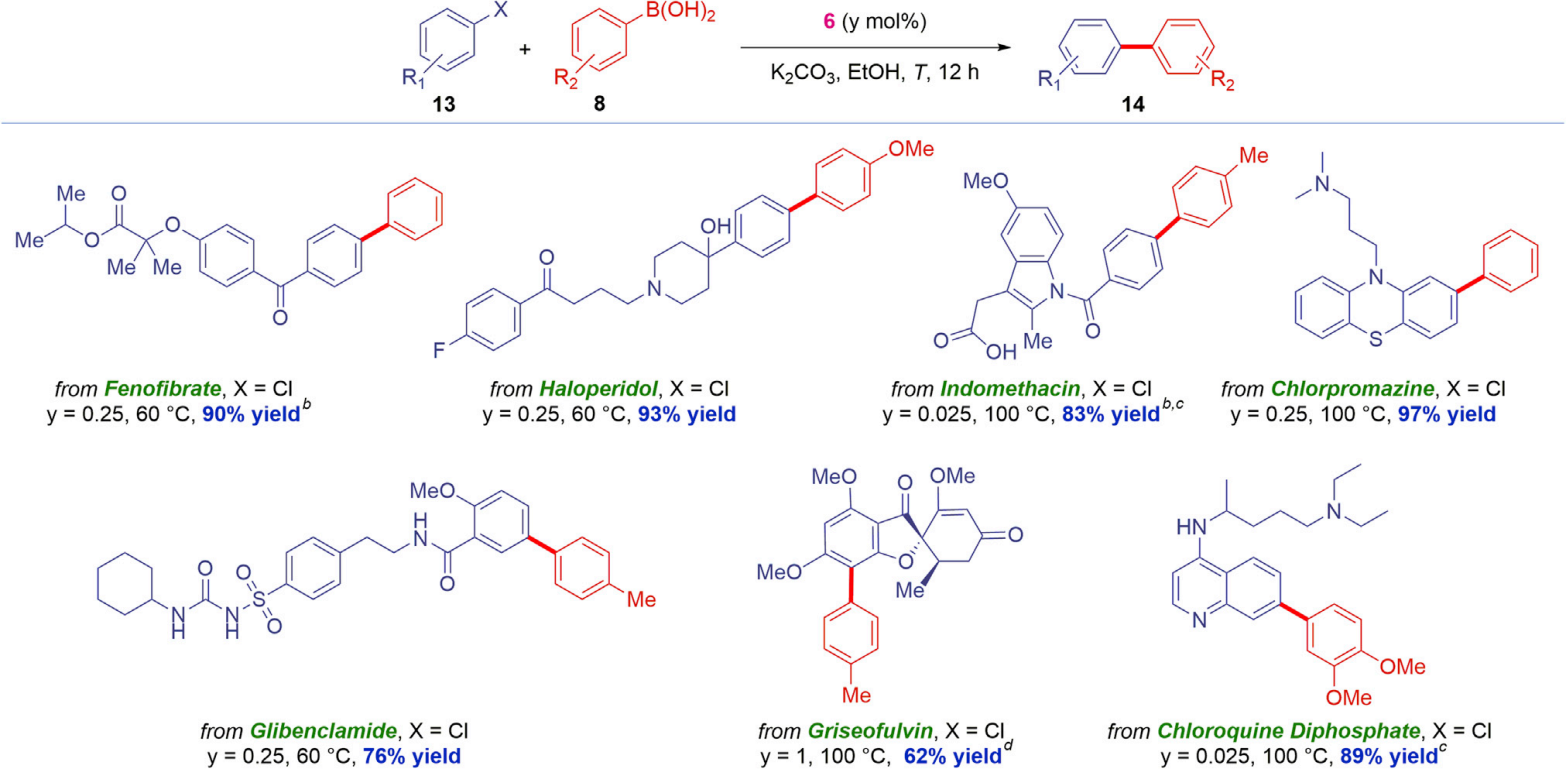
Direct Cross-Coupling of Pharmaceuticals Conditions: Ar-X (1.0 equiv), Ar-B(OH)_2_ (2.0 equiv), K_2_CO_3_ (3.0 equiv), [Pd(IPr)(μ-Cl)Cl]_2_ (6) (y mol %), EtOH (0.50 M), 12 h. Isolated yields. ^a^*i*PrOH. ^b^K_2_CO_3_ (5 equiv). ^c^*t*-BuOH. See Transparent Methods for details.

With the knowledge that the NHC–Pd(II) chloro dimer [Pd(IPr)(μ-Cl)Cl]_2_ **6** is a highly effective catalyst operating under mild, synthetically useful conditions, we next investigated the synthetic scope of **6** with a focus on compatibility with privileged biaryls and the direct, late-stage functionalization of common drugs (Schemes 2, 3, and 4).

As outlined in Schemes 2 and 3, the NHC–Pd(II) chloro dimer [Pd(IPr)(μ-Cl)Cl]_2_ **6** can be deployed successfully with a remarkably broad range of aryl chlorides and boronic acids (Afagh and Yudin, 2010). Most crucially, the highlighted functional groups are among the most commonly encountered in pharmaceuticals and allow for further functionalization by traditional or orthogonal cross-coupling methods (Blakemore et al., 2018). A variety of synthetically useful substituents is tolerated, including nitriles; unprotected hydroxyl; free amines; pyridines; esters; free indoles; triazines; benzofurazans; aldehydes; free carboxylic acids; dioxolanes; polyfluorinated substrates; Boc-protected amines; NH-benzamides; pyridazines; primary, secondary, tertiary amides; sulfonamides; 2,1,3-benzothiadiazoles; pyrazines; bis-heterocycles; pyrimidines; functionalized indoles; benzotriazoles; and pyrroles, enabling the synthesis of privileged biaryl motifs in excellent yields. When aryl chlorides gave lower conversion or are not easily available, aryl bromides could be used successfully.

Furthermore, the NHC–Pd(II) chloro dimer [Pd(IPr)(μ-Cl)Cl]_2_ **6** could be readily deployed in the direct cross-coupling of densely functionalized pharmaceuticals (Scheme 4), such as Fenofibrate, Haloperidol, Indomethacin, Chlorpromazine, Glibenclamide, Griseofulvin, and Chloroquine, thus clearly demonstrating the potential impact on the synthesis and potential late-stage further derivatization of complex biaryls in pharmaceutical settings. The selected substrates further demonstrate the functional group tolerance with respect to privileged motifs that are broadly present in pharmaceutical development.

Preliminary studies using the NHC–Pd(II) chloro dimer [Pd(IPr)(μ-Cl)Cl]_2_ **6** indicated that the cross-coupling at 25 ppm catalyst loading is also feasible using K_2_CO_3_ as a mild carbonate base (see Scheme S7). To our knowledge, these results establish the NHC–Pd(II) chloro dimer [Pd(IPr)(μ-Cl)Cl]_2_ **6** as the most active Pd(II)–NHC catalysts discovered to date and a major improvement over the overwhelmingly successful [Pd(NHC)(allyl)Cl] catalysts. The use of the commonly available IPr ligand and the commercial availability on large scale (i.e., kg scale) surely make the NHC–Pd(II) chloro dimer [Pd(IPr)(μ-Cl)Cl]_2_ **6** an attractive tool to be used in small- and larger-scale molecular assembly cross-coupling strategies.

### Mechanism Studies

To gain further insight into the reactivity of the palladium halide dimer catalysts, [Pd(NHC)(μ-X)X]_2_, we prepared the bromo- and iodo-based congeners, [Pd(IPr)(μ-Br)Br]_2_ and [Pd(IPr)(μ-I)I]_2_, and evaluated their reactivity in the model Suzuki cross-coupling (see Table S2). The bromo dimer showed slightly lower reactivity than the chloro relative, whereas the iodo dimer was completely unreactive across electronically and sterically differentiated substrates at room temperature; however, moderate conversion was observed at 60°C. This establishes the reactivity order of the halide dimer catalysts: Cl > Br > I, which is consistent with the activation of [Pd(NHC)(μ-X)X]_2_ halide dimer catalysts to yield the active, monoligated NHC–Pd(0) complex (Fairlamb et al., 2006).

**Scheme 5:**
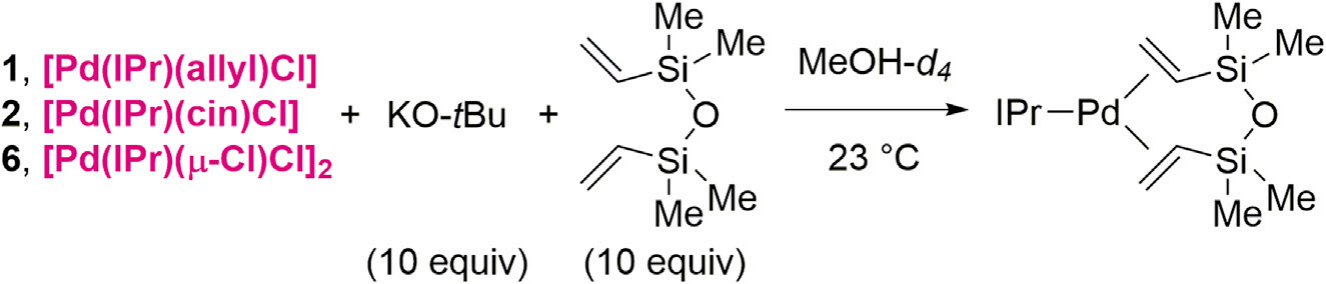
Rates of Activation of Allyl, Cinnamyl and Chloro Dimer, [(NHC)Pd(μ-Cl)Cl]_2_, Complexes Conditions: Pd–NHC (1.0 equiv), KO*t*-Bu (10 equiv), dvds (10 equiv), MeOH-*d*_*4*_, 23°C, 0–3 h.

To further understand the high reactivity of **6**, we measured the activation rate to the monoligated IPr–Pd(0) (Scheme 5). The rate was measured in the presence of dvds (dvds = 1,3-divinyl-1,1,3,3-tetramethyldisiloxane) and base (Hruszkewycz et al., 2014). We found that, in a comparison between [Pd(IPr)(allyl)Cl], [Pd(IPr)(cin)Cl], and [Pd(IPr)(μ-Cl)Cl]_2_ (**6**), the allyl complex is activated the fastest (*k*_obs_ = 1.1 ∗ 10^−3^ s^−1^), whereas the chloro dimer (*k*_obs_ = 7.0 ∗ 10^−4^ s^−1^) was activated faster than the cinnamyl complex (*k*_obs_ = 3.0 ∗ 10^−4^ s^−1^) (see Scheme S8). The absence of an allyl moiety in **6** obviously excludes a decomposition route leading to bridged-allyl dinuclear palladium complexes. The high activation rate of [Pd(IPr)(μ-Cl)Cl]_2_ is consistent with the excellent activity of this catalyst in cross-coupling.

### Computational Analysis of [(IPr)Pd(***μ***-Cl)Cl]_2_ Activation

**Figure 4:**
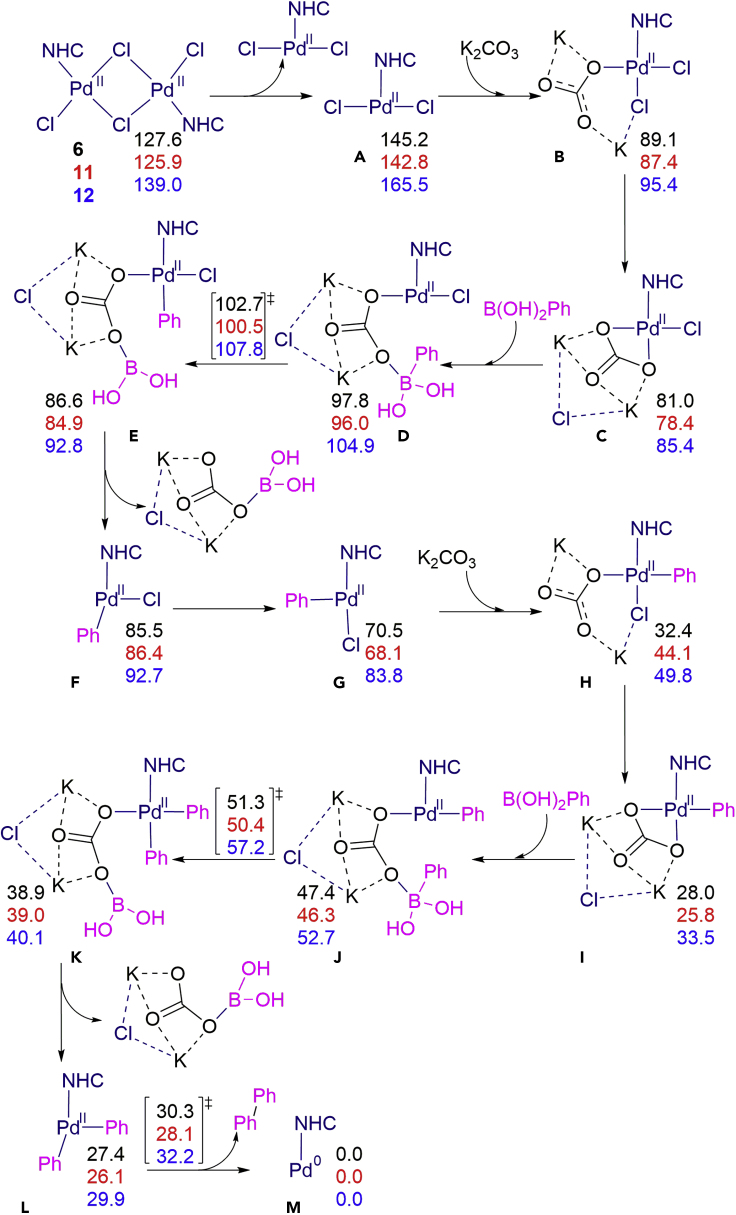
DFT-Optimized Pathway (Relative Energies to Pd(0) in kcal/mol) for the Activation of Catalysts **6** (black), **11** (red), and **12** (blue). **6** = IPr, **11** = SIPr, **12** = IPr∗, [Pd(NHC)(μ-Cl)Cl]_2_

DFT studies (M06/Def2TZVP ∼ SDD//BP86-d3(PCM,THF)/SVP ∼ SDD) were conducted to gain insight into the exact activation pathway employed by **6** and compare it with those of other classes of air-stable Pd(II) precatalysts (Data S1, Cartesian coordinates and energies, related to Figure 4). From catalyst **6**, via a barrierless step (checked by a linear transit), the simple cleavage of the dimer requires 17.6 kcal/mol, thus affordable at room temperature. Analyzing the halide that holds together the dimer structure, calculations validated the results found in the reactivity order of the halide dimer catalysts (see Table S2), with higher thermodynamic cost for the dimer cleavage of 2.1 and 10.5 kcal/mol for Br and I, respectively. The latter value is in perfect agreement with experiments and confirms the activity at 60°C and the poorer results at rt. Second, the analysis moved to the different NHC ligands that occupy different space around the metal. The mechanism to activate catalysts **6**, **11**, and **12**, i.e., that leads to the active catalytic Pd(0) species, is included in Figure 4. The computed values for the barrierless dimer cleavage are 17.6 (IPr), 16.8 (SIPr), and 26.5 (IPr∗) kcal/mol, thus becoming unfavored for larger NHC ligands (Falivene et al., 2016, 2019). The higher energy cost for the cleavage of **12** is in agreement with experimental results (see Scheme S1), explaining the poor performance of the sterically very encumbered **12** at rt, but much improved activity at more elevated temperatures.

**Figure 5:**
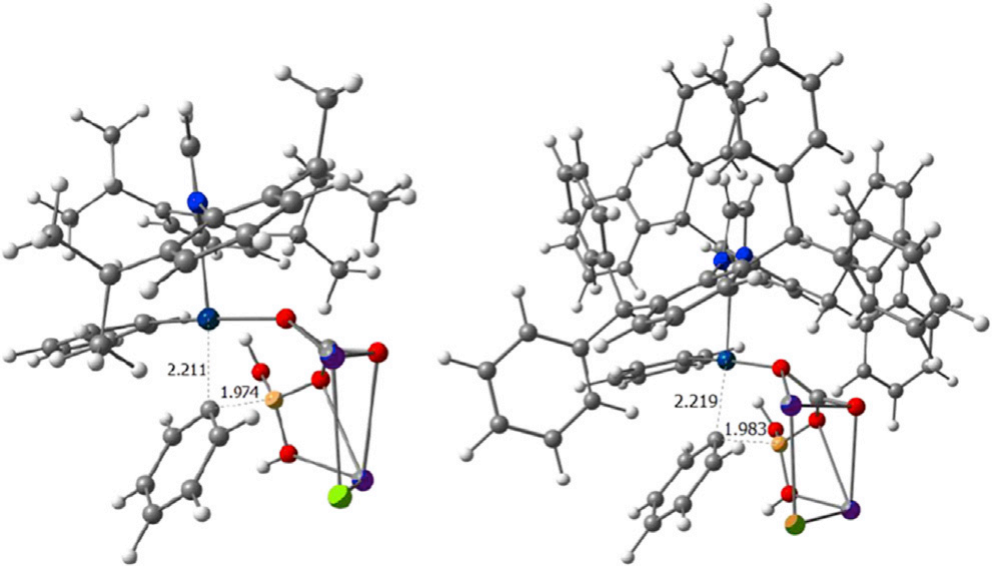
DFT-Optimized Transition State of the Second Aryl Transfer from Boron to Palladium for **6** (left) and **12** (right); Main Distances Are Given in Å

Post dimer cleavage, we envisaged that Ph-B(OH)_2_ together with the base K_2_CO_3_ must assist in the displacement/removal of one of the halides and deliver a phenyl ligand. This hypothesis is supported by results in Table 1 where better catalytic performance is obtained with an excess of boronic acid. After the first rearrangement caused by the entering K_2_CO_3_, the Ph-B(OH)_2_ bonds to the ionic KCO_3_ moiety, and the aryl group on boron is transferred to the palladium in the **e**→**f** step with an energetic cost of 21.7, 22.1, and 22.4 kcal/mol for **6**, **11**, and **12**, respectively, calculated not from intermediate **d**, but **c** as a reference. In the absence of base, the aryl transfer to the metal shows an increase in the energy barrier for **6** of 18.2 kcal/mol. Next, there is the favorable thermodynamic dissociation of the K_2_CO_3_ClB(OH)_2_ moiety, followed by a second coordination of a base that in combination of a second Ph-B(OH)_2_ moiety allows the aryl transfer from boron to palladium (see Figure 5). The kinetic requirement of the latter **j**→**k** step is 23.3, 24.6, and 23.6 kcal/mol for **6**, **11**, and **12**, respectively, calculated from intermediate **i**. In the precatalyst activation sequence, this latter step becomes the rate determining step (rds) for **6** and **11**, whereas for **12** this remains the halide bond cleavage of the dimer. Finally, once the K_2_CO_3_ClB(OH)_2_ moiety is released, the two aryl groups bound to palladium eliminate and form biphenyl and yield a Pd(0) species. Alternatively, instead of involving a second equivalent of base, the release of chlorobenzene from the initially formed [Pd(NHC)(Ph)Cl] was studied. This reductive elimination was found to be not kinetically facile, with an energy barrier of 22.4 kcal/mol, together with a thermodynamic cost of 18.1 kcal/mol (see Figure S1). Using the Pd(0) species for the acyl Suzuki-Miyaura cross-coupling of amides by N–C(O) cleavage has been previously shown to involve upper energy barriers of 23.8 and 26.5 kcal/mol for catalysts **6** and **12** (Li et al., 2018), thus mirroring the same trend as in the pre-activation of the corresponding catalysts.

We also compared the energetics for the dimeric **6** with those for the monomeric **1** and **2** leading to the Pd(0) species (see Figure S2). Even though the kinetics require just 25.3 and 23.3 kcal/mol for **1** and **2**, respectively, generation of an active species is hindered by the starting metal catalyst since formation of a bridged allyl dipalladium is highly favored by 17.4 and 14.3 kcal/mol. And this forces a kinetic requirement of 30.9 and 27.2 kcal/mol to recover the Pd(0) species. Thus, the catalyst itself with the off-cycle intermediate blocks the formation of the catalytic active species Pd(0) at mild temperature, contrarily to what happens with simple halide bridged catalysts **6**, **11**, and even **12**, studied here. Not having any allyl or substituted allyl supporting ligand appears to represent the simplest solution to avoiding catalyst deactivation.

### One-Pot Synthesis of [Pd(IPr)(***μ***-Cl)Cl]_2_

**Scheme 6:**
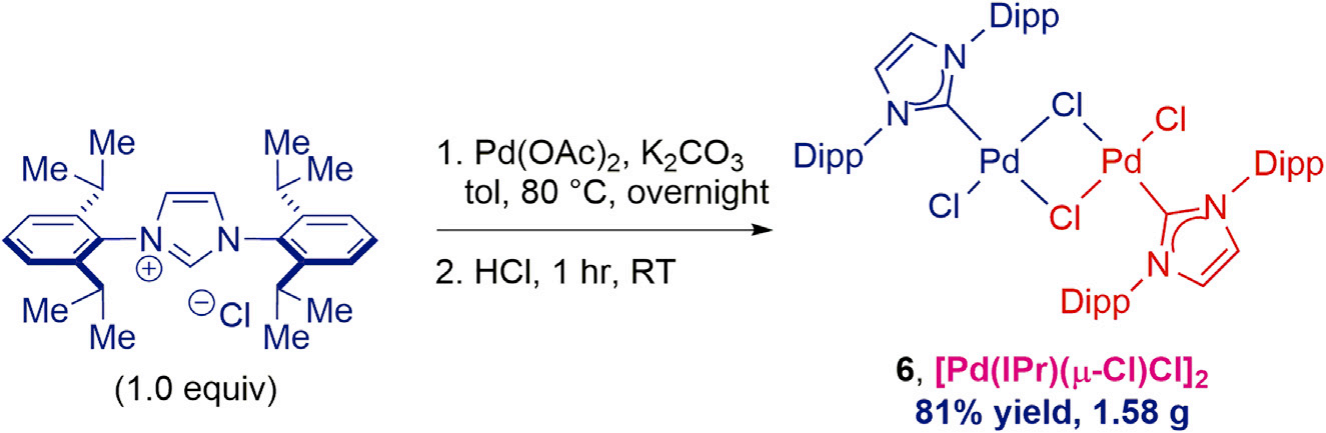
Facile, One-Step Synthesis of **6** Conditions: IPrHCl (1.0 equiv, 3.7 mmol), Pd(OAc)_2_ (1.2 equiv), K_2_CO_3_ (4 equiv), toluene, 80°C, followed by addition of HCl.

Our catalytic experiments clearly indicated the excellent activity of the NHC–Pd(II) chloro dimer [Pd(IPr)(μ-Cl)Cl]_2_ **6**. To provide practical synthetic technologies to practitioners, we developed a facile one-pot synthesis of NHC–Pd(II) chloro dimers from NHC salts (Scheme 6). As such, the air-stable NHC–Pd(II) chloro dimer [Pd(IPr)(μ-Cl)Cl]_2_ **6** could be readily prepared both on a small scale (0.11 mmol, ca. 60 mg) or on a preparative gram scale (3.7 mmol, 1.69 g) in 81% yield. The rapid availability of **6** compares very favorably with other Pd(II)–NHC precatalysts (*note that* ***6*** *is also already commercially available*) and should provide facile access to this class of catalysts for various cross-coupling technologies as well as for a plethora of other catalytic reactions that require monoligated Pd complexes, including C–H activation and hydrofunctionalization processes (Hopkinson et al., 2014; Nolan and Cazin, 2017).

### Conclusions

In summary, we have established air-stable NHC–Pd(II) chloro dimers, [Pd(NHC)(μ-Cl)Cl]_2_, as the most reactive Pd(II)–NHC catalysts developed to date. The key feature of this class of catalysts is that replacement of the allyl throw-away ligand from the overwhelmingly successful [Pd(NHC)(allyl)Cl] complexes by a bridging chloride imparts a facile activation by dissociation, prevents the formation of off-cycle allyl products, and eliminates synthetic and economic technological issues associated with allyl ligands. These catalysts are highly reactive, easy to prepare, readily activated to Pd(0)–NHC by dimer dissociation (cf. allyl displacement), and avoid cost- and waste-generating allyl ligands. The utility of this class of catalysts has been demonstrated in the synthesis of privileged biaryls and the direct, late-stage functionalization of pharmaceuticals, showing excellent functional group tolerance and chemoselectivity. Computational studies provided key insight into the NHC–Pd(II) chloro dimer activation pathway and rationalized the superior catalytic performance of the dimer catalysts compared with that of the allyl and substituted-allyl palladium catalysts. Crucially, a facile, one-pot synthesis of NHC–Pd(II) chloro dimers has been developed, thus enabling simple and scalable access to [Pd(NHC)(μ-Cl)Cl]_2_ complexes. Overall, the scope of the reactions catalyzed by [Pd(NHC)(μ-Cl)Cl]_2_ complexes supersedes other classes of Pd–NHC catalysts, including activation, rate of cross-coupling of model substrates in different reaction classes, and catalyst synthesis. Our future studies will be focused on expanding the range of transformations mediated by [Pd(NHC)(μ-Cl)Cl]_2_ complexes.

Considering the tremendous impact of Pd-catalyzed cross-coupling reactions in chemical synthesis and the tremendous success of [Pd(NHC)(allyl)Cl] precatalysts by practitioners worldwide, we believe that NHC–Pd(II) chloro dimers, [Pd(NHC)(μ-Cl)Cl]_2_, should be routinely considered as go-to precatalysts of choice in cross-coupling processes. The exceptional performance of [Pd(NHC)(μ-Cl)Cl]_2_ catalysts provides a strong foundation to accelerate applications in the synthesis of medicines, organic molecules, and polymers.

### Limitations of the Study

Limitations are typical to NHC-based catalyst systems and include lower efficiency for highly sterically hindered substrates using IPr ligand and high reaction temperature using ppm catalyst levels. Our future studies will focus on the development of more active ligands and catalysts to expand the scope of application of Pd–NHC catalysis.

### Resource Availability

#### Lead Contact

Further information and requests for resources and reagents should be directed to and will be fulfilled by the Lead Contact, Michal Szostak (michal.szostak@rutgers.edu).

#### Materials Availability

This study did not generate new unique reagents.

#### Data and Code Availability

The published article includes all data generated during this study.

## Methods

All methods can be found in the accompanying Transparent Methods supplemental file.

## Supplemental Information

Document S1. Transparent Methods, Figures S1–S113, Scheme S1–S8, and Tables S1 and S2

Data S1. Cartersian Coordinates and Energies, Related to Figure 4

## Footnotes

Supplemental Information can be found online at https://doi.org/10.1016/j.isci.2020.101377.
