# Supplementary material for: [Pd(NHC)(μ-Cl)Cl]2: Versatile and Highly Reactive Complexes for Cross-Coupling Reactions that Avoid Formation of Inactive Pd(I) Off-Cycle Products
Source: iScience. 2020 Jul 16;23(8):101377. doi: 10.1016/j.isci.2020.101377 (PMC7404551; doi:10.1016/j.isci.2020.101377)
Supplement: Document S1. Transparent Methods, Figures S1–S113, Scheme S1–S8, and Tables S1 and S2 [file mmc1.pdf]

iScience, Volume *23*

Supplemental Information

[Pd(NHC)(μ-Cl)Cl]$_2$: Versatile and Highly Reactive Complexes for Cross-Coupling Reactions that Avoid Formation of Inactive Pd(I) Off-Cycle Products

Tongliang Zhou, Siyue Ma, Fady Nahra, Alan M.C. Obled, Albert Poater, Luigi Cavallo, Catherine S.J. Cazin, Steven P. Nolan, and Michal Szostak

Figure S1. Full DFT-optimized pathway (relative energies to Pd(0) in kcal/mol) for the activation of catalysts **6** (black), **11** (red) and **12** (blue); including the release of PhCl and PhPh, Related to **Figure 4**.

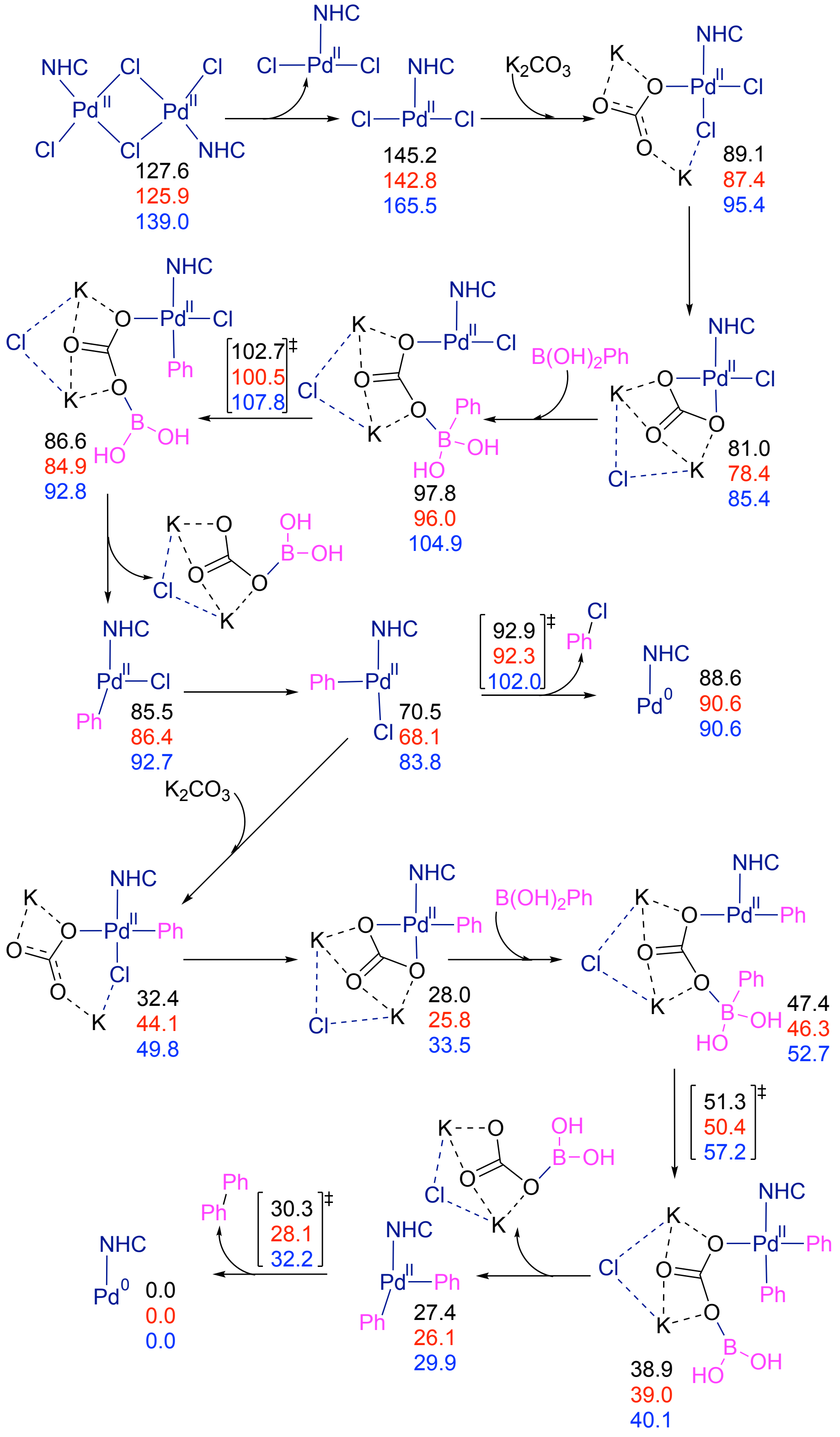

Figure S2. DFT-optimized pathway for the activation of catalysts **1** and **2** (relative energies to Pd(0) in kcal/mol), Related to **Figure 4**.

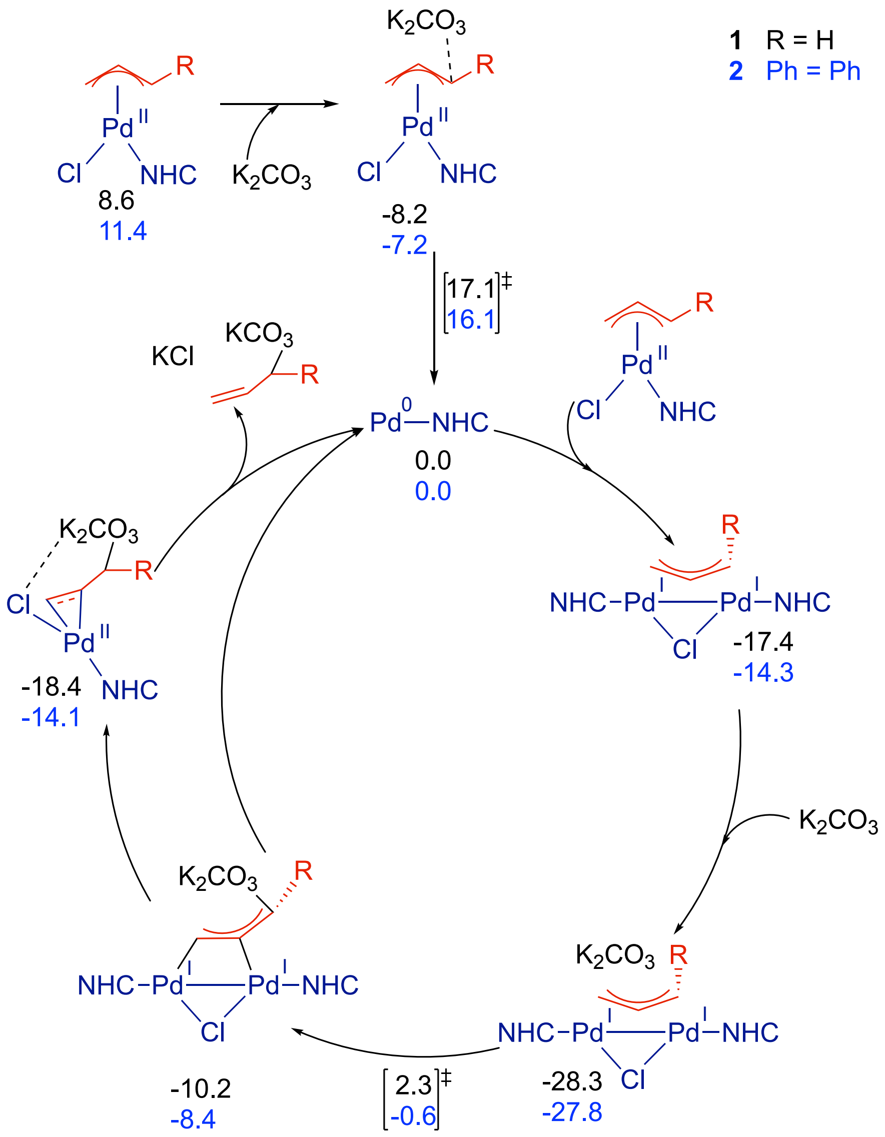

Figure S3. ^{1}H NMR spectrum of phenyl(*p*-tolyl)methanone, related to **Scheme 1**

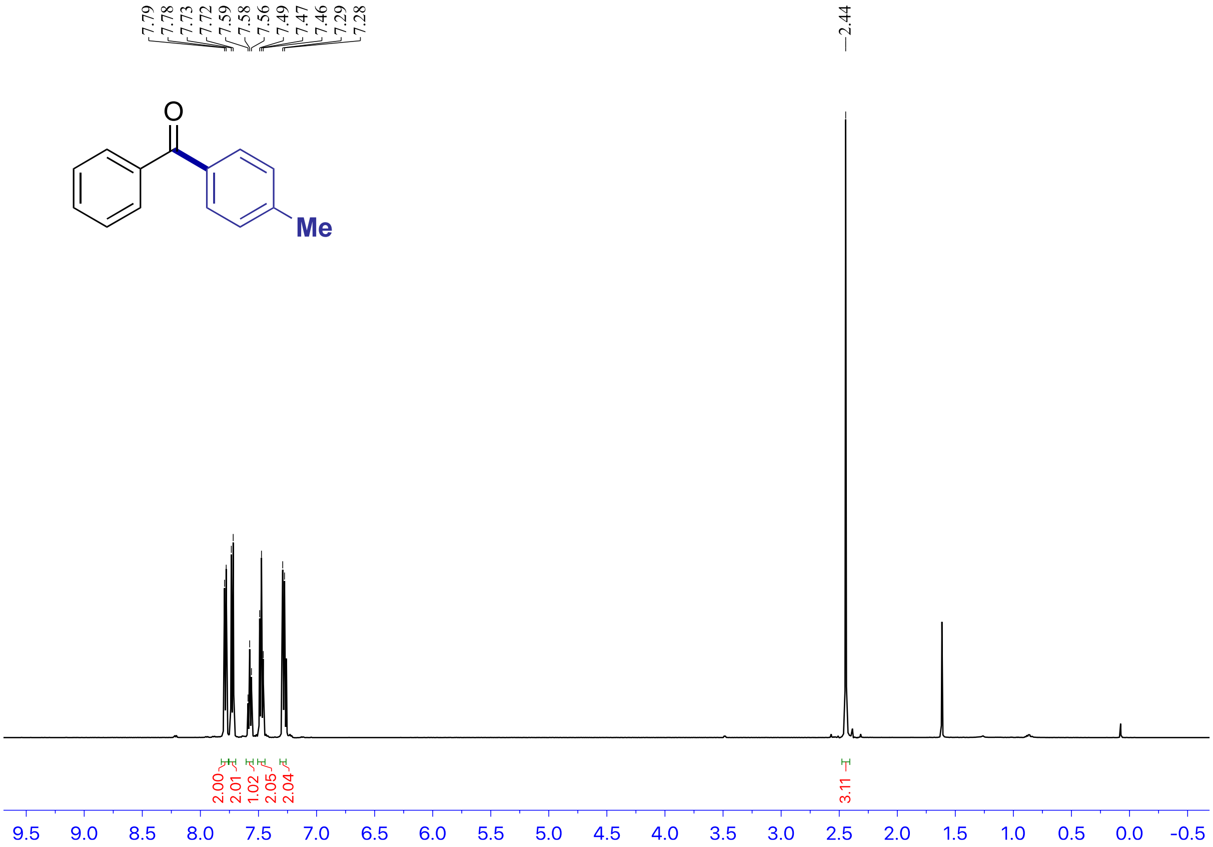

Figure S4. ^{13}C NMR spectrum of phenyl(*p*-tolyl)methanone, related to **Scheme 1**

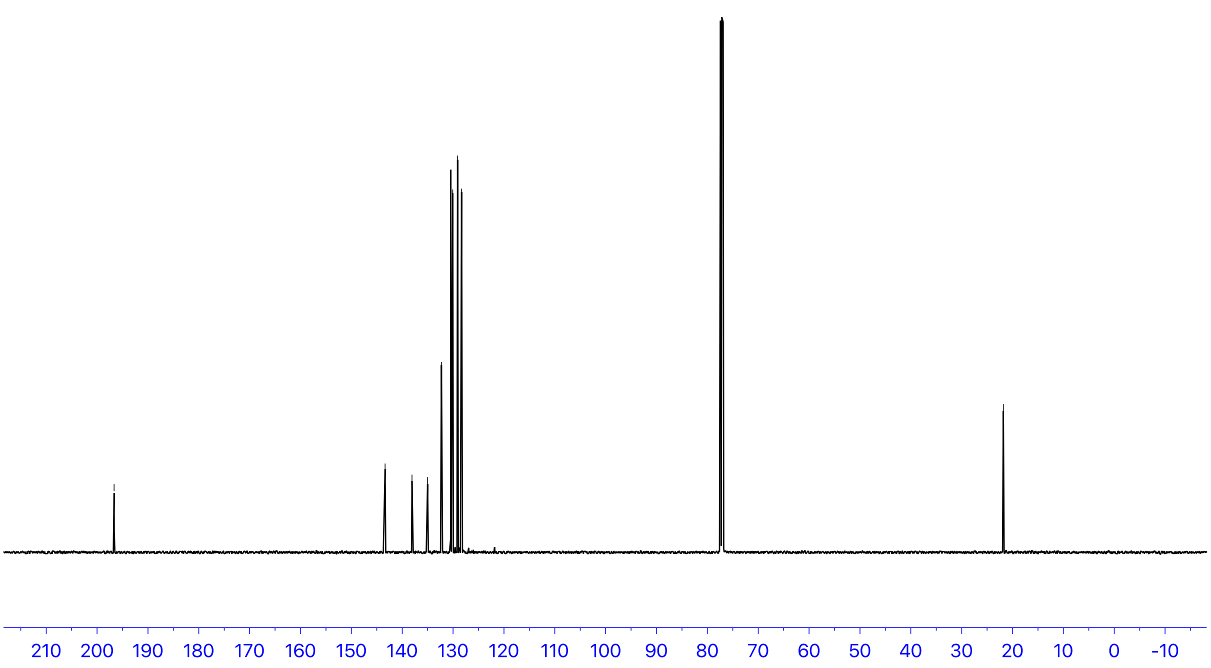

Figure S5. ^{1}H NMR spectrum of (4-methoxyphenyl)(phenyl)methanone, related to **Scheme 1**

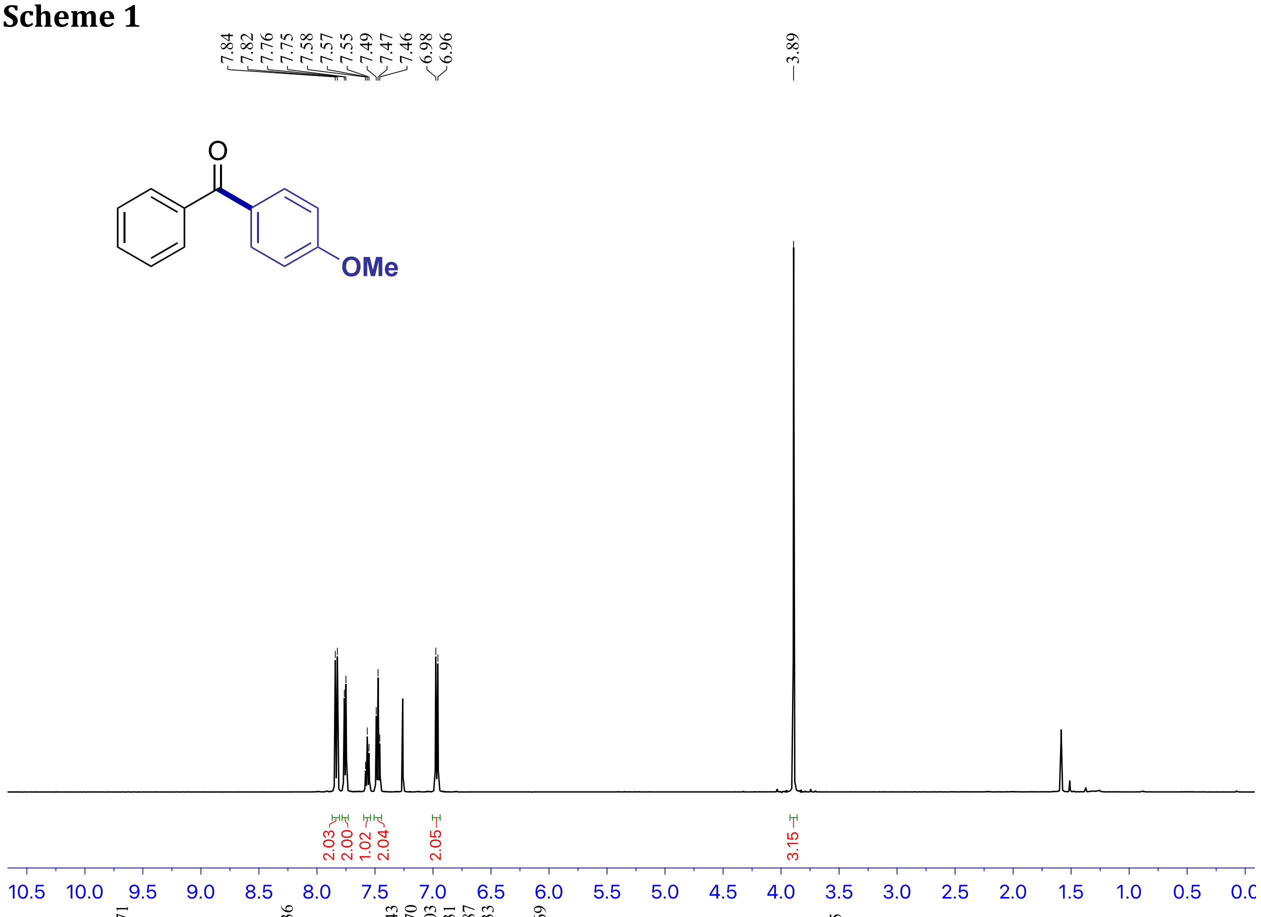

Figure S6. ^{13}C NMR spectrum of (4-methoxyphenyl)(phenyl)methanone, related to **Scheme 1**

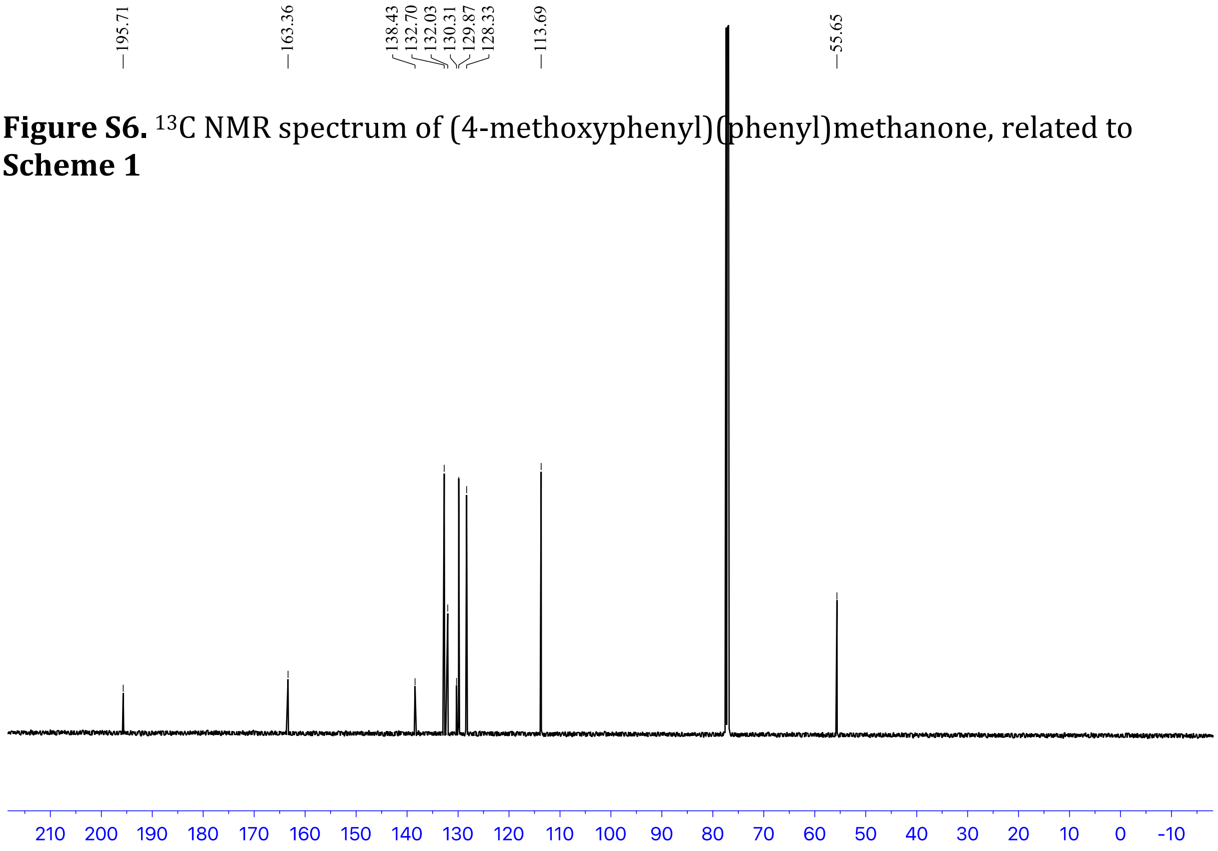

Figure S7. ^{1}H NMR spectrum of phenyl(4-(trifluoromethyl)phenyl)methanone, related to **Scheme 1**

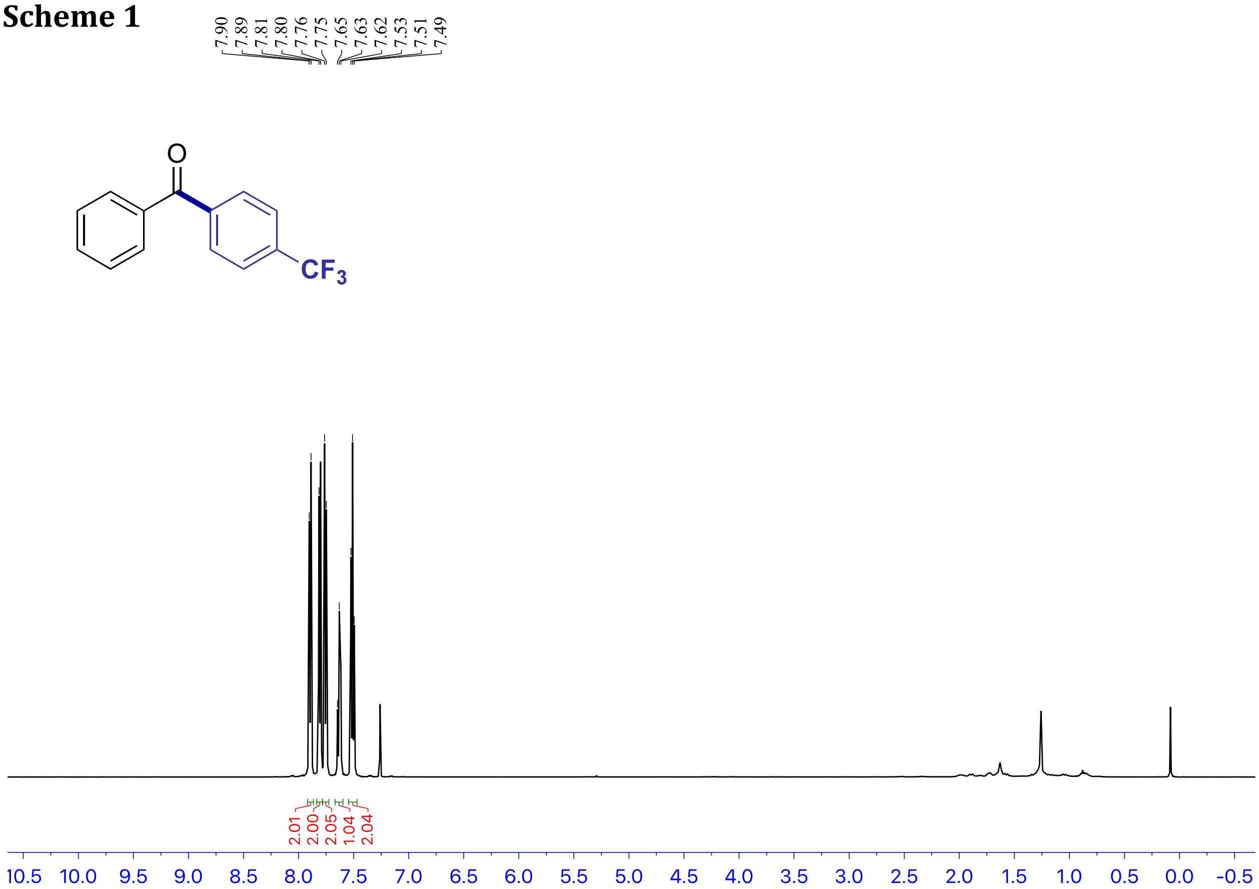

Figure S8. ^{13}C NMR spectrum of phenyl(4-(trifluoromethyl)phenyl)methanone, related to **Scheme 1**

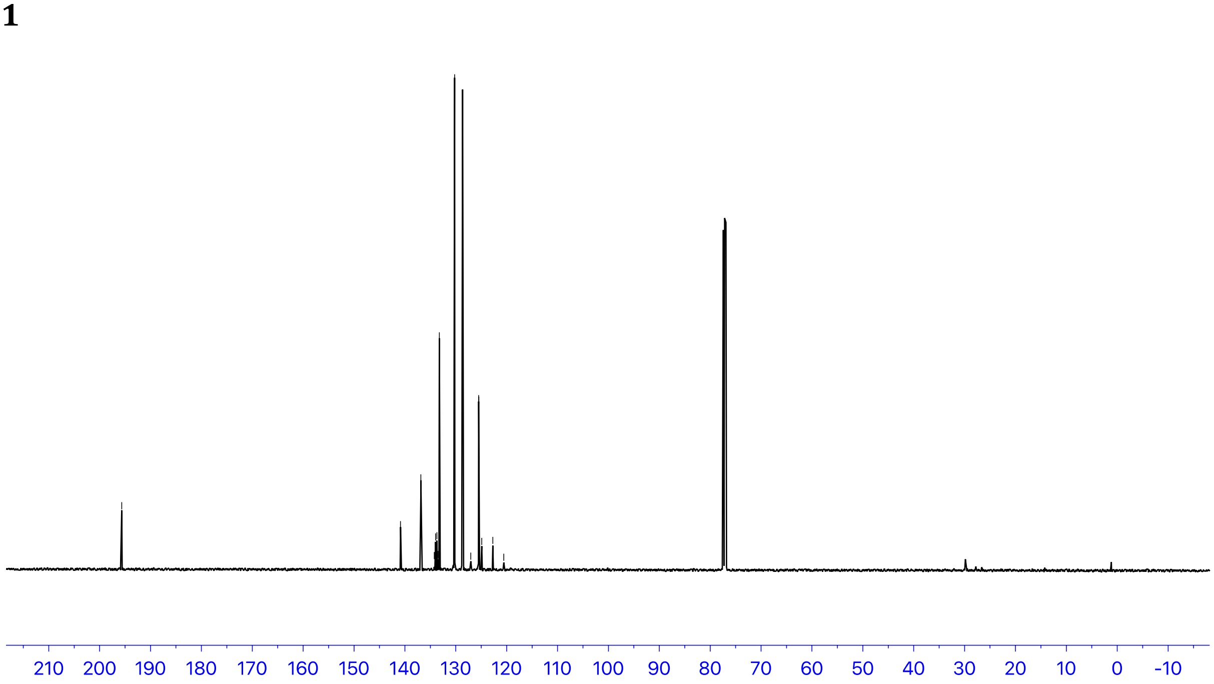

Figure S9. ^{19}F NMR spectrum of phenyl(4-(trifluoromethyl)phenyl)methanone, related to **Scheme 1**

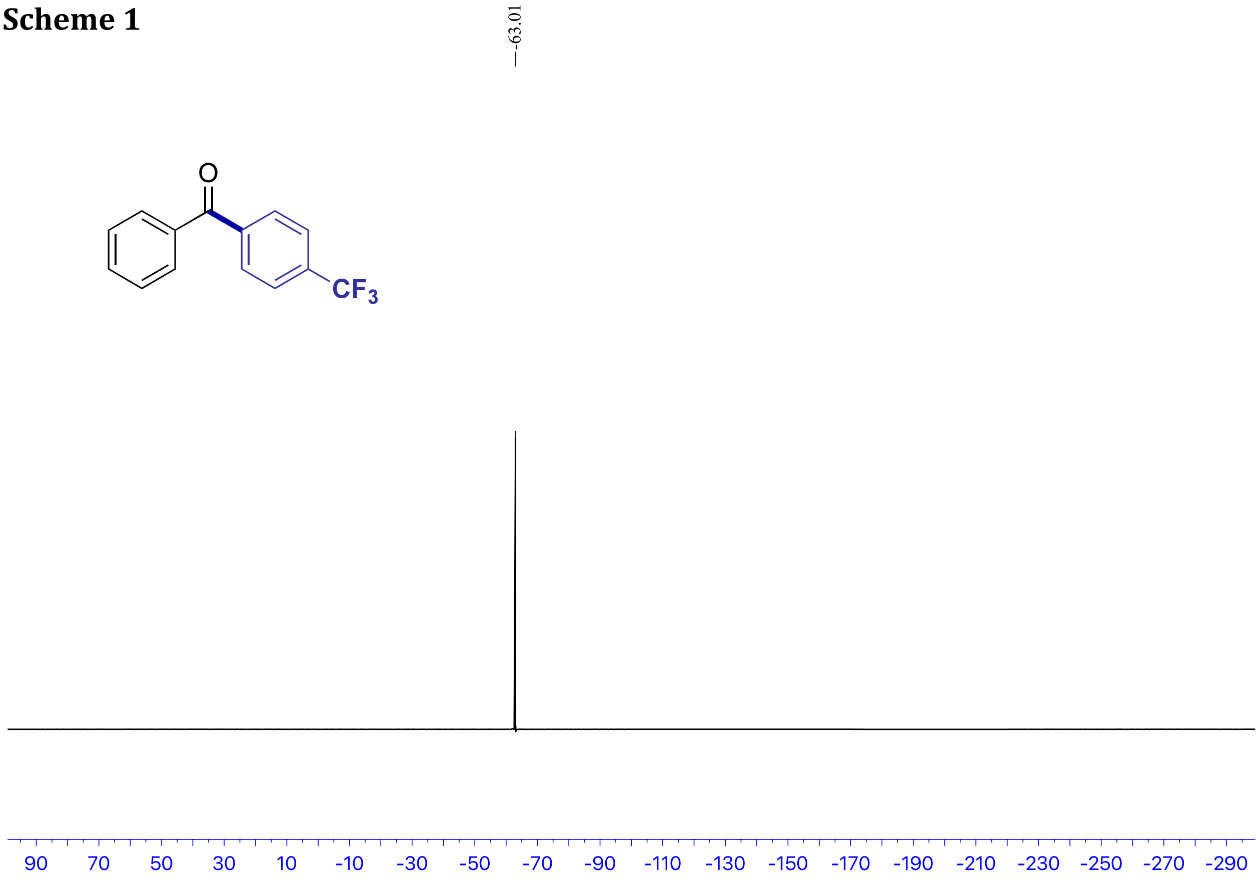

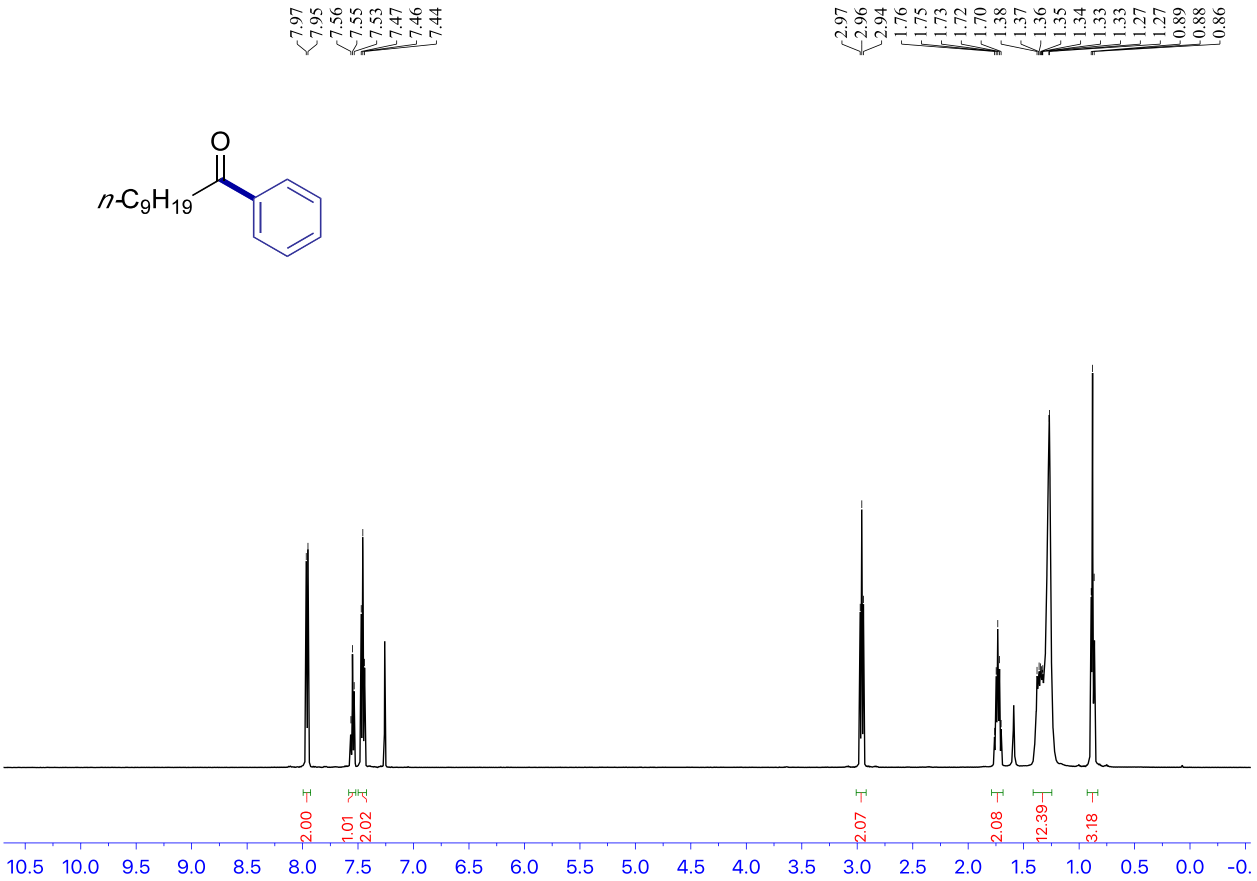

7.97
7.95
7.56
7.55
7.53
7.47
7.46
7.44
2.97
2.96
2.94
1.76
1.75
1.73
1.72
1.70
1.38
1.37
1.36
1.35
1.34
1.33
1.33
1.27
1.27
0.89
0.88
0.86
O
n-C9H19
2.00
1.01
2.02
2.07
2.08
12.39
3.18
10.5 10.0 9.5 9.0 8.5 8.0 7.5 7.0 6.5 6.0 5.5 5.0 4.5 4.0 3.5 3.0 2.5 2.0 1.5 1.0 0.5 0.0

Figure S11. ^{13}C NMR spectrum of 1-phenyldecan-1-one, related to **Scheme 1**

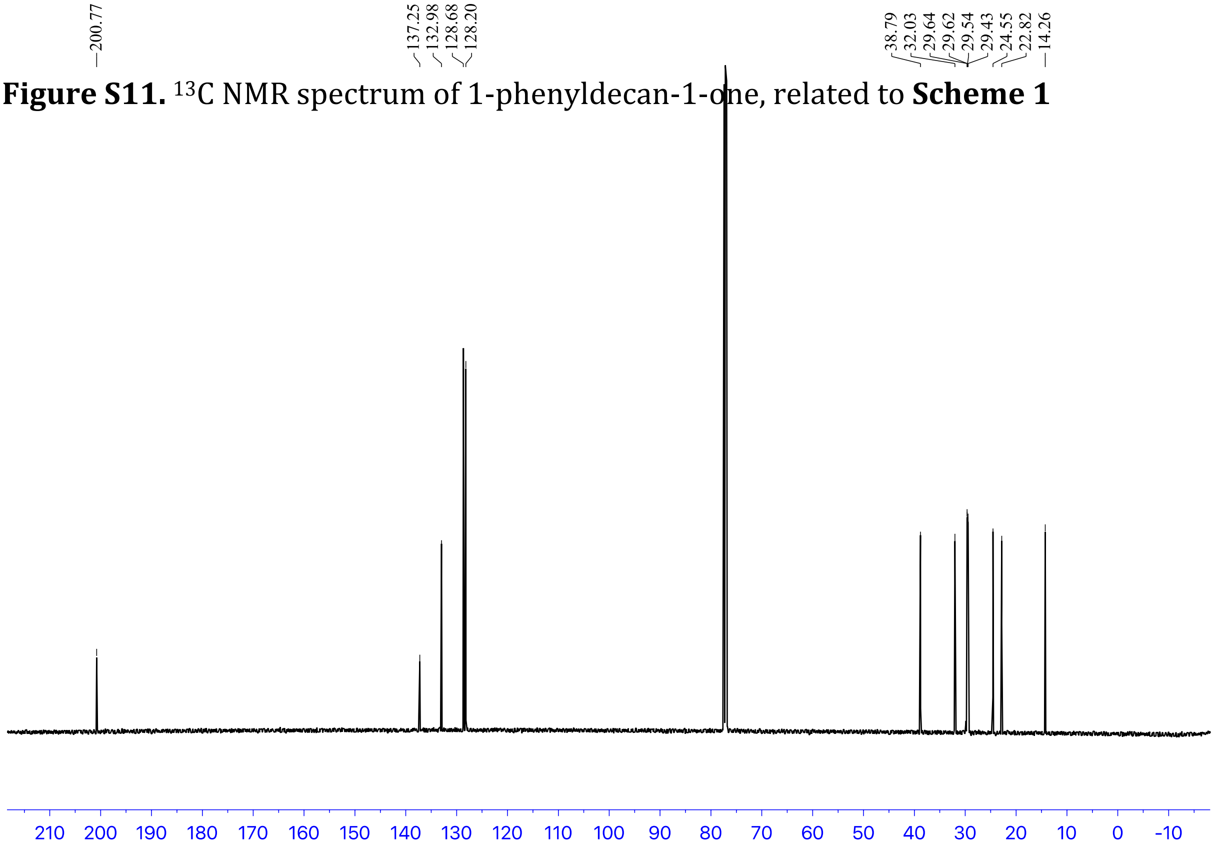

Figure S12. ^{1}H NMR spectrum of methyl 4-benzoylbenzoate, related to **Scheme 1**

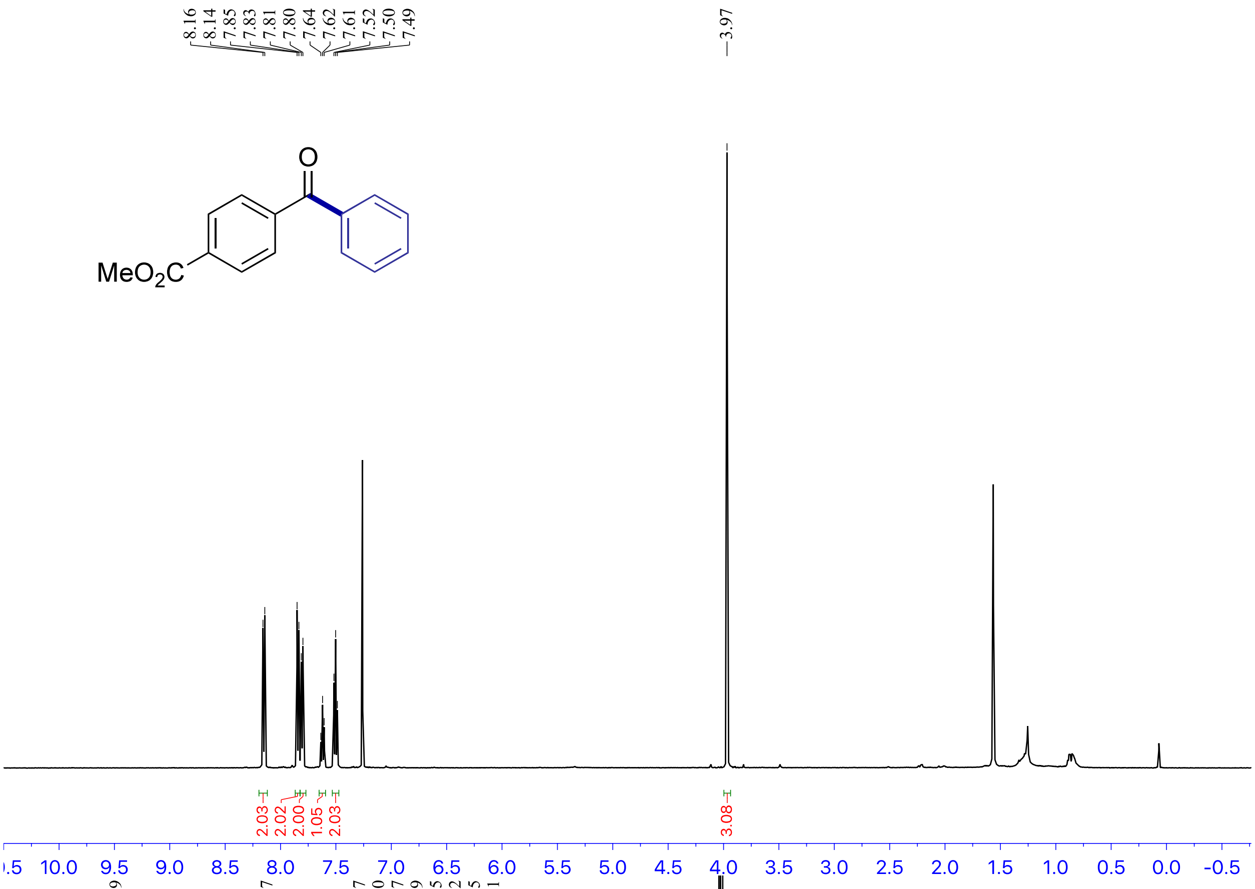

Figure S13. ^{13}C NMR spectrum of methyl 4-benzoylbenzoate, related to **Scheme 1**

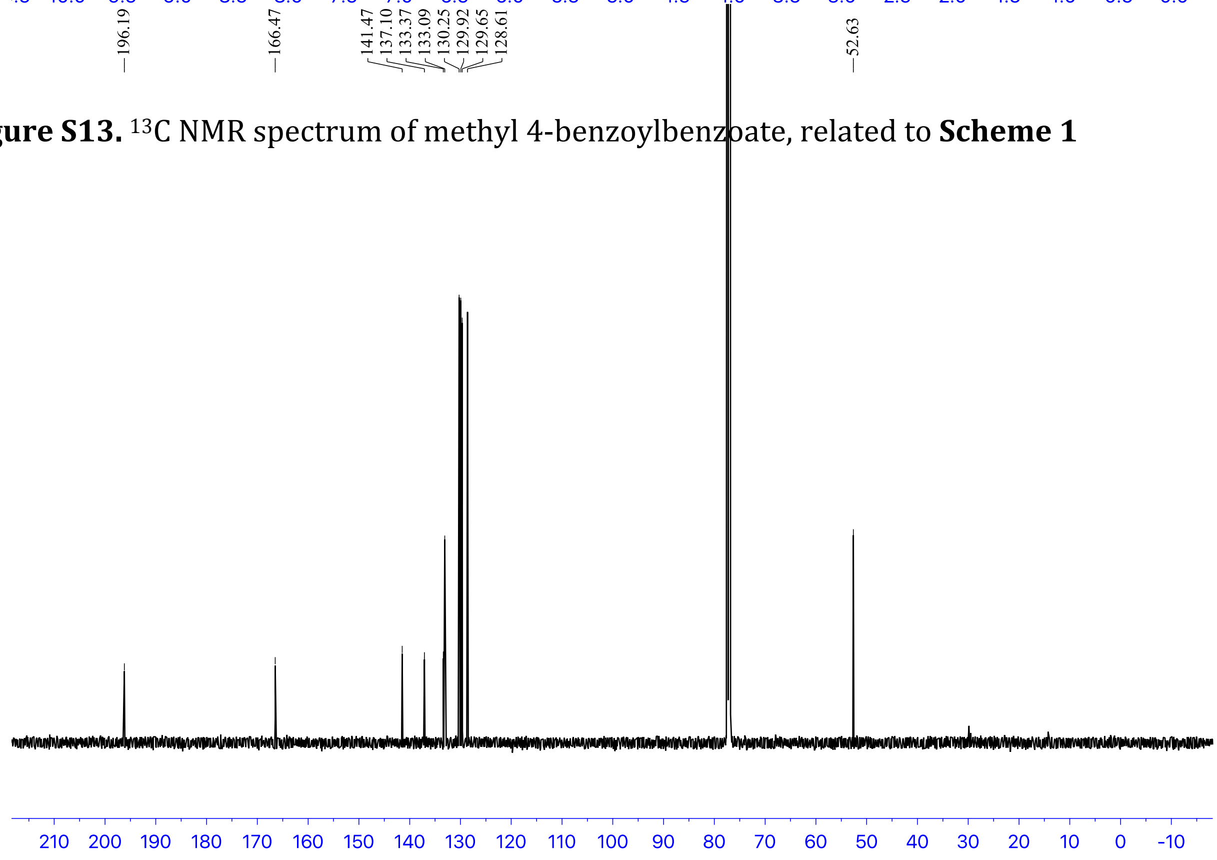

Figure S14. ^{1}H NMR spectrum of phenyl(*o*-tolyl)methanone, related to **Scheme 1**

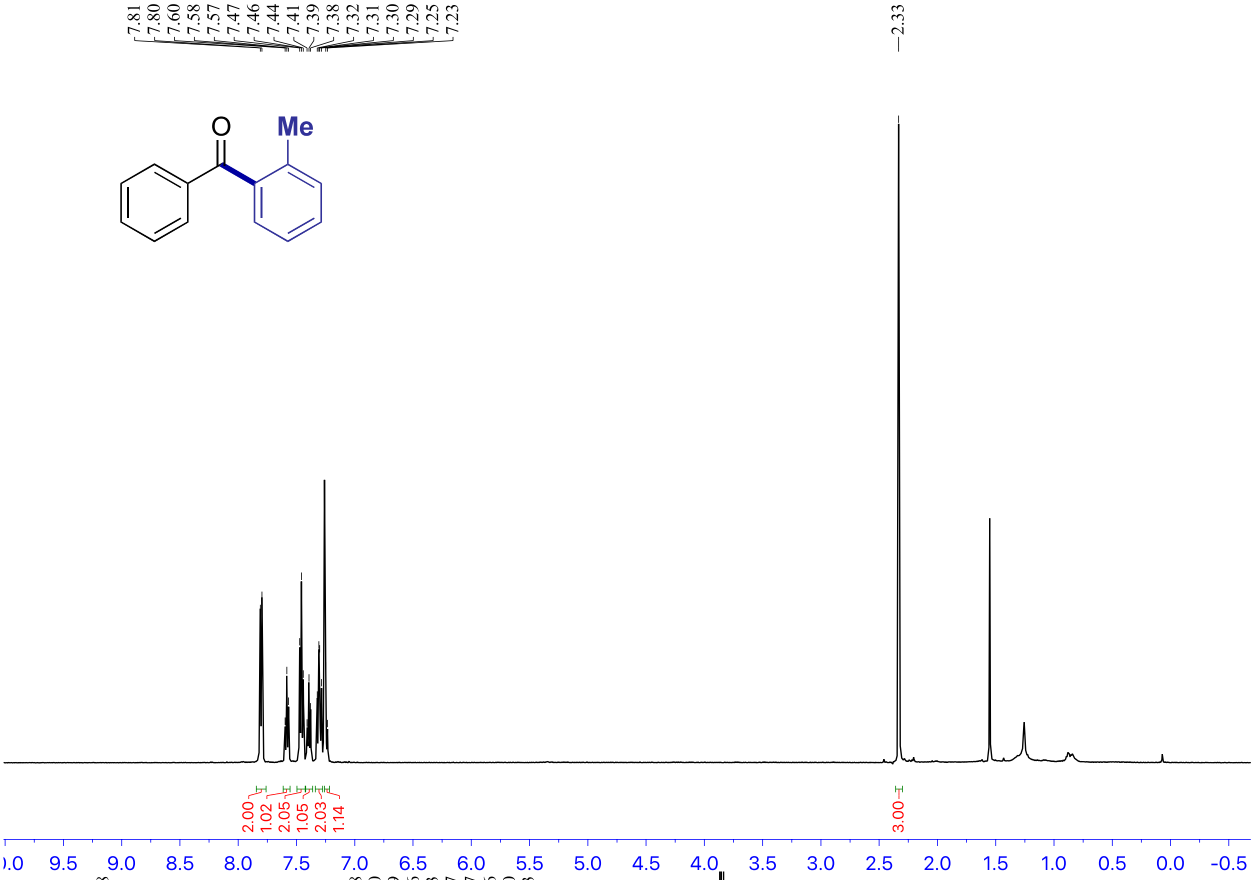

Figure S15. ^{13}C NMR spectrum of phenyl(*o*-tolyl)methanone, related to **Scheme 1**

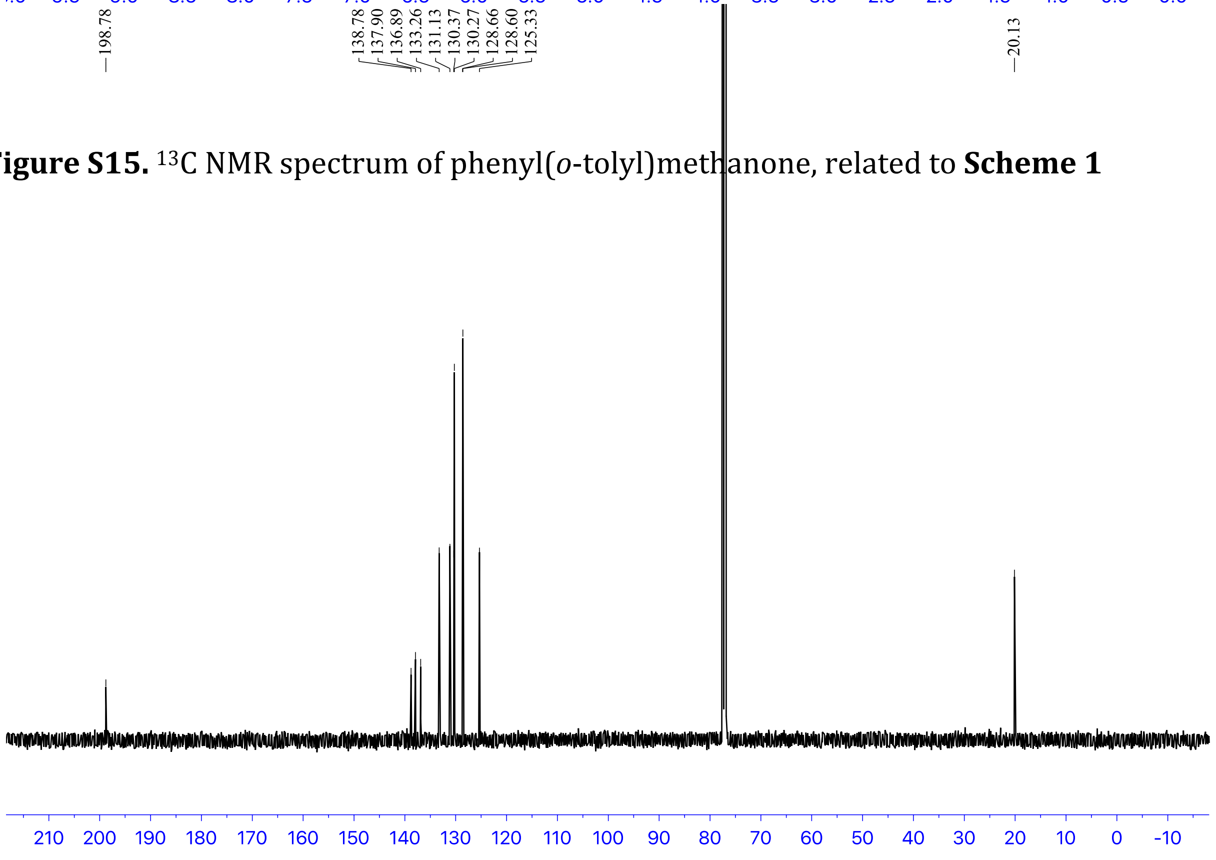

Figure S16. ^{1}H NMR spectrum of 4-methylbiphenyl, related to **Scheme 2**

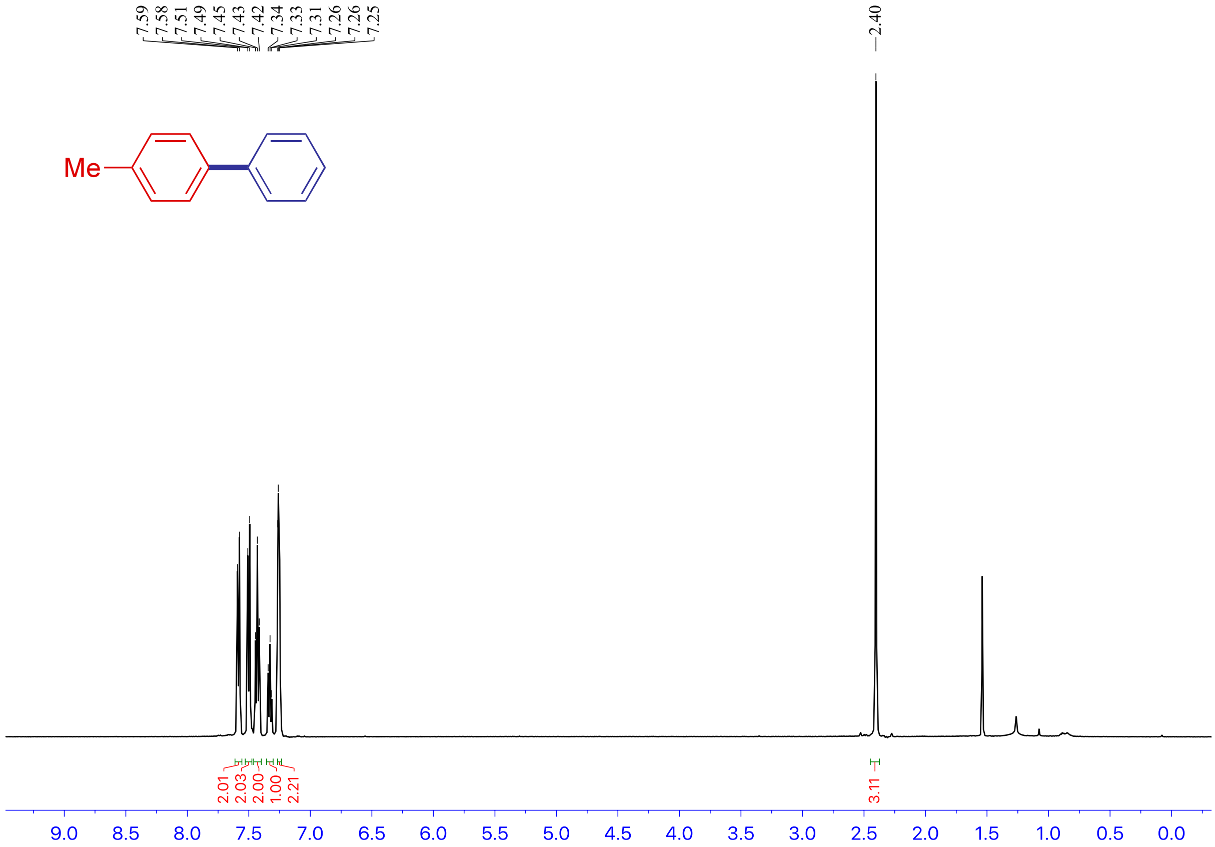

Figure S17. ^{13}C NMR spectrum of 4-methylbiphenyl, related to **Scheme 2**

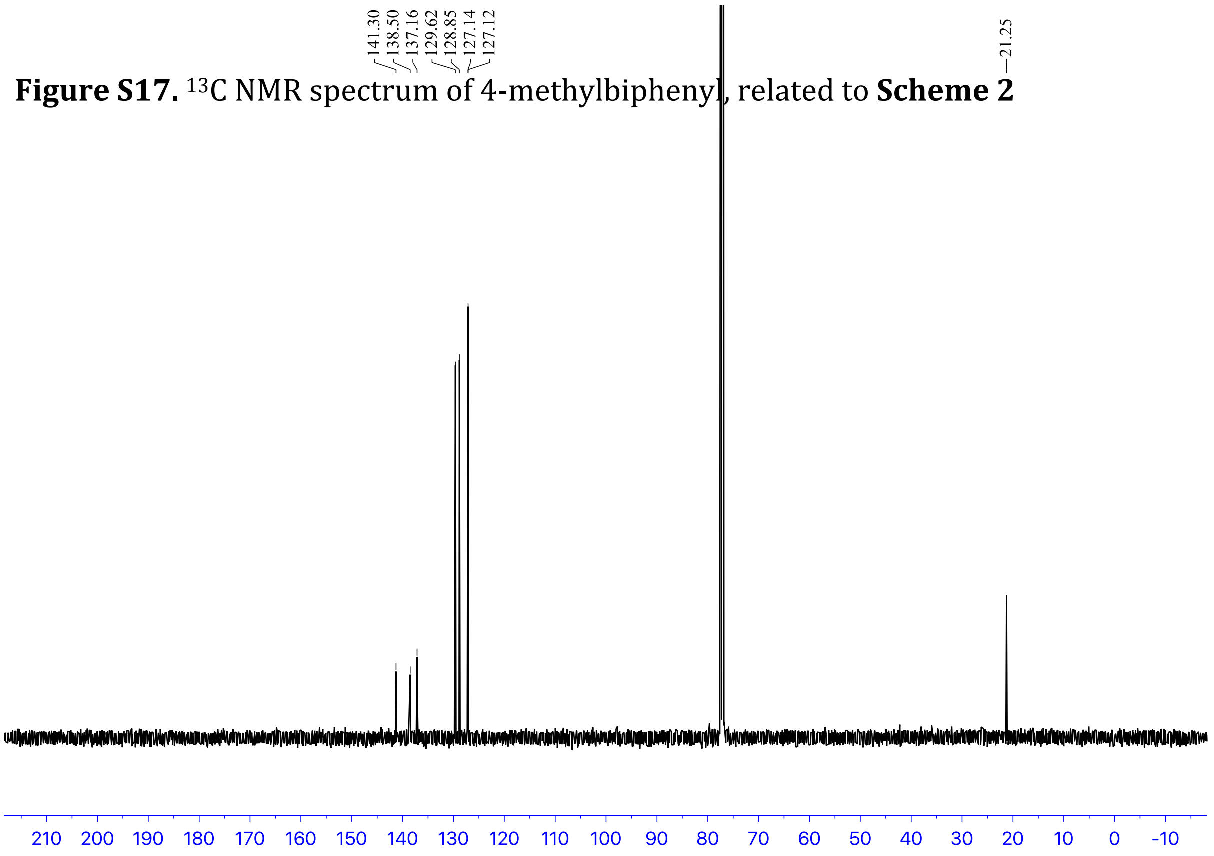

Figure S18. ^{1}H NMR spectrum of 4-methoxybiphenyl, related to **Scheme 2**

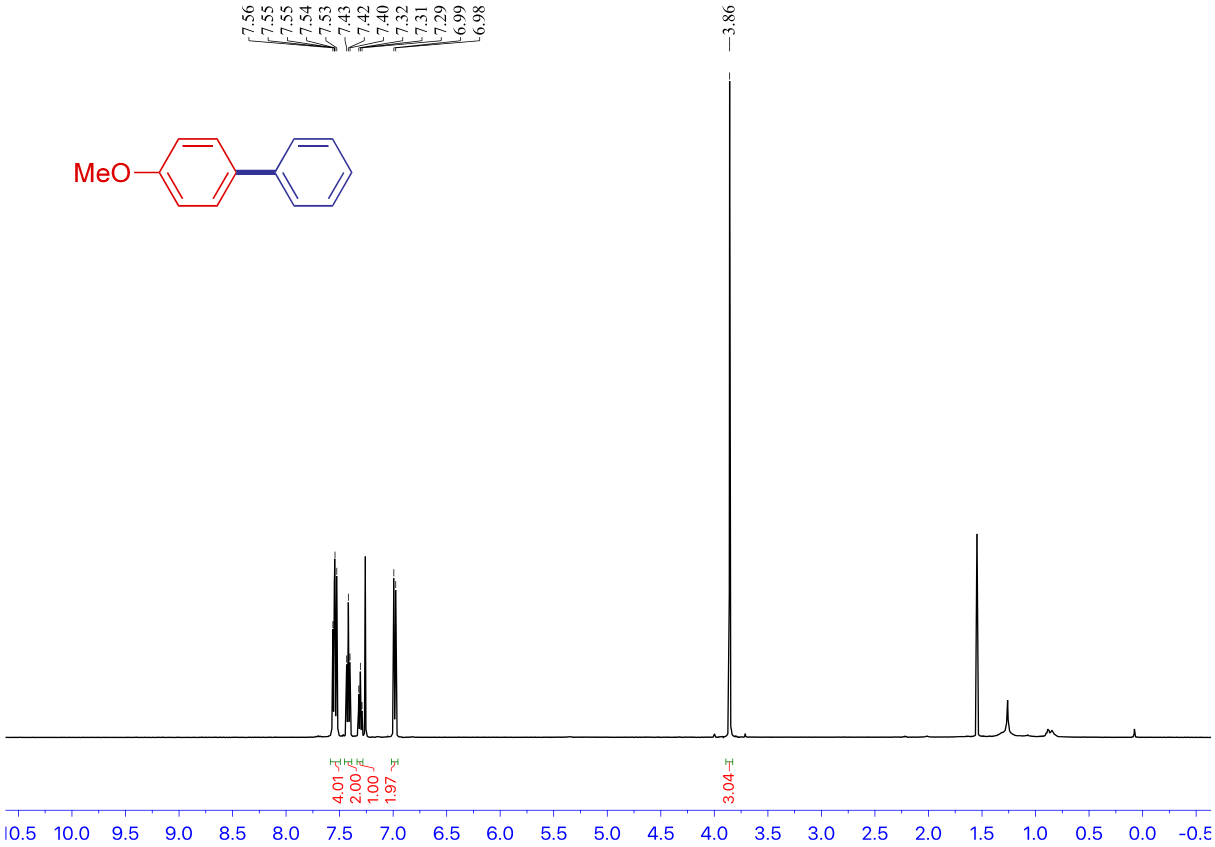

Figure S19. ^{13}C NMR spectrum of 4-methoxybiphenyl, related to **Scheme 2**

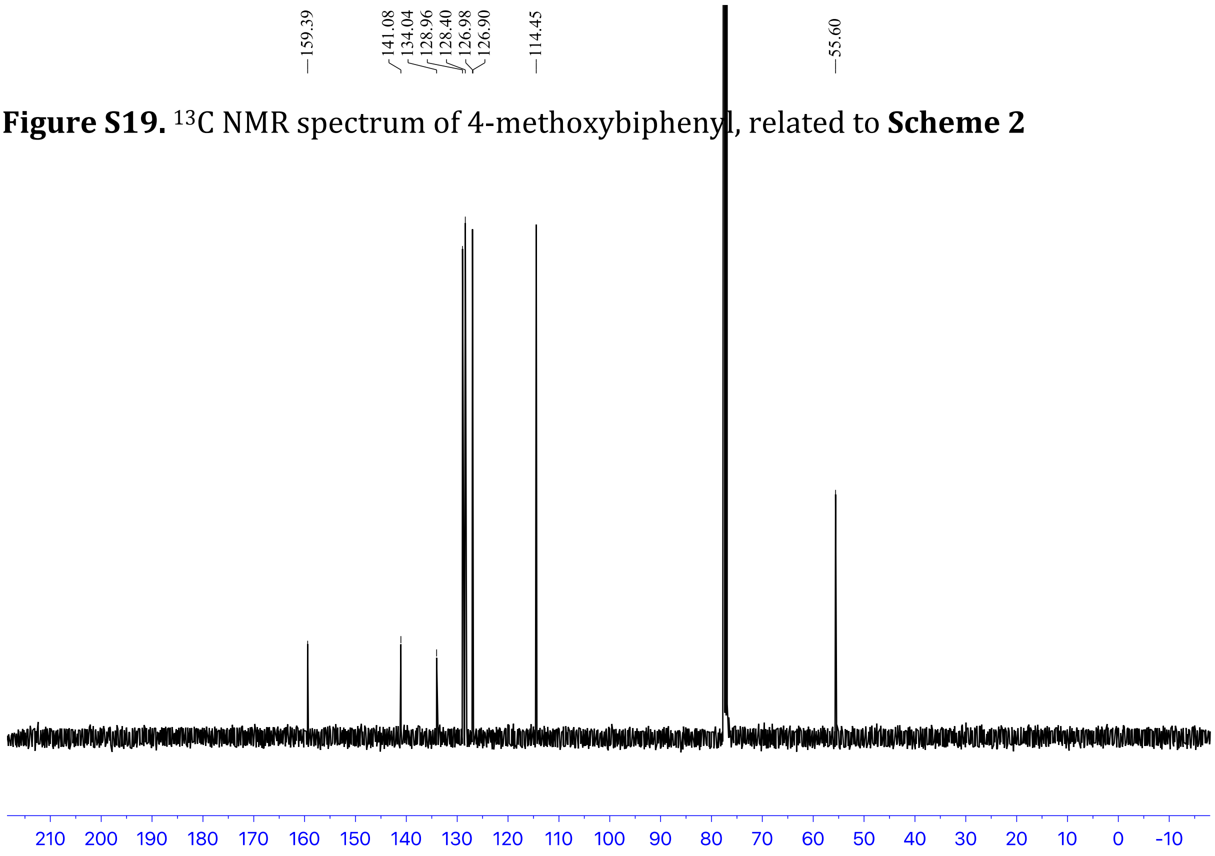

Figure S20. ^{1}H NMR spectrum of 4'-methoxy-2,6-dimethylbiphenyl, related to **Scheme 2**

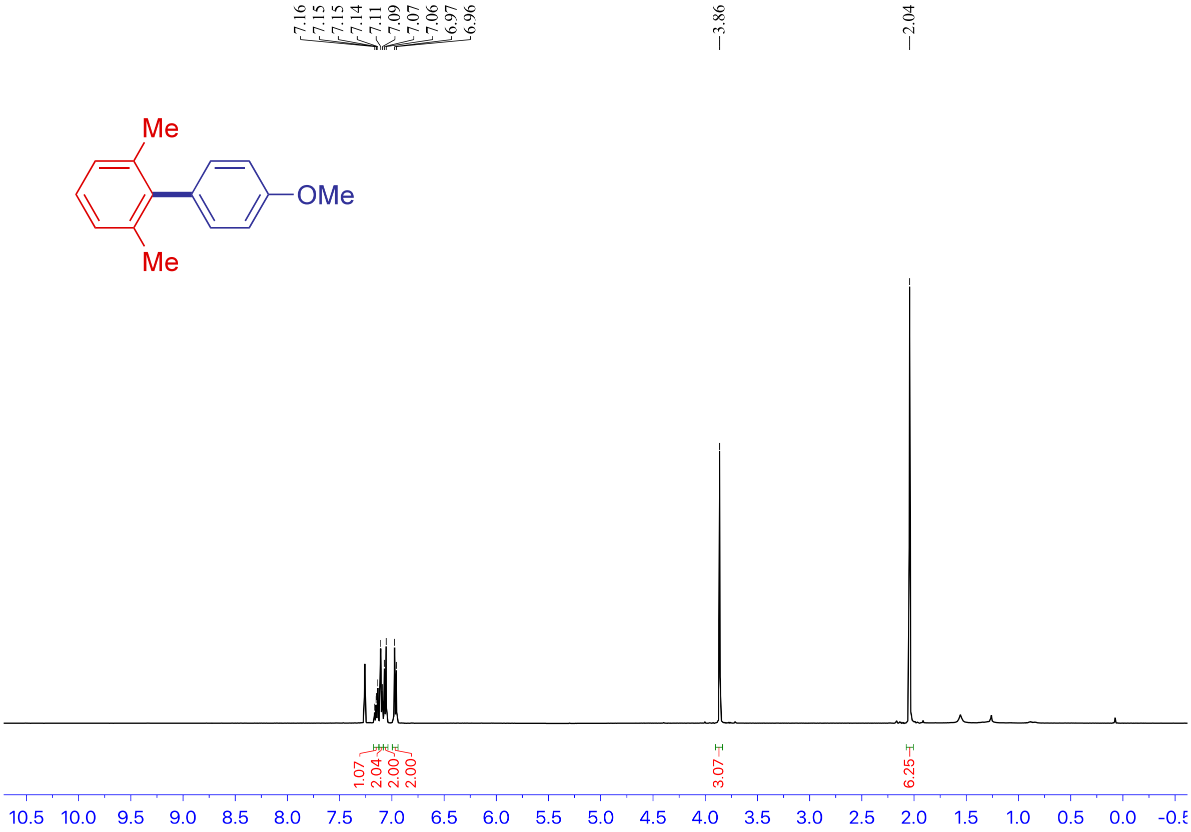

Figure S21. ^{13}C NMR spectrum of 4'-methoxy-2,6-dimethylbiphenyl, related to **Scheme 2**

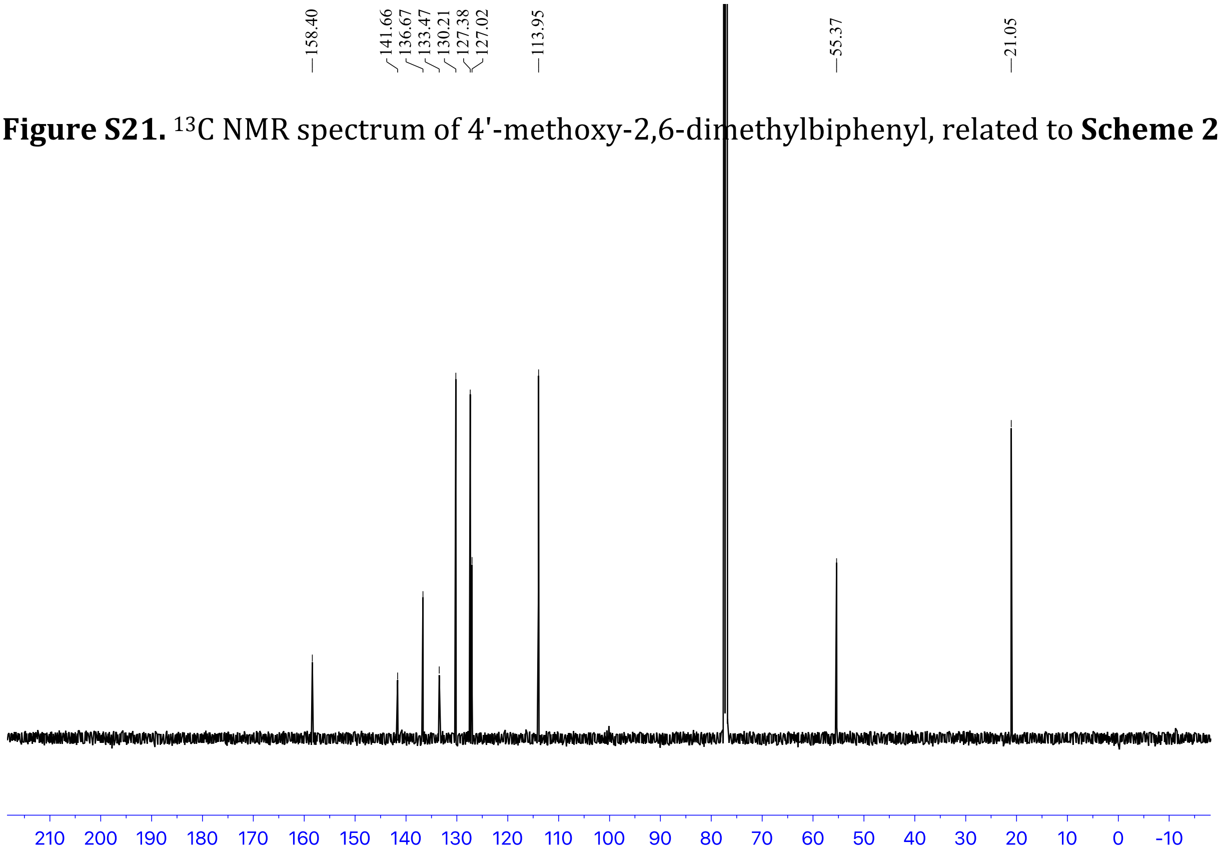

Figure S22. ^{1}H NMR spectrum of 4-cyano-4'-methylbiphenyl, related to **Scheme 2**

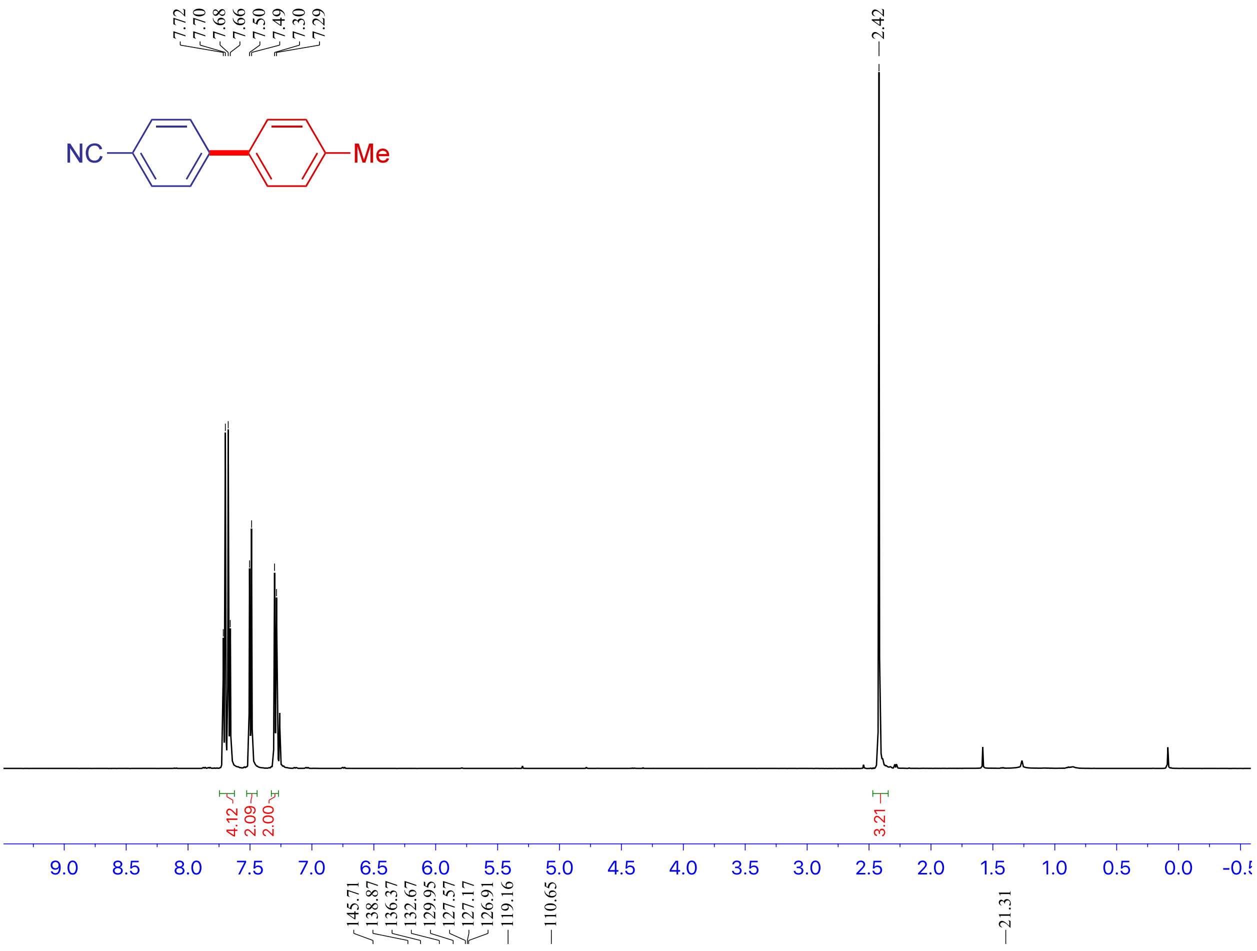

Figure S23. ^{13}C NMR spectrum of 4-cyano-4'-methylbiphenyl, related to **Scheme 2**

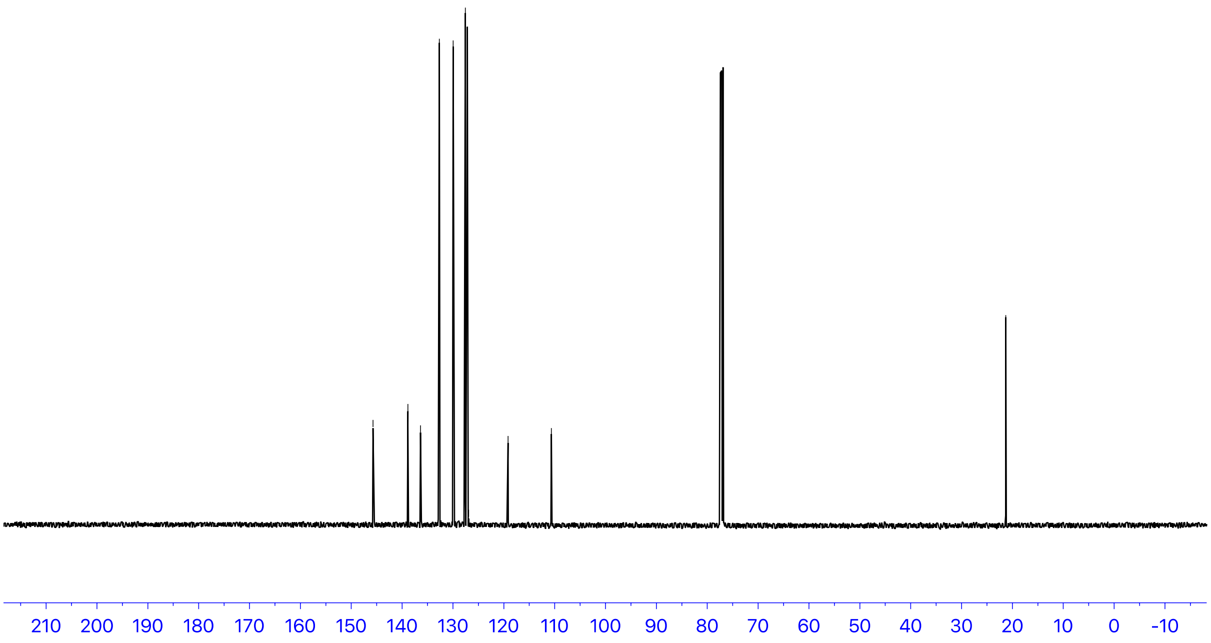

Figure S24. ^{1}H NMR spectrum of 4-hydroxy-4'-methylbiphenyl, related to **Scheme 2**

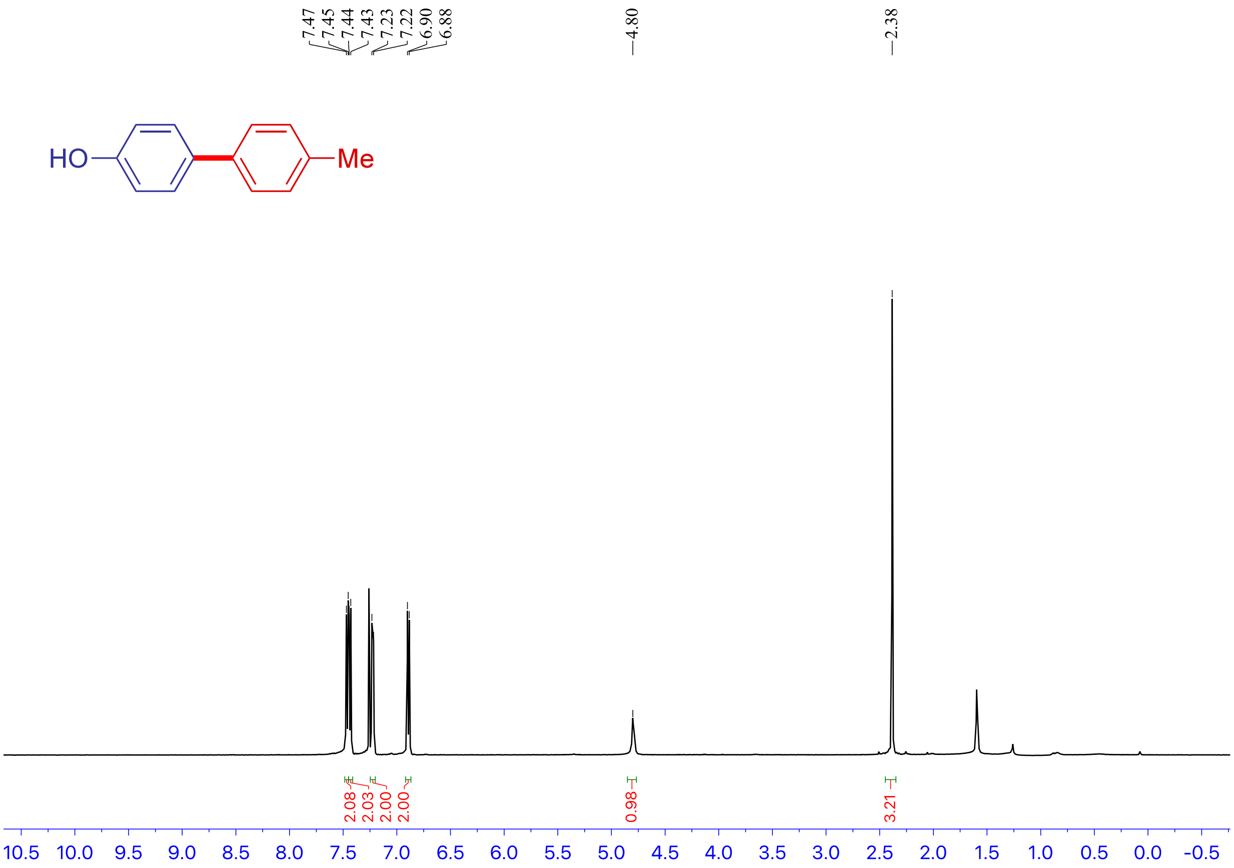

Figure S25. ^{13}C NMR spectrum of 4-hydroxy-4'-methylbiphenyl, related to **Scheme 2**

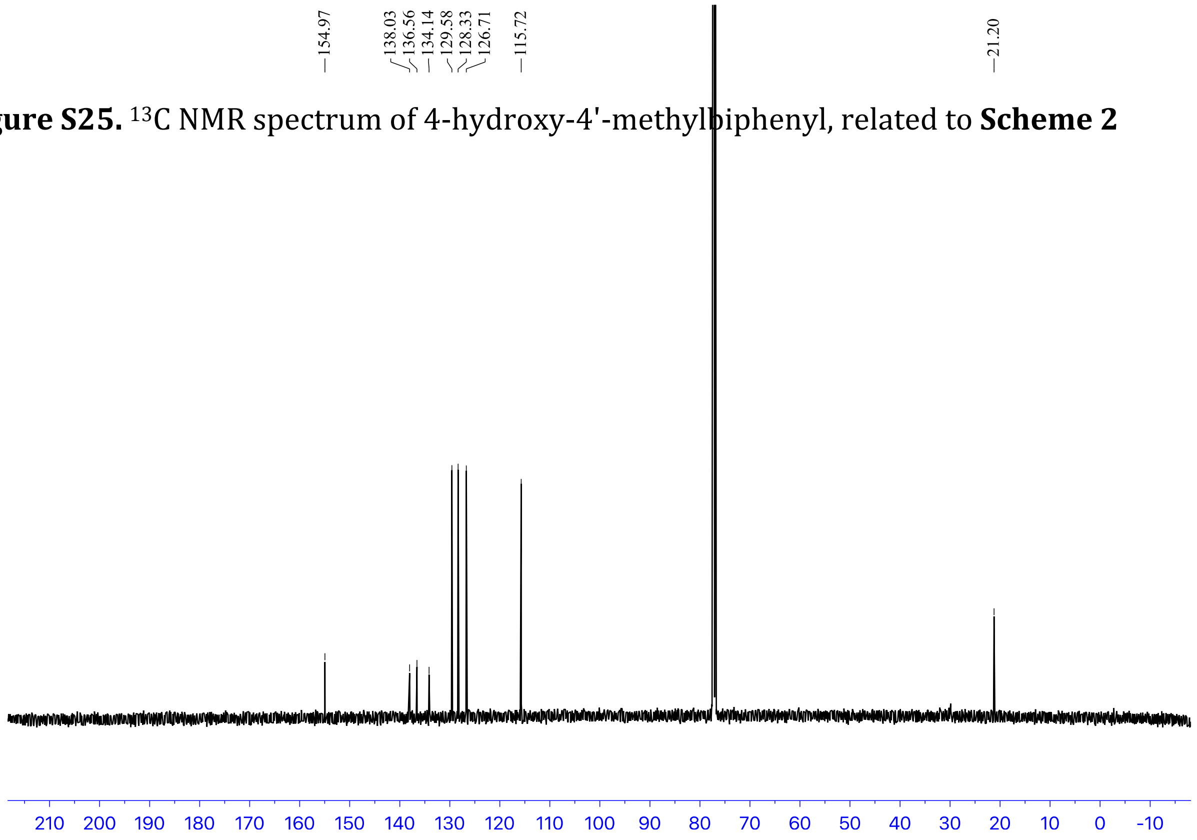

Figure S26. ^{1}H NMR spectrum of 4-amino-4'-methylbiphenyl, related to **Scheme 2**

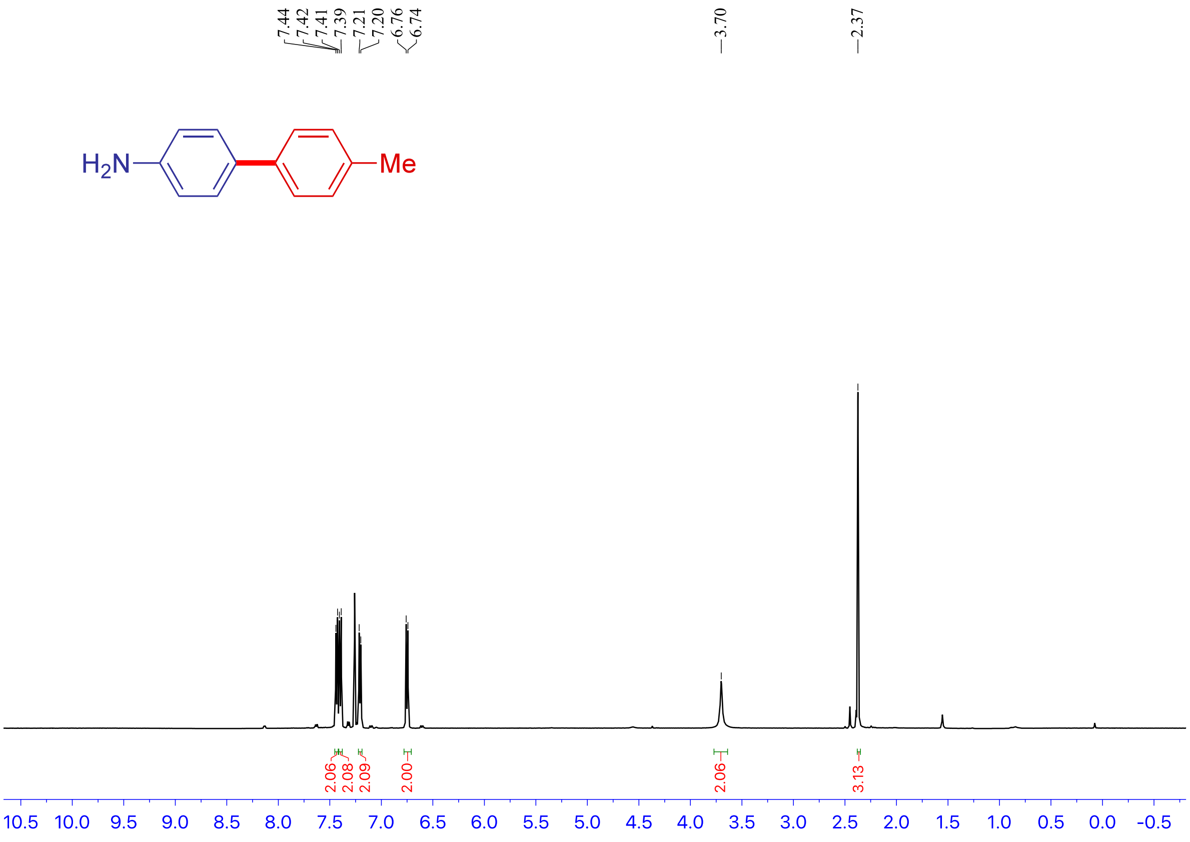

Figure S27. ^{13}C NMR spectrum of 4-amino-4'-methylbiphenyl, related to **Scheme 2**

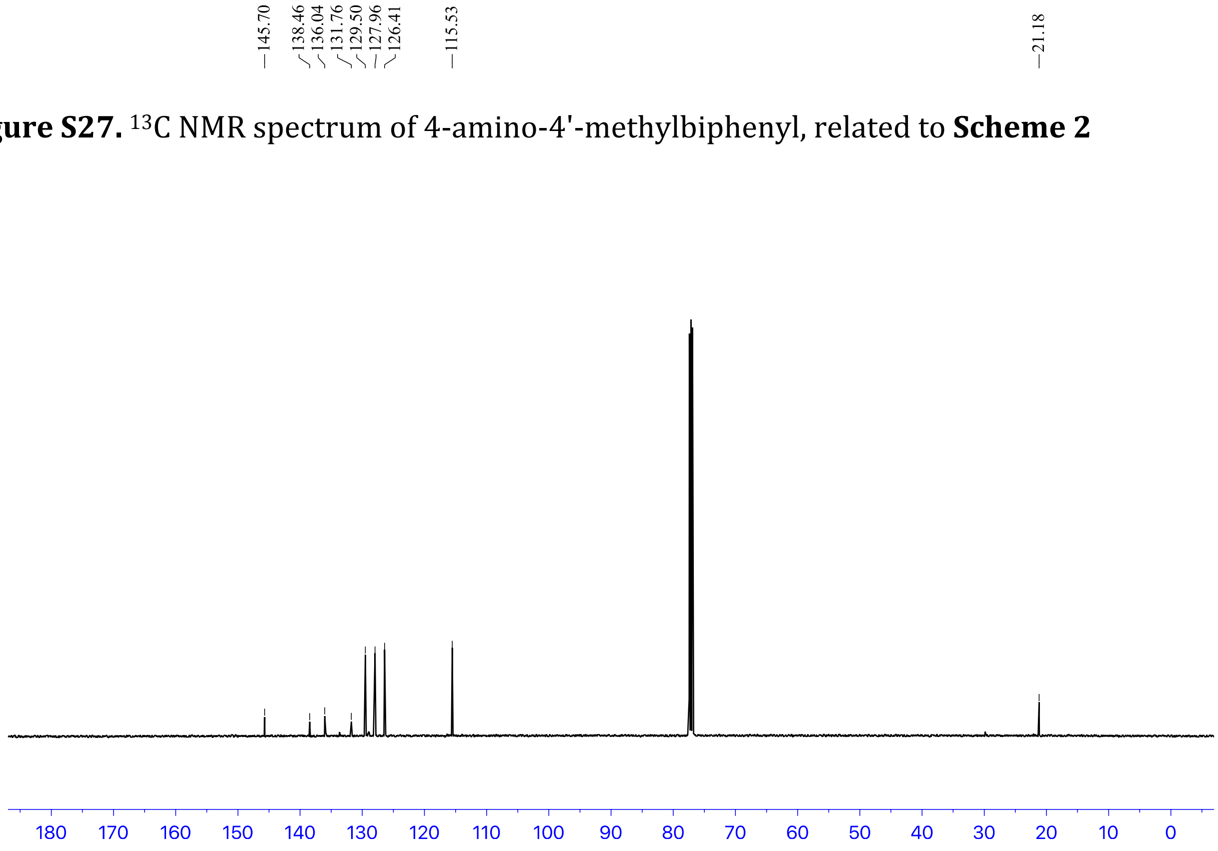

Figure S28. ^{1}H NMR spectrum of 2-amino-4'-methylbiphenyl, related to **Scheme 2**

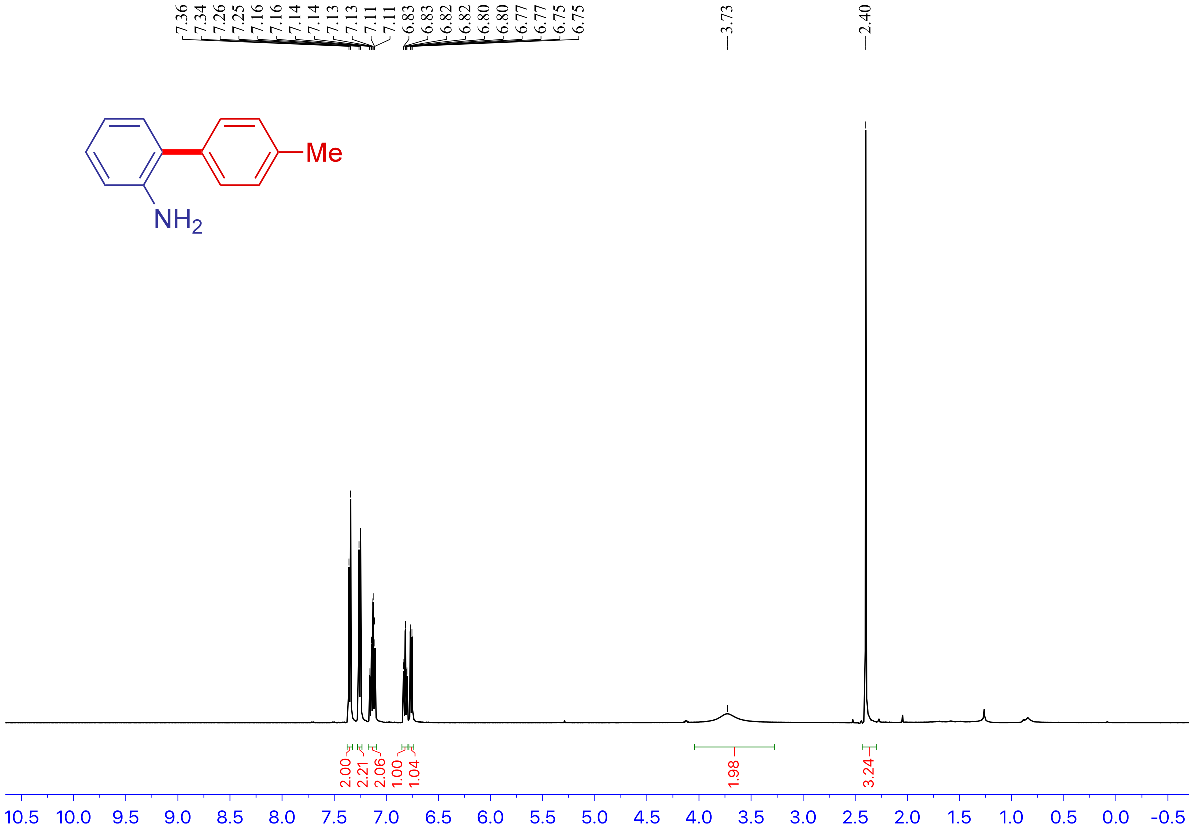

Figure S29. ^{13}C NMR spectrum of 2-amino-4'-methylbiphenyl, related to **Scheme 2**

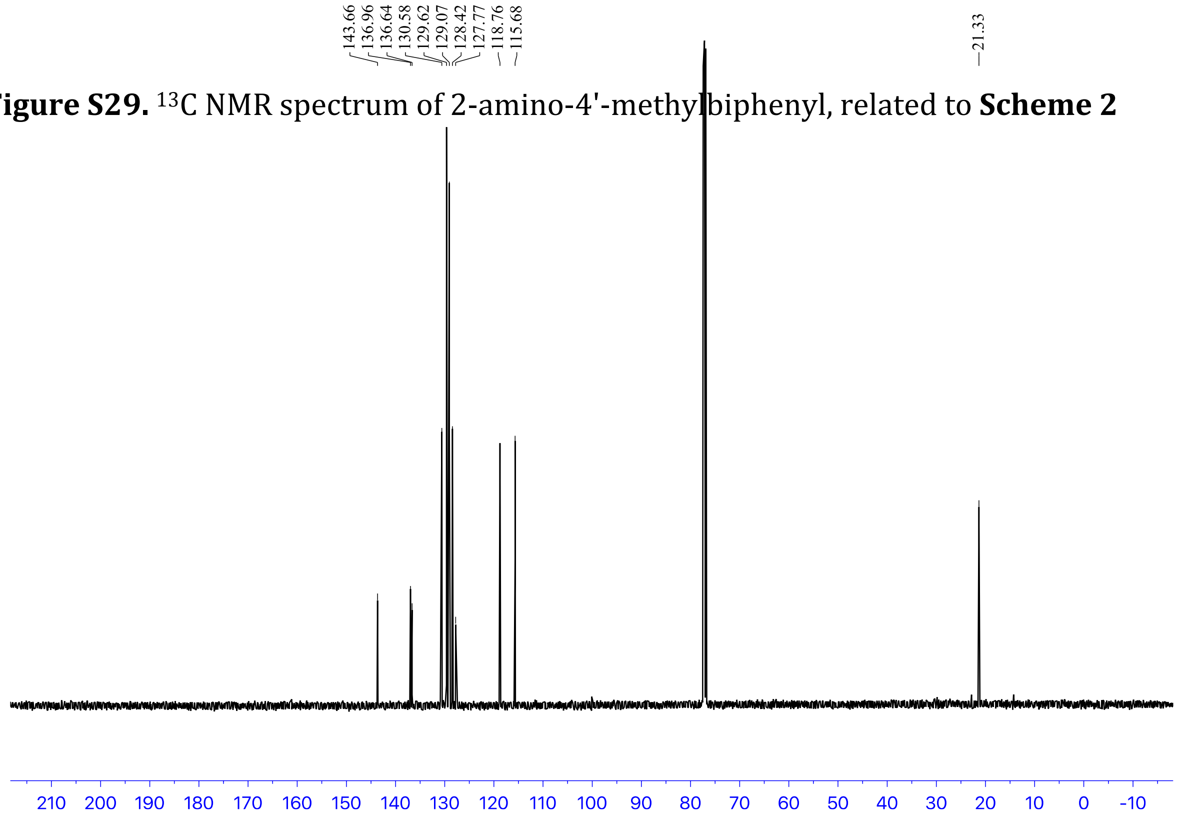

Figure S30. ^{1}H NMR spectrum of 2-(*p*-tolyl)pyridine, related to **Scheme 2**

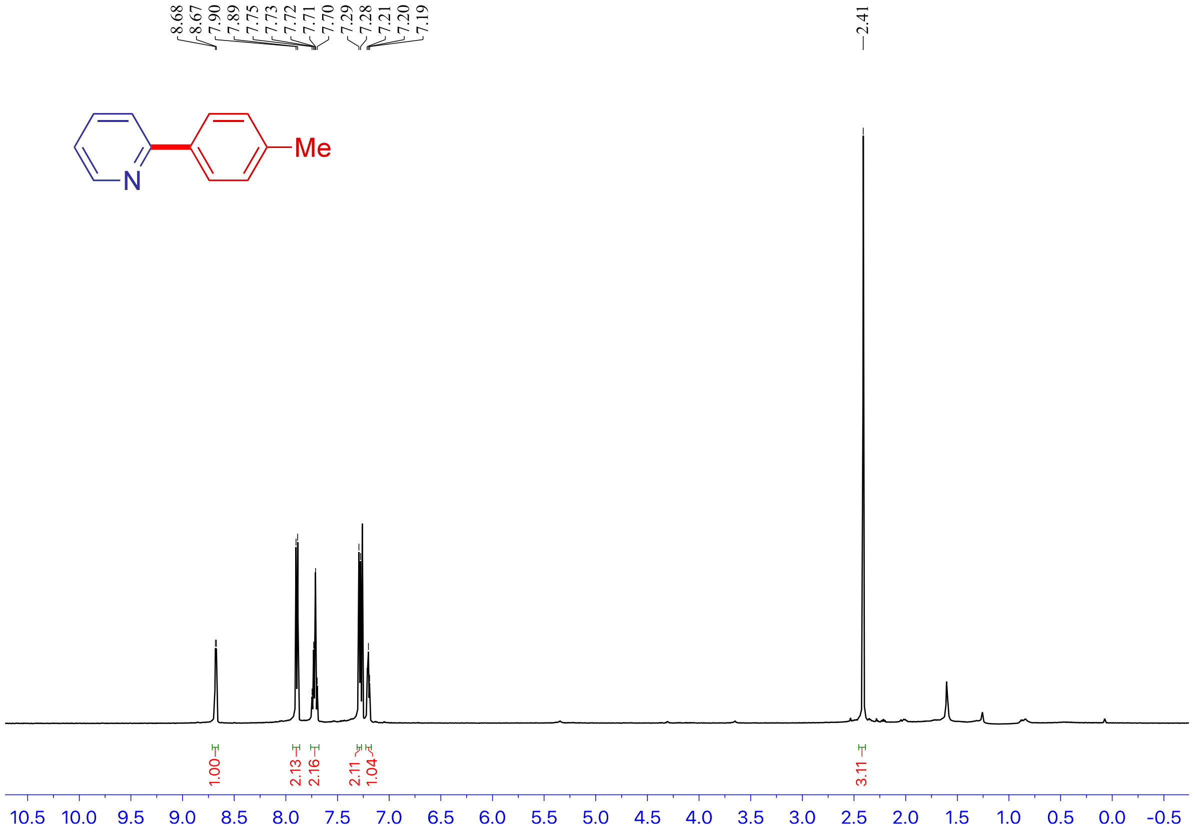

Figure S31. ^{13}C NMR spectrum of 2-(*p*-tolyl)pyridine, related to **Scheme 2**

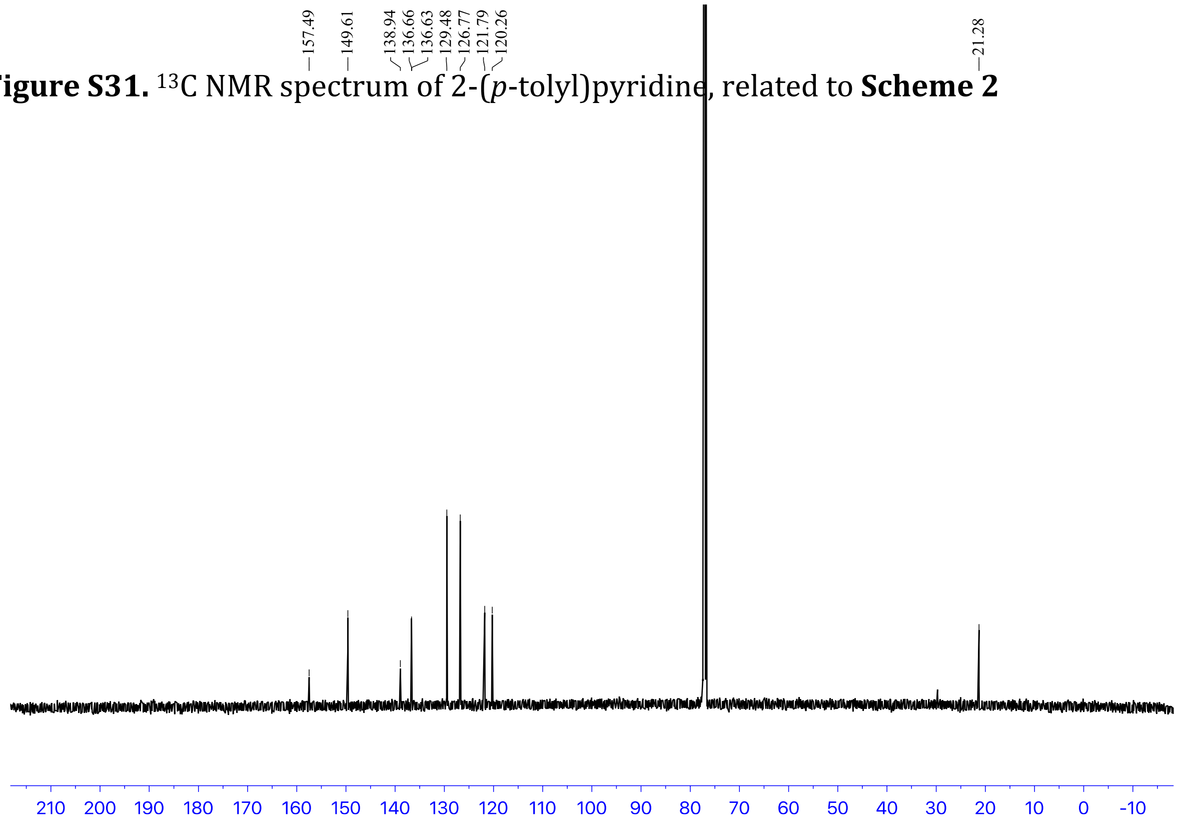

Figure S32. ^{1}H NMR spectrum of 3-(6-methoxynaphthalen-2-yl)pyridine, related to **Scheme 2**

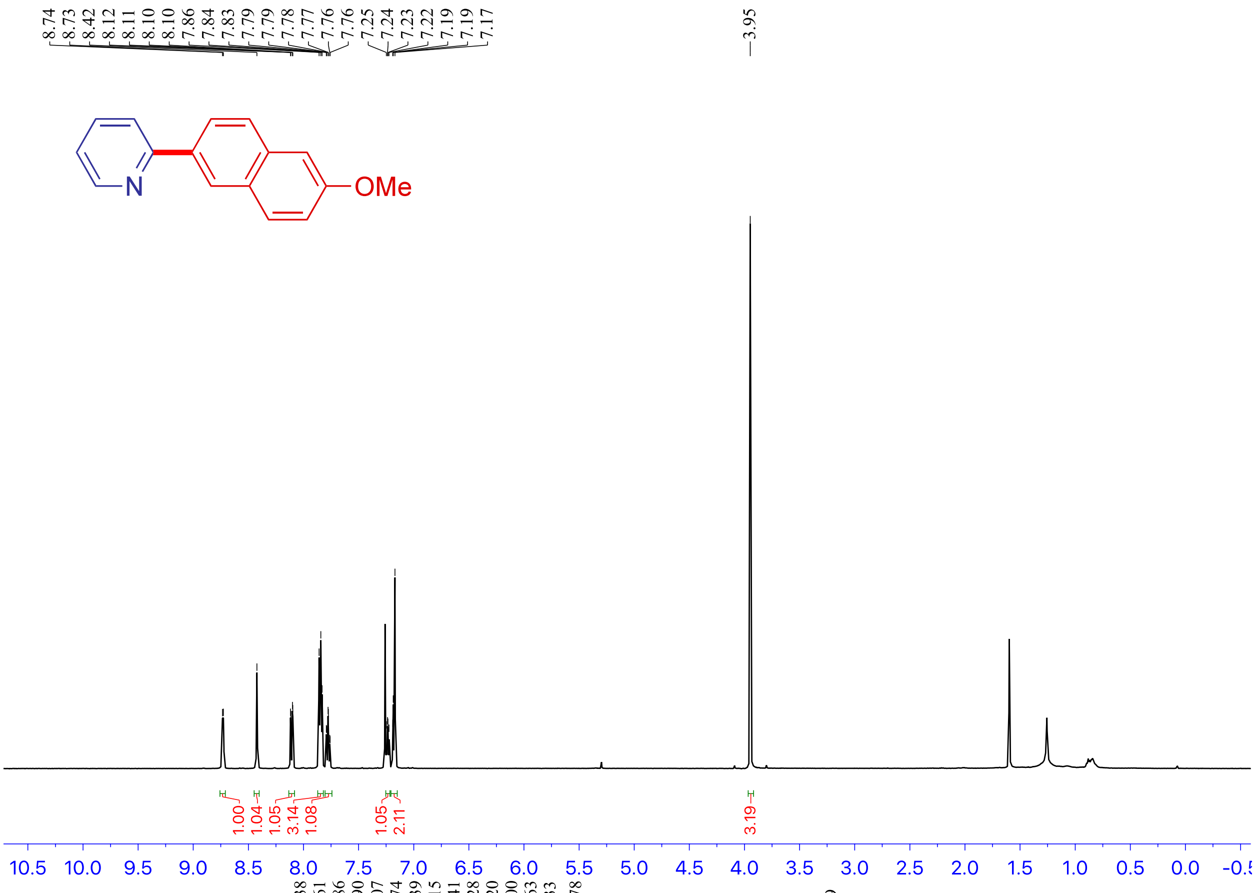

Figure S33. ^{13}C NMR spectrum of 3-(6-methoxynaphthalen-2-yl)pyridine, related to **Scheme 2**

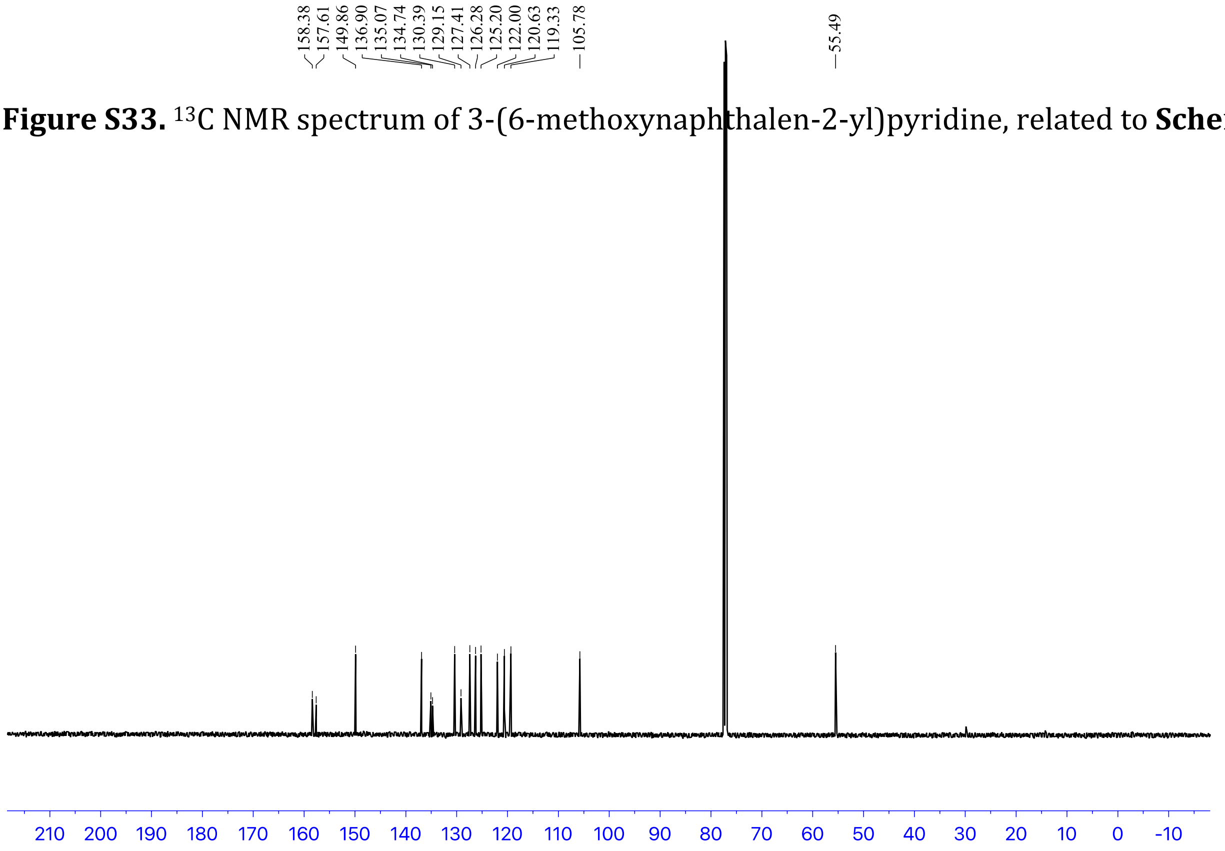

Figure S34. ^{1}H NMR spectrum of methyl 2-(*p*-tolyl)nicotinate, related to **Scheme 2**

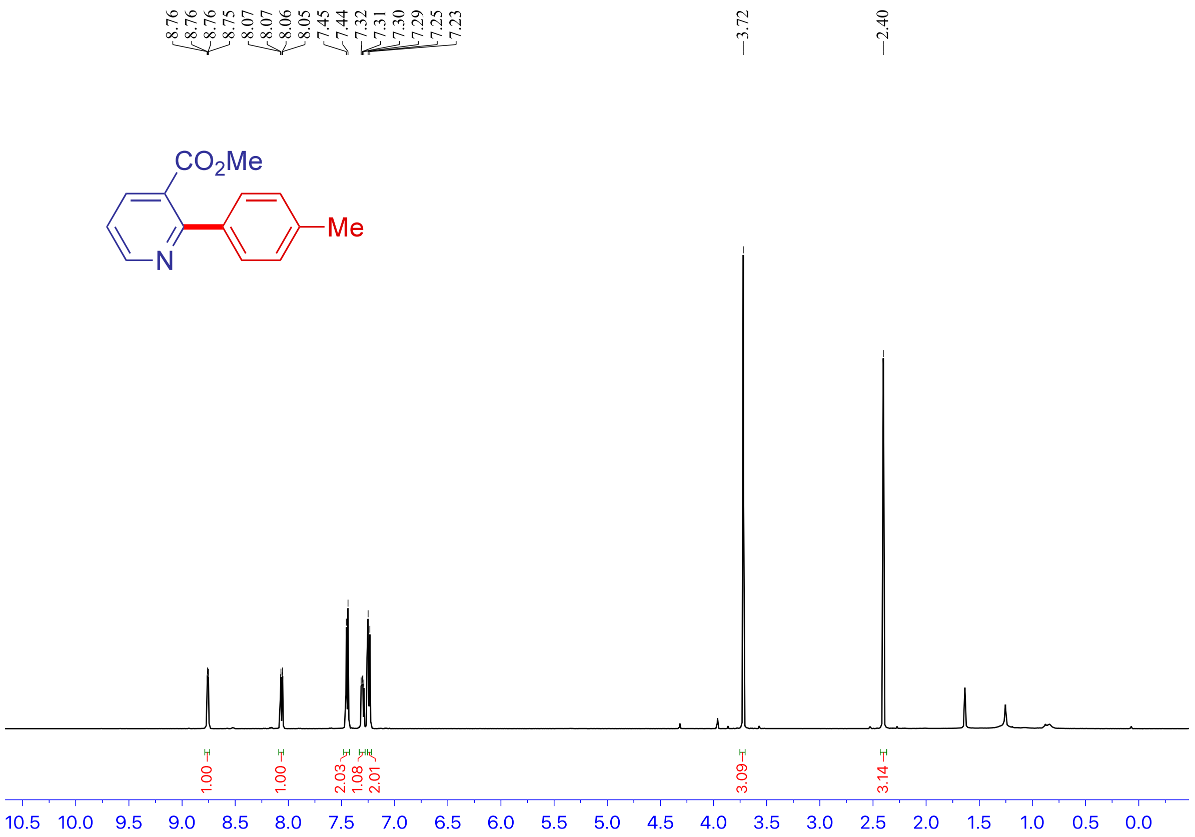

Figure S35. ^{13}C NMR spectrum of methyl 2-(*p*-tolyl)nicotinate, related to **Scheme 2**

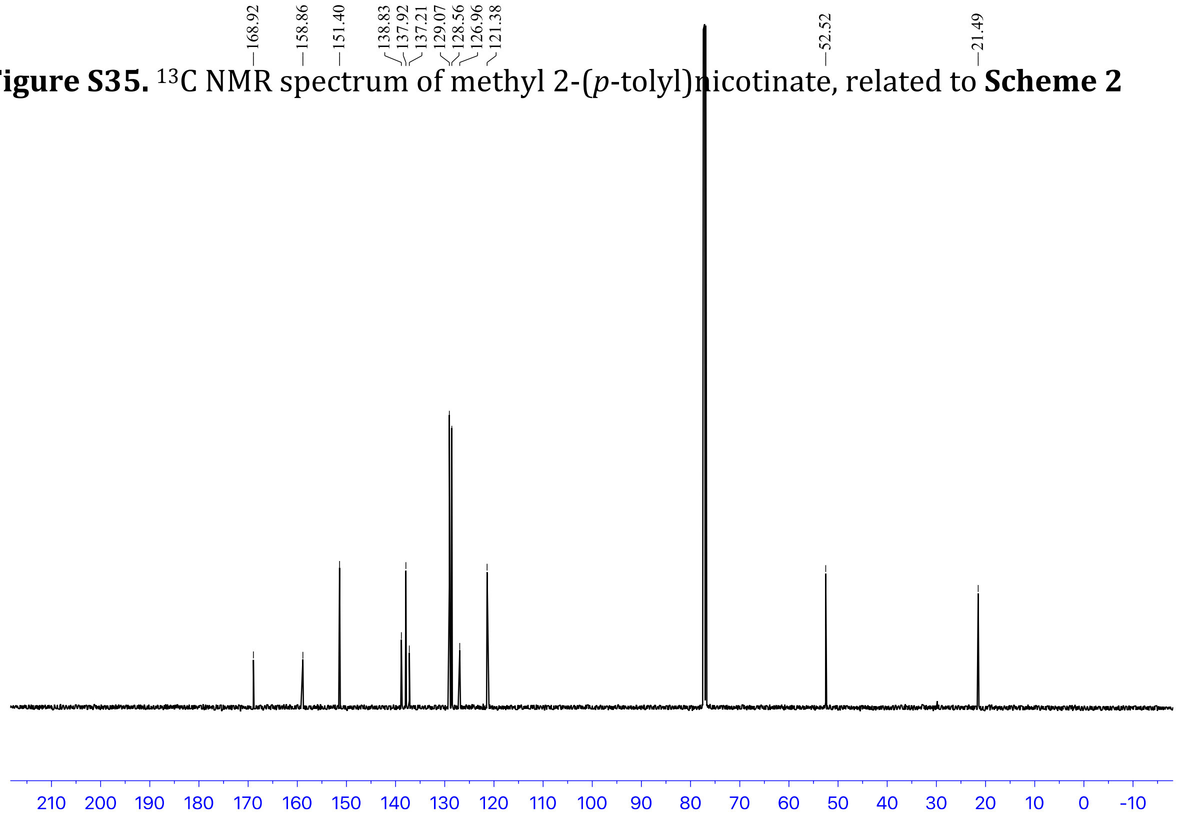

Figure S36. ^{1}H NMR spectrum of 3-(*p*-tolyl)pyridine, related to **Scheme 2**

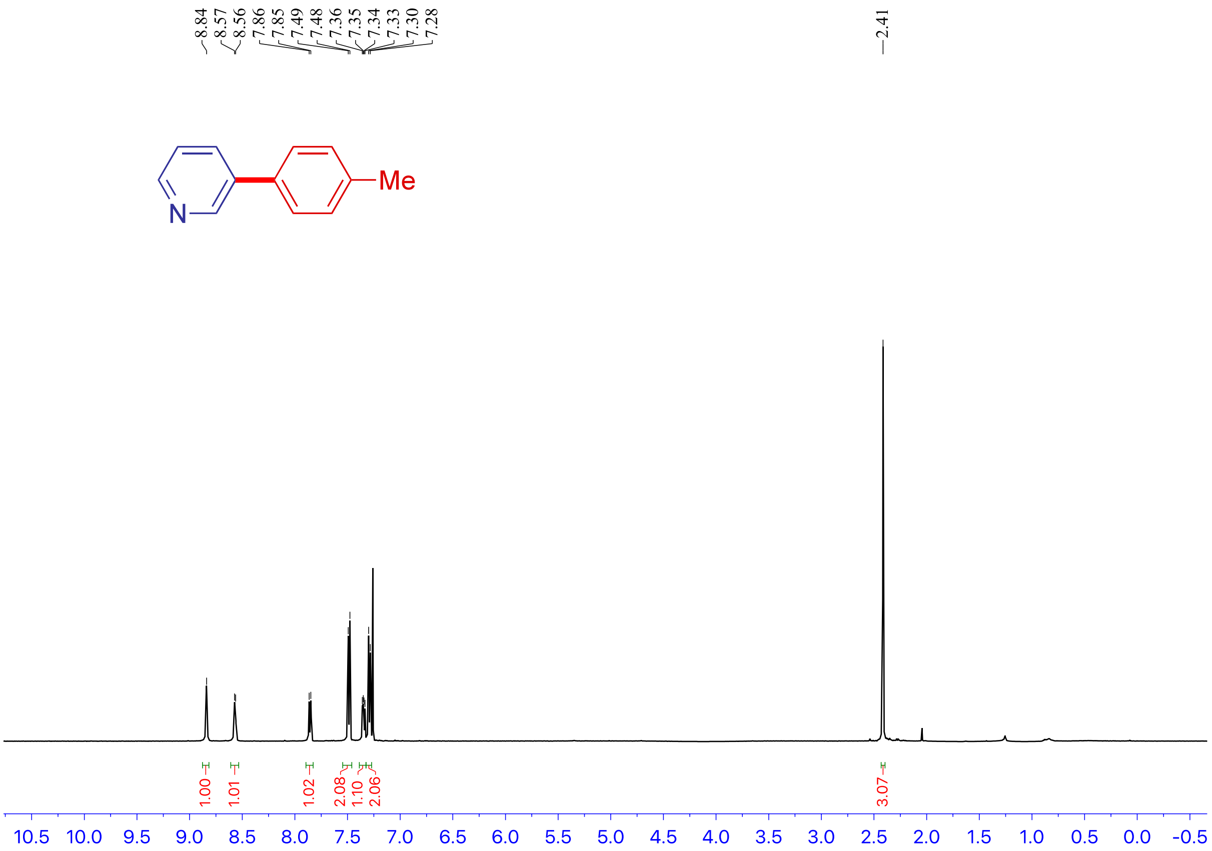

Figure S37. ^{13}C NMR spectrum of 3-(*p*-tolyl)pyridine, related to **Scheme 2**

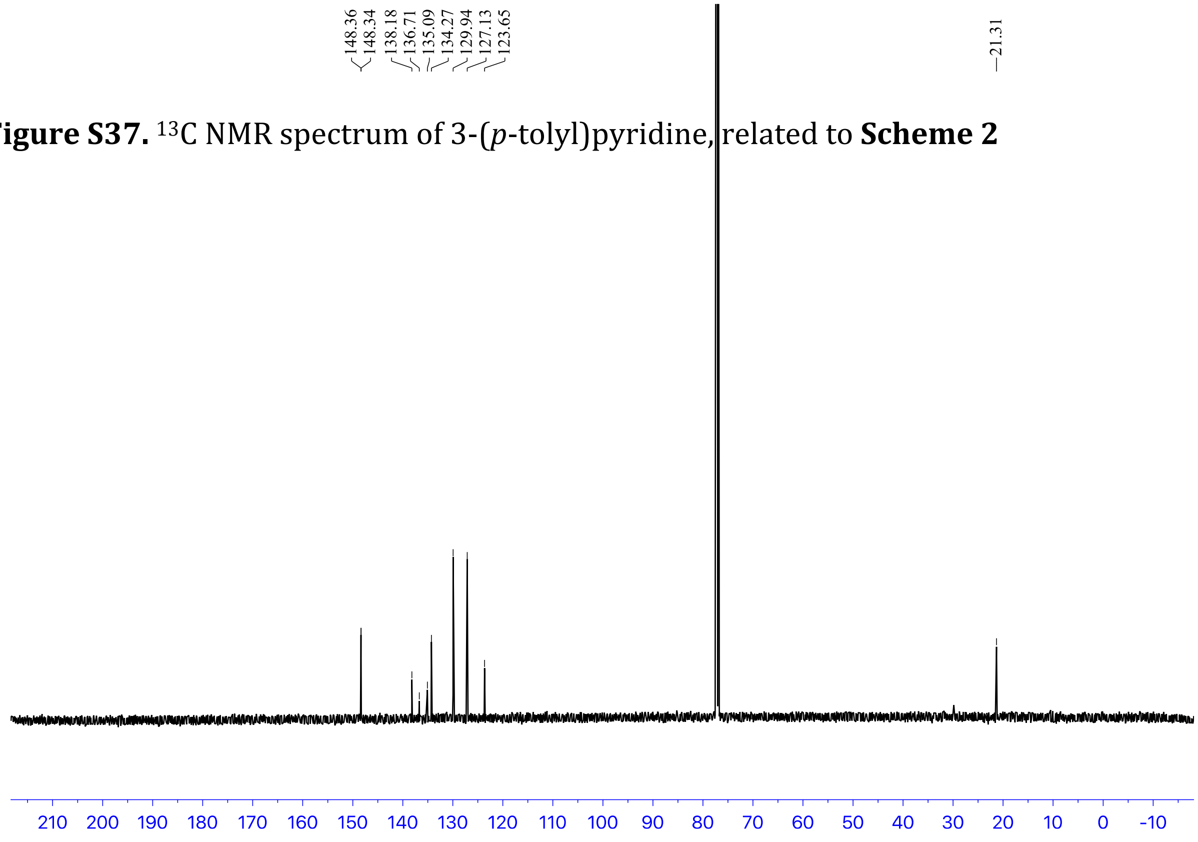

Figure S38. ^{1}H NMR spectrum of 3-(6-methoxynaphthalen-2-yl)pyridine, related to **Scheme 2**

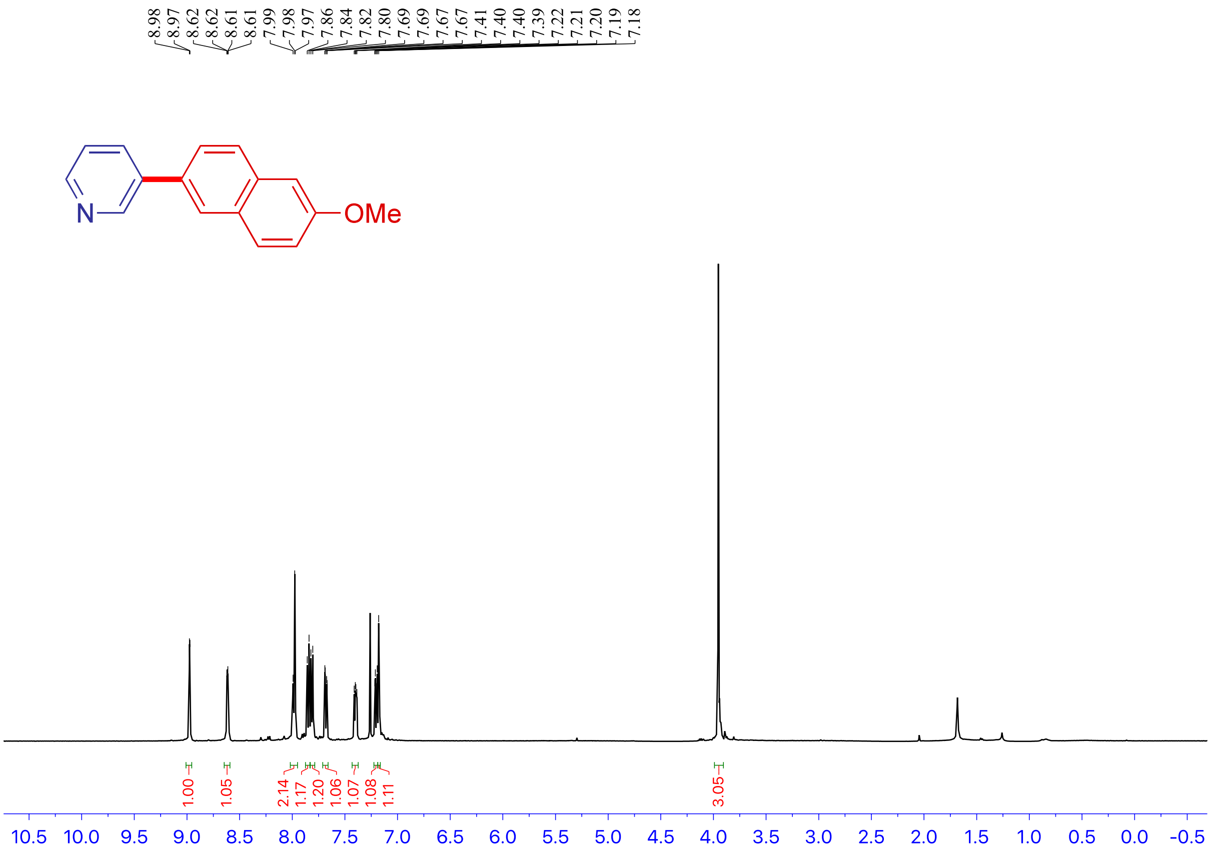

Figure S39. ^{13}C NMR spectrum of 3-(6-methoxynaphthalen-2-yl)pyridine, related to **Scheme 2**

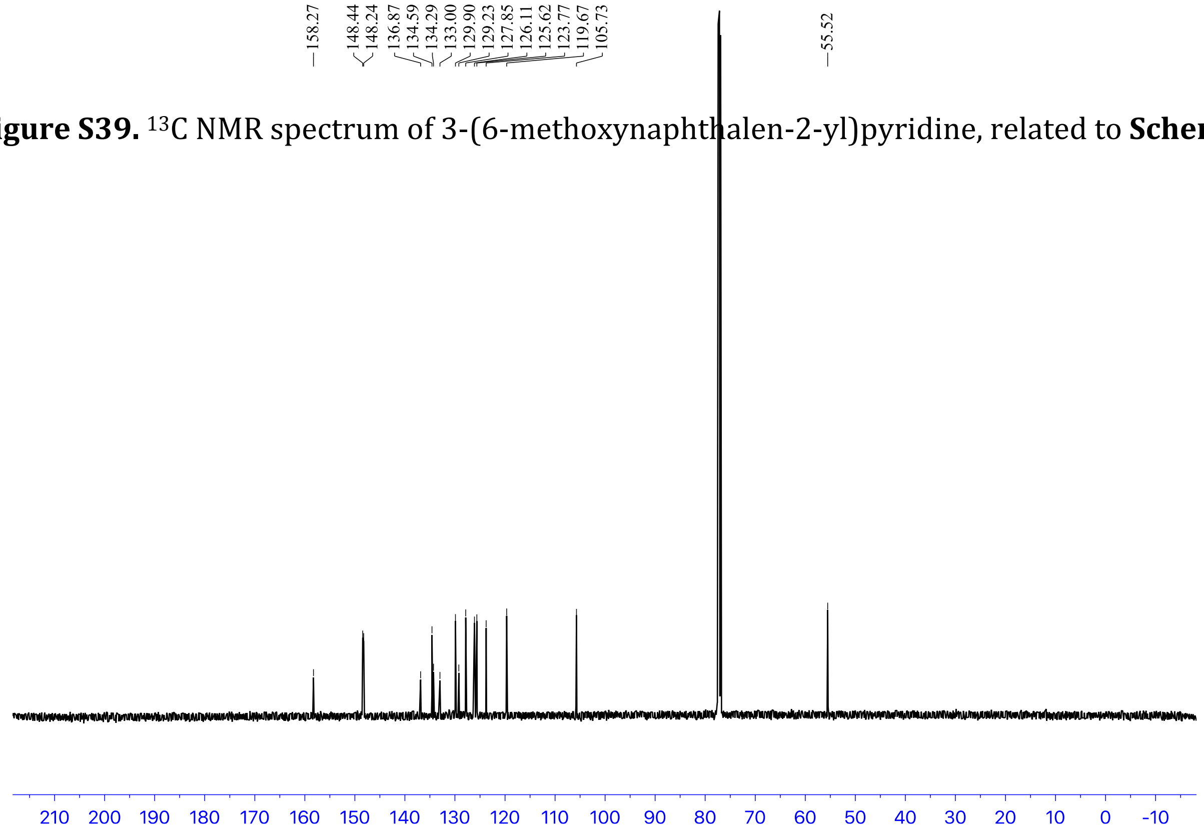

Figure S40. ^{1}H NMR spectrum of 5-(3,4-dimethoxyphenyl)-1*H*-indole, related to **Scheme 2**

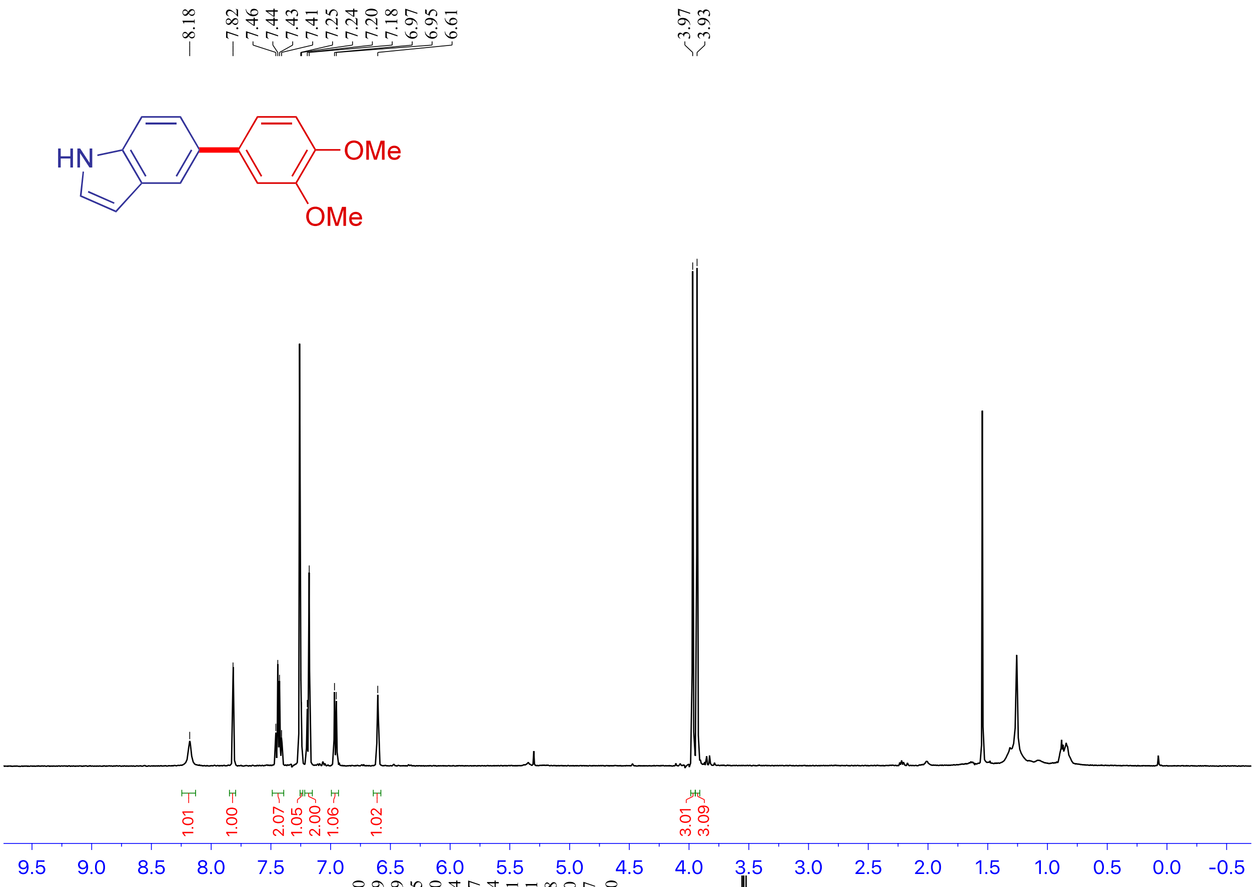

Figure S41. ^{13}C NMR spectrum of 5-(3,4-dimethoxyphenyl)-1*H*-indole, related to **Scheme 2**

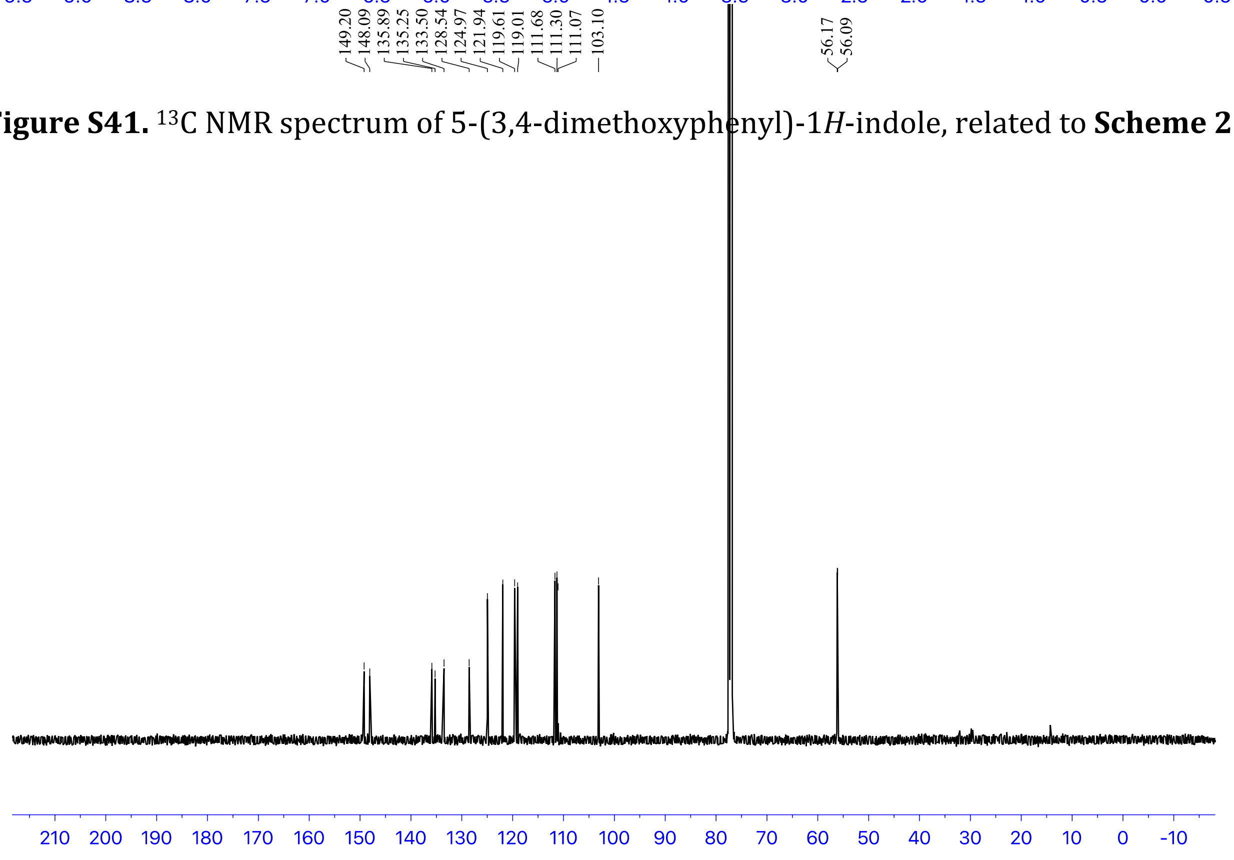

Figure S42. ^{1}H NMR spectrum of 2-(4-fluorophenyl)quinoline, related to **Scheme 2**

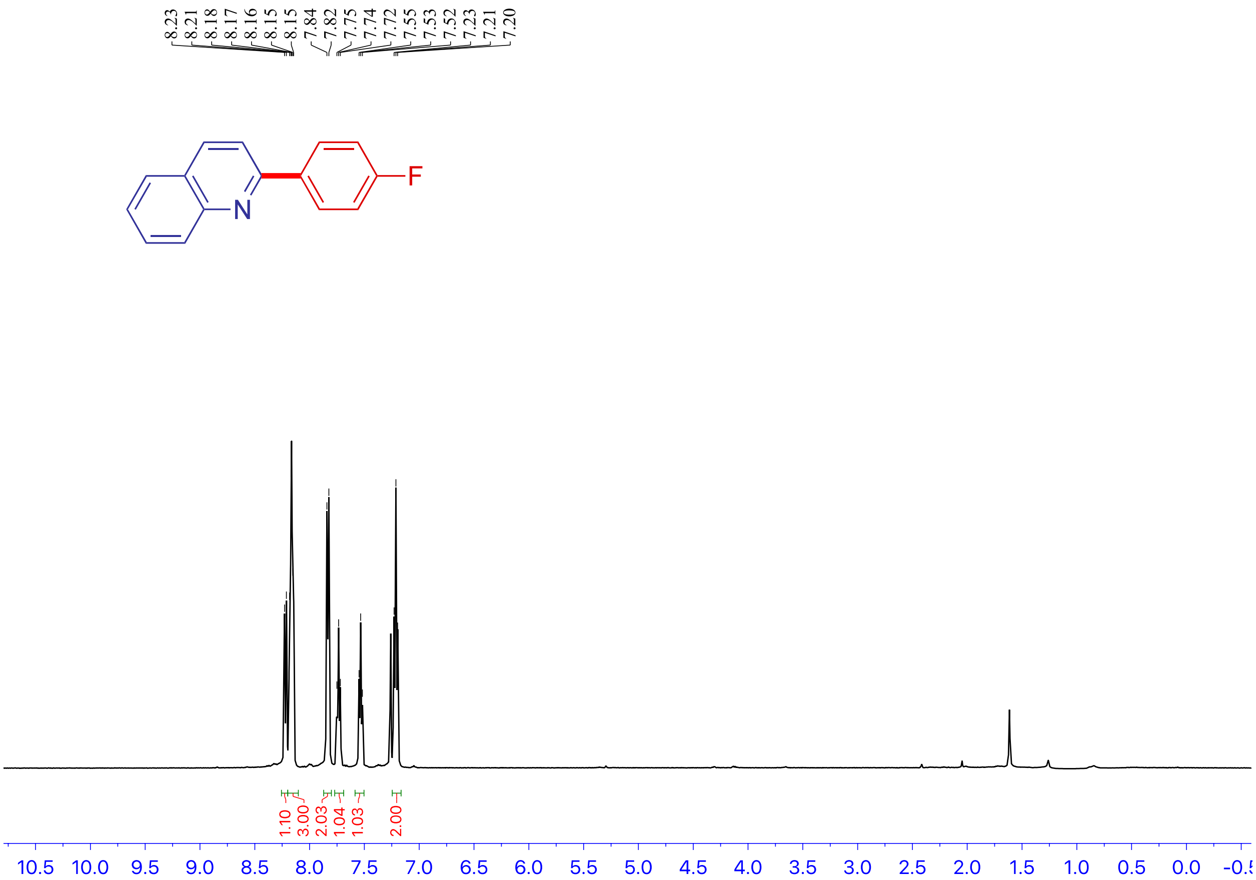

Figure S43. ^{13}C NMR spectrum of 2-(4-fluorophenyl)quinoline, related to **Scheme 2**

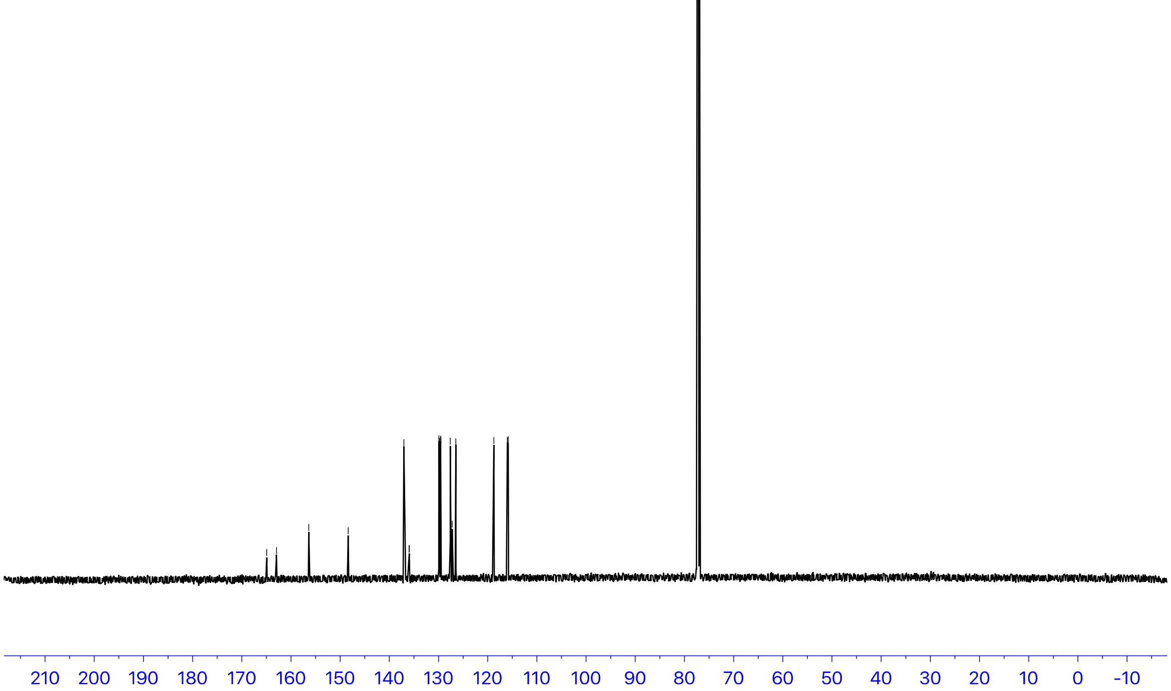

Figure S44. ^{19}F NMR spectrum of 2-(4-fluorophenyl)quinoline, related to **Scheme 2**

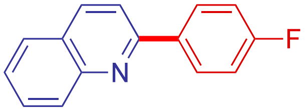

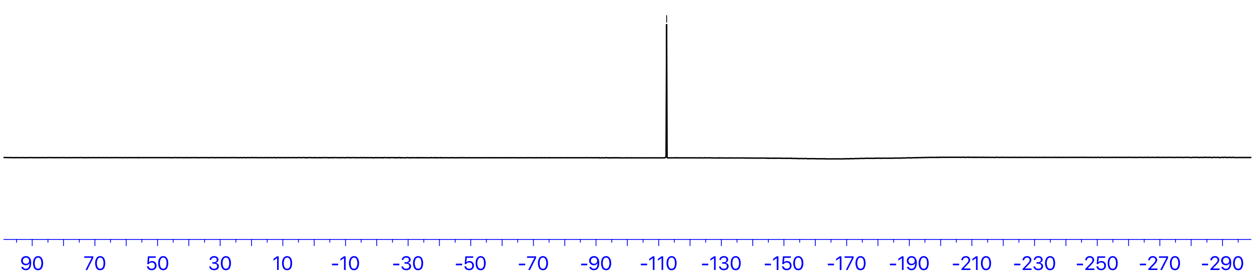

Figure S45. ^{1}H NMR spectrum of 2,4-dimethoxy-6-(*p*-tolyl)-1,3,5-triazine, related to **Scheme 2**

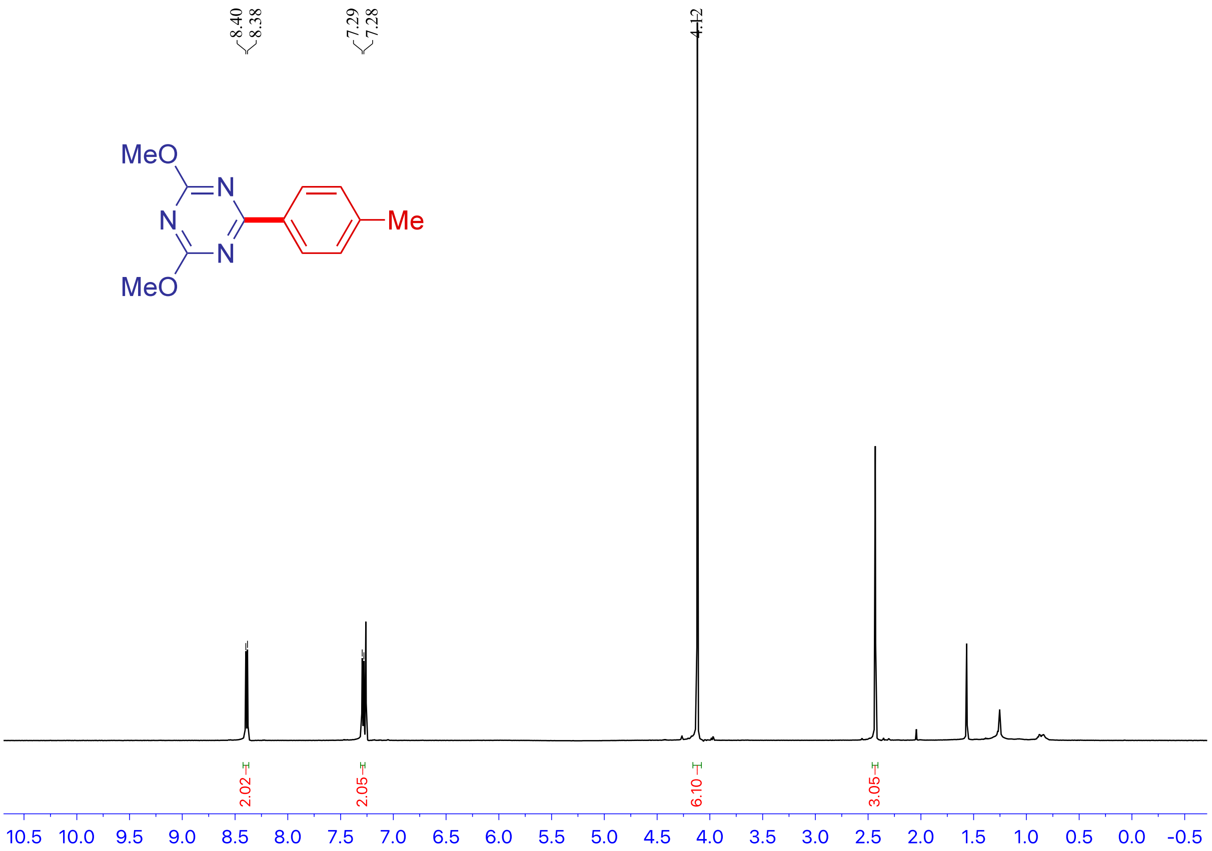

Figure S46. ^{13}C NMR spectrum of 2,4-dimethoxy-6-(*p*-tolyl)-1,3,5-triazine, related to **Scheme 2**

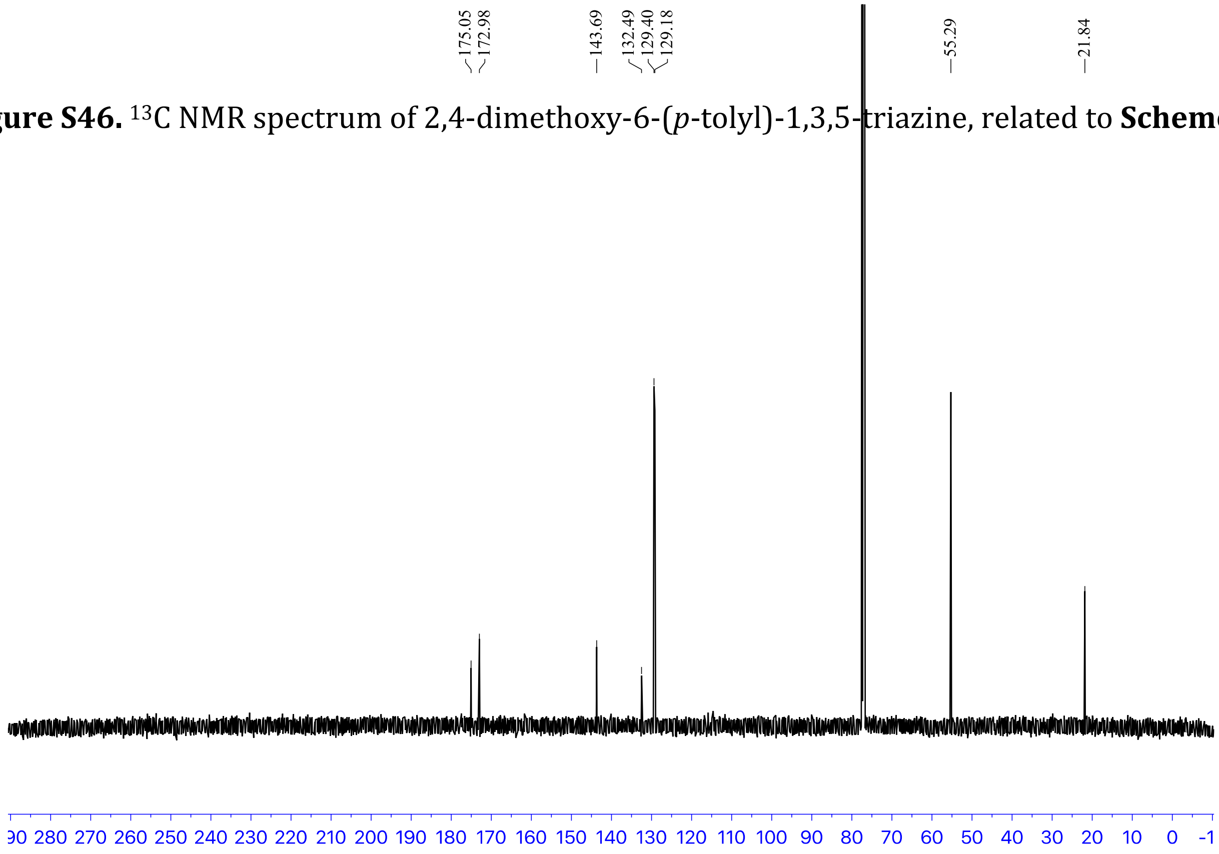

Figure S47. ^{1}H NMR spectrum of 4-(4-(*tert*-butyl)phenyl)-7-nitrobenzo[*c*][1,2,5]oxadiazole, related to **Scheme 2**

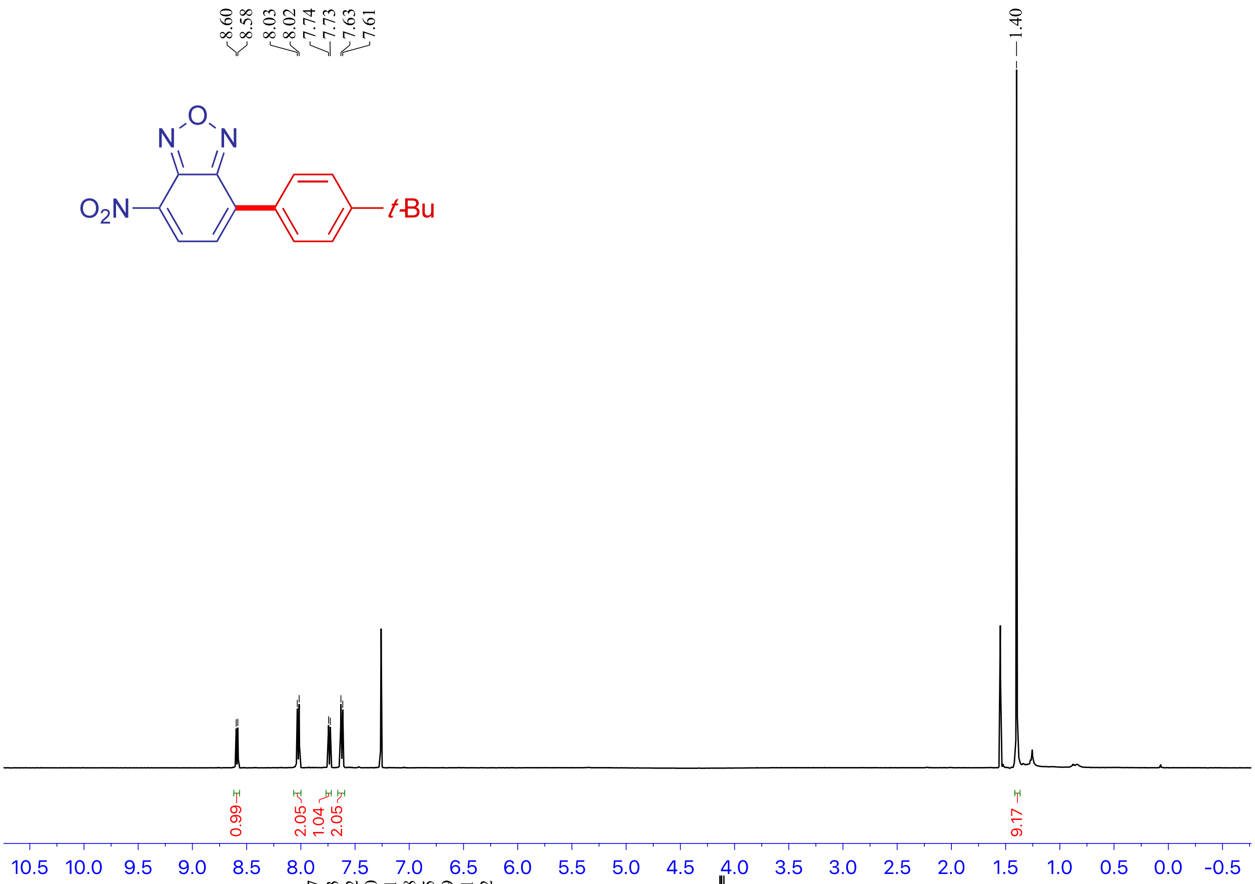

Figure S48. ^{13}C NMR spectrum of 4-(4-(*tert*-butyl)phenyl)-7-nitrobenzo[*c*][1,2,5]oxadiazole, related to **Scheme 2**

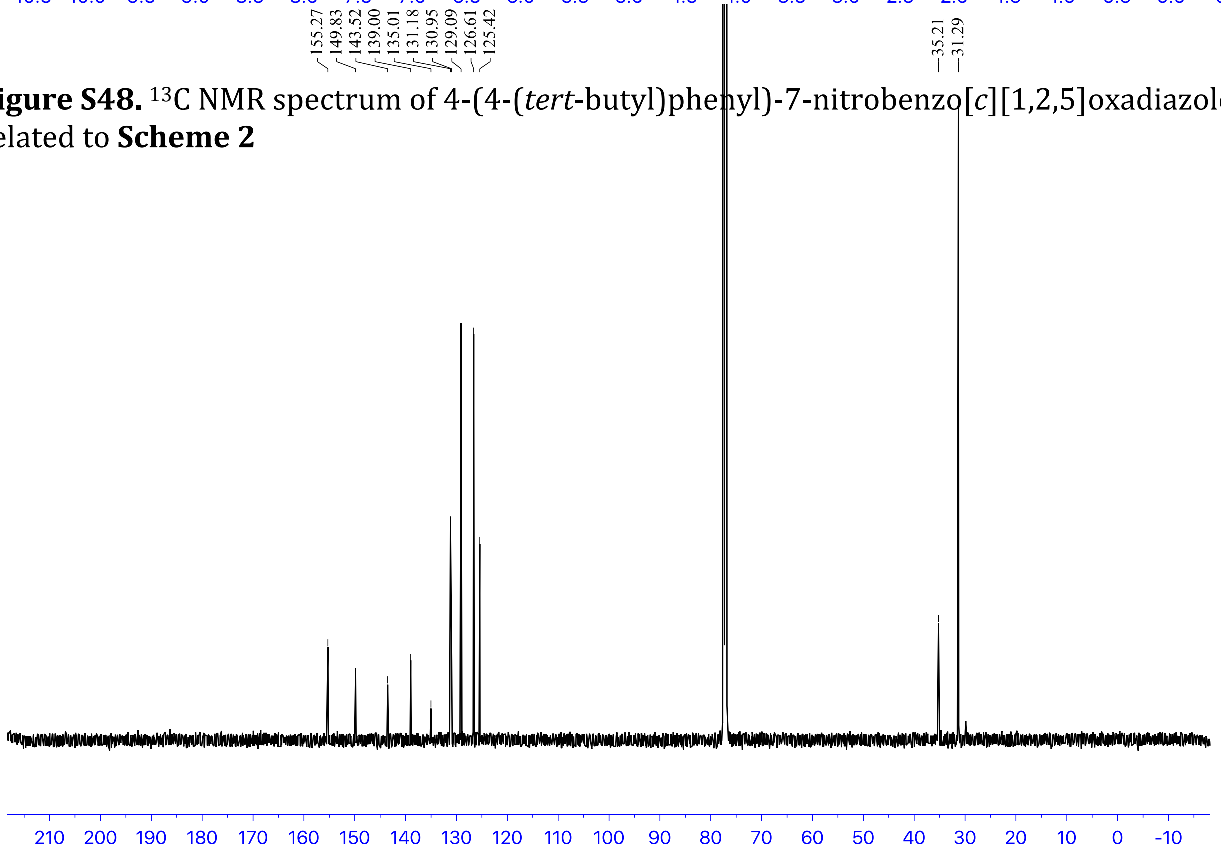

Figure S49. ${}^{1}H$ NMR spectrum of 3',4'-dimethoxy-4-formylbiphenyl, related to **Scheme 2**

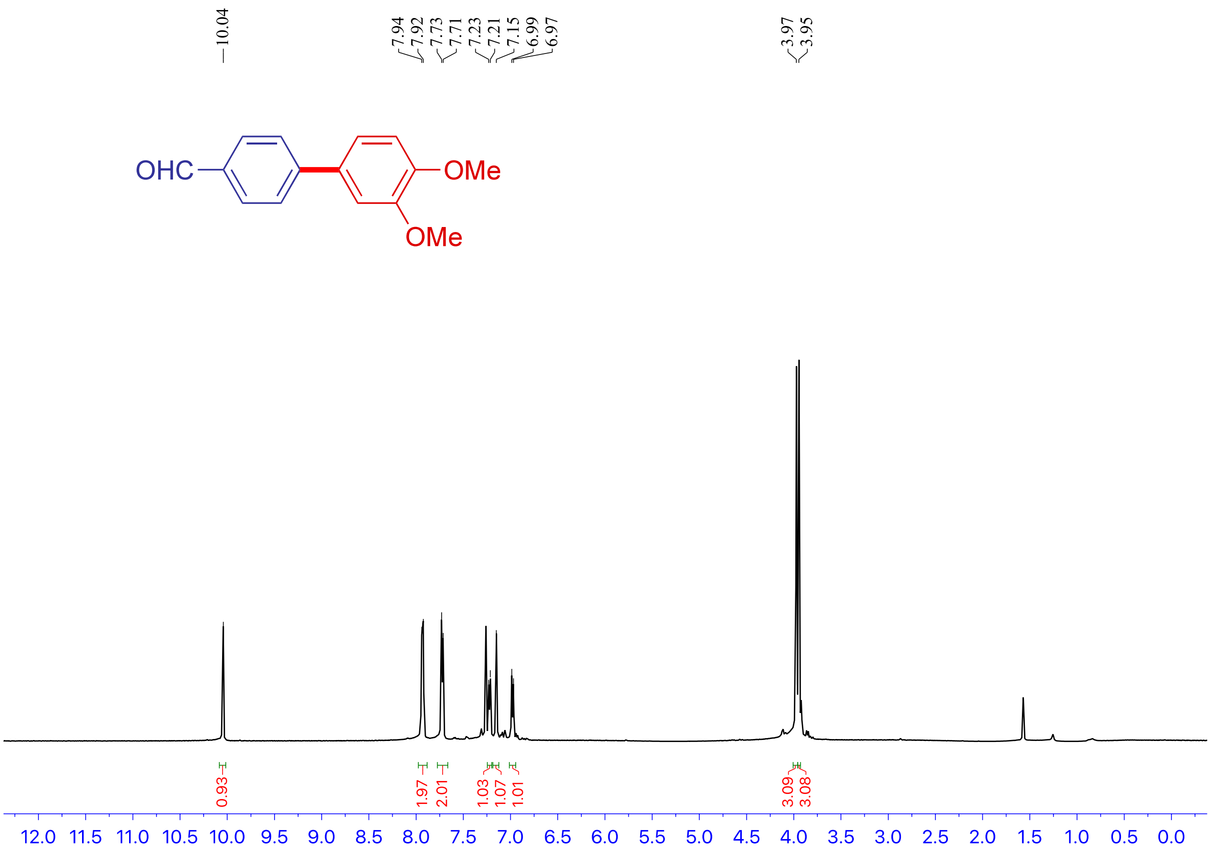

Figure S50. ${}^{13}C$ NMR spectrum of 3',4'-dimethoxy-4-formylbiphenyl, related to **Scheme 2**

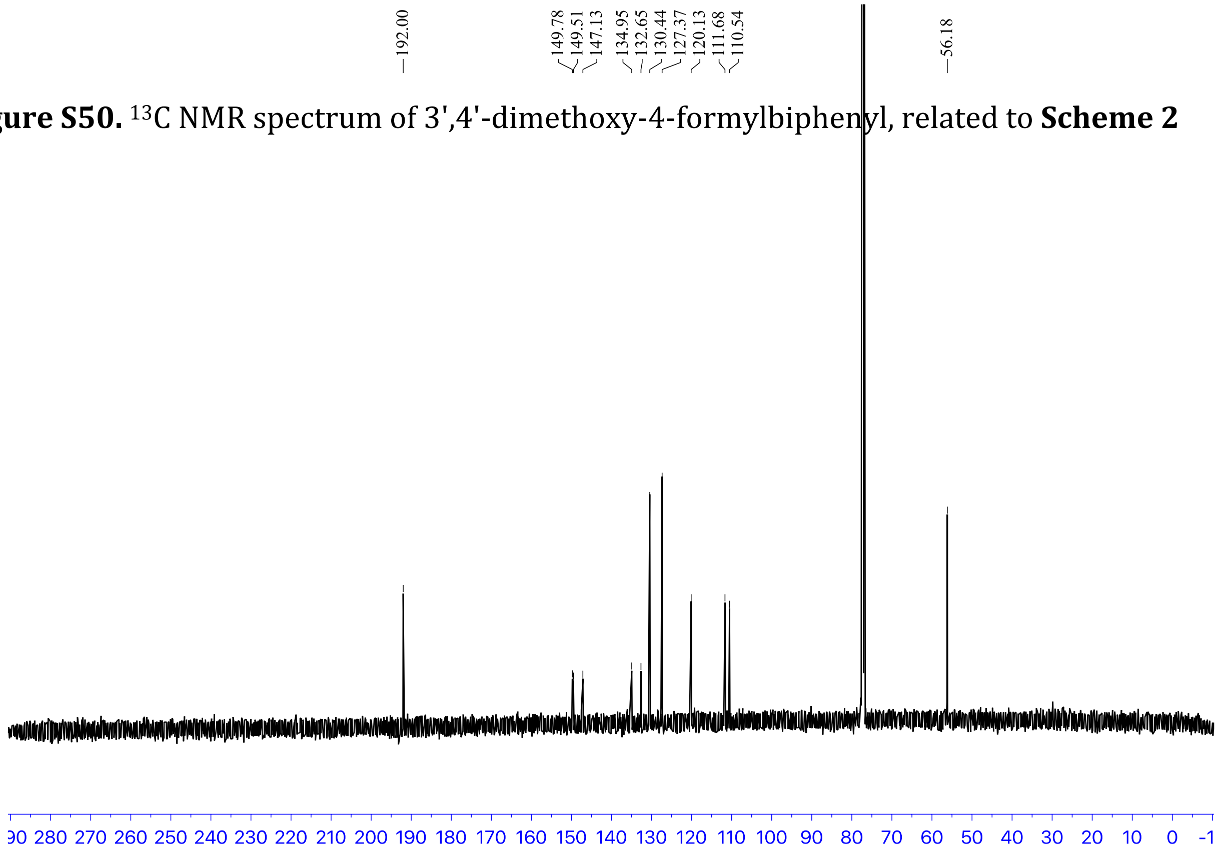

Figure S51. ^{1}H NMR spectrum of 4'-methyl-[1,1'-biphenyl]-4-carboxylic acid, related to **Scheme 2**

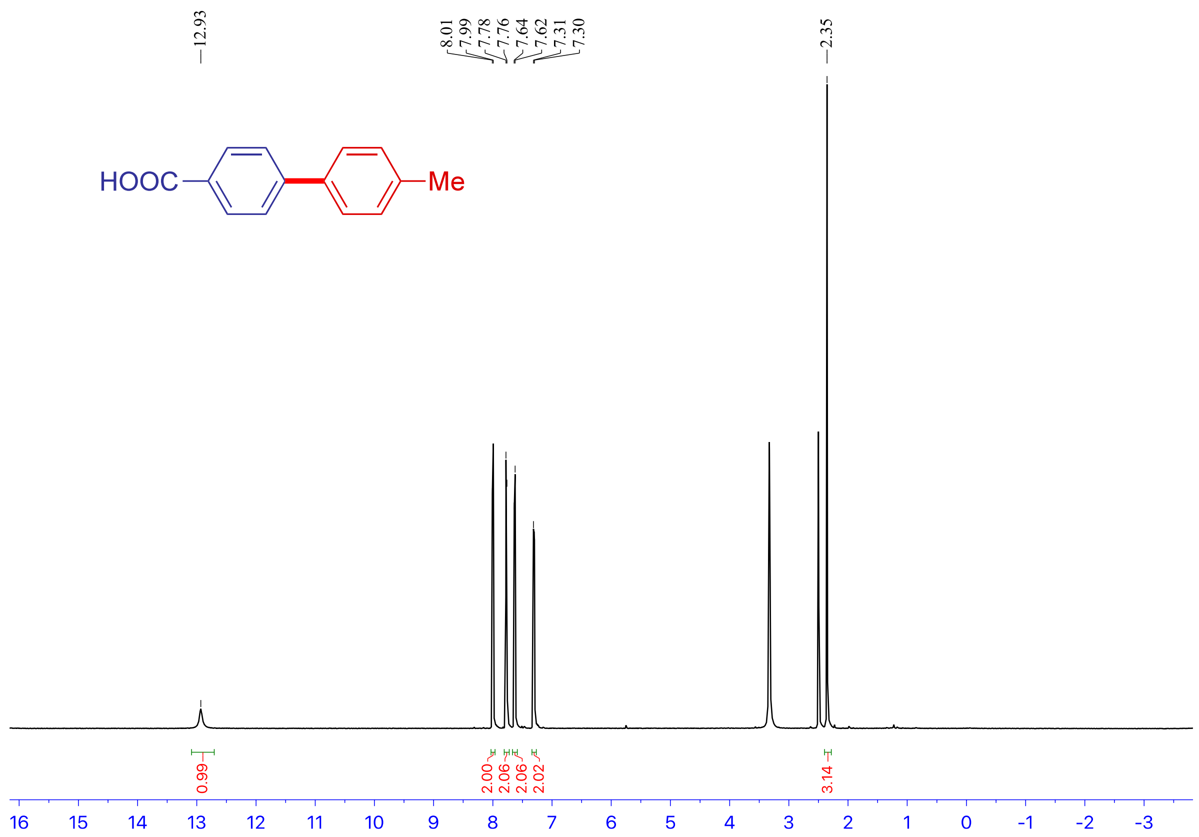

Figure S52. ^{13}C NMR spectrum of 4'-methyl-[1,1'-biphenyl]-4-carboxylic acid, related to **Scheme 2**

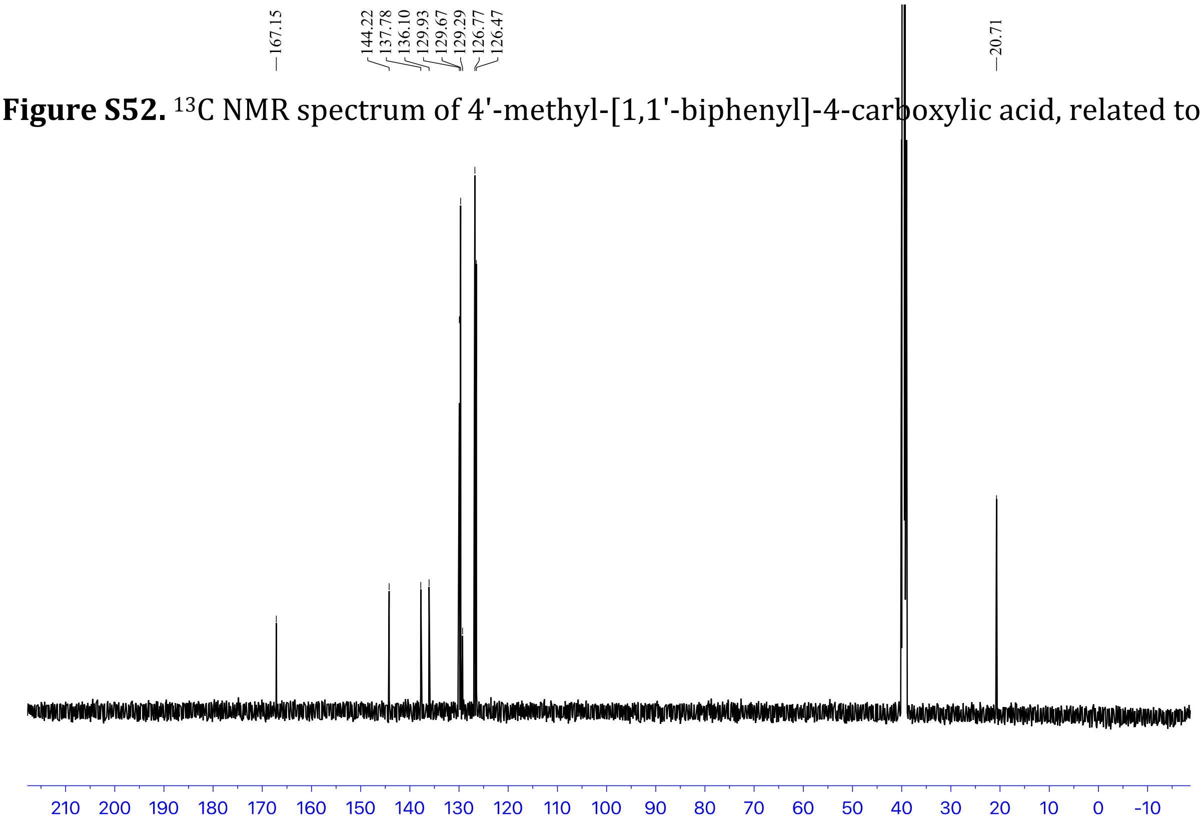

Figure S53. ^{1}H NMR spectrum of 5-(*p*-tolyl)benzo[*d*][1,3]dioxole, related to **Scheme 2**

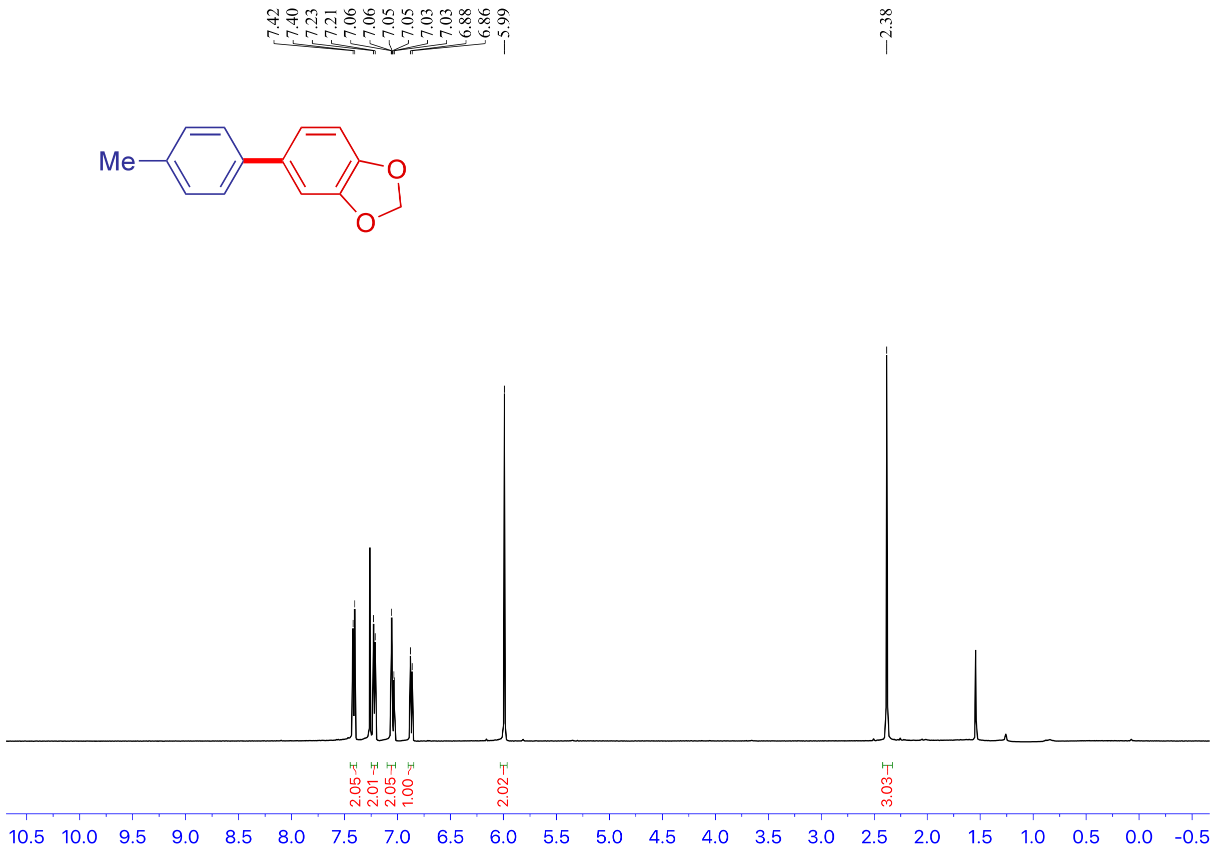

Figure S54. ^{13}C NMR spectrum of 5-(*p*-tolyl)benzo[*d*][1,3]dioxole, related to **Scheme 2**

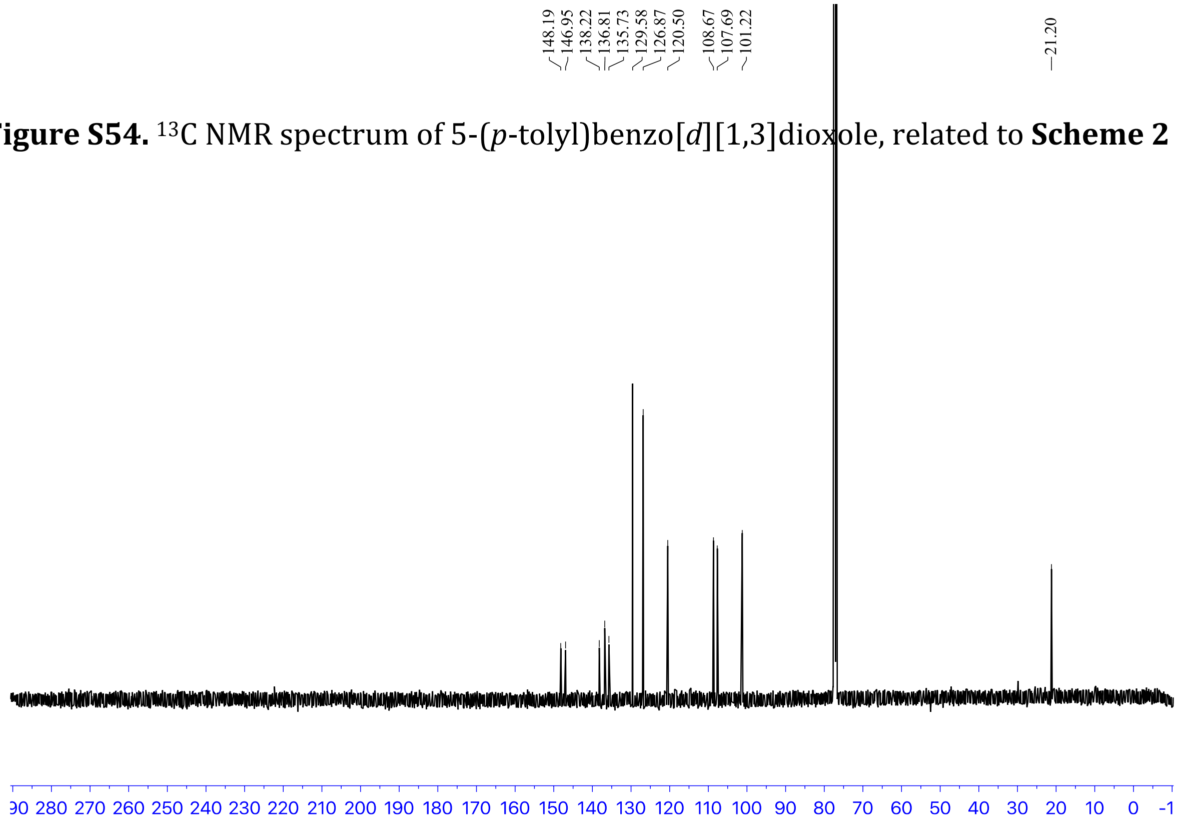

Figure S55. ^{1}H NMR spectrum of 2-(2,4-difluorophenyl)pyridine, related to **Scheme 2**

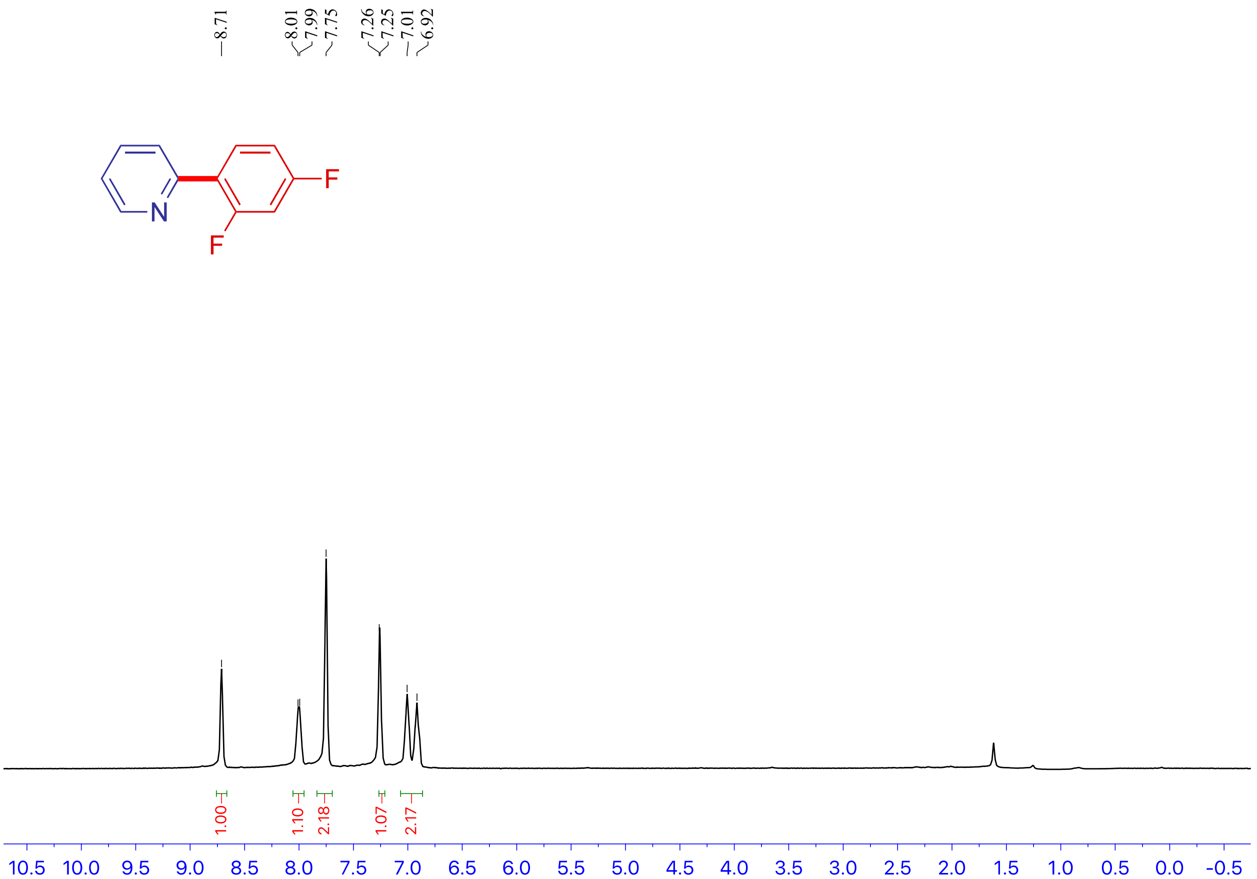

Figure S56. ^{13}C NMR spectrum of 2-(2,4-difluorophenyl)pyridine, related to **Scheme 2**

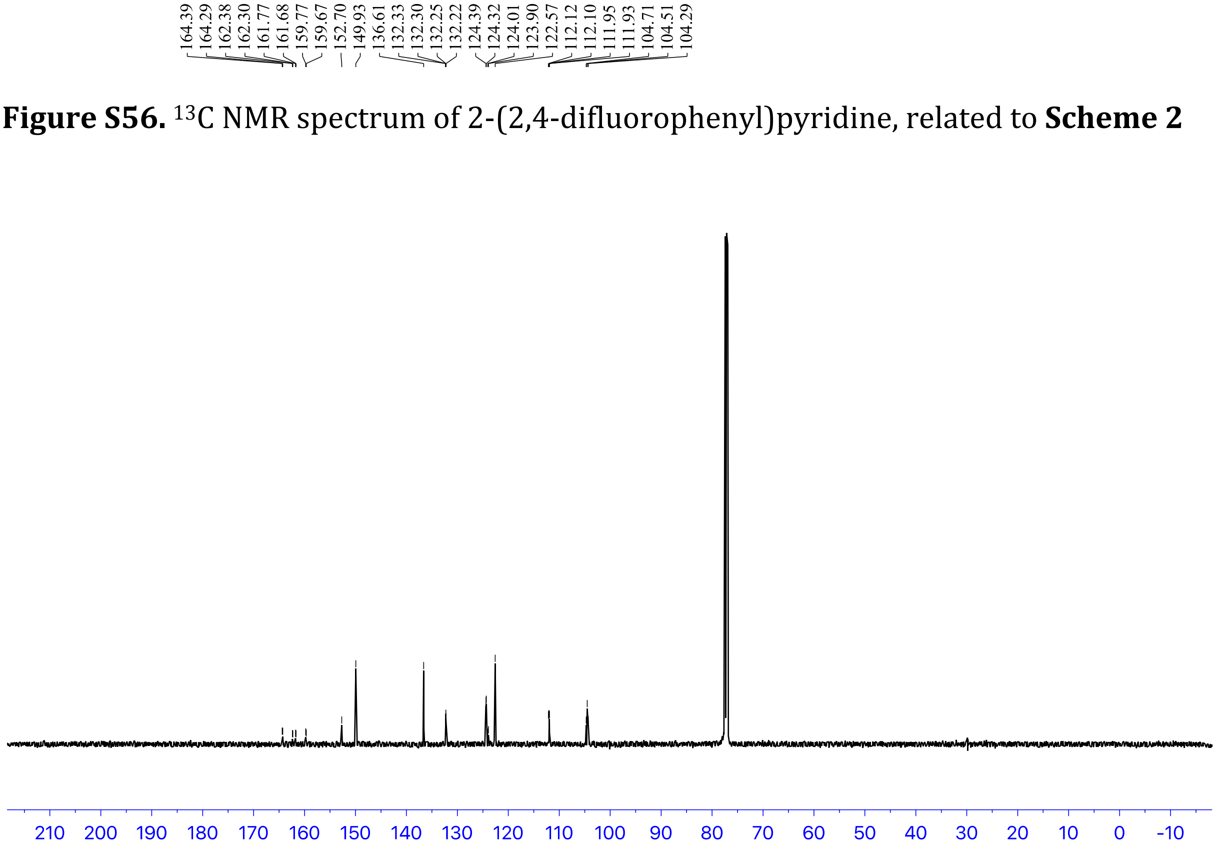

Figure S57. ^{19}F NMR spectrum of 2-(2,4-difluorophenyl)pyridine, related to **Scheme 2**

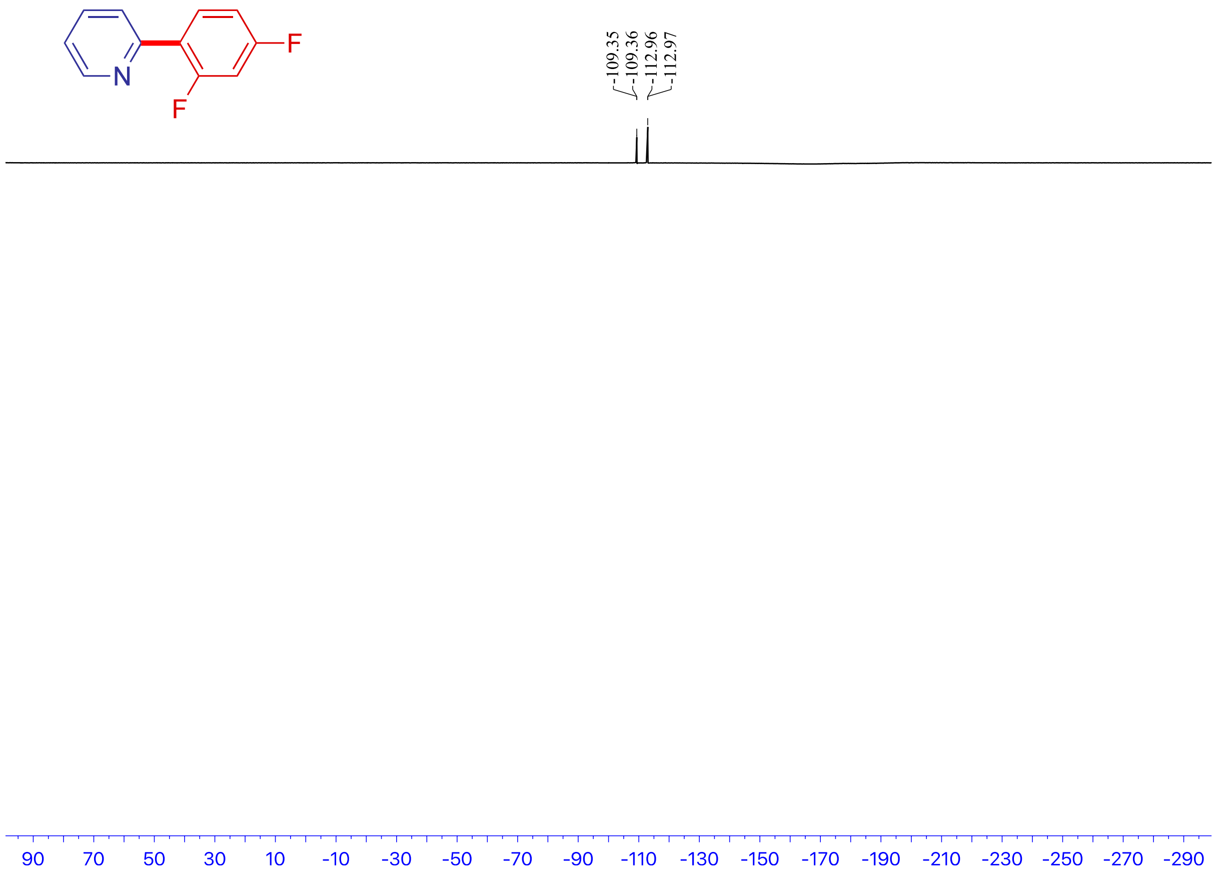

Figure S58. ^{1}H NMR spectrum of 2,4-dimethoxy-6-(thiophen-3-yl)-1,3,5-triazine, related to **Scheme 2**

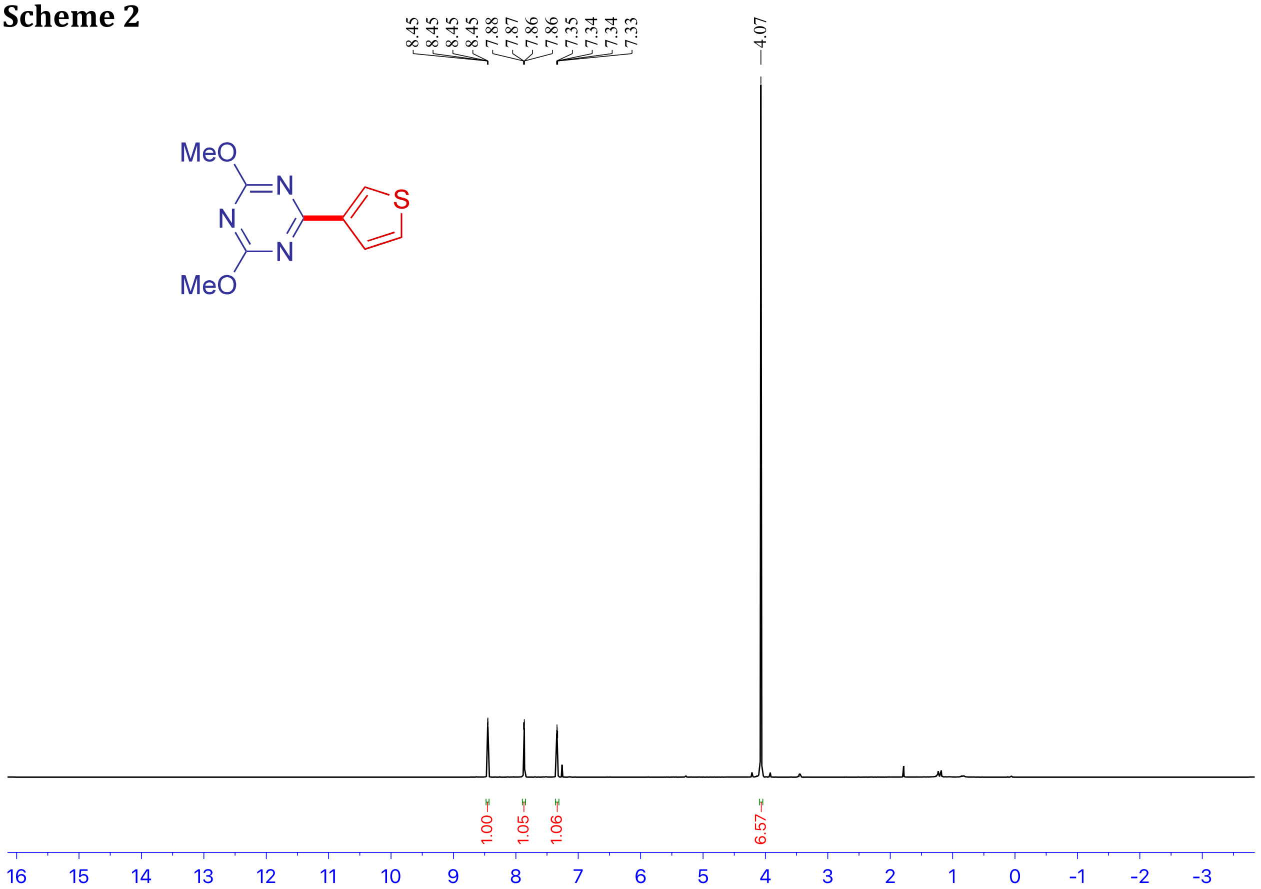

Figure S59. ^{13}C NMR spectrum of 2,4-dimethoxy-6-(thiophen-3-yl)-1,3,5-triazine, related to **Scheme 2**

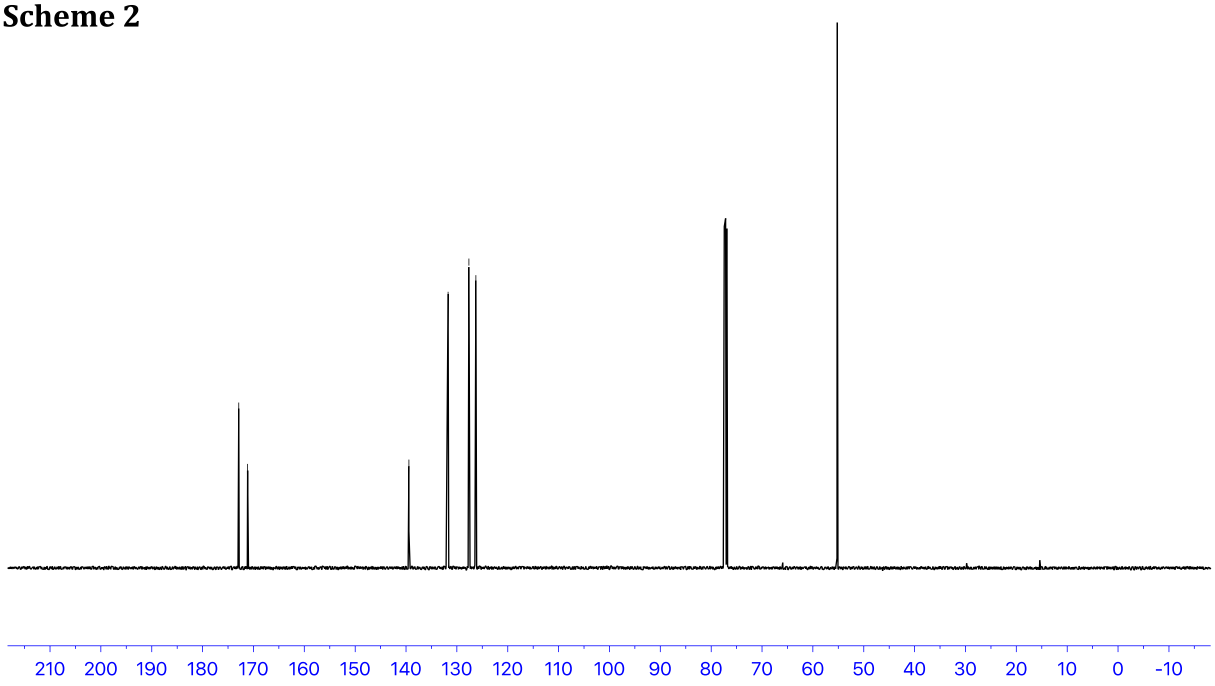

Figure S60. ^{1}H NMR spectrum of 3-(4,6-dimethoxy-1,3,5-triazin-2-yl)benzonitrile, related to **Scheme 2**

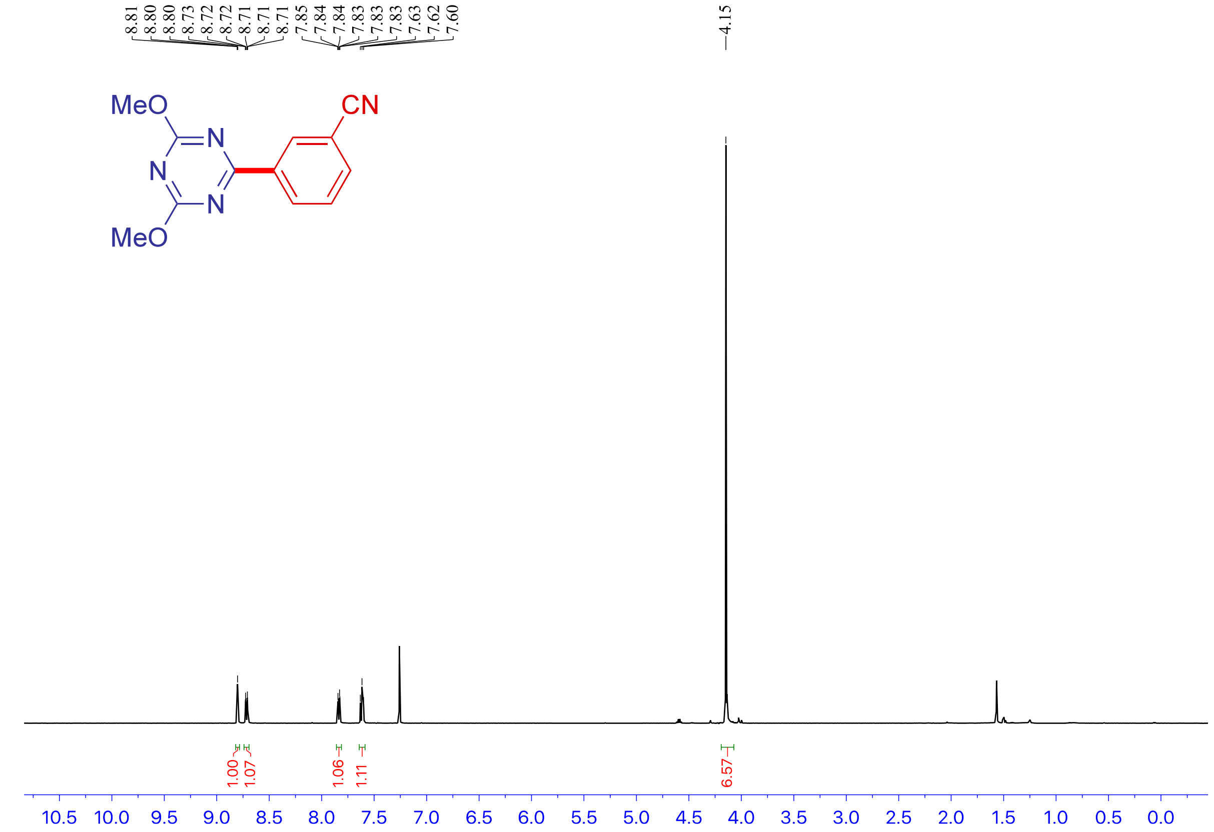

Figure S61. ^{13}C NMR spectrum of 3-(4,6-dimethoxy-1,3,5-triazin-2-yl)benzonitrile, related to **Scheme 2**

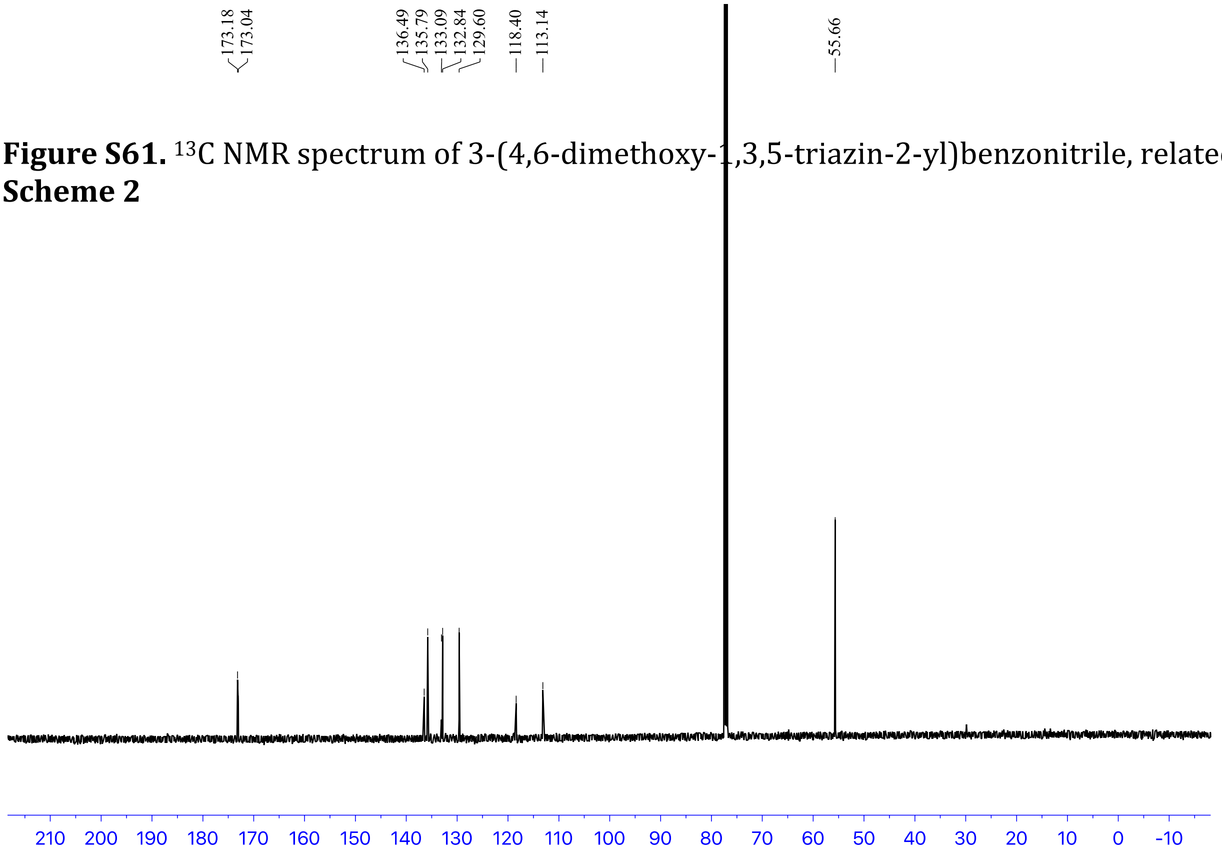

Figure S62. ^{1}H NMR spectrum of 2-methoxy-5-(*p*-tolyl)pyridine, related to **Scheme 3**

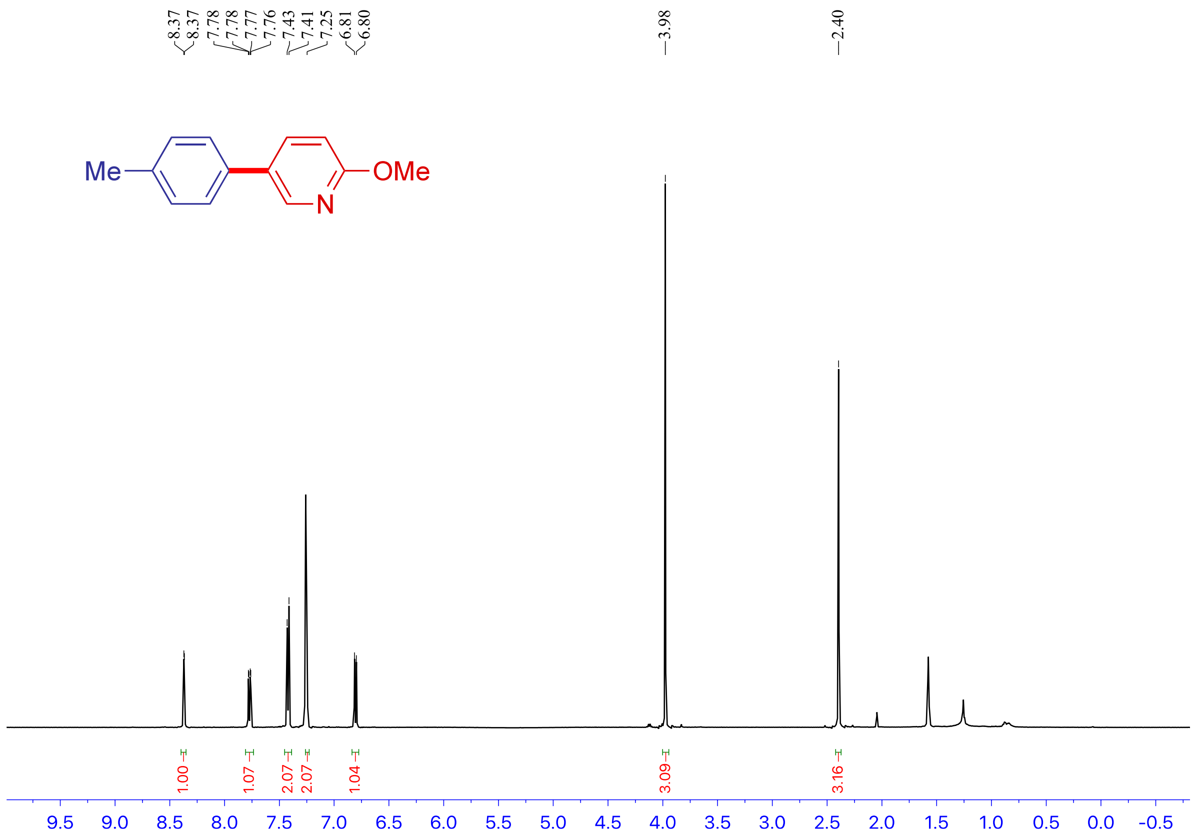

Figure S63. ^{13}C NMR spectrum of 2-methoxy-5-(*p*-tolyl)pyridine, related to **Scheme 3**

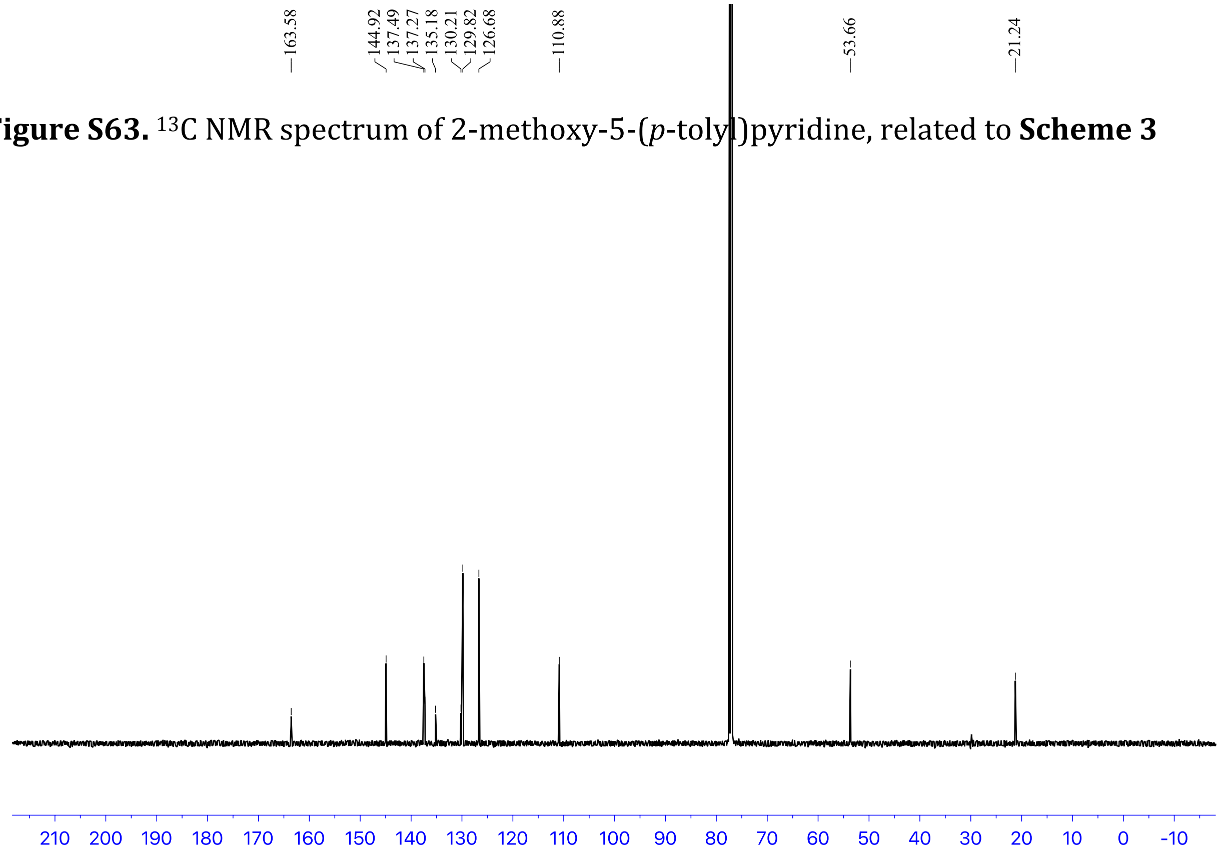

Figure S64. ^{1}H NMR spectrum of *tert*-butyl (4-(quinolin-2-yl)phenyl)carbamate, related to **Scheme 3**

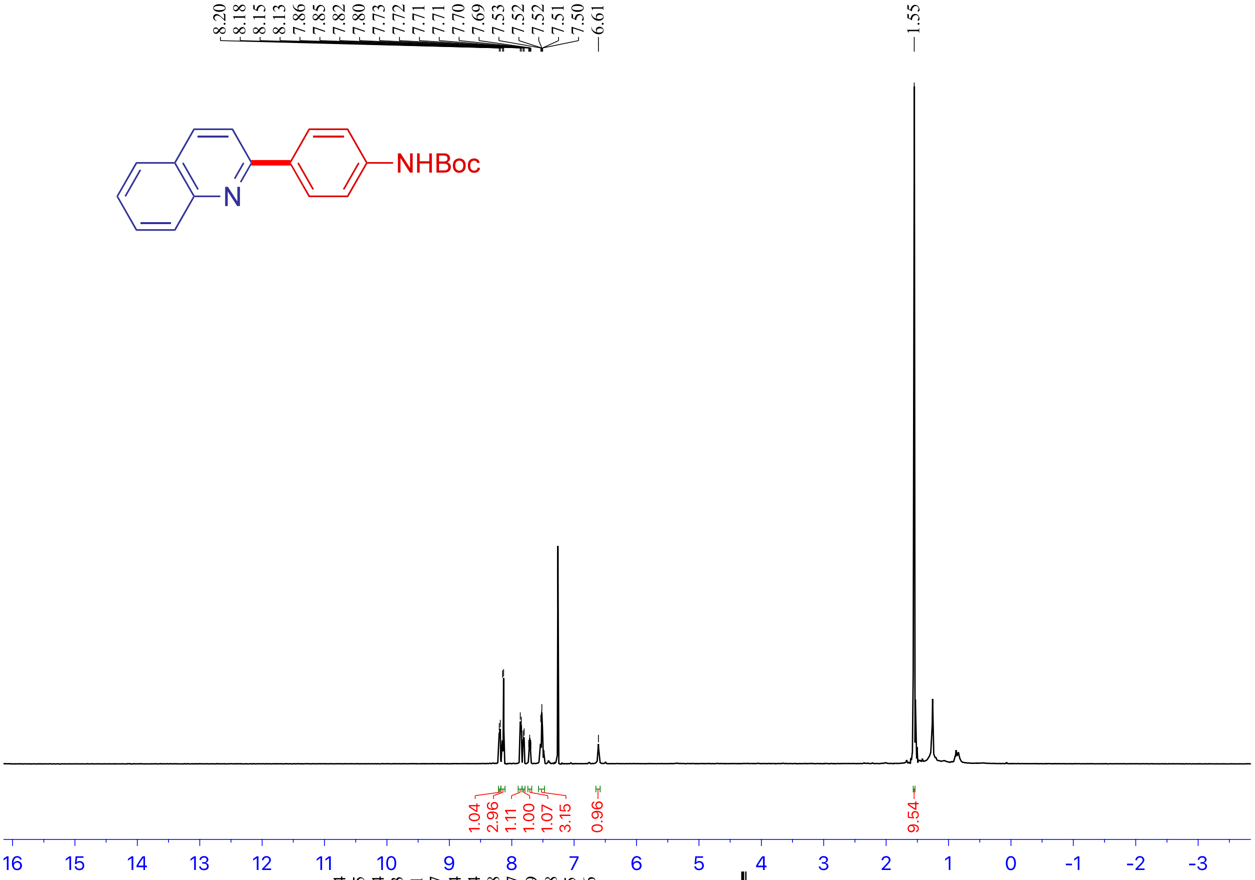

Figure S65. ^{13}C NMR spectrum of *tert*-butyl (4-(quinolin-2-yl)phenyl)carbamate, related to **Scheme 3**

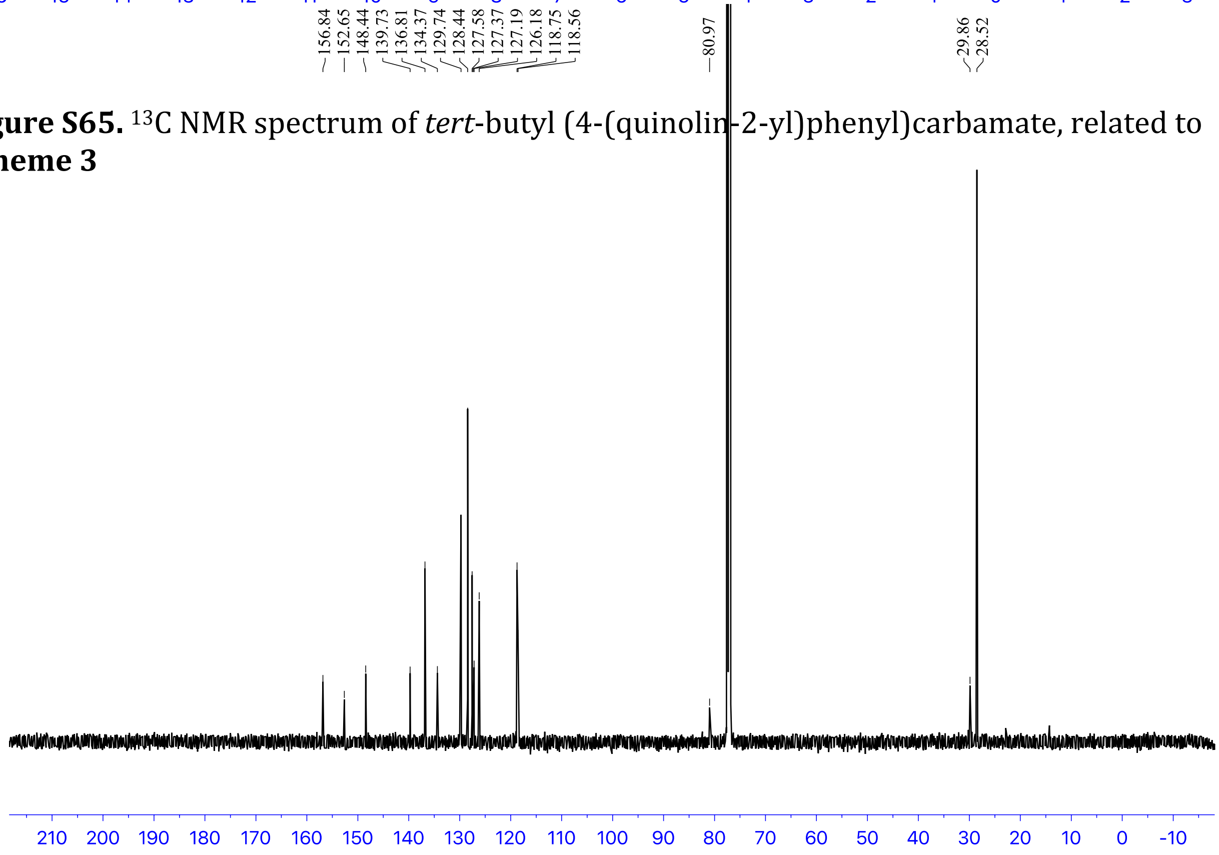

Figure S66. ^{1}H NMR spectrum of 4'-methyl-*N*-phenyl-[1,1'-biphenyl]-4-carboxamide, related to **Scheme 3**

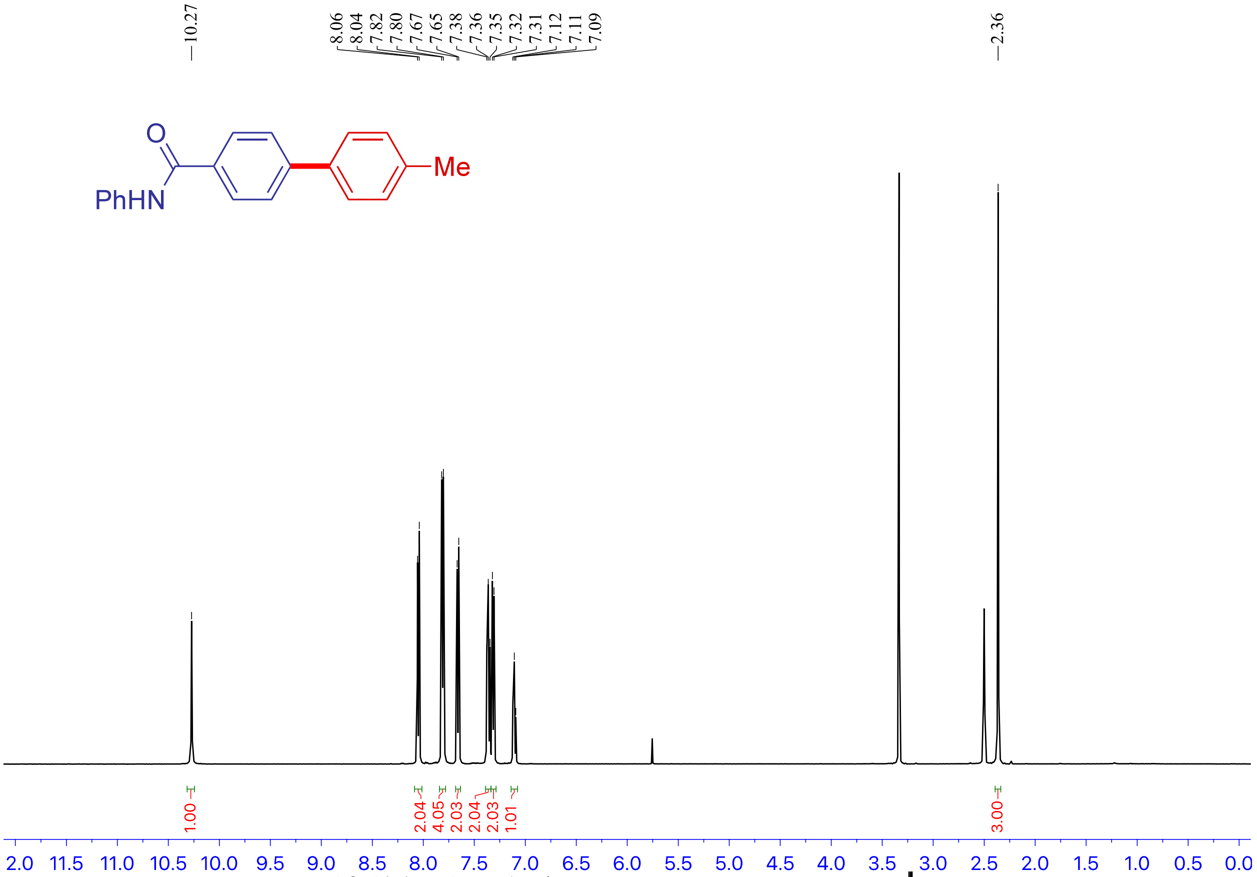

Figure S67. ^{13}C NMR spectrum of 4'-methyl-*N*-phenyl-[1,1'-biphenyl]-4-carboxamide, related to **Scheme 3**

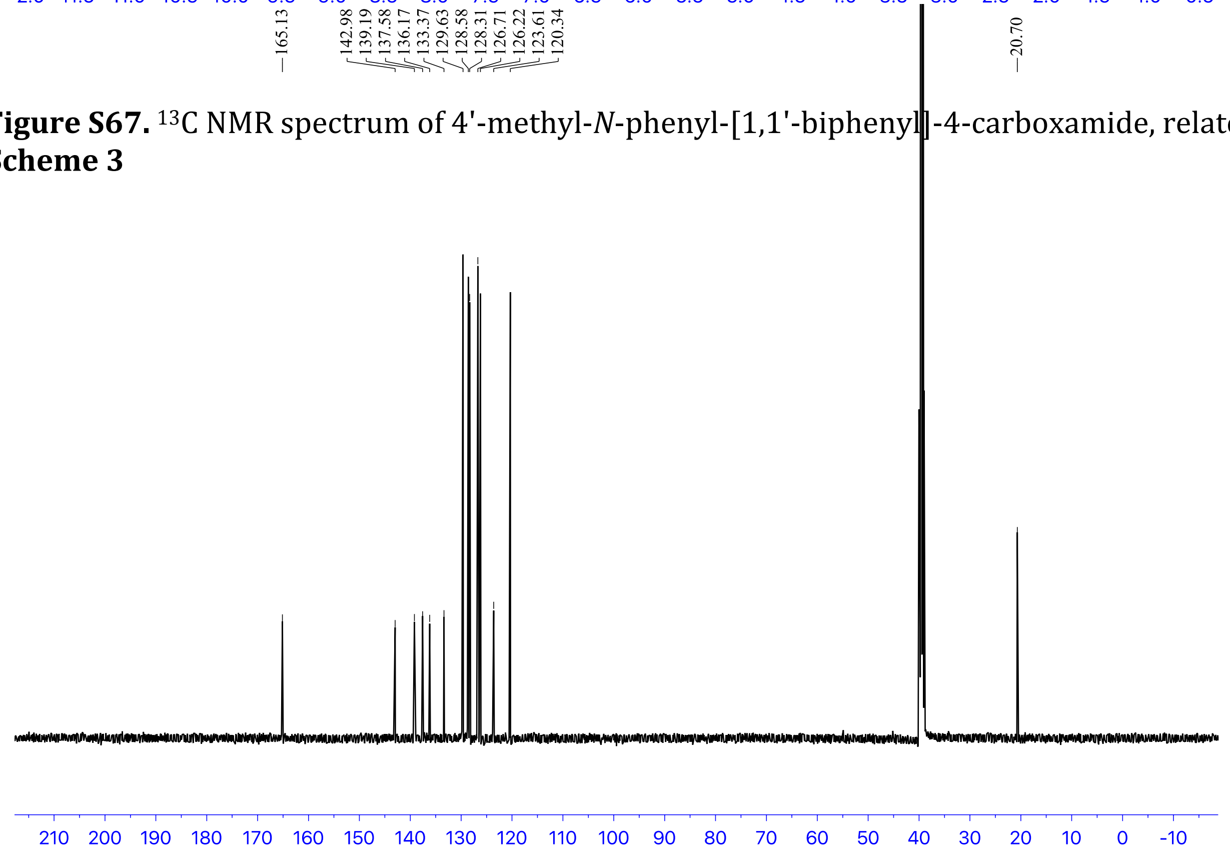

Figure S68. ^{1}H NMR spectrum of 3-methoxy-6-phenylpyridazine, related to **Scheme 3**

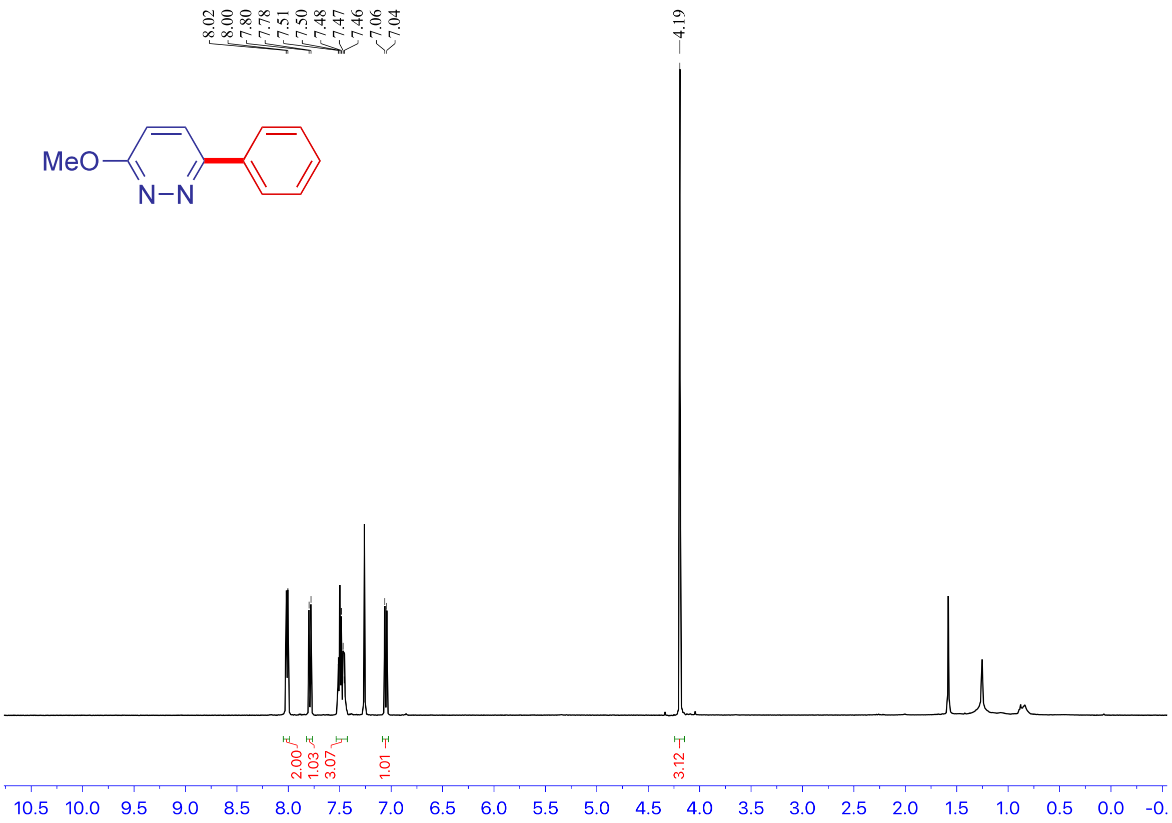

Figure S69. ^{13}C NMR spectrum of 3-methoxy-6-phenylpyridazine, related to **Scheme 3**

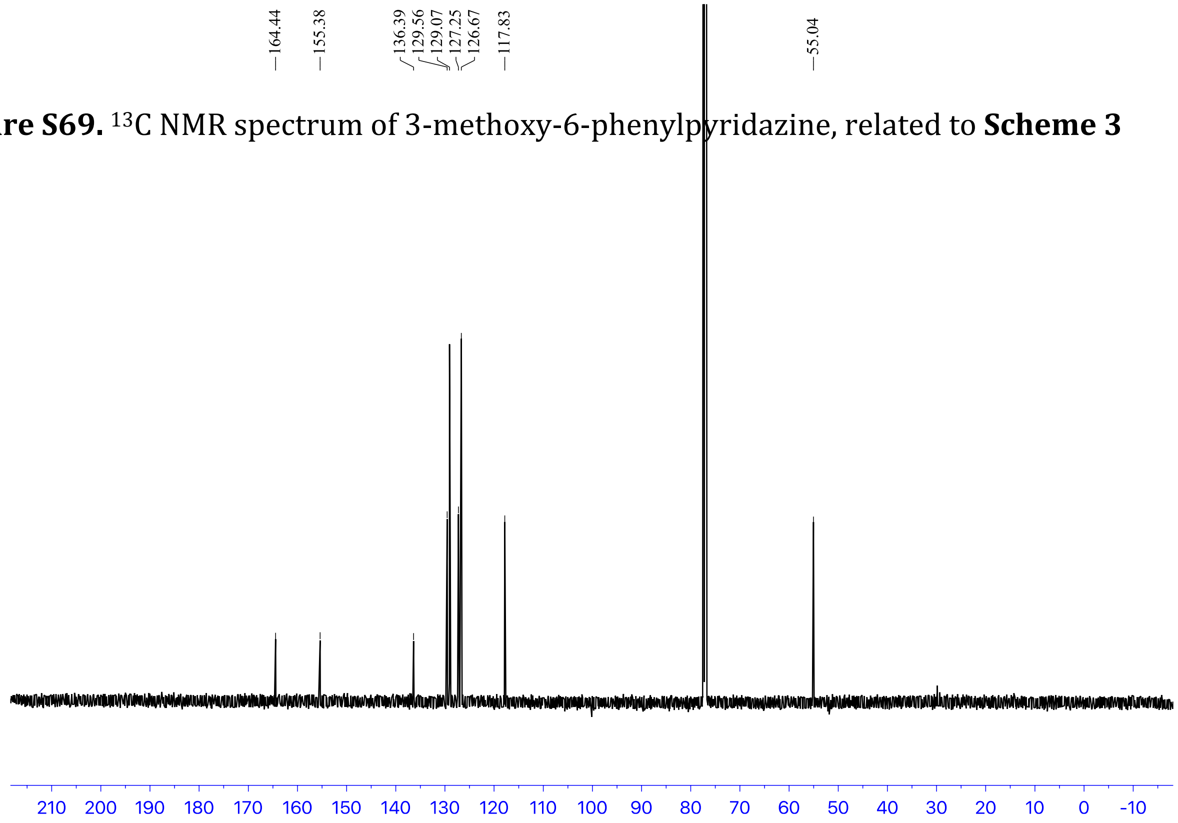

Figure S70. ^{1}H NMR spectrum of 4'-methyl-[1,1'-biphenyl]-4-carboxamide, related to **Scheme 3**

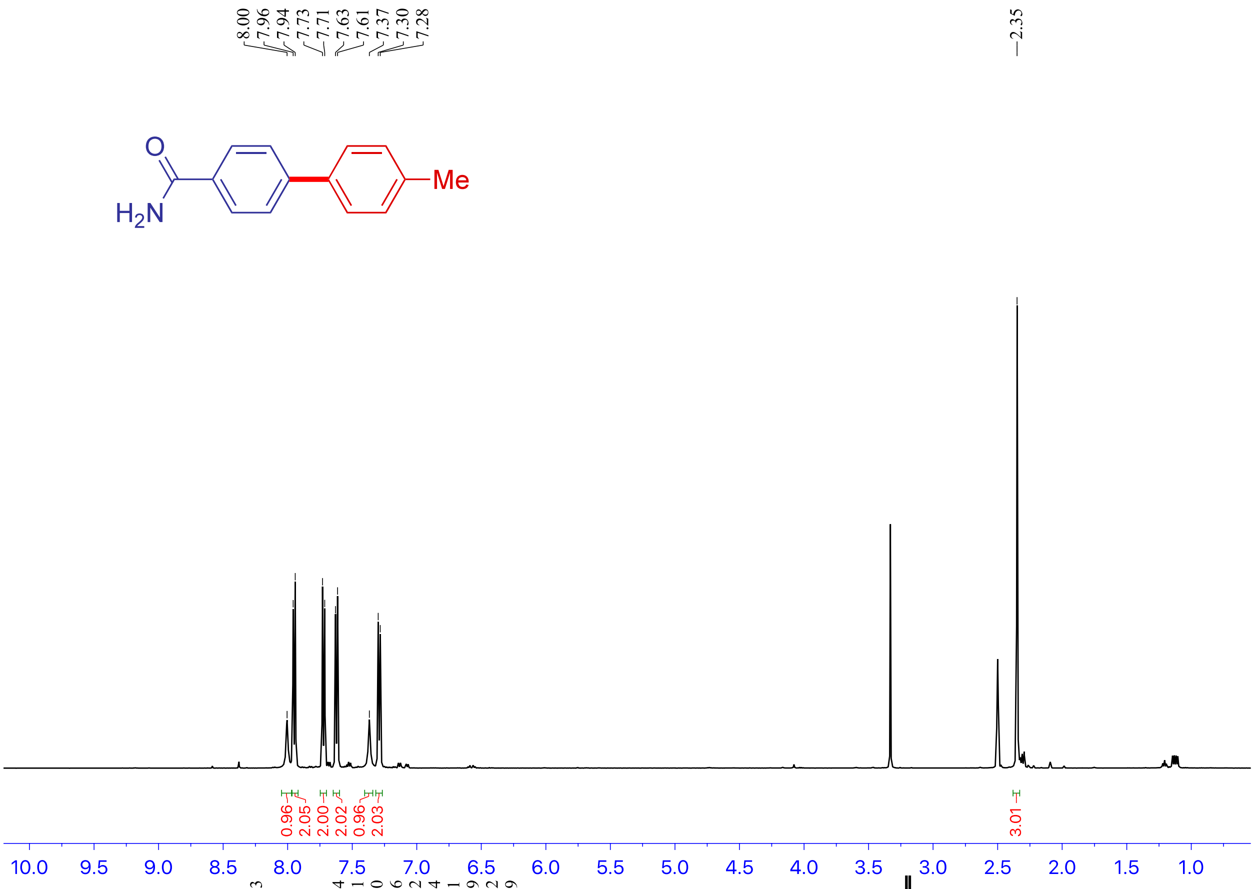

Figure S71. ^{13}C NMR spectrum of 4'-methyl-[1,1'-biphenyl]-4-carboxamide, related to **Scheme 3**

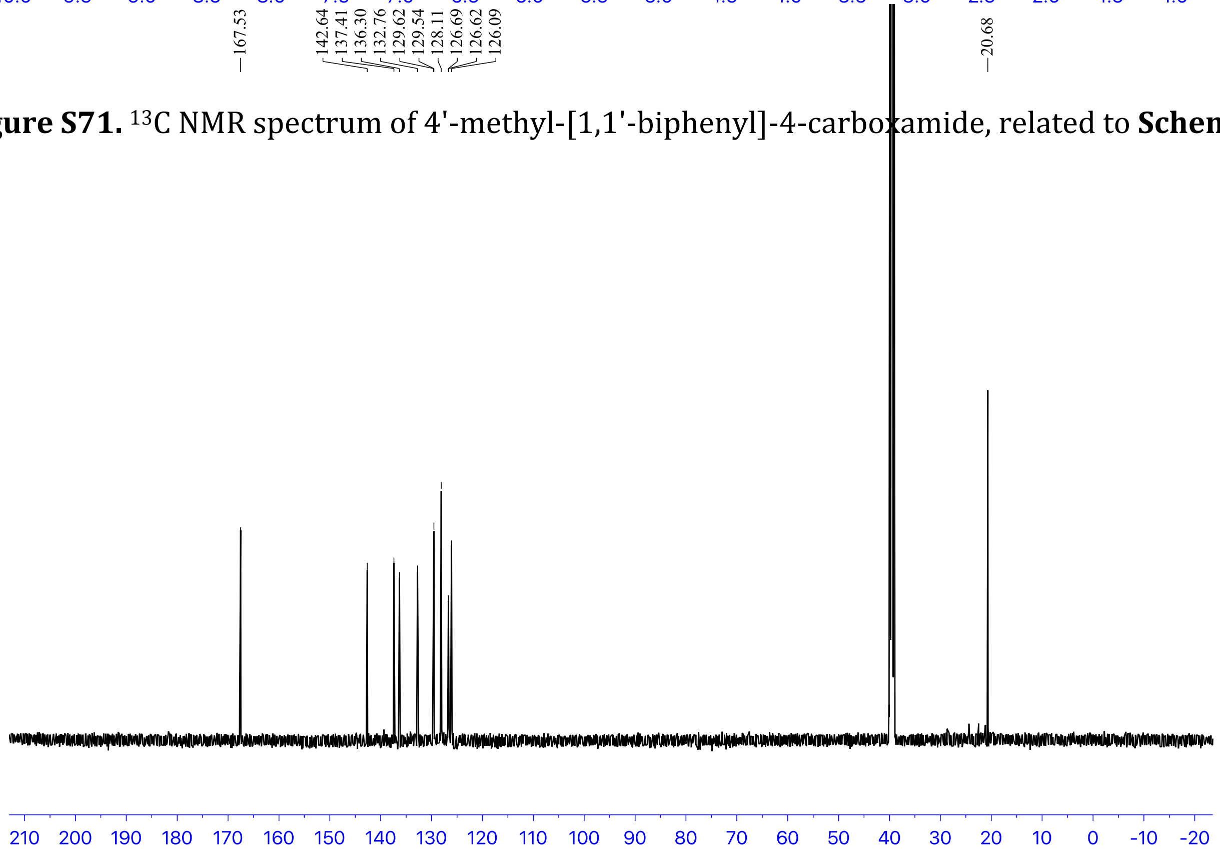

Figure S72. ^{1}H NMR spectrum of *N*-methyl-[1,1'-biphenyl]-4-carboxamide, related to **Scheme 3**

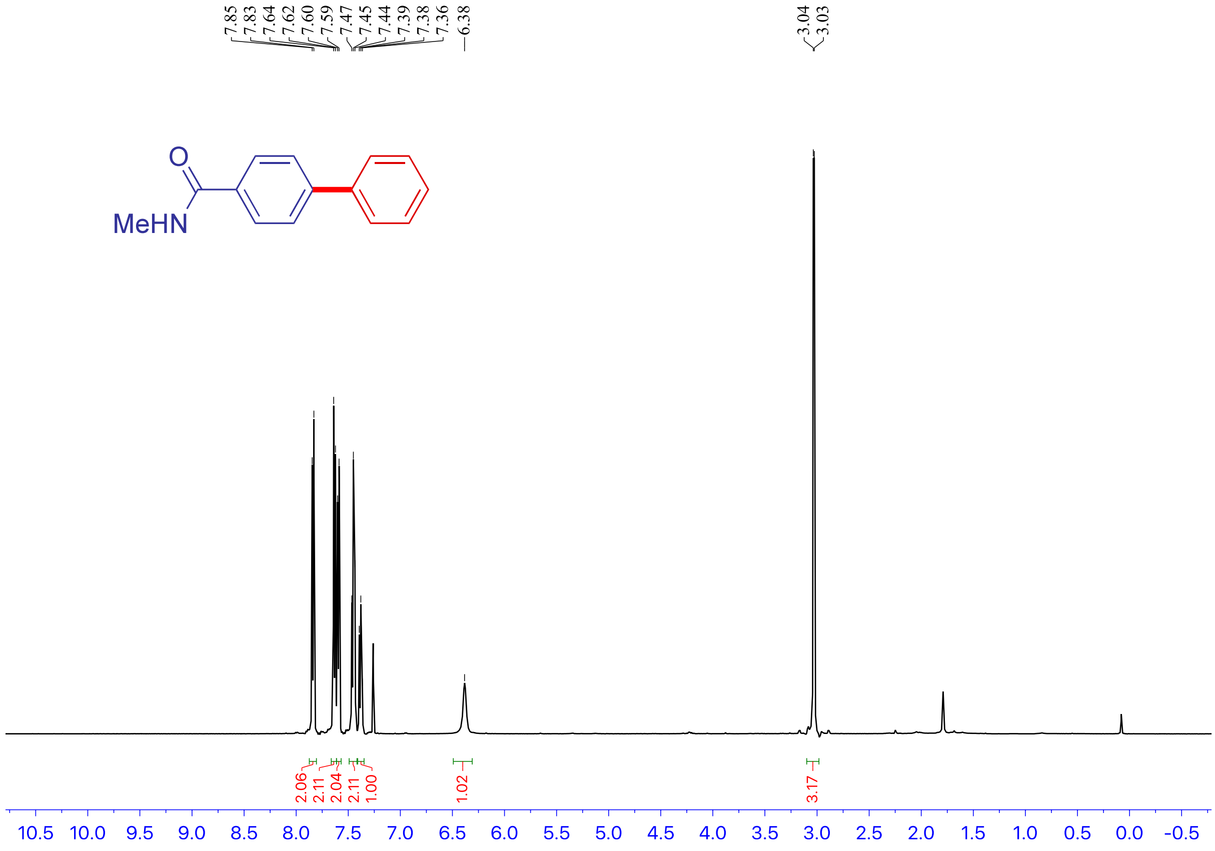

Figure S73. ^{13}C NMR spectrum of *N*-methyl-[1,1'-biphenyl]-4-carboxamide, related to **Scheme 3**

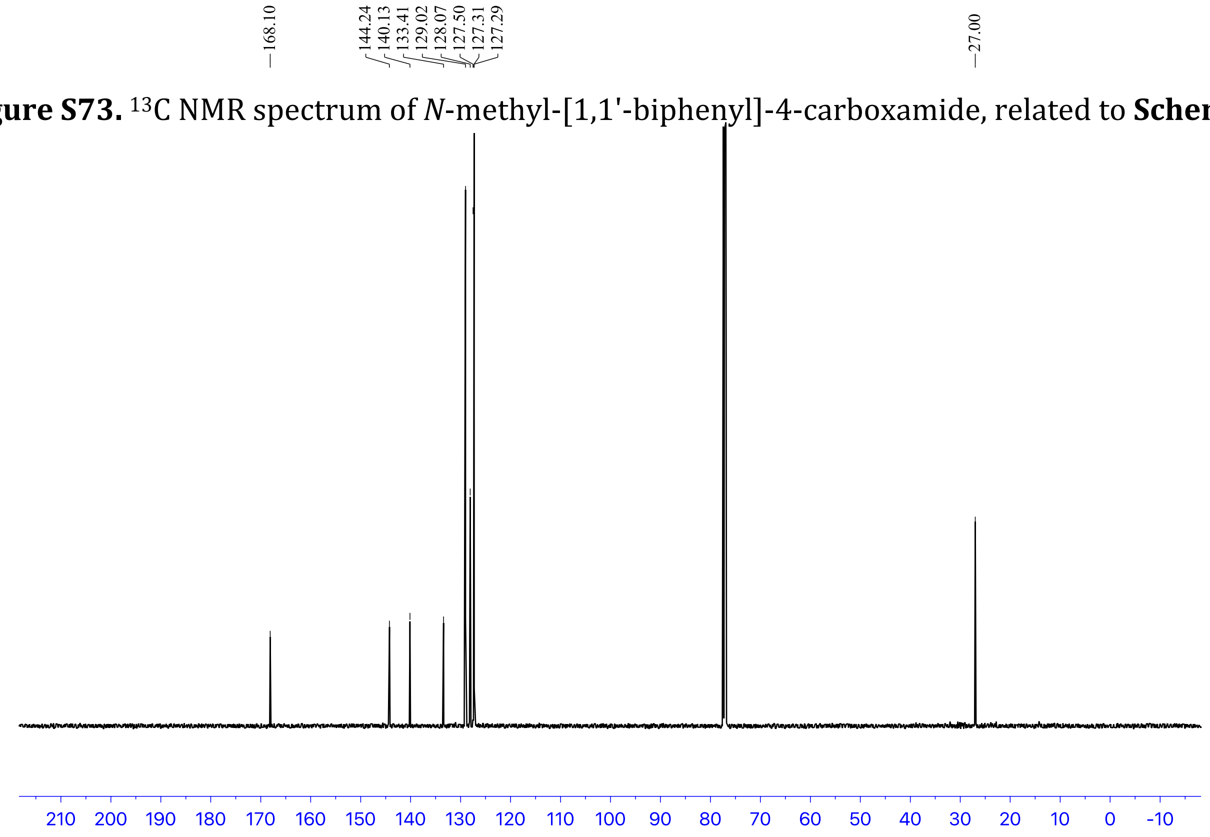

Figure S74. ${}^{1}H$ NMR spectrum of *N,N*-dimethyl-[1,1'-biphenyl]-4-carboxamide, related to **Scheme 3**

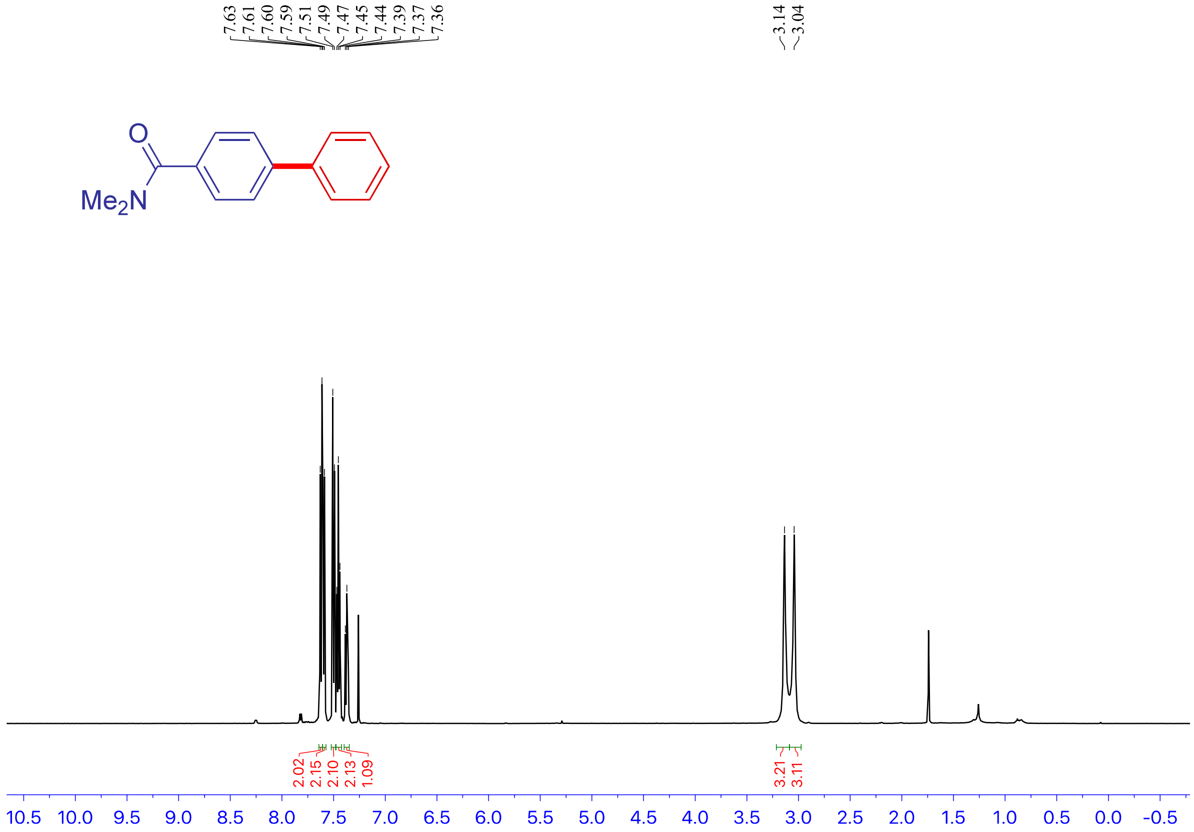

Figure S75. ${}^{13}C$ NMR spectrum of *N,N*-dimethyl-[1,1'-biphenyl]-4-carboxamide, related to **Scheme 3**

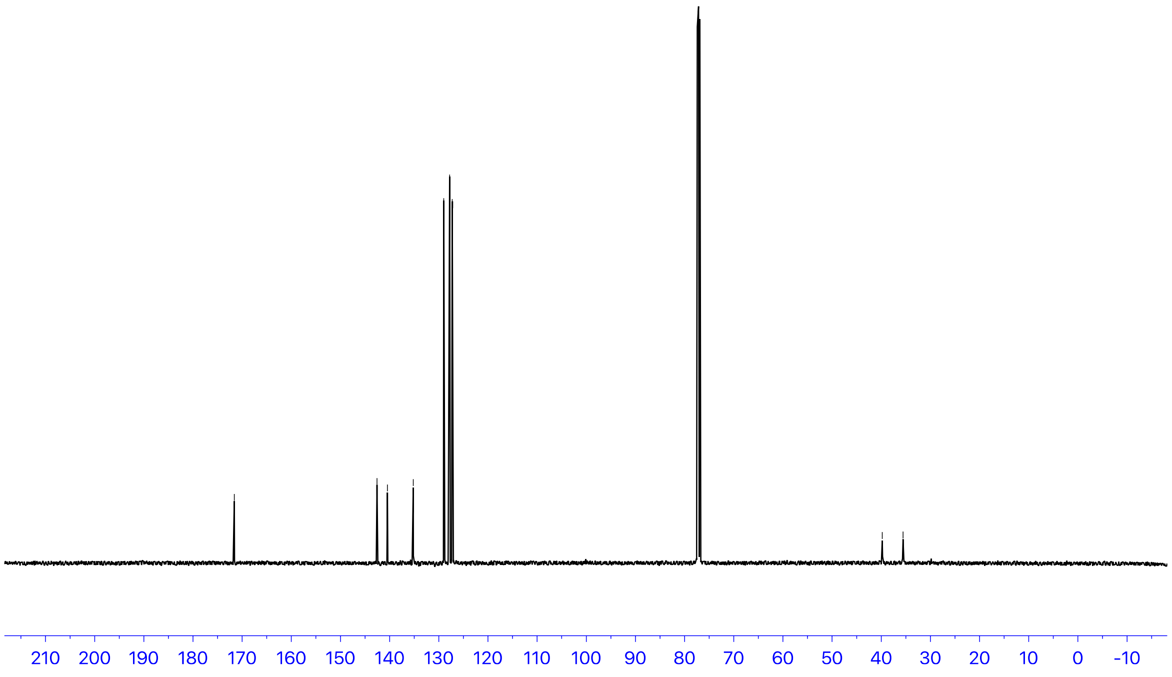

Figure S76. ^{1}H NMR spectrum of *N*-methyl-[1,1'-biphenyl]-4-sulfonamide, related to **Scheme 3**

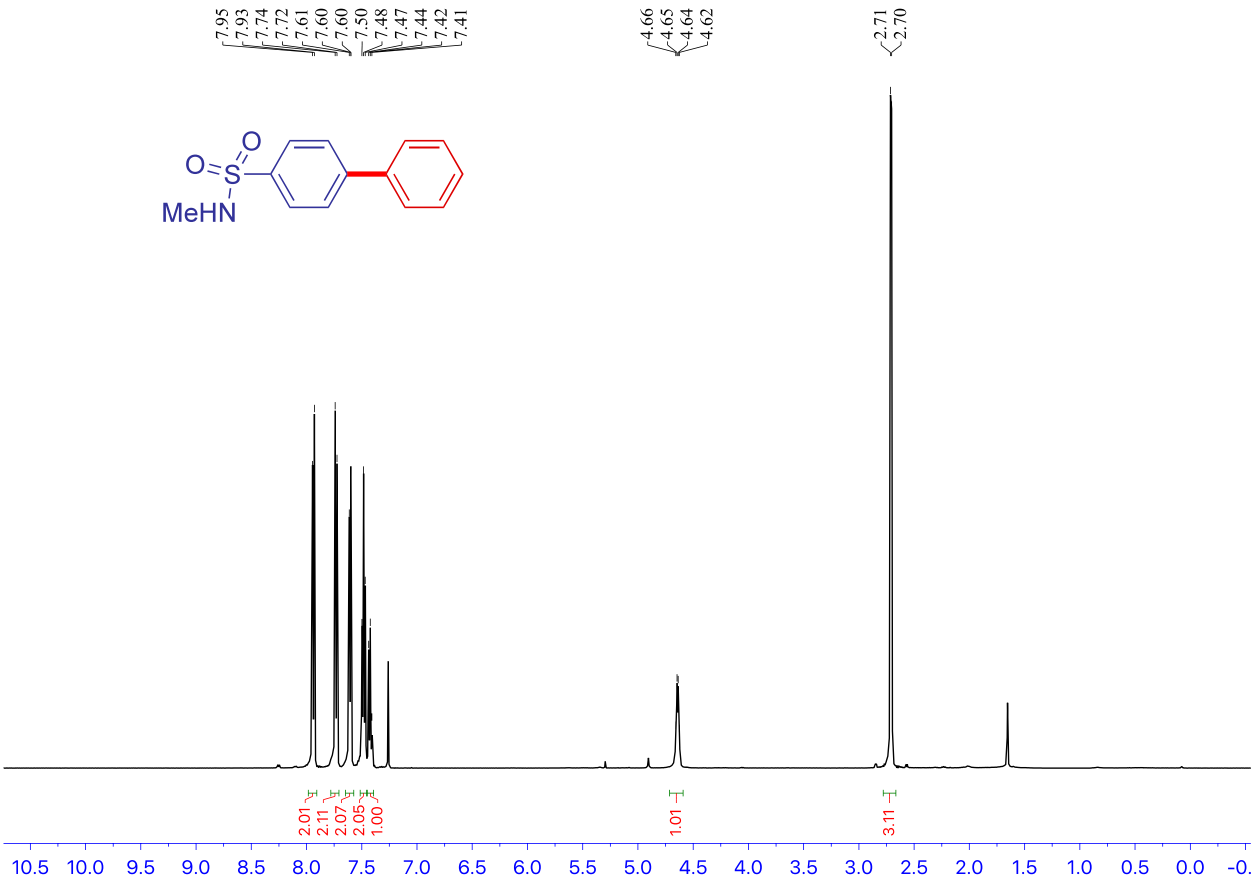

Figure S77. ^{13}C NMR spectrum of *N*-methyl-[1,1'-biphenyl]-4-sulfonamide, related to **Scheme 3**

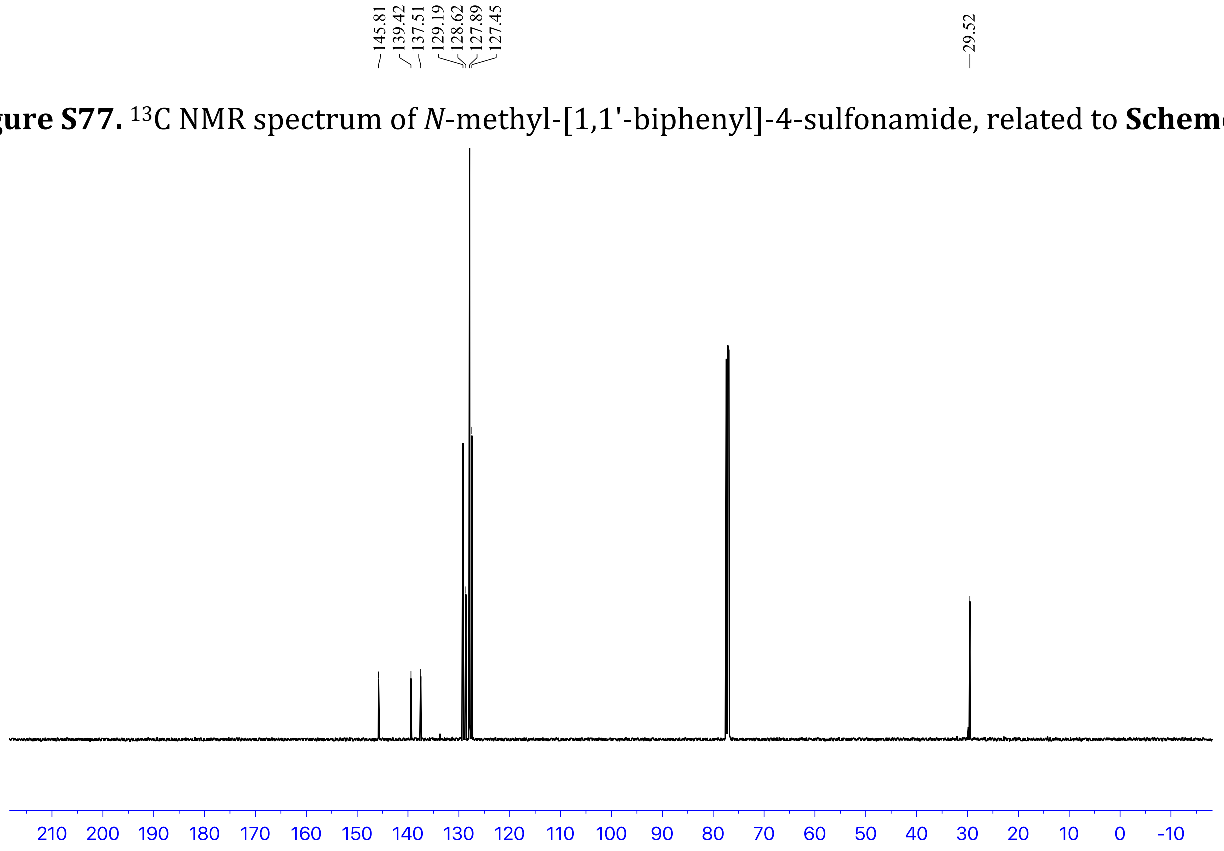

Figure S78. ^{1}H NMR spectrum of 4,7-di(thiophen-2-yl)benzo[*c*][1,2,5]thiadiazole, related to **Scheme 3**

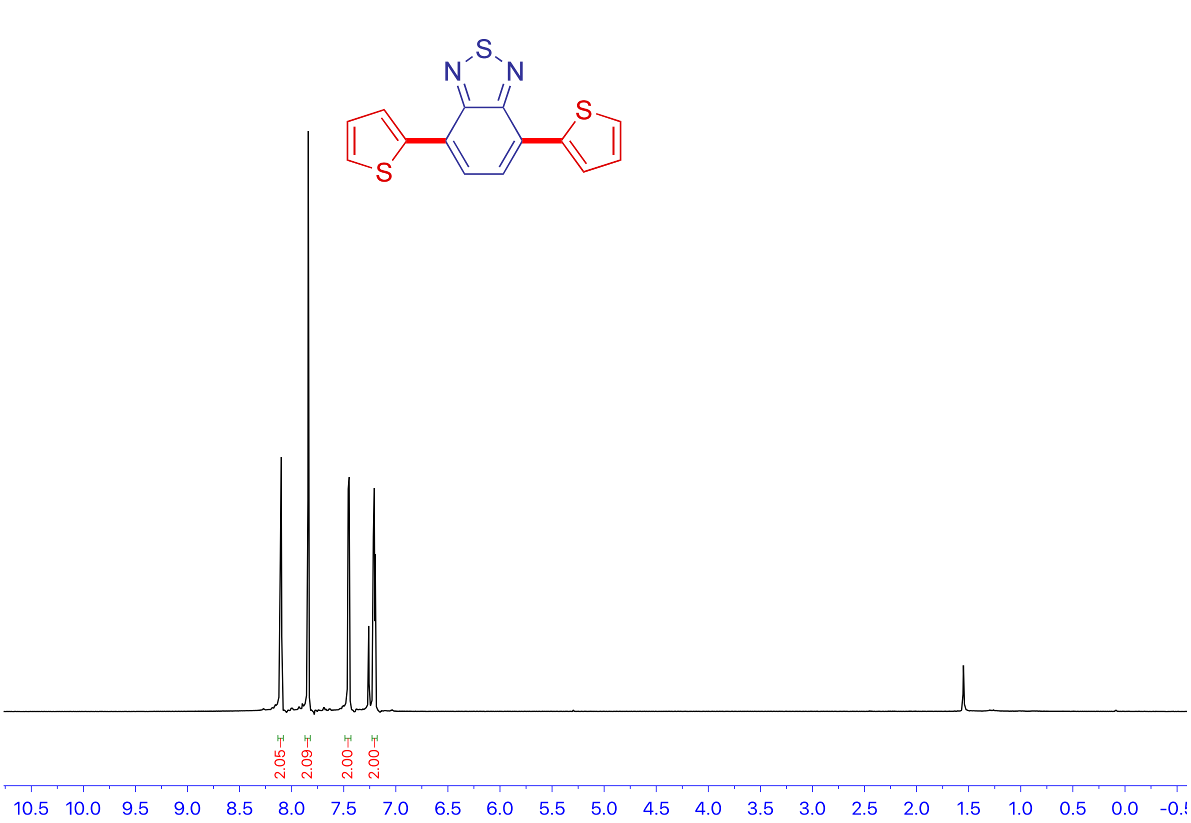

Figure S79. ^{13}C NMR spectrum of 4,7-di(thiophen-2-yl)benzo[*c*][1,2,5]thiadiazole, related to **Scheme 3**

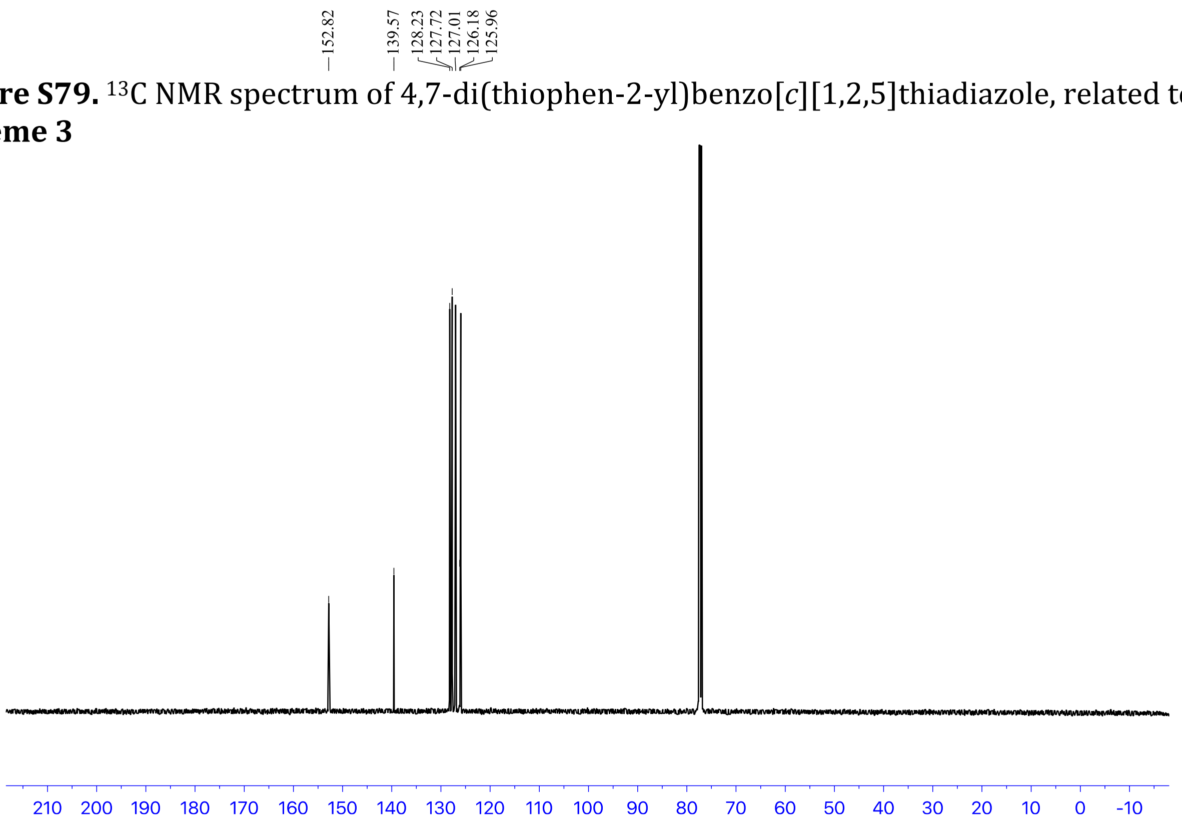

Figure S80. ^{1}H NMR spectrum of 2-(3,4,5-trifluorophenyl)pyrazine, related to **Scheme 3**

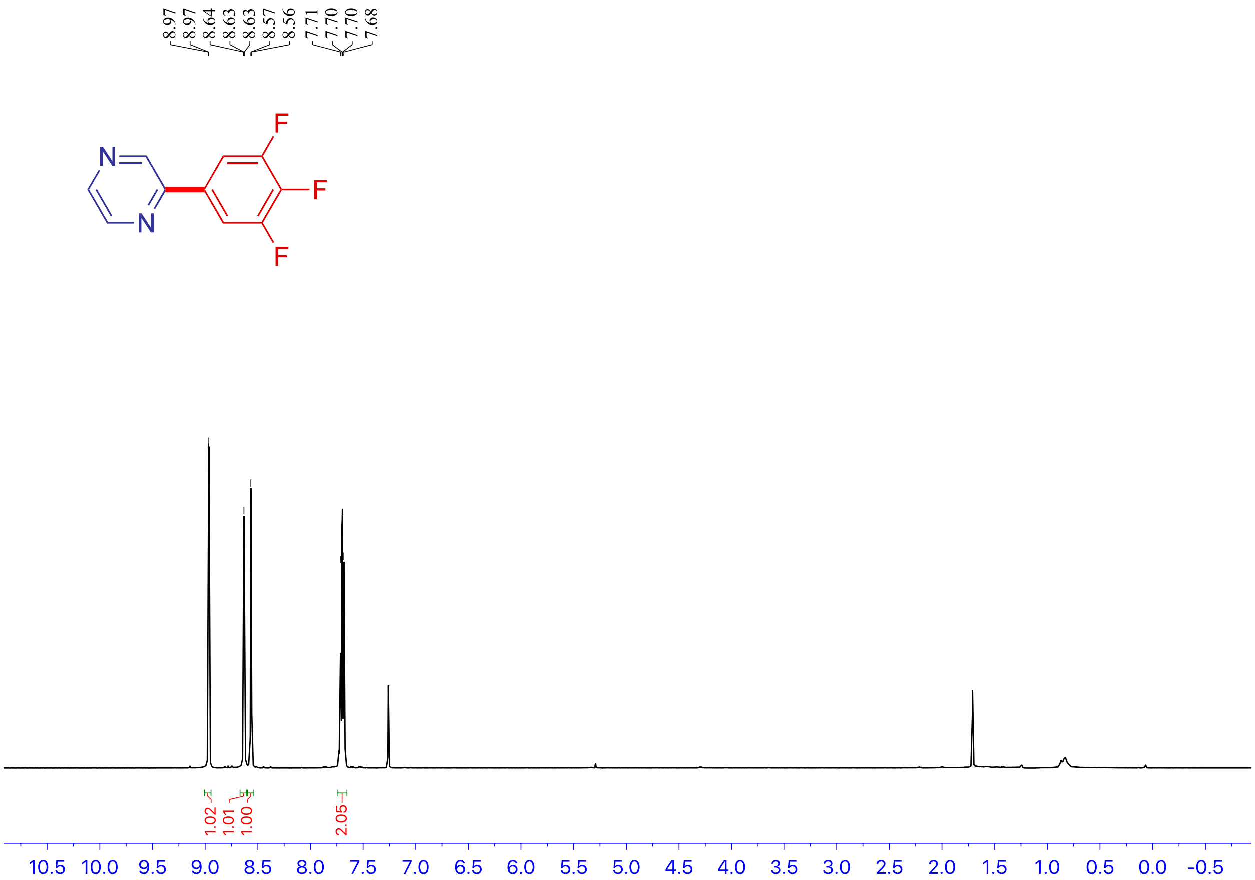

Figure S81. ^{13}C NMR spectrum of 2-(3,4,5-trifluorophenyl)pyrazine, related to **Scheme 3**

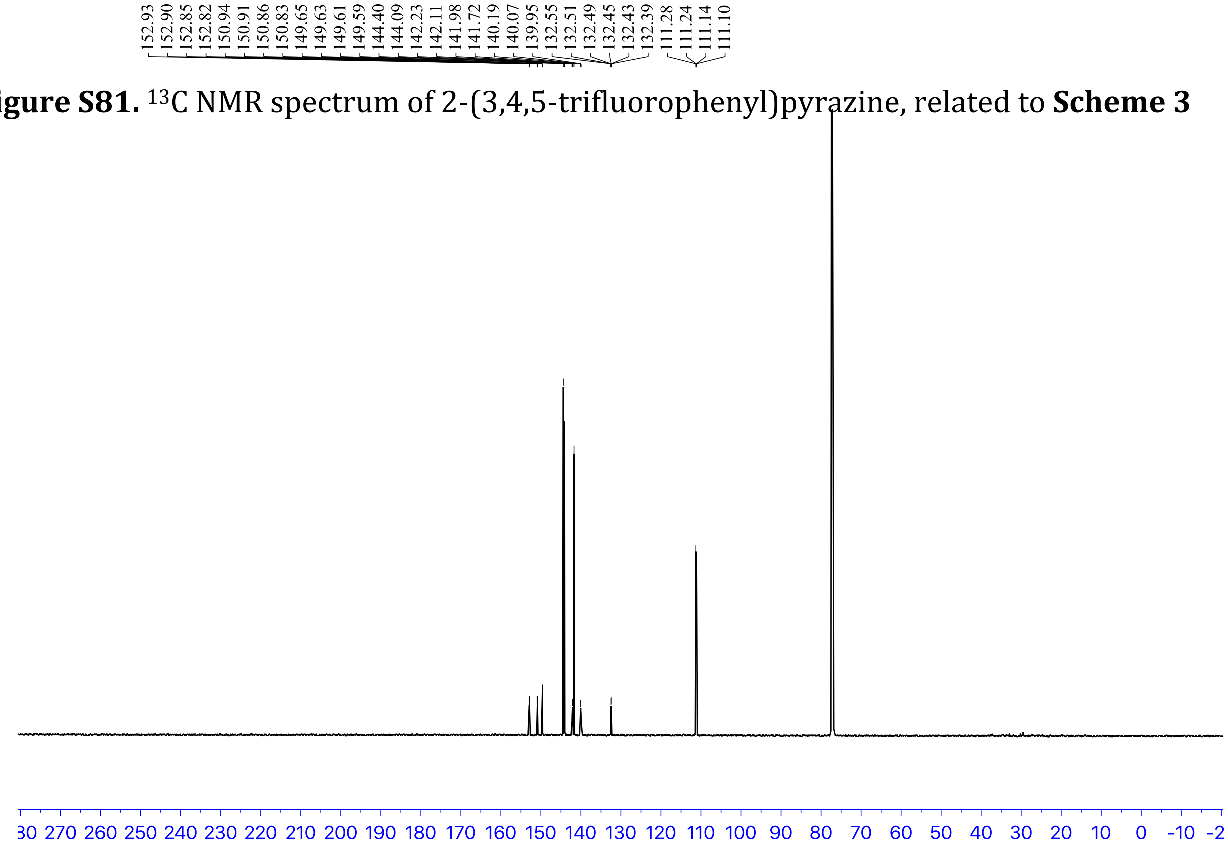

Figure S82. ^{19}F NMR spectrum of 2-(3,4,5-trifluorophenyl)pyrazine, related to **Scheme 3**

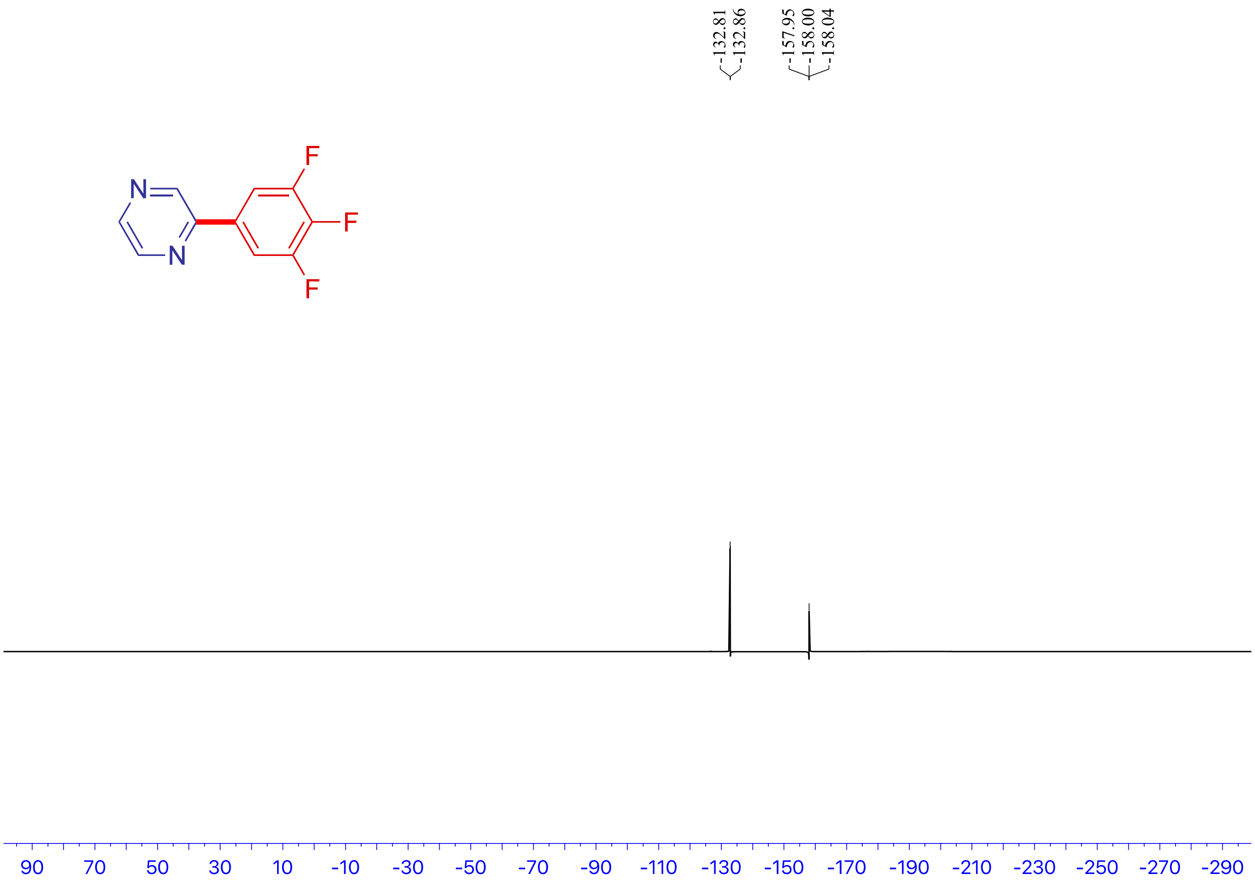

Figure S83. ^{1}H NMR spectrum of 2-(6-methoxypyridin-3-yl)pyrazine, related to **Scheme 3**

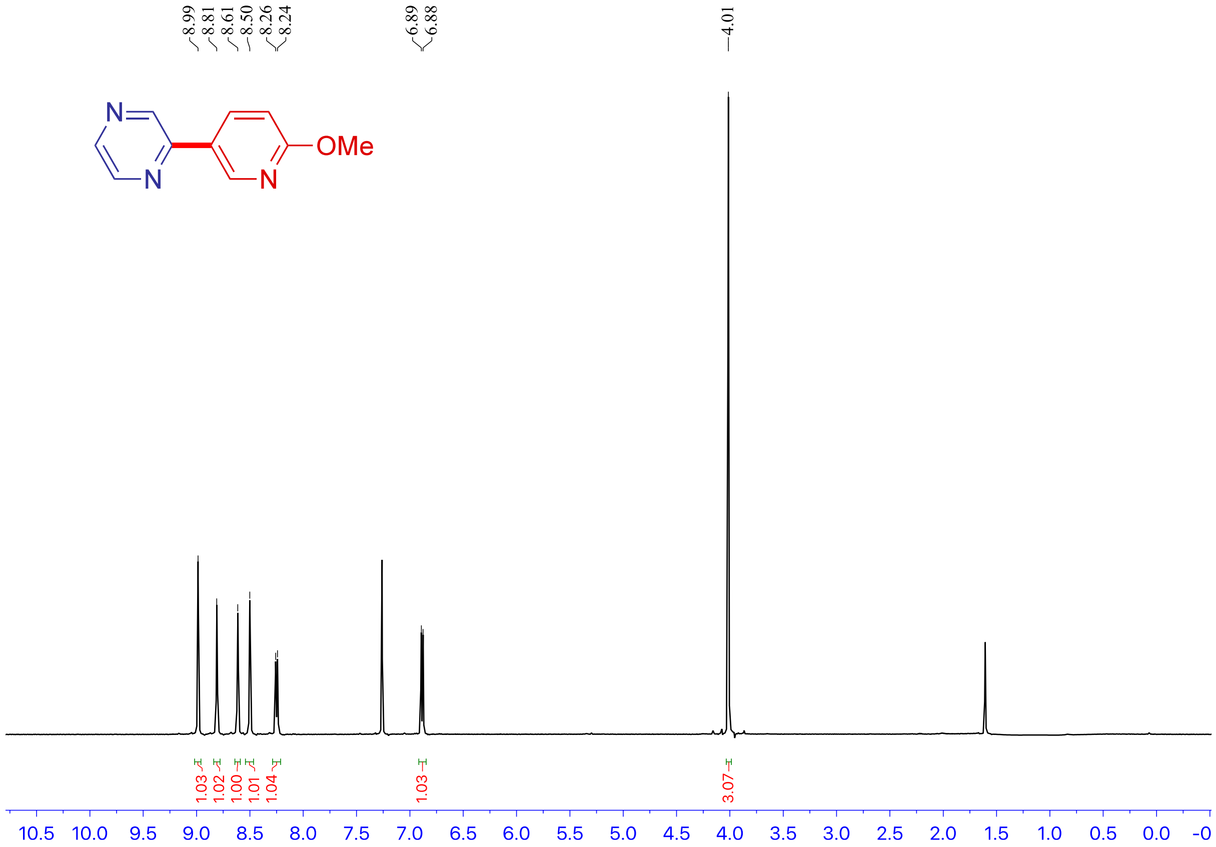

Figure S84. ^{13}C NMR spectrum of 2-(6-methoxypyridin-3-yl)pyrazine, related to **Scheme 3**

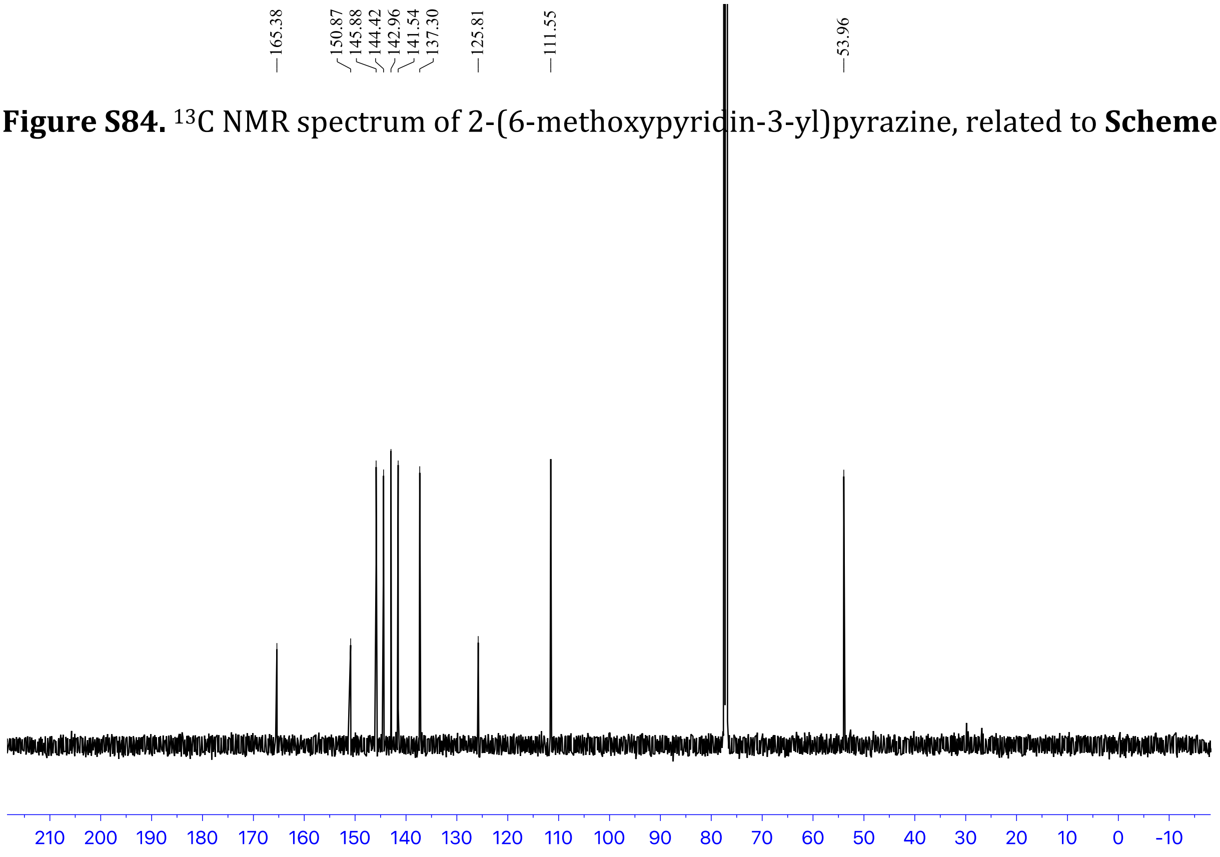

Figure S85. ^{1}H NMR spectrum of 3-(pyridin-2-yl)quinoline, related to **Scheme 3**

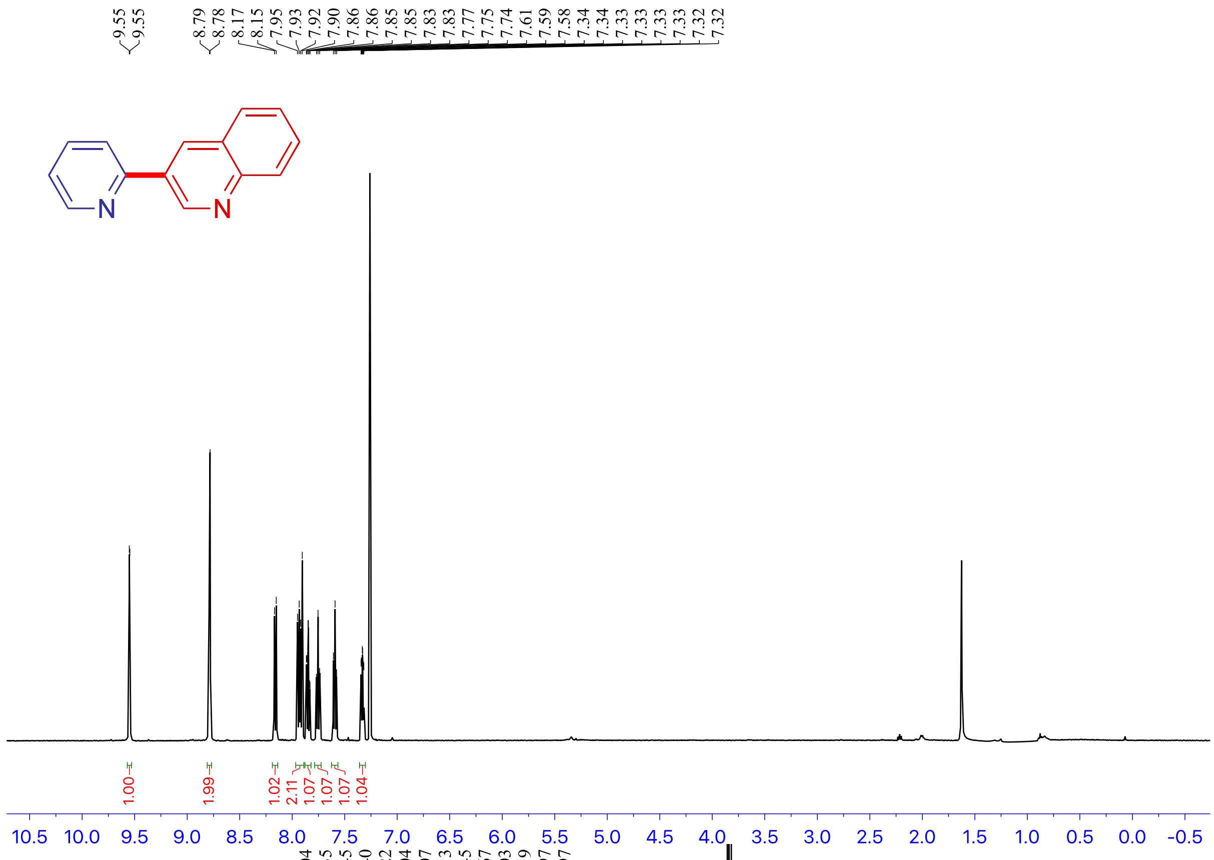

Figure S86. ^{13}C NMR spectrum of 3-(pyridin-2-yl)quinoline, related to **Scheme 3**

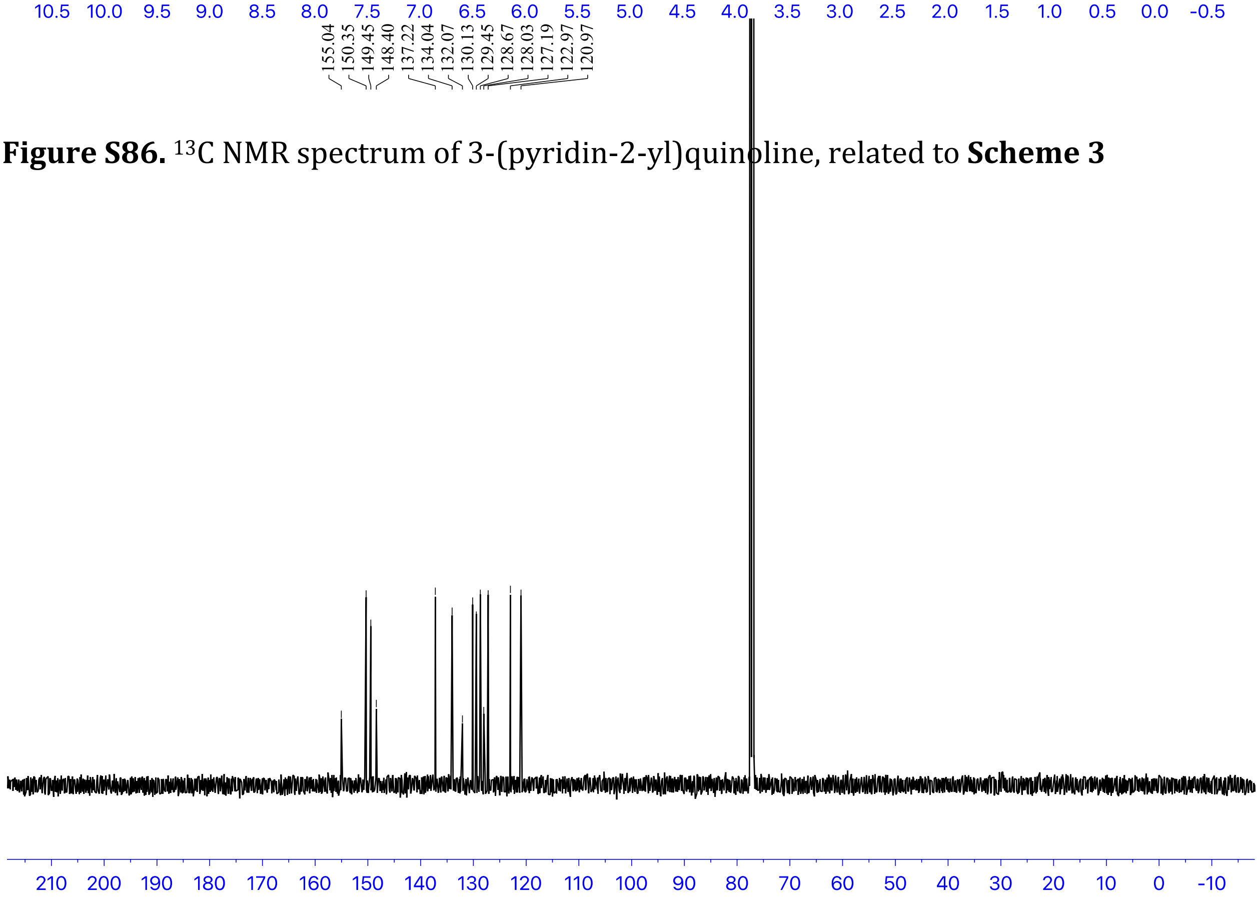

Figure S87. ^{1}H NMR spectrum of 4,6-dimethoxy-2-(naphthalen-1-yl)pyrimidine, related to **Scheme 3**

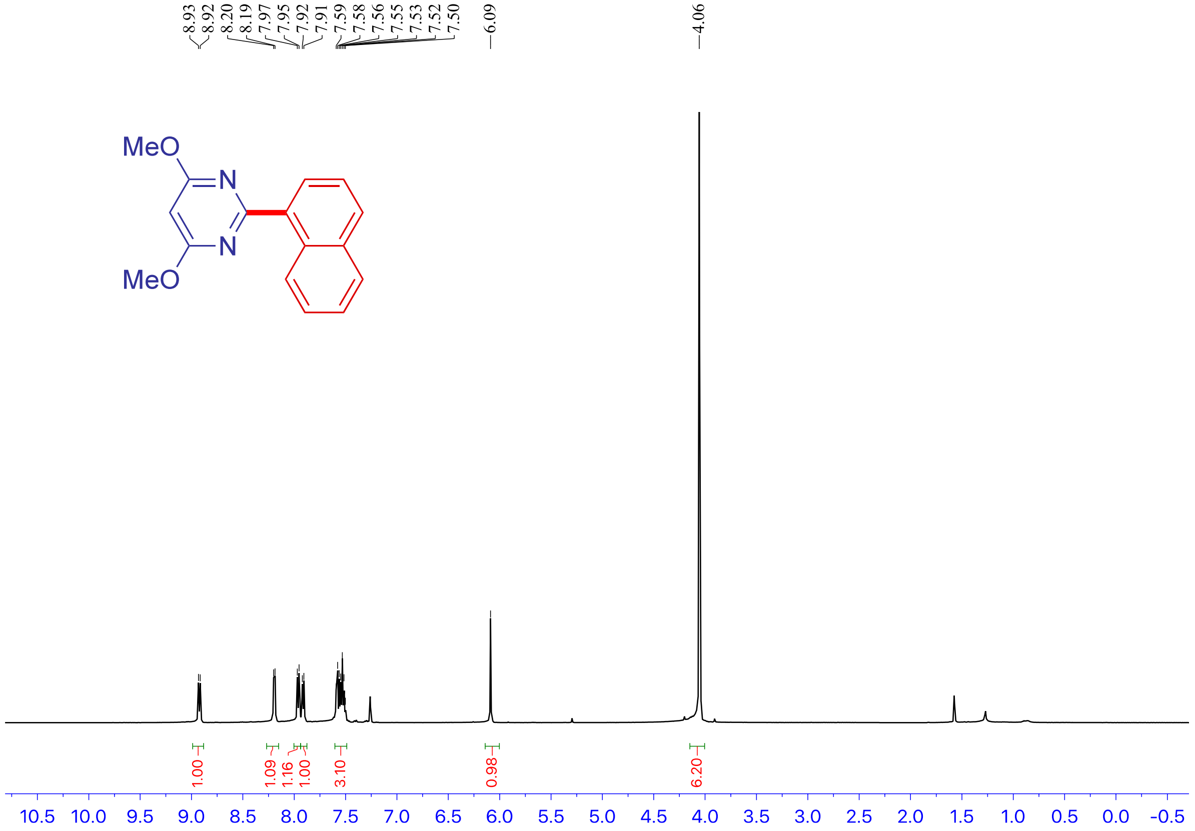

Figure S88. ^{13}C NMR spectrum of 4,6-dimethoxy-2-(naphthalen-1-yl)pyrimidine, related to **Scheme 3**

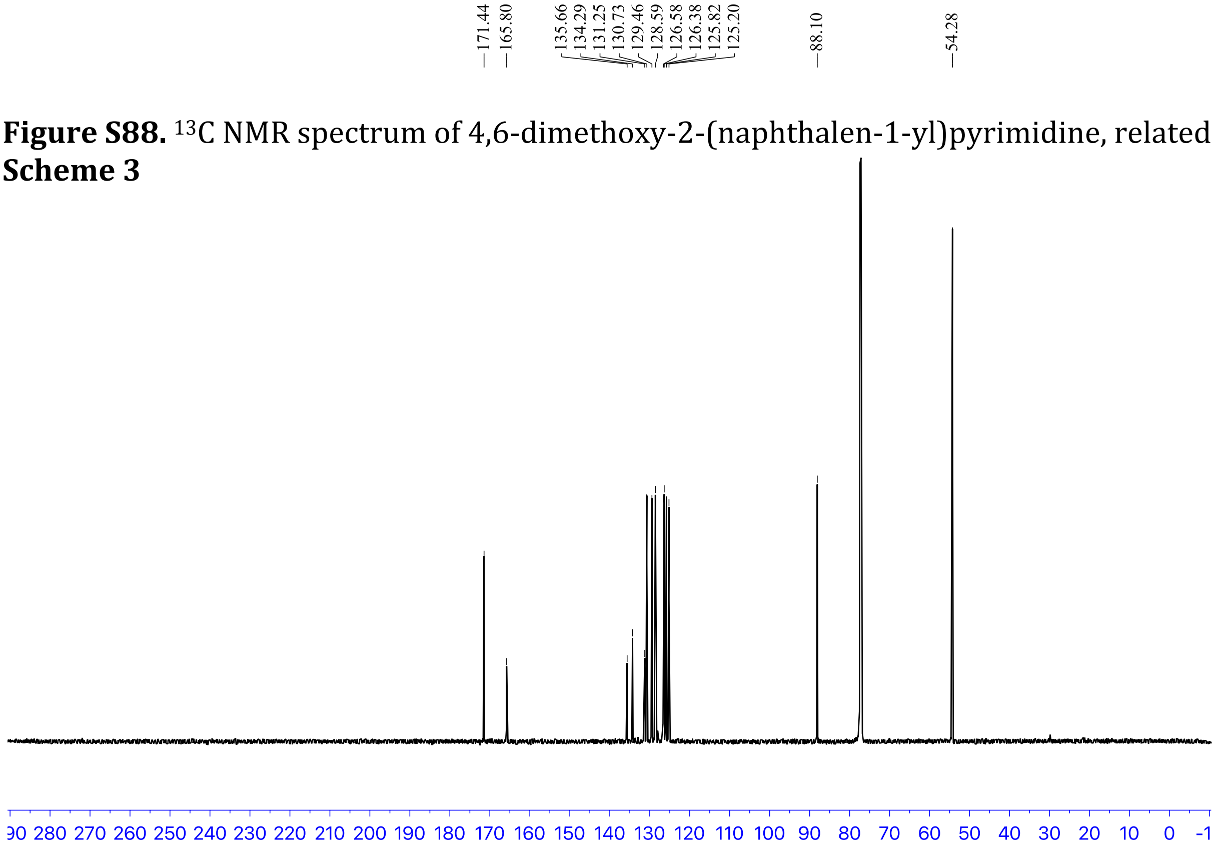

Figure S89. ^{1}H NMR spectrum of ethyl 1-methyl-5-phenyl-1*H*-indole-2-carboxylate, related to **Scheme 3**

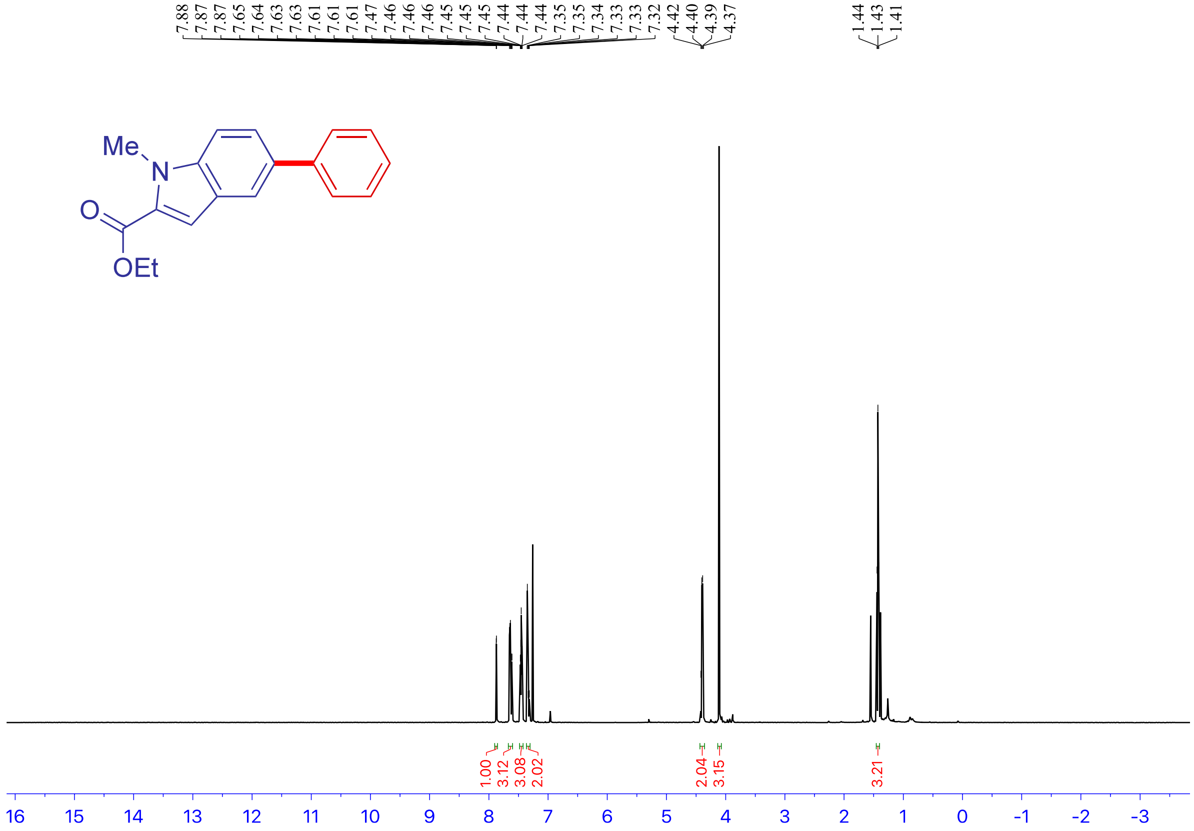

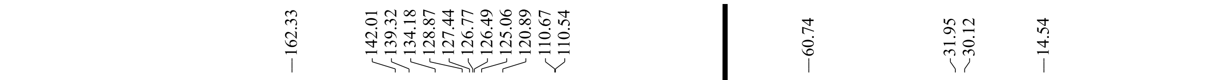

Figure S90. ^{13}C NMR spectrum of ethyl 1-methyl-5-phenyl-1*H*-indole-2-carboxylate, related to **Scheme 3**

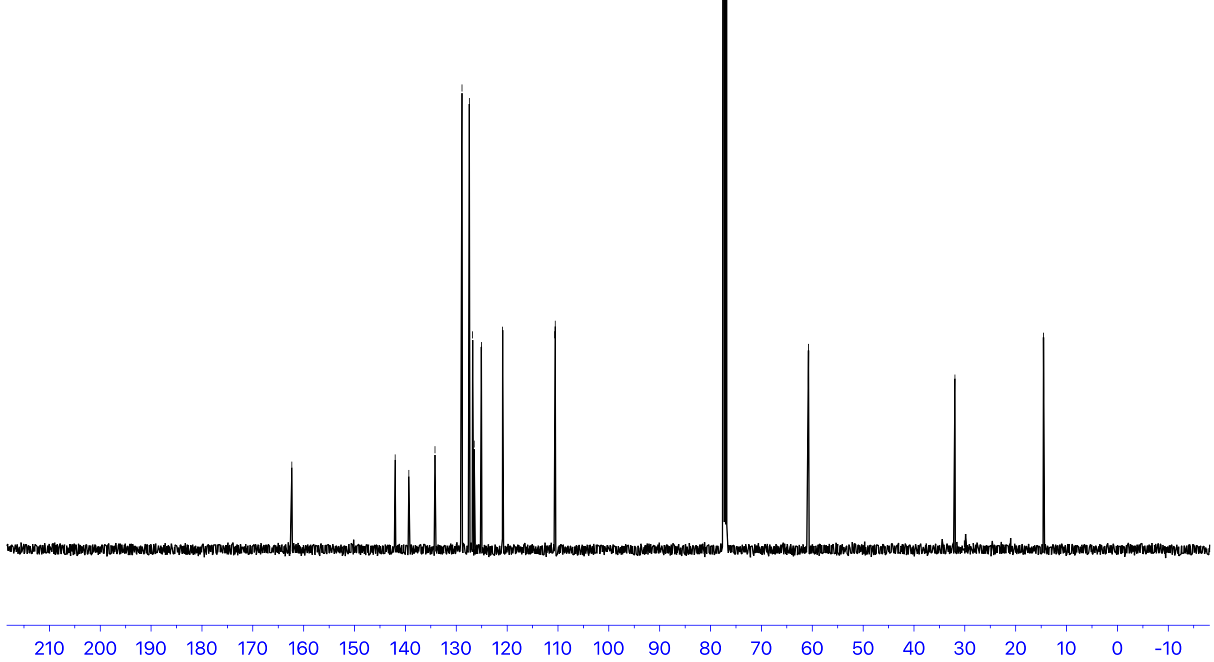

Figure S91. ^{1}H NMR spectrum of 2,4-di-*tert*-butyl-6-(5-(4-(trifluoromethyl)phenyl)-2*H*-benzo[*d*][1,2,3]triazol-2-yl)phenol, related to **Scheme 3**

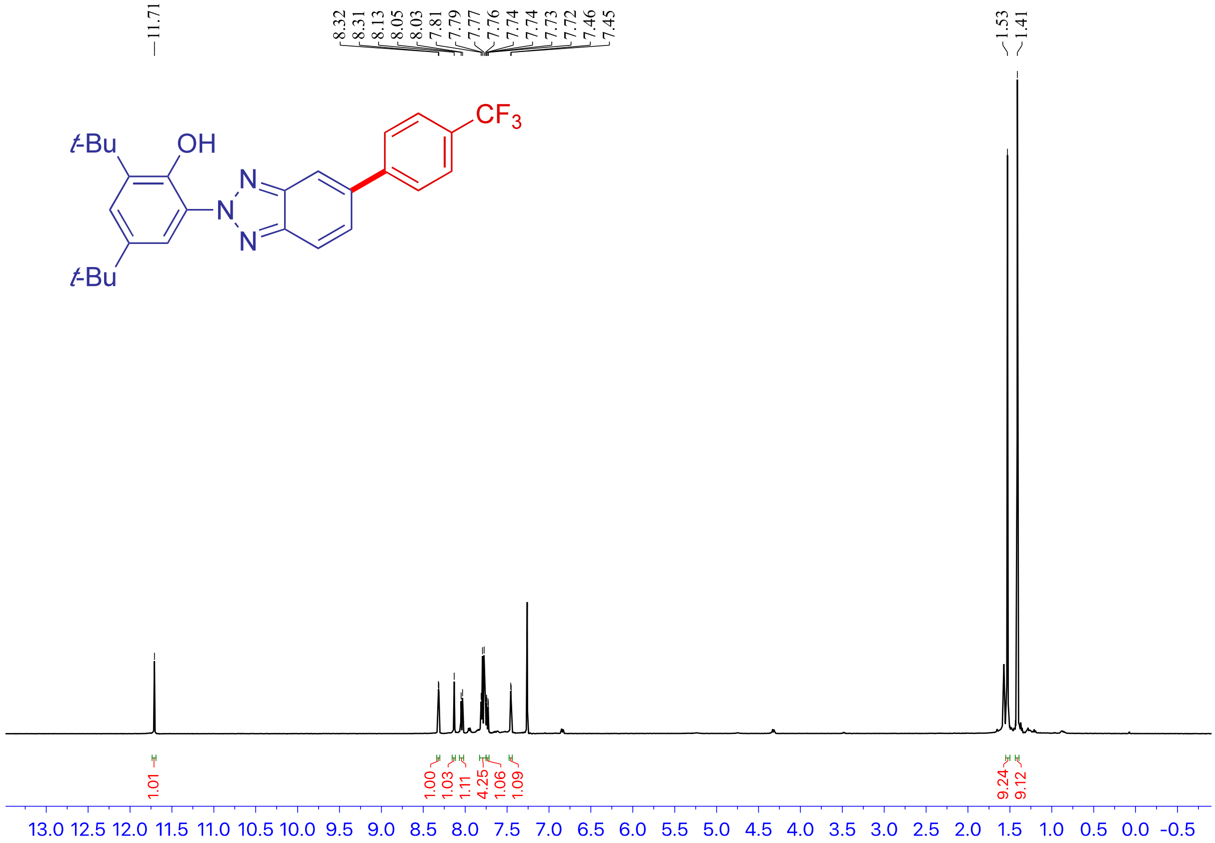

Figure S92. ^{13}C NMR spectrum of 2,4-di-*tert*-butyl-6-(5-(4-(trifluoromethyl)phenyl)-2*H*-benzo[*d*][1,2,3]triazol-2-yl)phenol, related to **Scheme 3**

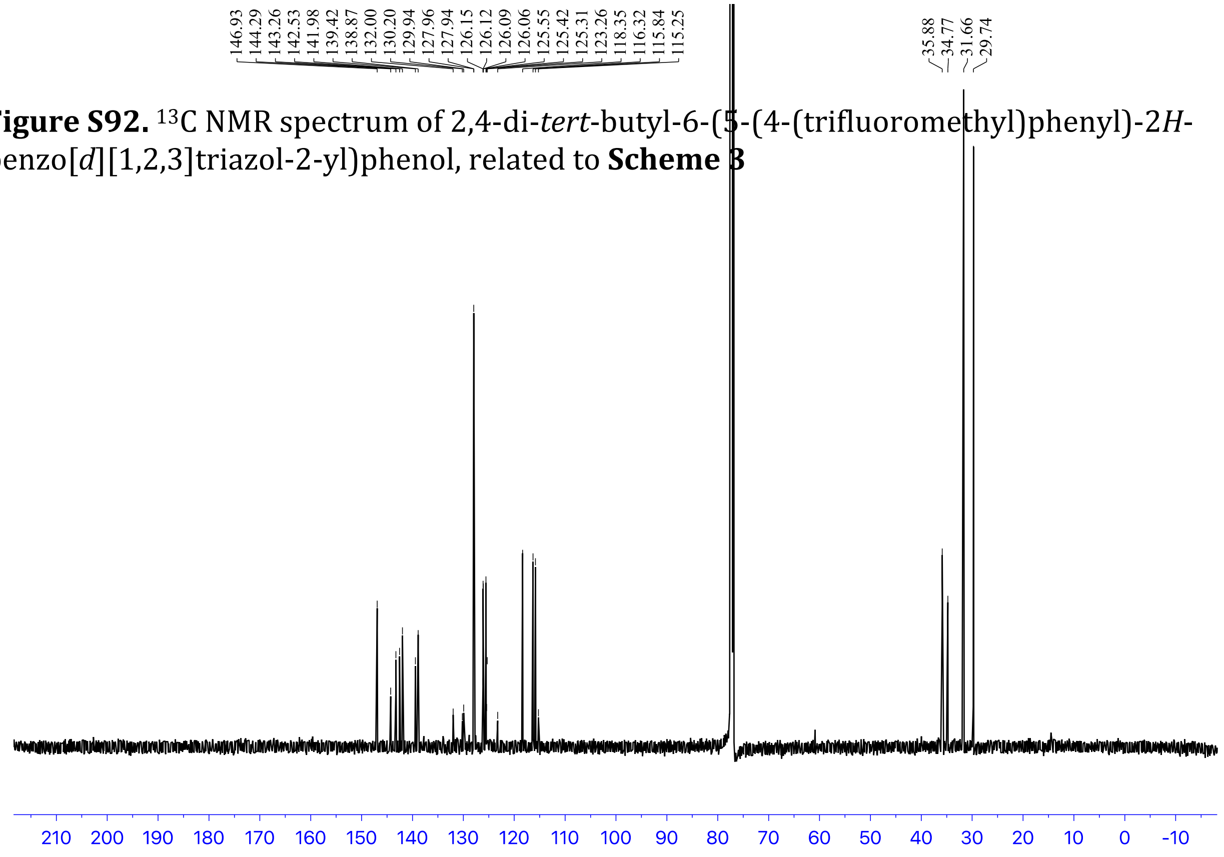

Figure S93. ^{19}F NMR spectrum of 2,4-di-*tert*-butyl-6-(5-(4-(trifluoromethyl)phenyl)-2*H*-benzo[*d*][1,2,3]triazol-2-yl)phenol, related to **Scheme 3**

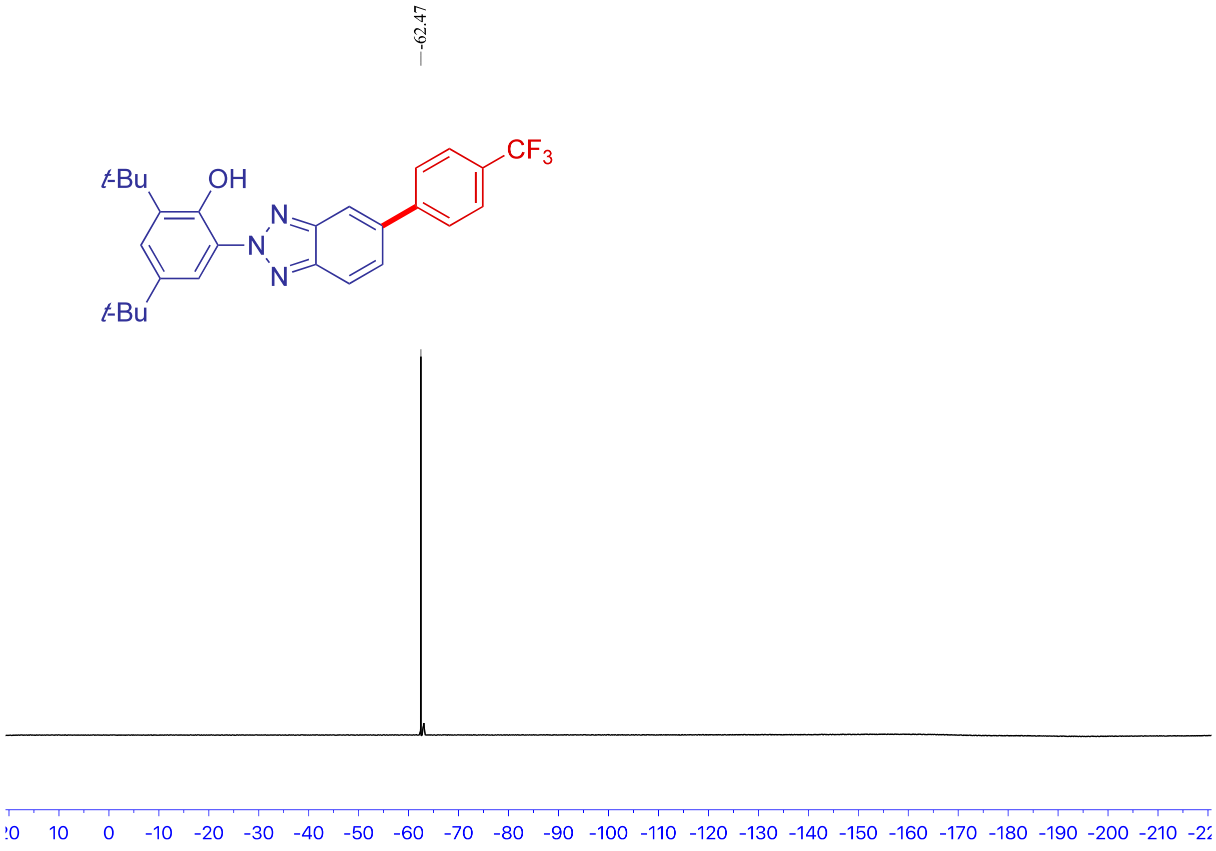

Figure S94. ^{1}H NMR spectrum of 4'-methoxy-3,5-bis(trifluoromethyl)biphenyl, related to **Scheme 3**

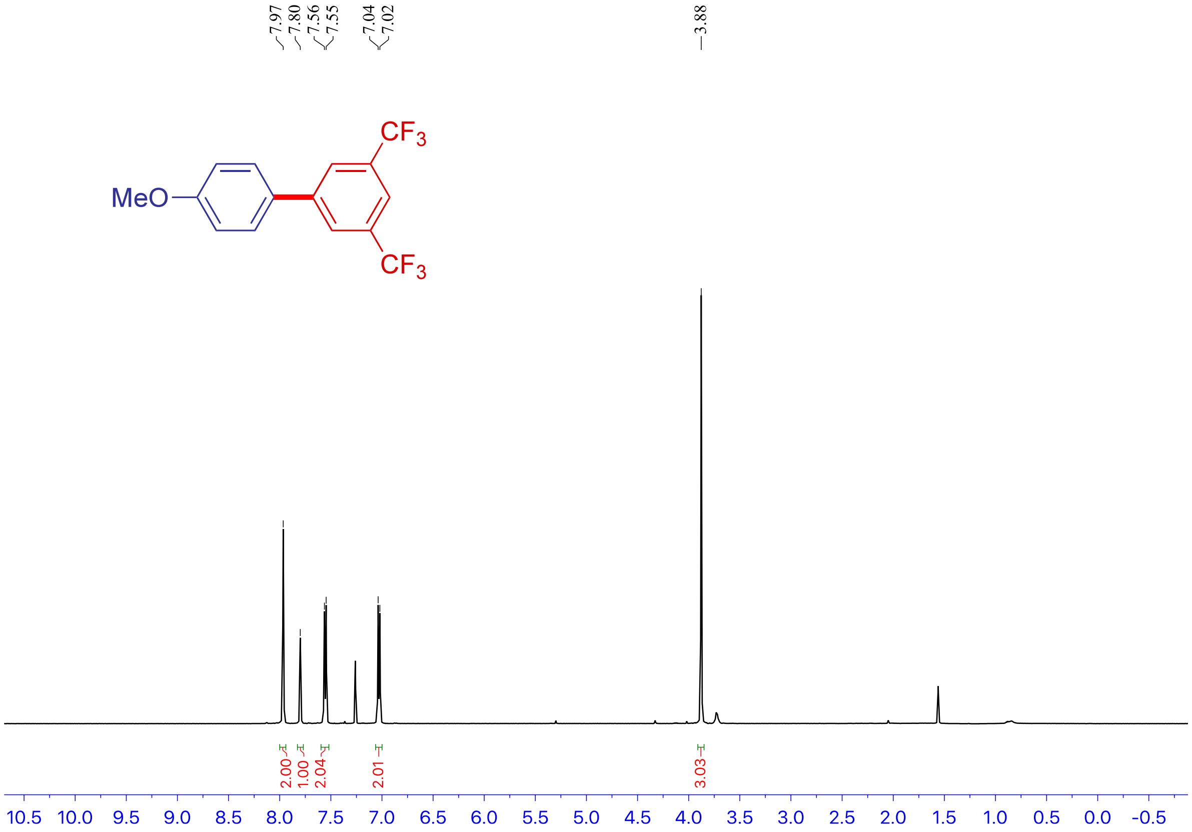

Figure S95. ^{13}C NMR spectrum of 4'-methoxy-3,5-bis(trifluoromethyl)biphenyl, related to **Scheme 3**

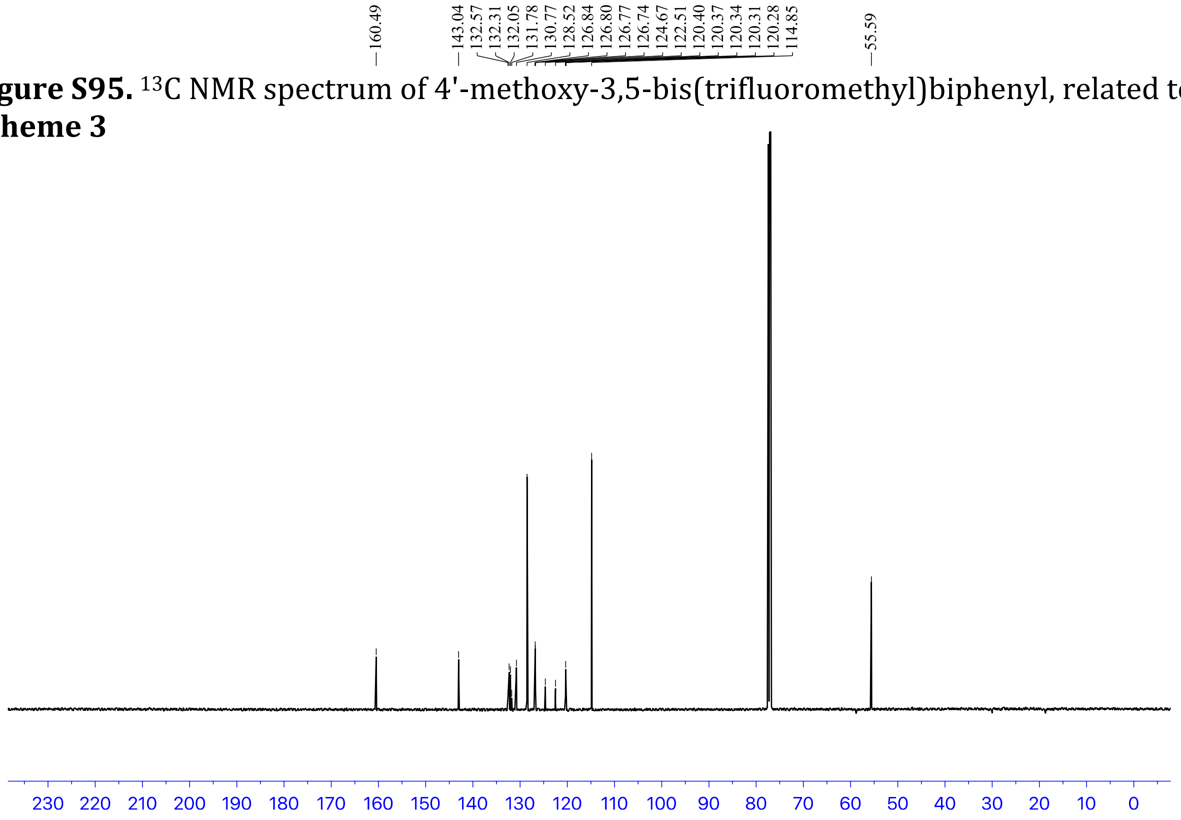

Figure S96. ^{19}F NMR spectrum of 4'-Methoxy-3,5-bis(trifluoromethyl)biphenyl, related to **Scheme 3**

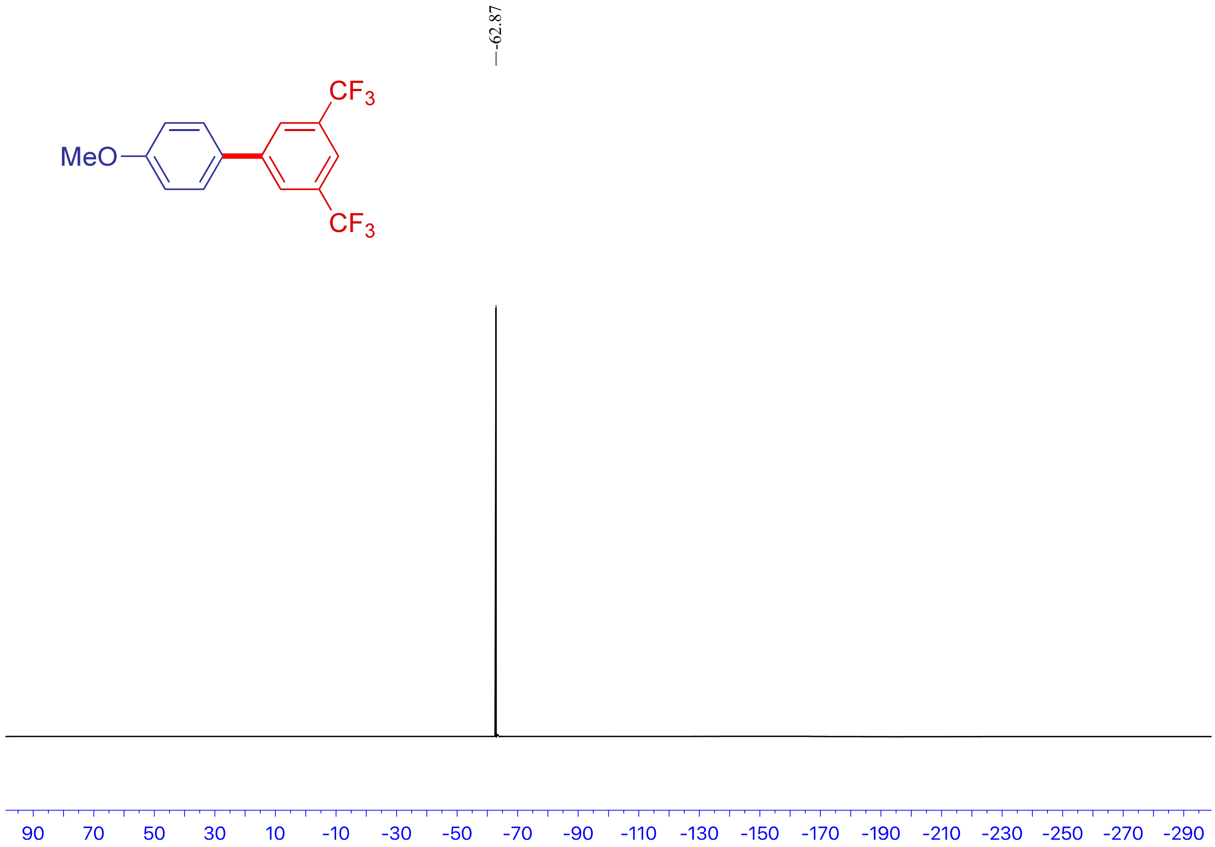

Figure S97. ^{1}H NMR spectrum of *tert*-butyl 2-(4,6-dimethoxypyrimidin-2-yl)-1*H*-pyrrole-1-carboxylate, related to **Scheme 3**

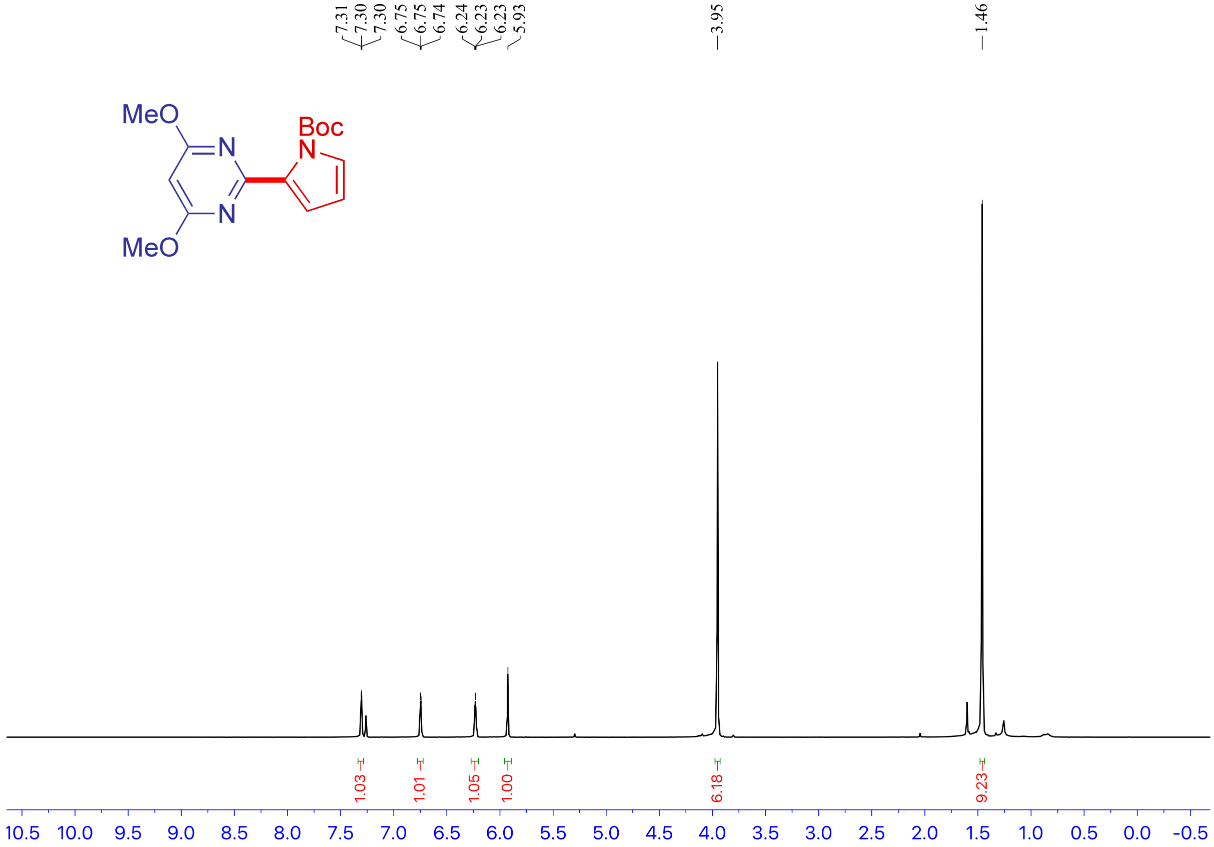

Figure S98. ^{13}C NMR spectrum of *tert*-butyl 2-(4,6-dimethoxypyrimidin-2-yl)-1*H*-pyrrole-1-carboxylate, related to **Scheme 3**

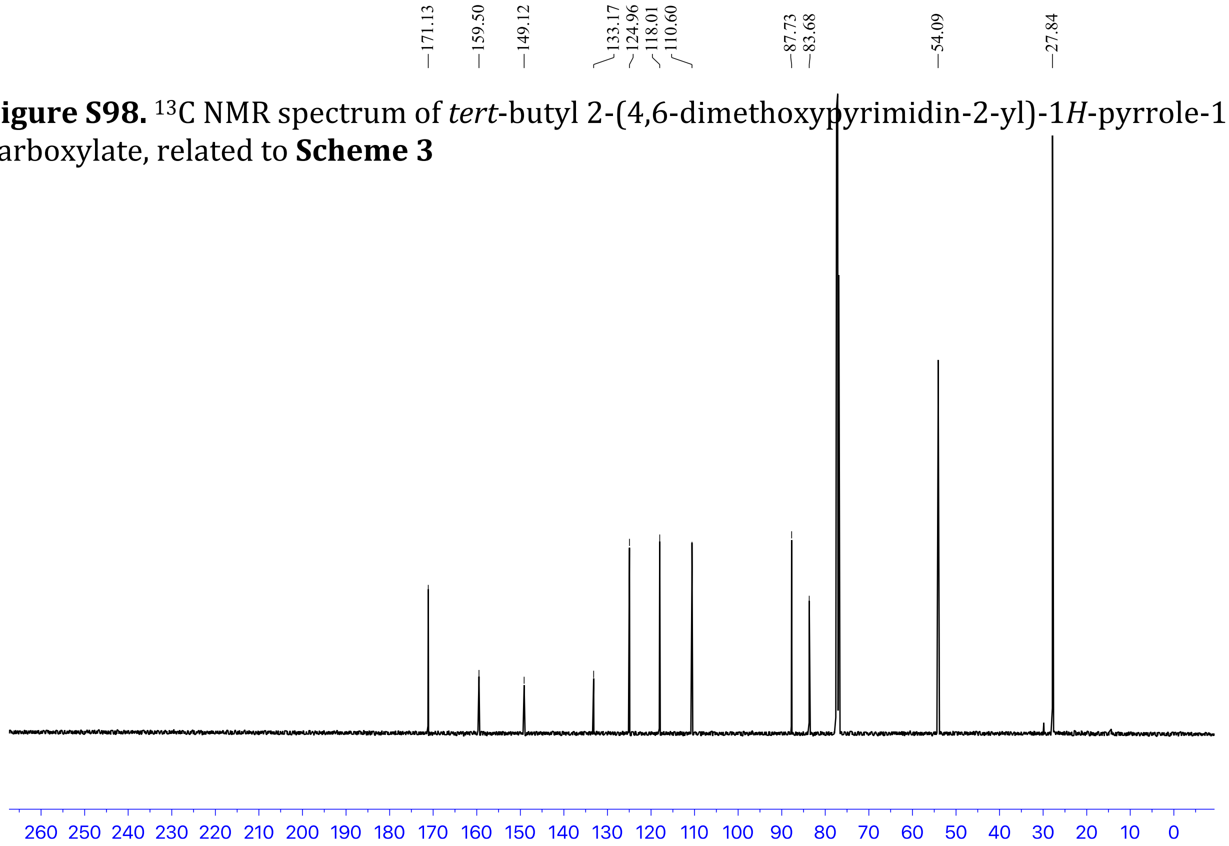

Figure S99. ^{1}H NMR spectrum of isopropyl 2-(4-([1,1'-biphenyl]-4-carbonyl)phenoxy)-2-methylpropanoate, related to **Scheme 4**

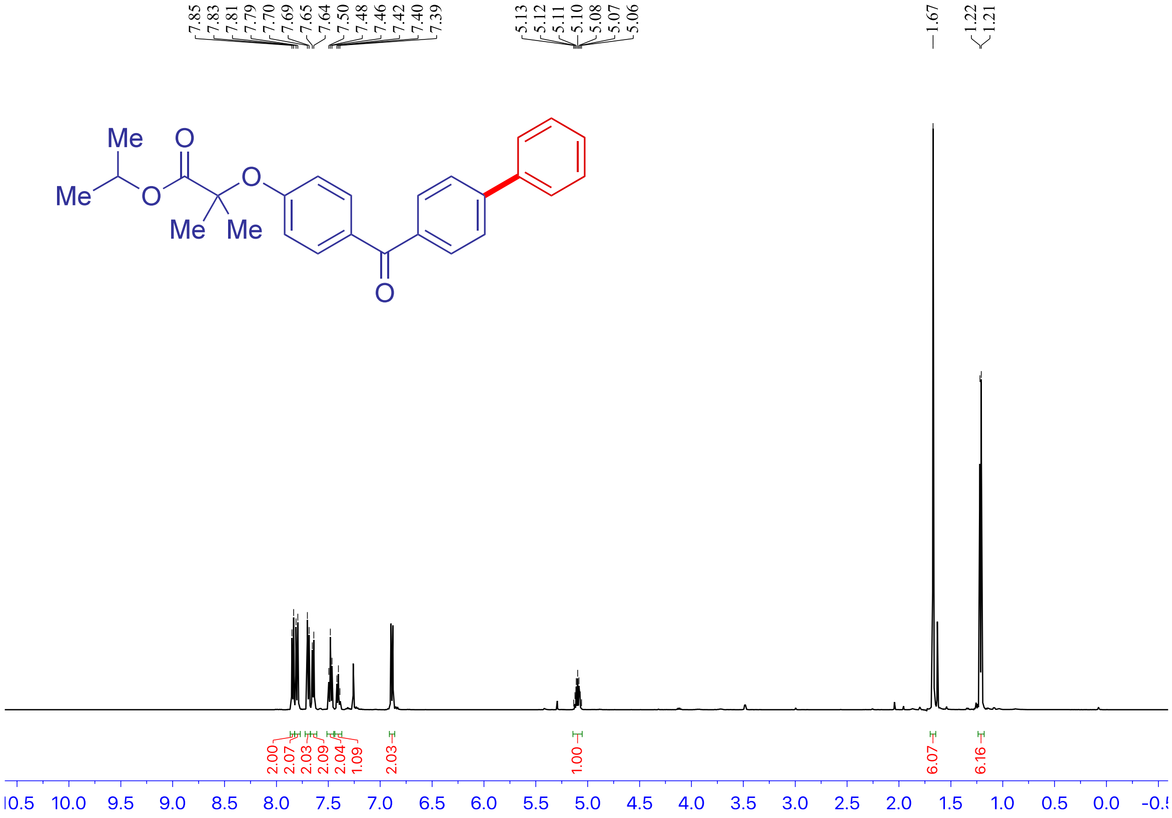

Figure S100. ^{13}C NMR spectrum of isopropyl 2-(4-([1,1'-biphenyl]-4-carbonyl)phenoxy)-2-methylpropanoate, related to **Scheme 4**

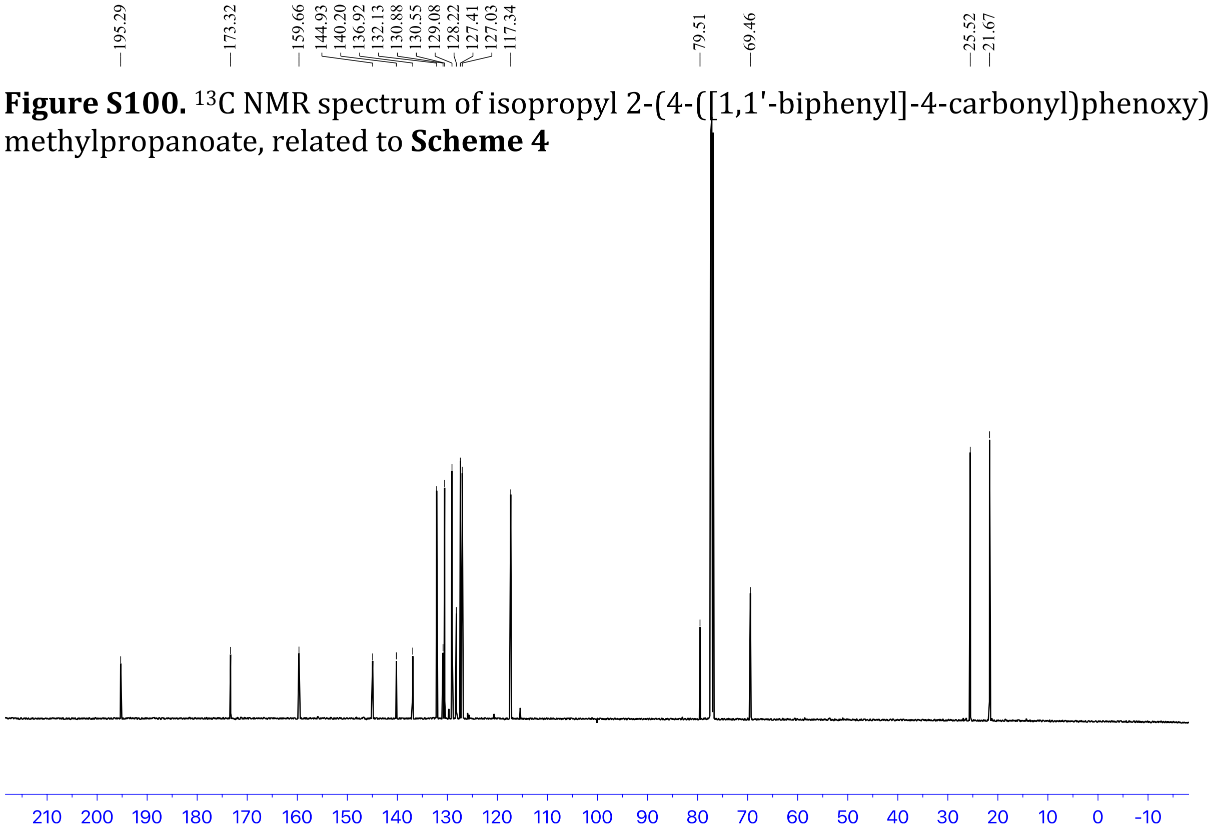

Figure S101. ^{1}H NMR spectrum of 1-(4-fluorophenyl)-4-(4-hydroxy-4-(4'-methoxy-[1,1'-biphenyl]-4-yl)piperidin-1-yl)butan-1-one, related to **Scheme 4**

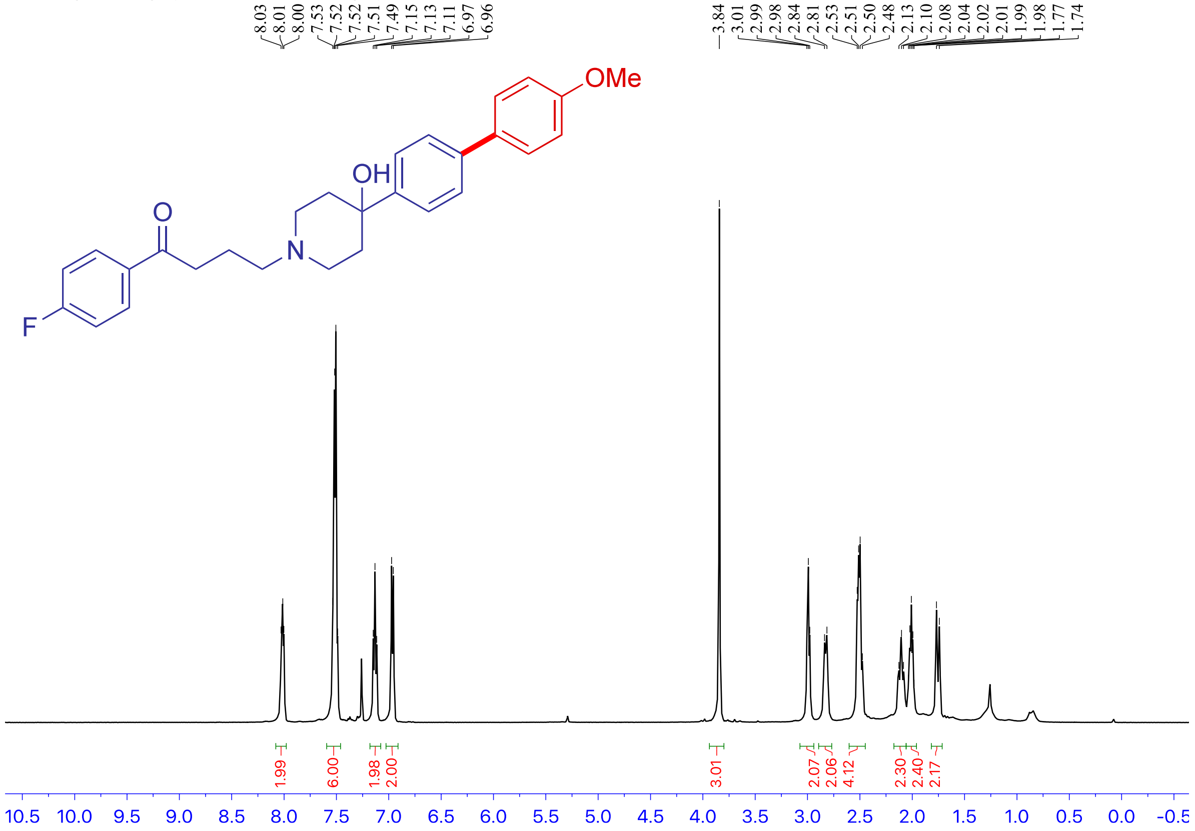

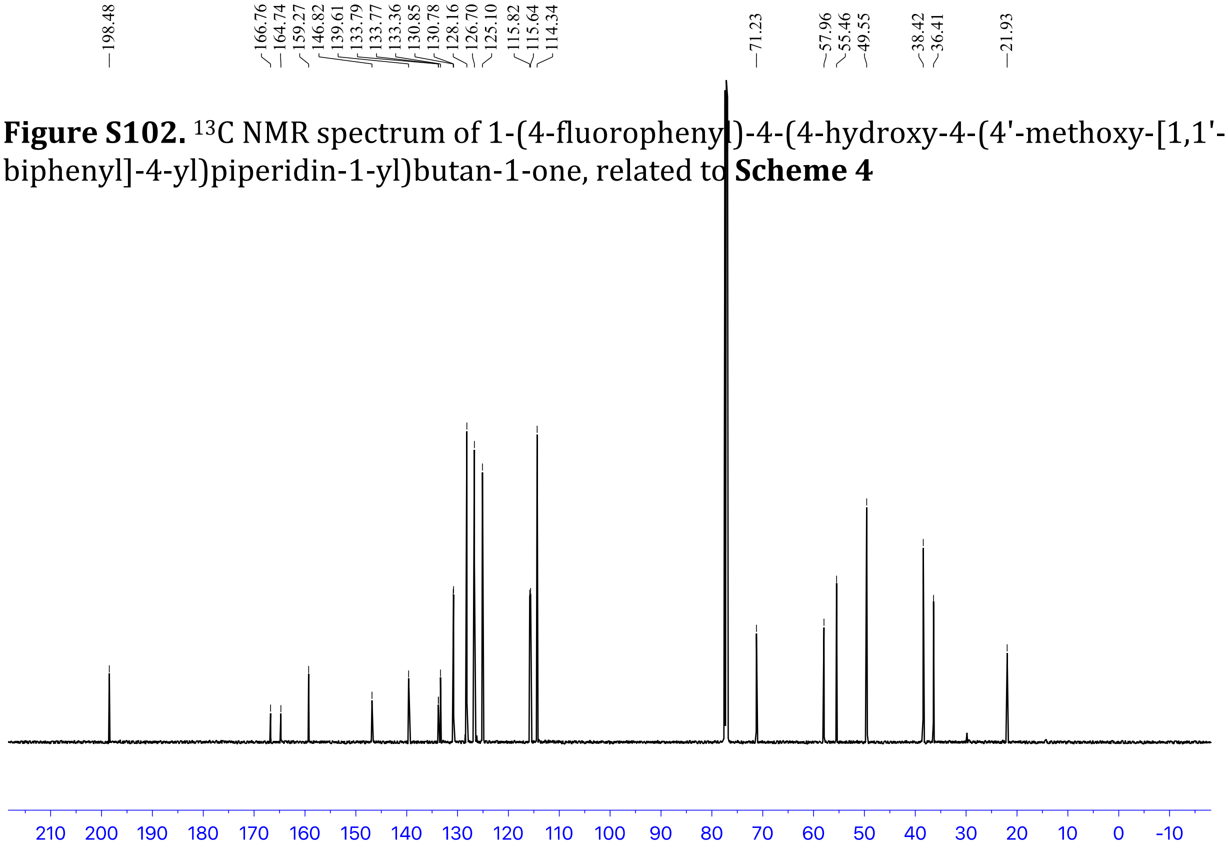

Figure S102. ^{13}C NMR spectrum of 1-(4-fluorophenyl)-4-(4-hydroxy-4-(4'-methoxy-[1,1'-biphenyl]-4-yl)piperidin-1-yl)butan-1-one, related to **Scheme 4**

Figure S103. ^{19}F NMR spectrum of 1-(4-fluorophenyl)-4-(4-hydroxy-4-(4'-methoxy-[1,1'-biphenyl]-4-yl)piperidin-1-yl)butan-1-one, related to **Scheme 4**

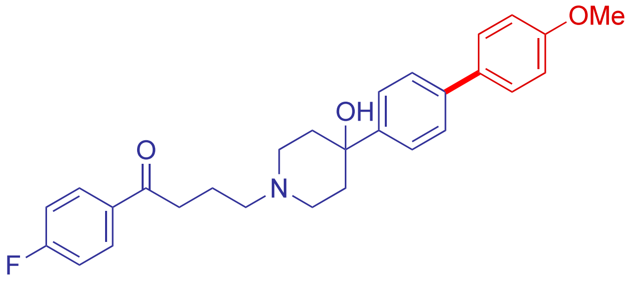

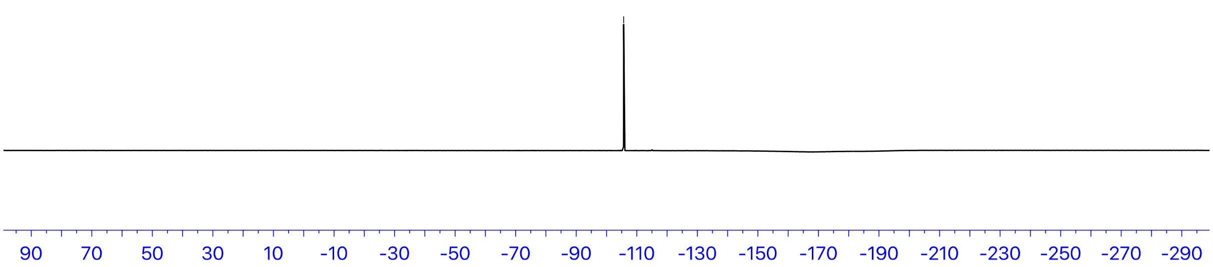

Figure S104. ^{1}H NMR spectrum of 2-(5-methoxy-2-methyl-1-(4'-methyl-[1,1'-biphenyl]-4-carbonyl)-1*H*-indol-3-yl)acetic acid, related to **Scheme 4**

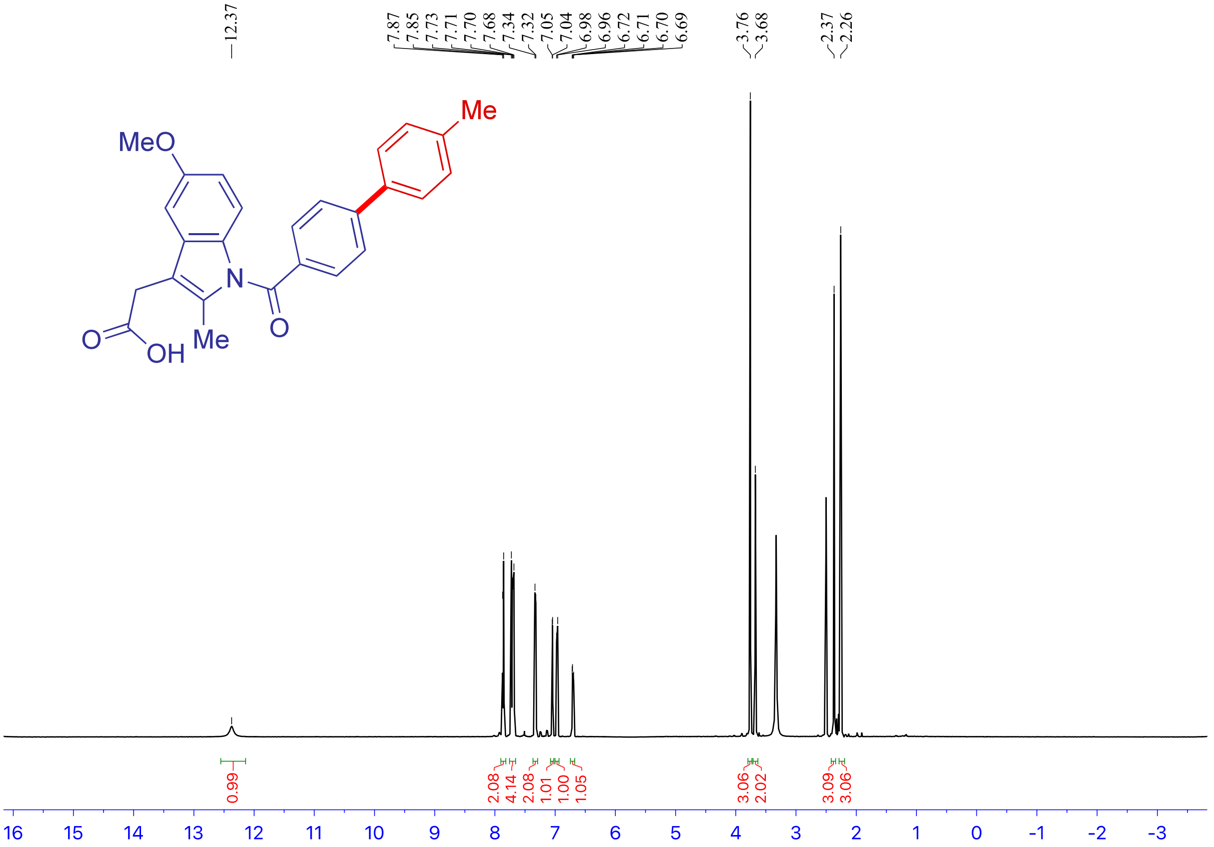

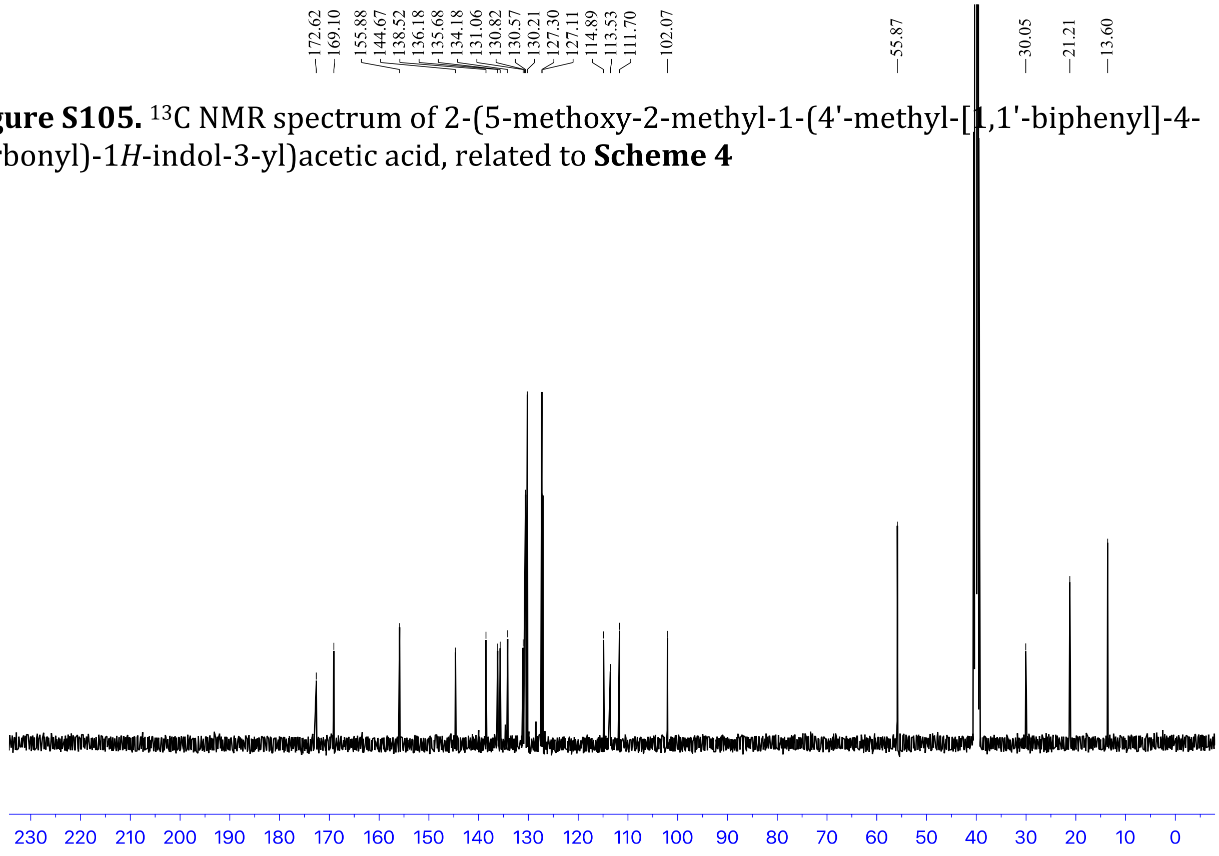

Figure S105. ^{13}C NMR spectrum of 2-(5-methoxy-2-methyl-1-(4'-methyl-[1,1'-biphenyl]-4-carbonyl)-1*H*-indol-3-yl)acetic acid, related to **Scheme 4**

Figure S106. ^{1}H NMR spectrum of *N,N*-dimethyl-3-(2-phenyl-10*H*-phenothiazin-10-yl)propan-1-amine, related to **Scheme 4**

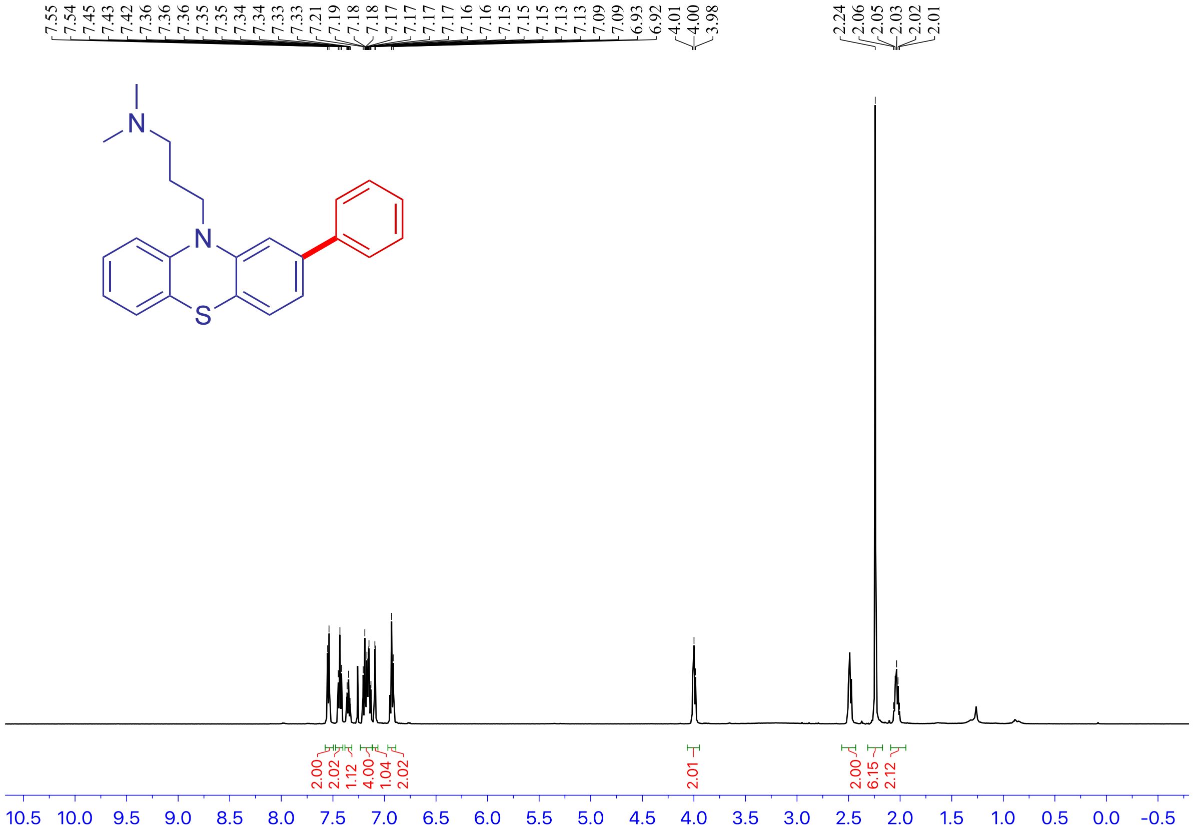

Figure S107. ^{13}C NMR spectrum of *N,N*-dimethyl-3-(2-phenyl-10*H*-phenothiazin-10-yl)propan-1-amine, related to **Scheme 4**

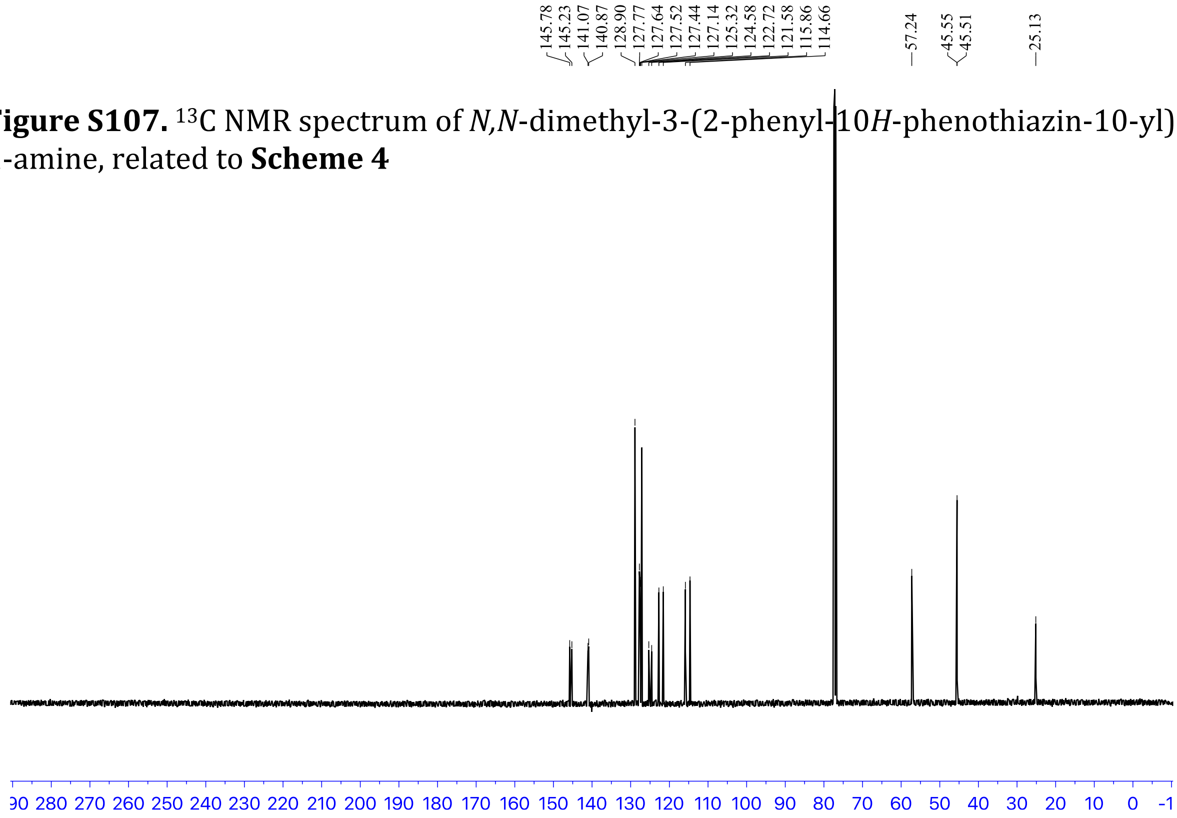

Figure S108. ^{1}H NMR spectrum of *N*-(4-(*N*-(cyclohexylcarbamoyl)sulfamoyl)phenethyl)-4-methoxy-4'-methyl-[1,1'-biphenyl]-3-carboxamide, related to **Scheme 4**

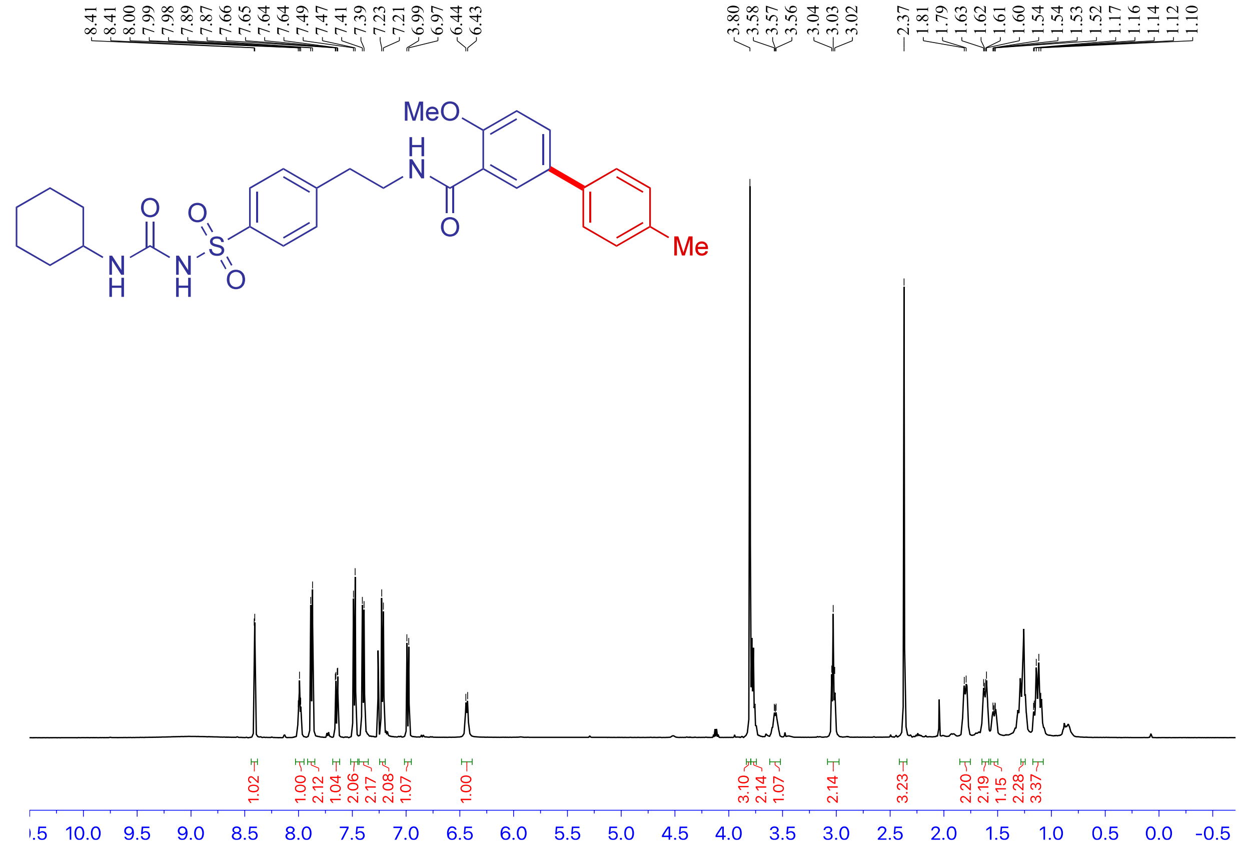

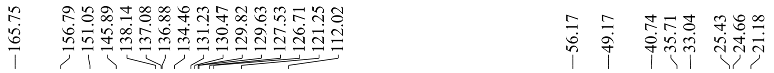

Figure S109. ^{13}C NMR spectrum of *N*-(4-(*N*-(cyclohexylcarbamoyl)sulfamoyl)phenethyl)-4-methoxy-4'-methyl-[1,1'-biphenyl]-3-carboxamide, related to **Scheme 4**

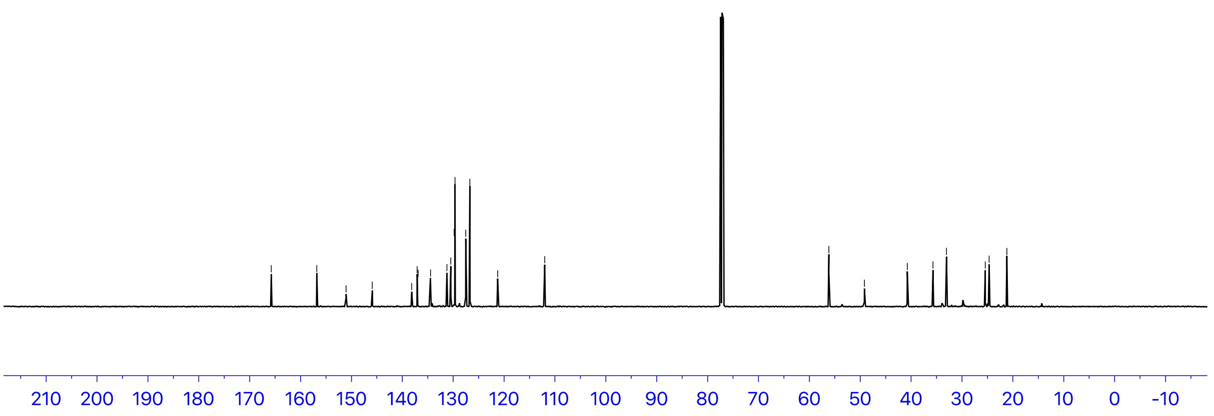

Figure S110. ^{1}H NMR spectrum of (2*S*,6'*R*)-2',4,6-trimethoxy-6'-methyl-7-(*p*-tolyl)-3*H*-spiro[benzofuran-2,1'-cyclohexan]-2'-ene-3,4'-dione, related to **Scheme 4**

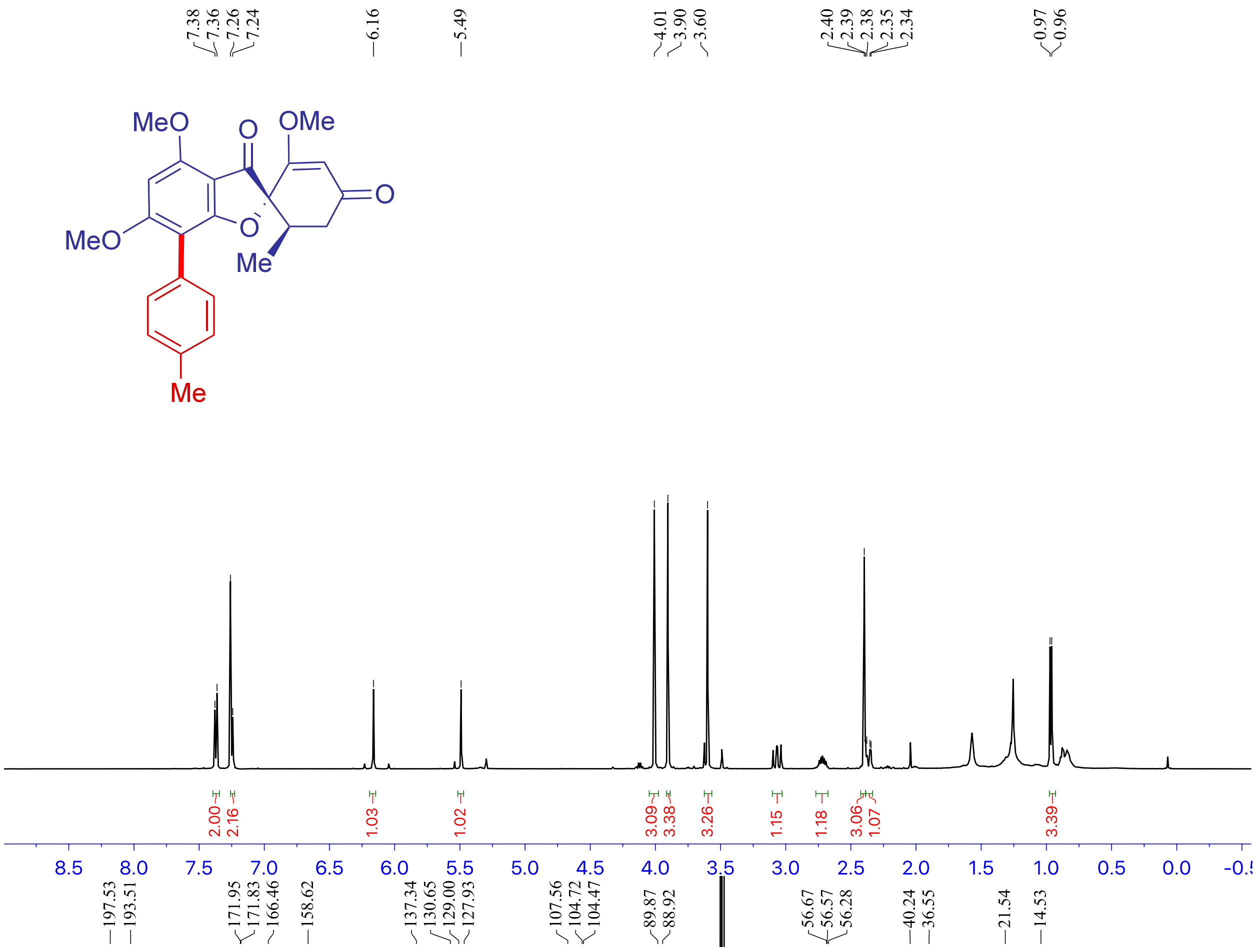

Figure S111. ^{13}C NMR spectrum of (2*S*,6'*R*)-2',4,6-trimethoxy-6'-methyl-7-(*p*-tolyl)-3*H*-spiro[benzofuran-2,1'-cyclohexan]-2'-ene-3,4'-dione, related to **Scheme 4**

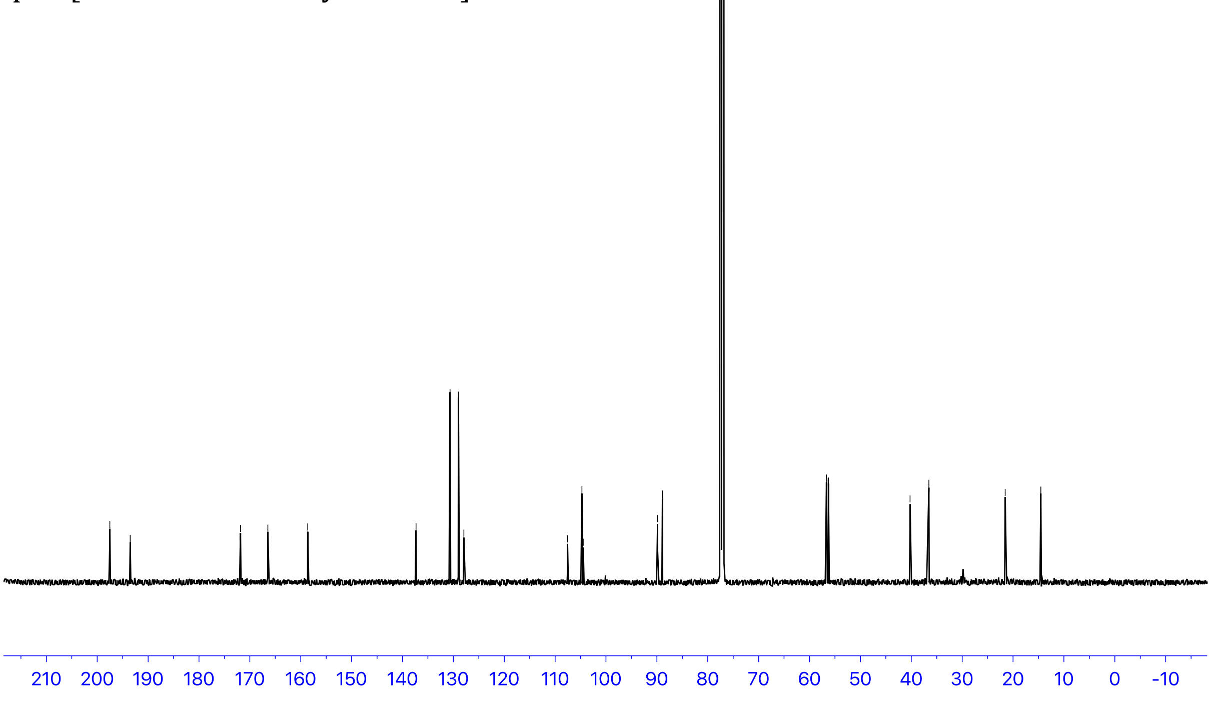

Figure S112. ^{1}H NMR spectrum of *N*^{4}-(7-(3,4-dimethoxyphenyl)quinolin-4-yl)-*N*^{1},*N*^{1}-diethylpentane-1,4-diamine, related to **Scheme 4**

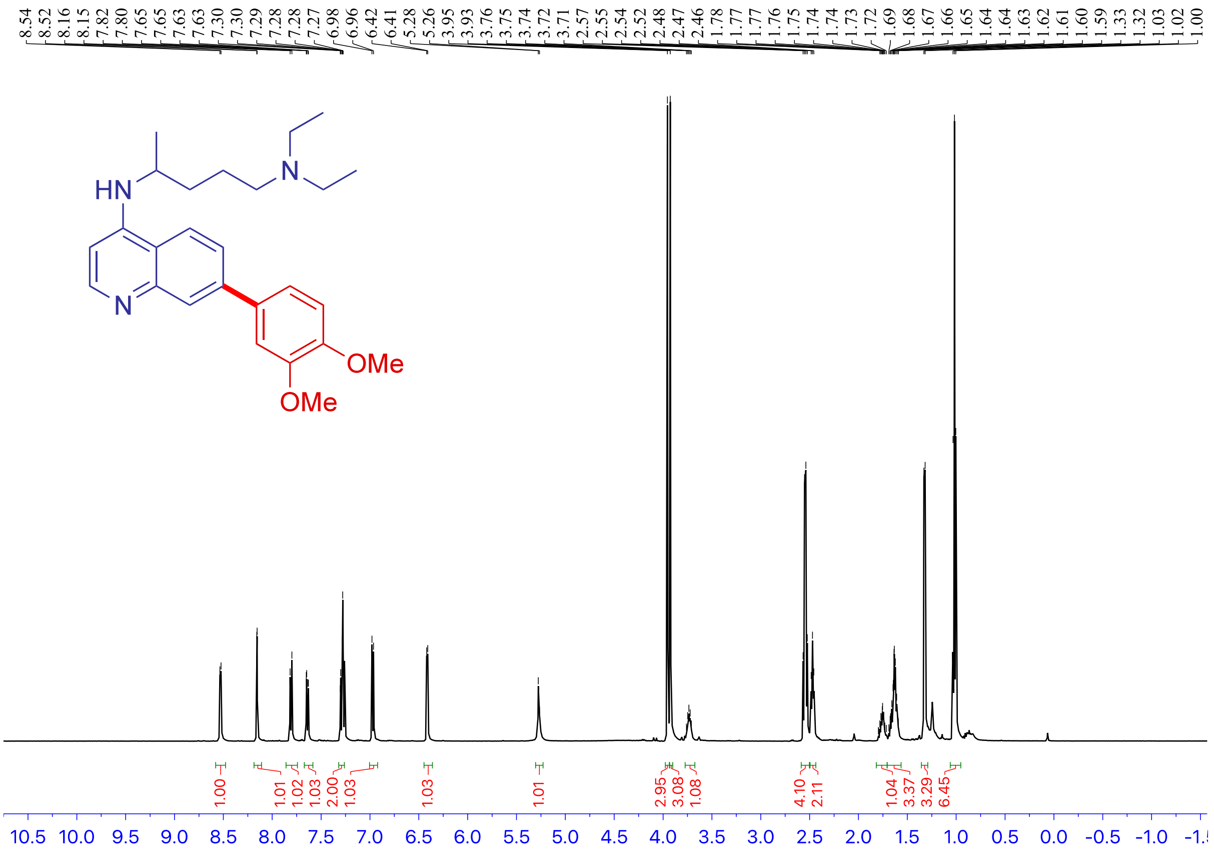

Figure S113. ^{13}C NMR spectrum of *N*^{4}-(7-(3,4-dimethoxyphenyl)quinolin-4-yl)-*N*^{1},*N*^{1}-diethylpentane-1,4-diamine, related to **Scheme 4**

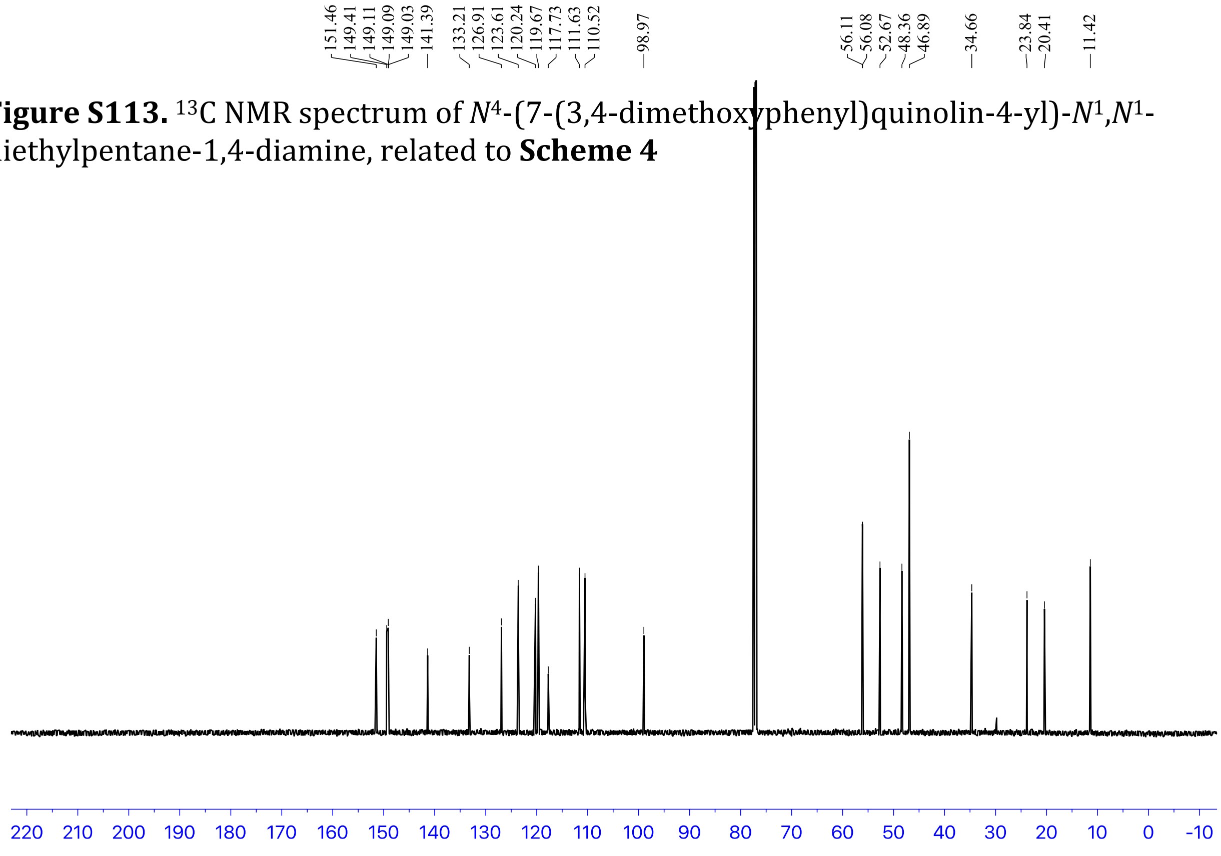

Scheme S1. Comparison of IPr, SIPr and IPr*, Related to **Table 1**.

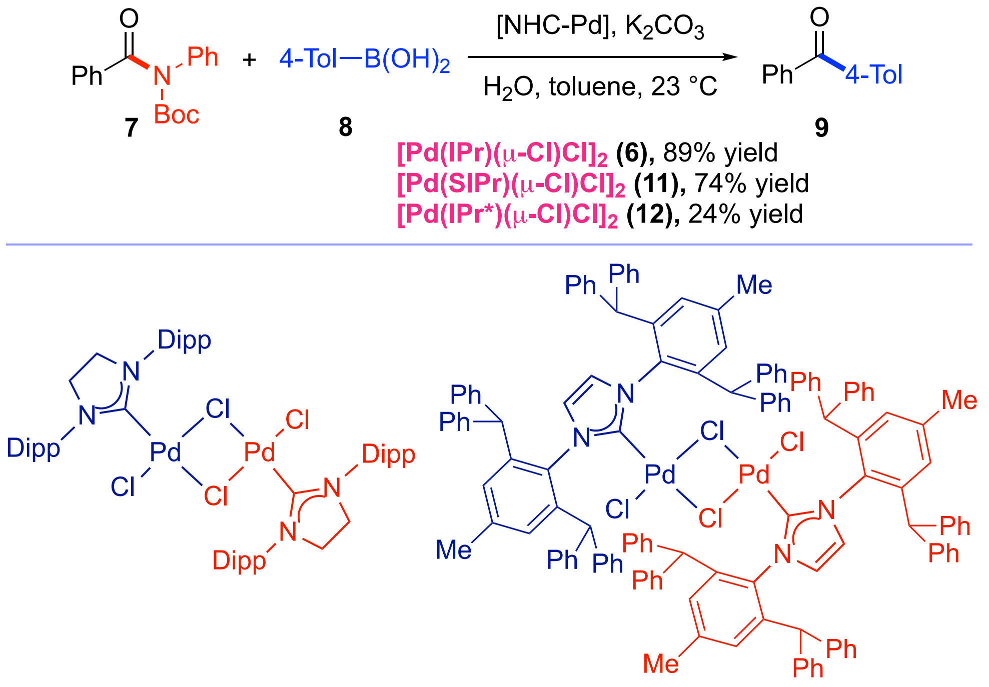

11, **[Pd(SIPr)(μ-Cl)Cl]$_2$** **12**, **[Pd(IPr*)(μ-Cl)Cl]$_2$**

Conditions: amide (1.0 equiv), 4-Tol-B(OH)$_2$ (2.0 equiv), catalyst ([Pd(NHC)(μ-Cl)Cl]$_2$, 0.05 mol%), K_2CO_3 (3.0 equiv), H_2O (5 equiv), toluene (0.50 M), 23 °C, 16 h.

Scheme S2. Determination of Relative Reaction Rates in the Suzuki-Miyaura Cross-Coupling Catalyzed by [Pd-NHC], Related to **Figure 2**.

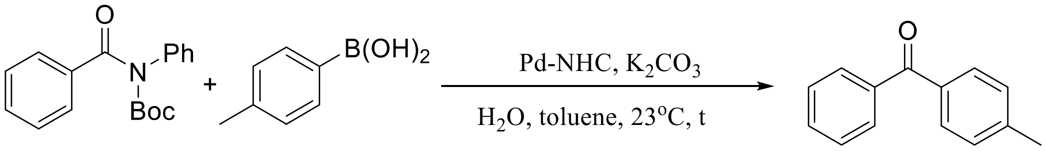

Conditions: amide (1.0 equiv), boronic acid (2 equiv), K_2CO_3 (3 equiv), H_2O (5.0 equiv), [(IPr)Pd(μ-Cl)Cl]$_2$ (0.05 mol%), for other catalysts (0.1 mol%), toluene (0.5 M).

t/h	[(IPr)Pd(cin)Cl]	[(IPr)Pd(1-*t*Bu-ind)Cl]	Pd-PEPPSI-IPr	[(IPr)Pd(μ-Cl)Cl]$_2$
0.167	0	0	0	4
0.5	0	0	0	9
1	1	2	1	35
2	21	9	9	60
4	42	25	21	89
6	62	48	29	94
8	76	67	36	97
12	77	90	48	99
20	80	92	59	100

Scheme S3. Determination of Turnover number (TON) in the Suzuki-Miyaura Cross-Coupling Catalyzed by $[(IPr)Pd(\mu\text{-}Cl)Cl]_2$, Related to **Scheme 1**.

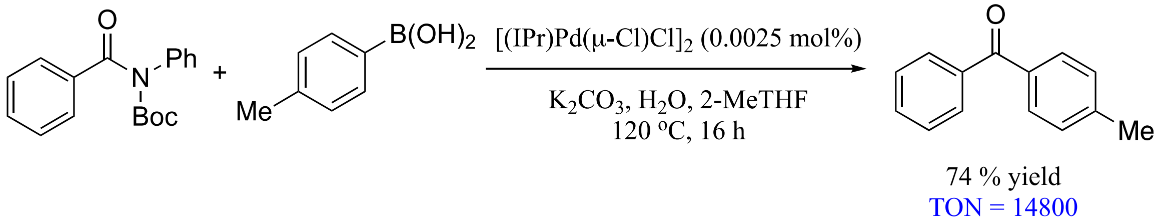

Scheme S4. Determination of Relative Reaction Rates in the Suzuki-Miyaura Cross-Coupling Catalyzed by [Pd-NHC], Related to **Figure 3**.

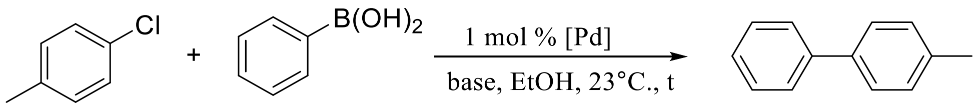

Conditions: ArCl (0.2 mmol), boronic acid (1.05 equiv), KO*t*Bu (1.1 equiv) or K_2CO_3 (2.2 equiv), [(IPr)Pd(μ-Cl)Cl]$_2$ (0.5 mol%), (IPr)Pd(1-*t*Bu-ind)Cl (1 mol%), EtOH (0.5 M), 23 °C.

t/min	[(IPr)Pd(μ-Cl)Cl]$_2$		[(IPr)Pd(1-*t*Bu-ind)Cl]	
	KO*t*Bu	K_2CO_3	KO*t*Bu	K_2CO_3
5	0	2	6	0
10	24	28	5	4
15	79	67	81	43
30	83	82	83	60
60	85	91	84	68

Scheme S5. Suzuki-Miyaura Cross-Coupling of 4-Chloroanisole, Related to **Figure 3**.

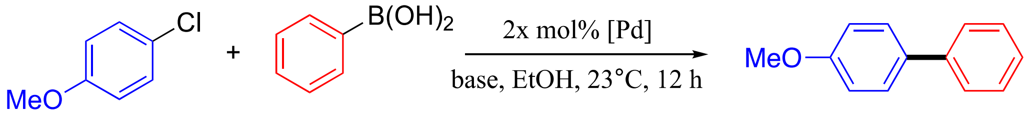

Conditions: ArCl (0.2 mmol), boronic acid (1.05 equiv), KO*t*Bu (1.1 equiv) or K_2CO_3 (2.2 equiv), [(IPr)Pd(μ-Cl)Cl]$_2$ (x mol %), [(IPr)Pd(1-*t*Bu-ind)Cl] (2x mol%), EtOH (0.5 M), 23 °C, 12 h; GC/^{1}H NMR yields.

**Entry**	**Base**	**x**	**Yield %**	
			[(IPr)Pd(μ-Cl)Cl]$_2$	[(IPr)Pd(1-*t*Bu-ind)Cl]
1		0.5	93	82
2	KO*t*Bu	0.1	94	78
3		0.05	68	65
4		0.5	99	95
5	K_2CO_3	0.1	99	95
6		0.05	83	81

Scheme S6. Suzuki-Miyaura Cross-Coupling of 2-Chloro-*m*-xylene, Related to **Figure 3**.

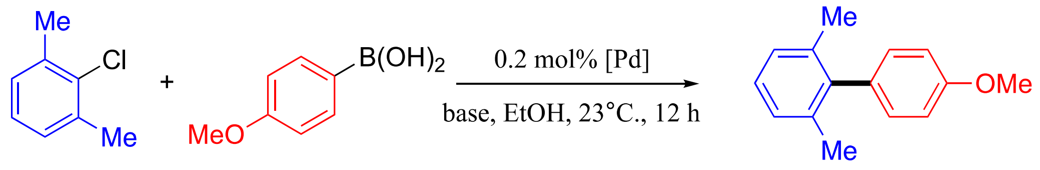

Conditions: ArCl (0.2 mmol), boronic acid (1.05 equiv), KO*t*Bu (1.1 equiv) or K_2CO_3 (2.2 equiv), $[(IPr)Pd(\mu\text{-}Cl)Cl]_2$ (0.1 mol %)/[(IPr)Pd(1-*t*Bu-ind)Cl] (0.2 mol%), EtOH (0.5 M), 23 °C, 12 h; GC/^{1}H NMR yields.

Entry	Base	Yield %	
		$[(IPr)Pd(\mu\text{-}Cl)Cl]_2$	[(IPr)Pd(1-*t*Bu-ind)Cl]
1	KO*t*Bu	76	63
2	K_2CO_3	99	99

Scheme S7. Suzuki-Miyaura of Aryl Chlorides at 50 ppm Pd Loading, Related to **Figure 3**.

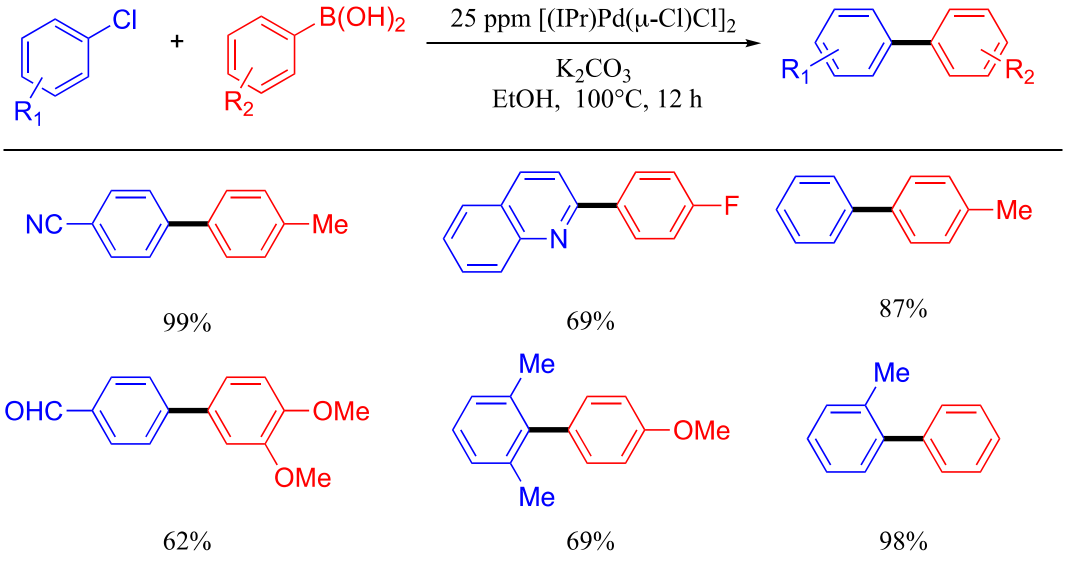

Conditions: ArCl (0.2 mmol), boronic acid (2 equiv), K_2CO_3 (3 equiv), $[(IPr)Pd(\mu\text{-}Cl)Cl]_2$ (0.0025 mol %), EtOH (0.5 M), 12 h. GC/^{1}H NMR yields.

Scheme S8. Plot of ln([Prod]end-[Prod]) versus time for a representative reaction involving catalyst, KOtBu and dvds in d_4-MeOH, Related to **Figure 3**.

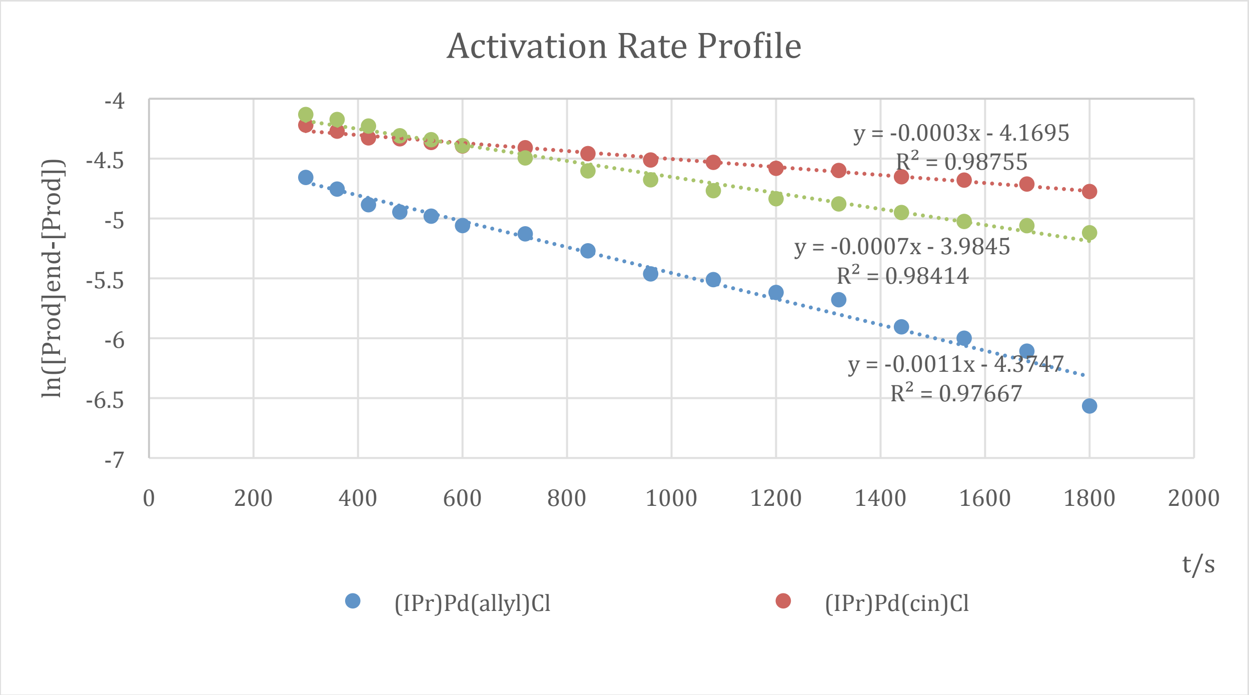

Table S1. Summary of Optimization of Pd-Catalyzed Biaryl Suzuki-Miyaura Cross-Coupling, Related to **Figure 3**.

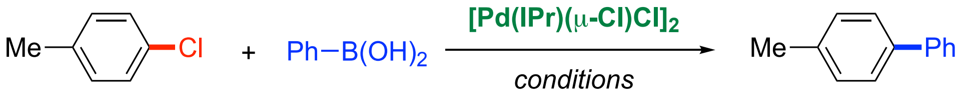

entry	solvent	base	base (equiv)	T (°C)	yield[b] (%)
1	*i*-PrOH	KO*t*-Bu	1.1	23	32
2	EtOH	KO*t*-Bu	2.2	23	>98
3	EtOH	KO*t*-Bu	1.1	23	>98
4	dioxane	KO*t*-Bu	1.5	80	76
5	dioxane	Cs_2CO_3	1.5	80	63
6	DME	Cs_2CO_3	1.5	80	50
7	EtOH	K_2CO_3	2.2	23	>98
8	EtOH	K_2CO_3	1.5	23	93
9	EtOH	K_2CO_3	1.1	23	78
10	MeOH	KO*t*-Bu	1.1	23	91

Conditions: ArCl (1.0 equiv), catalyst (0.50 mol%), 4-Tol-$B(OH)_2$ (1.05), base (1.1-2.2 equiv), solvent (0.50 M), 23-80 °C, 12 h. [b]GC/^{1}H NMR yields.

Table S2. Pd-Catalyzed Biaryl Suzuki-Miyaura Cross-Coupling with Different [Pd(NHC)(μ-X)X]$_2$ Catalysts, Related to **Figure 3**.

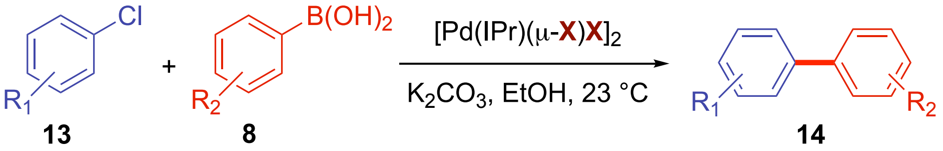

	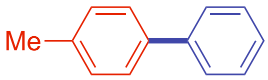	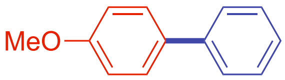	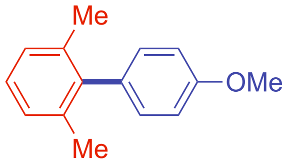
	yield (%)	yield (%)	yield (%)
X = Cl	>98	>98	>98
X = Br	85	87	82
X = I	<5	<5	<5
X = I	42[b]	33[b]	96[b]

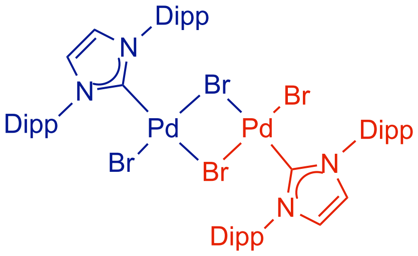

15, **[Pd(IPr)(μ-Br)Br]$_2$**

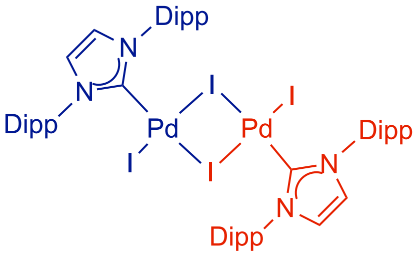

16, **[Pd(IPr)(μ-I)I]$_2$**

Conditions: ArCl (1.0 equiv), catalyst (0.05 mol%), Ar-B(OH)$_2$ (1.05), K_2CO_3 (2.2 equiv), EtOH (0.50 M), 23 °C, 12 h. [b]60 °C. Catalysts: X = Cl: [Pd(IPr)(μ-Cl)Cl]$_2$ (**6**); X = Br: [Pd(IPr)(μ-Br)Br]$_2$ (**15**); X = I: [Pd(IPr)(μ-I)I]$_2$ (**16**).

Transparent Methods

Computational Details

All DFT static calculations were performed at the GGA level with the Gaussian 09 set of programs (Frisch et al, 2016) using the BP86 functional of Becke and Perdew (Becke, 1988; Perdew, 1986; Perdew, 1986). The electronic configuration of the molecular systems was described with the standard split valence basis set with a polarization function of Ahlrichs and co-workers for H, C, B, N, O and Cl (SVP keyword in Gaussian) (Schafer et al., 1994) and Def2-QZVPP for K (Weigend, 2006). For Pd we used the quasi-relativistic Stuttgart/Dresden effective core potential (Kechle et al., 1994; Leininger et al., 1996) with an associated valence basis set (standard SDD keywords in Gaussian 09). Geometry optimizations were performed without symmetry constraints, and the characterization of the stationary points was performed by analytical frequency calculations. These frequencies were used to calculate unscaled zero-point energies (ZPEs) as well as thermal corrections and entropy effects at 298 K and 1 atm by using the standard statistical mechanics relationships for an ideal gas. Moreover, we also included the D3 Grimme pairwise scheme to account for dispersion corrections in the geometry optimizations (Grimme et al., 2010). Energies were obtained via single-point calculations on the BP86-optimized geometries using the M06 functional (Zhao et al., 2008). In these single-point energy calculations, H, C, B, N, O and Cl were described by using the Def2-TZVP basis set that includes polarization functions (Weigend, et al., 2005), Def2-QZVPP for K, whereas for the metal (Pd), the SDD basis set has been employed. On top of the M06/Def2-TZVP~sdd//BP86-D3/SVP~sdd energies, we added the ZPEs thermal and entropy corrections obtained at the BP86-D3/SVP~sdd level. In addition, to calculate the reported Gibbs energies, we included solvent effects of THF solution estimated with the polarizable continuous solvation model (PCM) as implemented in Gaussian 16 (Barone et al., 1998; Tomasi et al., 1994).

One-Step Synthesis of $[Pd(IPr)(m\text{-}Cl)Cl]_2$

In a glass vial, IPr·HCl (47.3 mg, 1 equiv.), $Pd(OAc)_2$ (30 mg, 1.2 equiv.) and K_2CO_3 (55 mg, 3.5 equiv.) were added, followed by dry toluene (0.5 mL). The reaction was heated at 80 °C overnight. The reaction was then filtered on celite and washed with DCM. 4M HCl in dioxane (0.4 mL) was added to the filtrate solution, and the mixture was stirred for 5 min. The solution was concentrated under vacuum. Pentane was added and the precipitate was filtered to yield 61 mg of a dark yellow powder (81% yield).

Large scale: In a glass vial, IPr·HCl (1.58 g, 1 equiv.), $Pd(OAc)_2$ (1 g, 1.2 equiv.) and K_2CO_3 (2.05 g, 4 equiv.) were added, followed by dry toluene (17 mL). The reaction was heated at 80 °C overnight. The reaction was then filtered on celite and washed with DCM. 4M HCl in dioxane (10 mL) was added to the filtrate solution, and the mixture was stirred for 10 min. The solution was concentrated under vacuum. Pentane was added and the precipitate was filtered to yield 1.69 g of a dark yellow powder (81% yield).

^{1}H NMR (400 MHz, $CDCl_3$) δ 7.54 (t, J = 7.7 Hz, 4H), 7.34-7.29 (m, 8H), 6.98 (s, 4H), 2.86 (br. s, 4H), 2.60 (br. s, 4H), 1.30 (d, J = 39.9 Hz, 24H), 0.99 (d, J = 27.5 Hz, 24H). Elemental analysis: Calcd for $C_{54}H_{72}N_4Cl_4Pd_2$ C: 57.30; H:6.41; N : 4.95. Found: C: 57.40; H: 6.50; N: 5.02.

List of Known Compounds/General Methods

All starting materials reported in the manuscript have been previously described in literature and prepared by the method reported previously unless stated otherwise. Amides were prepared by standard methods. All experiments involving palladium were performed using standard Schlenk techniques under nitrogen or argon unless stated otherwise. All solvents were purchased at the highest commercial grade and used as received or after purification by passing through activated alumina columns or distillation from sodium/benzophenone under nitrogen. All solvents were deoxygenated prior to use. All other chemicals were purchased at the highest commercial grade and used as received. Reaction glassware was oven-dried at 140 °C for at least 24 h or flame-dried prior to use, allowed to cool under vacuum and purged with argon (three cycles). All products were identified using ^{1}H NMR analysis and comparison with authentic samples. GC and/or GC/MS analysis was used for volatile products. All yields refer to yields determined by ^{1}H NMR and/or GC or GC/MS using an internal standard (optimization) and isolated yields (preparative runs) unless stated otherwise.^{1}H NMR and ^{13}C NMR spectra were recorded in $CDCl_3$ or DMSO on Bruker spectrometers at 500 (^{1}H NMR), 125 (^{13}C NMR) and 471 (^{19}F NMR) MHz. All shifts are reported in parts per million (ppm) relative to residual $CHCl_3$ peak (7.26 and 77.16 ppm, ^{1}H NMR and ^{13}C NMR, respectively). All coupling constants (*J*) are reported in hertz (Hz). Abbreviations are: s, singlet; d, doublet; t, triplet; q, quartet; brs, broad singlet. GC-MS chromatography was performed using Agilent HP6890 GC System and Agilent 5973A inert XL EI/CI MSD using helium as the carrier gas at a flow rate of 1 mL/min and an initial oven temperature of 50 °C. The injector temperature was 250 °C. The detector temperature was 250 °C. For runs with the initial oven temperature of 50 °C, temperature was increased with a 10 °C/min ramp after 50 °C hold for 3 min to a final temperature of 220 °C, then hold at 220 °C for 15 min (splitless mode of injection, total run time 22.0 min). High-resolution mass spectra were measured on a 7T Bruker Daltonics FT-MS instrument. All flash chromatography was performed using silica gel, 60 A, 300 mesh. TLC analysis was carried out on glass plates coated with silica gel 60 F254, 0.2 mm thickness. The plates were visualized using a 254 nm ultraviolet lamp or aqueous potassium permanganate. ^{1}H NMR and ^{13}C NMR data are given for all compounds in the SI for characterization purposes. ^{1}H NMR, ^{13}C NMR and HRMS data are given for all new compounds. All products have been previously reported unless stated otherwise.

Experimental Procedures and Characterization Data

General Procedure for the Synthesis of Starting Materials. All amides used in this study have been previously reported and prepared by reported methods (Zhou et al., 2019; Lei et al.; 2017; Liu et al., 2018; Monguchi et al., 2012; Lipshutz et al., 2008). Starting materials were synthesized according to general methods reported in the literature (Al-Huniti et al., 2018; Ackermann et al., 2011; Patel et al., 2012; Sun et al, 2016; Wang et al., 2017; Zhang et al., 2017). Catalysts **[(IPr)Pd(μ-Br)Br]₂** and **[(IPr)Pd(μ-I)I]₂** were prepared according to literature (Deska et al., 2010; Flahaut et al., 2009). ^{1}H NMR and ^{13}C NMR data are given for all starting materials in the section below for characterization purposes.

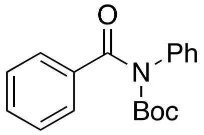

***tert*-Butyl benzoyl(phenyl)carbamate**. White solid. ^{1}H NMR (500 MHz, $CDCl_3$) δ 7.76 (d, *J* = 7.1 Hz, 2 H), 7.55 (t, *J* = 7.4 Hz, 1 H), 7.49-7.43 (m, 4 H), 7.37 (t, *J* = 7.4 Hz, 1 H), 7.30 (d, *J* = 7.4 Hz, 2 H), 1.26 (s, 9 H). ^{13}C NMR (125 MHz, $CDCl_3$) δ 172.78, 153.30, 139.10, 136.98, 131.72, 129.21, 128.28, 128.14, 127.96, 127.80, 83.50, 27.49. (Zhou et al., 2019)

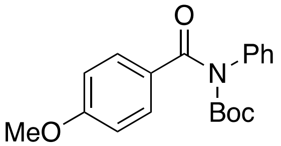

***tert*-Butyl (4-methoxybenzoyl)(phenyl)carbamate**. White solid. ^{1}H NMR (500 MHz, $CDCl_3$) δ 7.77 (d, *J* = 7.6 Hz, 2 H), 7.43 (t, *J* = 7.2 Hz, 2 H), 7.33 (t, *J* = 7.3 Hz, 1 H), 7.28 (d, *J* = 7.9 Hz, 2 H), 6.95 (d, *J* = 7.7 Hz, 2H), 3.88 (s, 3 H), 1.32 (s, 9 H). ^{13}C NMR (125 MHz, $CDCl_3$) δ 172.07, 162.78, 153.53, 139.48, 130.86, 129.12, 128.72, 127.75, 127.48, 113.56, 83.10, 55.49, 27.65. (Zhou et al., 2019)

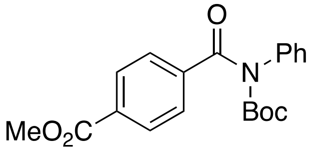

***tert*-Butyl phenyl((4-(methoxycarbonyl)benzoyl)carbamate.** White solid. ^{1}H NMR (500 MHz, $CDCl_3$) δ 8.14 (d, *J* = 8.1 Hz, 2 H), 7.78 (d, *J* = 8.1 Hz, 2 H), 7.46 (t, *J* = 7.6 Hz, 2 H), 7.38 (t, *J* = 7.3 Hz, 1 H), 7.28 (d, *J* = 7.8 Hz, 2 H), 3.97 (s, 3 H), 1.26 (s, 9 H). ^{13}C NMR (125 MHz, $CDCl_3$) δ 171.83, 166.21, 152.93, 141.01, 138.62, 132.53, 129.52, 129.27, 128.07, 127.99, 127.78, 84.01, 52.42, 27.51. (Zhou et al., 2019)

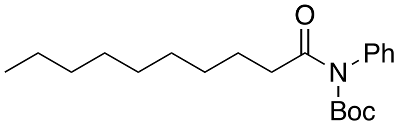

***tert*-Butyl decanoyl(phenyl)carbamate.** White solid. ^{1}H NMR (500 MHz, $CDCl_3$) δ 7.41(t, *J*=7.2 Hz, 2H), 7.34(t, *J*=7.3 Hz,1H), 7.09(d, *J*=7.7 Hz,2H), 2.92 (t, *J* = 7.4 Hz, 2 H), 1.70 (p, *J* = 7.3, 6.8 Hz, 2 H), 1.40 (s, 9 H), 1.29 (s, 12 H), 0.90 (t, *J* = 6.4 Hz, 3 H). ^{13}C NMR (125 MHz, $CDCl_3$) δ 176.0, 152.3, 139.2, 128.9, 128.2, 127.7, 82.9, 38.0, 31.9, 29.5, 29.3, 29.2, 27.8, 25.0, 22.7, 14.1. (Zhou et al., 2019)

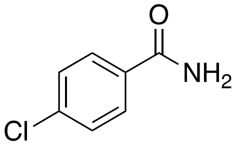

4-Chlorobenzamide. White solid. ^{1}H NMR (500 MHz, $CDCl_3$) δ 7.76 (d, *J* = 8.6 Hz, 2H), 7.43 (d, *J* = 8.5 Hz, 2H), 5.92 (brs, 2H). ^{13}C NMR (125 MHz, $CDCl_3$) δ 168.32, 138.51, 131.84, 129.07, 128.94. (Al-Huniti et al., 2018)

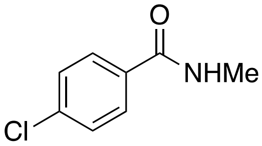

4-Chloro-*N*-methylbenzamide. White solid. ^{1}H NMR (500 MHz, $CDCl_3$) δ 7.70 (d, *J* = 8.1 Hz, 2H), 7.39 (d, *J* = 8.5 Hz, 2H), 6.24 (brs, 1H), 3.00 (d, *J* = 4.9 Hz, 3H). ^{13}C NMR (125 MHz, $CDCl_3$) δ 167.38, 137.75, 133.07, 128.95, 128.42, 27.06. (Ackermann et al., 2011)

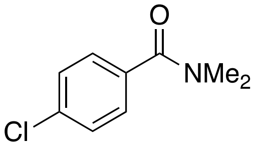

4-Chloro-*N,N*-dimethylbenzamide. White solid. ^{1}H NMR (500 MHz, $CDCl_3$) δ 7.36 (s, 4H), 3.09 (s, 3H), 2.96 (s, 3H). ^{13}C NMR (125 MHz, $CDCl_3$) δ 170.68, 135.70, 134.67, 131.44, 128.72, 39.68, 35.56. (Patel et al., 2012).

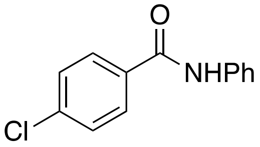

4-Chloro-*N*-phenylbenzamide. White solid. ^{1}H NMR (500 MHz, DMSO-d_6) δ 10.31 (s, 1H), 7.99 (d, *J* = 8.5 Hz, 2H), 7.77 (d, *J* = 7.6 Hz, 2H), 7.61 (d, *J* = 8.5 Hz, 2H), 7.36 (t, *J* = 7.9 Hz, 2H), 7.11 (t, *J* = 7.3 Hz, 1H). ^{13}C NMR (126 MHz, DMSO-d_6) δ 164.39, 138.94, 136.35, 133.63, 129.59, 128.59, 128.42, 123.79, 120.40. (Sun et al., 2016).

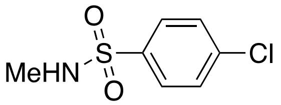

4-Chloro-*N*-methylbenzenesulfonamide. White solid. ^{1}H NMR (500 MHz, $CDCl_3$) δ 7.80 (d, *J* = 8.7 Hz, 2H), 7.50 (d, *J* = 8.6 Hz, 2H), 4.51 (d, *J* = 5.7 Hz, 1H), 2.67 (d, *J* = 5.4 Hz, 3H). ^{13}C NMR (125 MHz, $CDCl_3$) δ 139.42, 137.55, 129.58, 128.83, 29.45. (Wang et al., 2017)

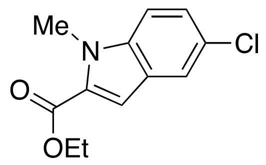

Ethyl 5-chloro-1-methyl-1*H*-indole-2-carboxylate. White solid. ^{1}H NMR (500 MHz, $CDCl_3$) δ 7.65 – 7.60 (m, 1H), 7.30 – 7.28 (m, 2H), 7.21 (s, 1H), 4.38 (q, *J* = 7.1 Hz, 2H), 4.06 (s, 4H), 1.41 (t, *J* = 7.1 Hz, 3H). ^{13}C NMR (125 MHz, $CDCl_3$) δ 162.05, 138.03, 129.30, 126.77, 126.30, 125.45, 121.72, 111.50, 109.41, 60.88, 31.96, 14.49. (Zhang et al., 2017)

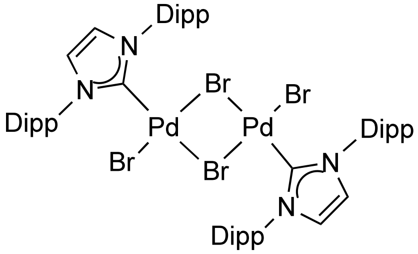

[(IPr)Pd(μ-Br)Br]$_2$. Brown solid. ^{1}H NMR (500 MHz, $CDCl_3$) δ 7.54 (t, *J* = 7.7 Hz, 4H), 7.34 (d, *J* = 7.7 Hz, 4H), 7.28 – 7.26 (m, 4H), 7.01 (s, 4H), 3.11 – 2.95 (m, 4H), 2.71 – 2.58 (m, 4H), 1.41 (d, *J* = 6.5 Hz, 12H), 1.23 (d, *J* = 7.8 Hz, 12H), 1.05 (d, *J* = 6.8 Hz, 12H), 0.94 (d, *J* = 6.8 Hz, 12H). ^{13}C NMR (126 MHz, $CDCl_3$) δ 153.13, 146.72, 146.31, 134.79, 130.49, 125.59, 124.52, 124.41, 28.96, 26.55, 26.52, 23.67, 23.62. (Deska et al., 2010)

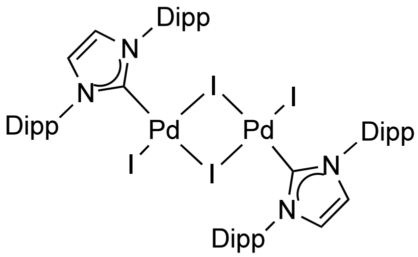

[(IPr)Pd(μ-I)I]$_2$. Red solid. ^{1}H NMR (500 MHz, $CDCl_3$) δ 7.51 (t, *J* = 7.8 Hz, 4H), 7.34 (d, *J* = 7.4 Hz, 4H), 7.27 – 7.22 (m, 4H), 7.09 (s, 4H), 3.34 – 3.24 (m, 4H), 2.89 – 2.62 (m, 4H), 1.47 (d, *J* = 6.6 Hz, 12H), 1.25 (d, *J* = 6.7 Hz, 12H), 1.08 (d, *J* = 6.9 Hz, 12H), 0.94 (d, *J* = 6.8 Hz, 12H). ^{13}C NMR (126 MHz, $CDCl_3$) δ 165.65, 146.53, 146.10, 135.53, 130.42, 125.54, 124.83, 124.42, 29.23, 26.67, 26.63, 24.19, 24.14. (Flahaut et al., 2009)

General Procedure for the Suzuki-Miyaura Cross-Coupling of Amides. An oven-dried vial equipped with a stir bar was charged with an amide substrate (neat, 1.0 equiv), potassium carbonate (typically, 3.0 equiv), boronic acid (typically, 2.0 equiv), Pd-NHC catalyst (typically, 0.5 mol%), water (typically, 5 equiv) placed under a positive pressure of argon, and subjected to three evacuation/backfilling cycles under high vacuum. Toluene (typically, 0.5 M) was added with vigorous stirring at room temperature, the reaction mixture was placed in a preheated oil bath and stirred for the indicated time. After the indicated time, the reaction mixture was cooled down to room temperature, diluted with CH_2Cl_2 (10 mL), filtered, and concentrated. A sample was analyzed by ^{1}H NMR ($CDCl_3$, 500 MHz) and GC-MS to obtain conversion, selectivity and

yield using internal standard and comparison with authentic samples. Purification by chromatography on silica gel (EtOAc/hexanes) afforded the tile product.

Phenyl(*p*-tolyl)methanone (Scheme 1, 9a)

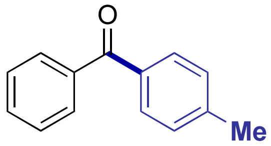

According to the general procedure, the reaction of *tert*-butyl benzoyl(phenyl)carbamate (0.20 mmol), 4-methylphenylboronic acid (2 equiv), K_2CO_3 (3 equiv), H_2O (5 equiv) and [(IPr)Pd(μ-Cl)Cl]$_2$ (0.05 mol%) in toluene (0.5 M) for 16 h at room temperature, afforded after work-up and chromatography the title compound in 89 % yield (34.9 mg). White solid. ^{1}H NMR (500 MHz, $CDCl_3$) δ 7.79 (d, *J* = 6.9 Hz, 2H), 7.73 (d, *J* = 8.2 Hz, 2H), 7.58 (t, *J* = 7.4 Hz, 1H), 7.47 (t, *J* = 7.7 Hz, 2H), 7.28 (d, *J* = 7.9 Hz, 2H), 2.44 (s, 3H). ^{13}C NMR (125 MHz, $CDCl_3$) δ 196.64, 143.37, 138.09, 135.01, 132.29, 130.44, 130.06, 129.10, 128.34, 21.80. NMR spectroscopic data agreed with literature values (Zhou et al., 2019).

(4-Methoxyphenyl)(phenyl)methanone (Scheme 1, 9b)

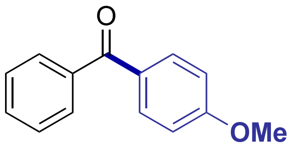

According to the general procedure, the reaction of *tert*-butyl benzoyl(phenyl)carbamate (0.20 mmol), phenylboronic acid (2 equiv), K_2CO_3 (3 equiv), H_2O (5 equiv) and [(IPr)Pd(μ-Cl)Cl]$_2$ (0.25 mol%) in toluene (1 M) for 12 h at room temperature, afforded after work-up and chromatography the title compound in 98 % yield (41.6 mg). White solid. ^{1}H NMR (500 MHz, $CDCl_3$) δ 7.83 (d, *J* = 8.8 Hz, 2H), 7.76 (d, *J* = 6.8 Hz, 2H), 7.57 (t, *J* = 7.4 Hz, 1H), 7.47 (t, *J* = 7.7 Hz, 2H), 6.97 (d, *J* = 8.9 Hz, 2H), 3.89 (s, 3H). ^{13}C NMR (125 MHz, $CDCl_3$) δ 195.71, 163.36, 138.43, 132.70, 132.03, 130.31, 129.87, 128.33, 113.69, 55.65. NMR spectroscopic data agreed with literature values (Zhou et al., 2019).

Phenyl(4-(trifluoromethyl)phenyl)methanone (Scheme 1, 9c)

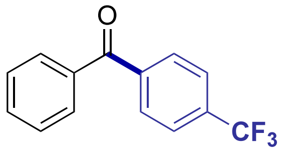

According to the general procedure, the reaction of *tert*-butyl benzoyl(phenyl)carbamate (0.20 mmol), (4-trifluoromethyl)phenylboronic acid (2 equiv), K_2CO_3 (3 equiv), H_2O (5 equiv) and [(IPr)Pd(μ-Cl)Cl]$_2$ (0.25 mol%) in toluene (1 M) for 12 h at room temperature, afforded after work-up and chromatography the title compound in 82 % yield (41.1 mg). ^{1}H NMR (500 MHz, $CDCl_3$) δ 7.89 (d, J = 8.0 Hz, 2H), 7.81 (d, J = 6.9 Hz, 2H), 7.76 (d, J = 8.1 Hz, 2H), 7.63 (t, J = 7.5 Hz, 1H), 7.51 (t, J = 7.8 Hz, 2H). ^{13}C NMR (125 MHz, $CDCl_3$) δ 195.65, 140.86, 136.86, 133.85 (q, J^F = 32.7 Hz), 133.22, 130.27, 130.24, 128.66, 125.48 (q, J^F = 3.6 Hz), 123.81 (q, J^F = 272.7 Hz). ^{19}F NMR (471 MHz, $CDCl_3$) δ -63.01. NMR spectroscopic data agreed with literature values (Zhou et al., 2019).

1-Phenyldecan-1-one (Scheme 1, 9d)

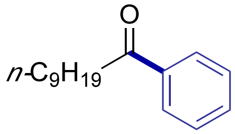

According to the general procedure, the reaction of *tert*-butyl decanoyl(phenyl)carbamate (0.20 mmol), phenylboronic acid (2 equiv), K_2CO_3 (3 equiv), H_2O (5 equiv) and [(IPr)Pd(μ-Cl)Cl]$_2$ (0.5 mol%) in toluene (1 M) for 12 h at room temperature, afforded after work-up and chromatography the title compound in 91 % yield (42.3 mg). White solid. ^{1}H NMR (500 MHz, $CDCl_3$) δ 7.96 (d, J = 7.3 Hz, 2H), 7.55 (t, J = 7.4 Hz, 1H), 7.46 (t, J = 7.6 Hz, 2H), 2.96 (t, J = 7.4 Hz, 2H), 1.73 (p, J = 7.4 Hz, 2H), 1.41 – 1.25 (m, 12H), 0.88 (t, J = 6.8 Hz, 3H). ^{13}C NMR (125 MHz, $CDCl_3$) δ 200.77, 137.25, 132.98, 128.68, 128.20, 38.79, 32.03, 29.64, 29.62, 29.54, 29.43, 24.55, 22.82, 14.26. NMR spectroscopic data agreed with literature values (Lei et al., 2017).

(4-Methoxyphenyl)(phenyl)methanone (Scheme 1, 9b')

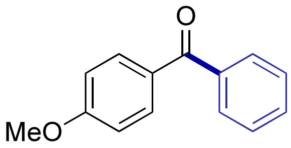

According to the general procedure, the reaction of *tert*-butyl (4-methoxybenzoyl)(phenyl)carbamate (0.20 mmol), phenylboronic acid (2 equiv), K_2CO_3 (3 equiv), H_2O (5 equiv) and [(IPr)Pd(μ-Cl)Cl]$_2$ (0.5 mol%) in toluene (0.5 M) for 12 h at room temperature, afforded after work-up and chromatography the title compound in 93 % yield (39.5 mg). White solid. ^{1}H NMR (500 MHz, $CDCl_3$) δ 7.83 (d, *J* = 8.8 Hz, 2H), 7.76 (d, *J* = 6.8 Hz, 2H), 7.57 (t, *J* = 7.4 Hz, 1H), 7.47 (t, *J* = 7.7 Hz, 2H), 6.97 (d, *J* = 8.9 Hz, 2H), 3.89 (s, 3H). ^{13}C NMR (125 MHz, $CDCl_3$) δ 195.71, 163.36, 138.43, 132.70, 132.03, 130.31, 129.87, 128.33, 113.69, 55.65. NMR spectroscopic data agreed with literature values (Zhou et al., 2019).

Methyl 4-benzoylbenzoate (Scheme 1, 9e)

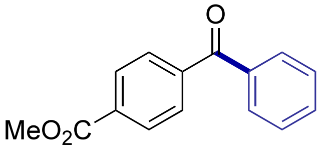

According to the general procedure, the reaction of *tert*-butyl phenyl((4-(methoxycarbonyl)benzoyl)carbamate (0.20 mmol), phenylboronic acid (2 equiv), K_2CO_3 (3 equiv), H_2O (5 equiv) and [(IPr)Pd(μ-Cl)Cl]$_2$ (0.5 mol%) in toluene (1 M) for 12 h at room temperature, afforded after work-up and chromatography the title compound in 99 % yield (47.5 mg). White solid. ^{1}H NMR (500 MHz, $CDCl_3$) δ 8.15 (d, *J* = 8.4 Hz, 2H), 7.84 (d, *J* = 8.4 Hz, 2H), 7.81 (d, *J* = 6.9 Hz, 2H), 7.62 (t, *J* = 7.5 Hz, 1H), 7.50 (t, *J* = 7.8 Hz, 2H), 3.97 (s, 3H). ^{13}C NMR (125 MHz, $CDCl_3$) δ 196.19, 166.47, 141.47, 137.10, 133.37, 133.09, 130.25, 129.92, 129.65, 128.61, 52.63. NMR spectroscopic data agreed with literature values (Zhou et al., 2019).

Phenyl(o-tolyl)methanone (Scheme 1, 9d)

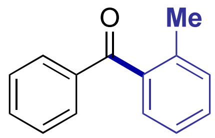

According to the general procedure, the reaction of *tert*-butyl benzoyl(phenyl)carbamate (0.20 mmol), 2-methylphenylboronic acid (2 equiv), K_2CO_3 (3 equiv), H_2O (5 equiv) and [(IPr*)Pd(μ-Cl)Cl]$_2$ (0.25 mol%) in toluene (1 M) for 12 h at room temperature, afforded after work-up and chromatography the title compound in 83 % yield (32.6 mg). ^{1}H NMR (500 MHz, $CDCl_3$) δ 7.80 (d, J = 6.8 Hz, 2H), 7.58 (t, J = 7.4 Hz, 1H), 7.46 (t, J = 7.7 Hz, 2H), 7.39 (t, J = 7.4 Hz, 1H), 7.34 – 7.28 (m, 2H), 7.24 (d, J = 7.5 Hz, 1H), 2.33 (s, 3H). ^{13}C NMR (125 MHz, $CDCl_3$) δ 198.78, 138.78, 137.90, 136.89, 133.26, 131.13, 130.37, 130.27, 128.66, 128.60, 125.33, 20.13. NMR spectroscopic data agreed with literature values (Zhou et al., 2019).

Determination of Kinetic Profiles Amides

General Procedure. An oven-dried vial equipped with a stir bar was charged with *tert*-butyl benzoyl(phenyl)carbamate (neat, 0.20 mmol, 1.0 equiv), potassium carbonate (3.0 equiv), 4-Tolylboronic acid (2.0 equiv), water (5.0 equiv), NHC-Pd (0.05 mol% for [(IPr)Pd(μ-Cl)Cl]$_2$, 0.1 mol% for other catalysts), placed under a positive pressure of argon, and subjected to three evacuation/backfilling cycles under high vacuum. Toluene (0.5 M) was added with vigorous stirring and the reaction mixture was stirred at 23 °C for the indicated time. After the indicated time, the reaction mixture was diluted with CH_2Cl_2 (10 mL), filtered, and concentrated. The sample was analyzed by ^{1}H NMR ($CDCl_3$, 500 MHz) and/or GC- MS to obtain conversion, selectivity and yield using internal standard and comparison with authentic samples.

Determination of Turnover Number

General Procedure. An oven-dried vial equipped with a stir bar was charged with an amide substrate (neat, 1.0 equiv), potassium carbonate (3.0 equiv), boronic acid (2.0 equiv) placed under a positive pressure of argon, and subjected to three evacuation/backfilling cycles under high vacuum. A stock solution of [(IPr)Pd(μ-Cl)Cl]$_2$ (0.0025 mol %) in 2-Methyltetrahydrofuran (0.5 M) was added with vigorous stirring at room temperature, the

reaction mixture was placed in a preheated oil bath at 100 °C or 120 °C and stirred the same temperature for 16 h. After the indicated time, the reaction mixture was cooled down to room temperature, diluted with CH_2Cl_2 (10 mL), filtered, and concentrated. The sample was analyzed by ^{1}H NMR ($CDCl_3$, 500 MHz) and/or GC-MS to obtain conversion, selectivity and yield using internal standard and comparison with authentic samples.

General Procedure for the Suzuki-Miyaura Cross-Coupling of Aryl Chlorides. An oven-dried vial equipped with a stir bar was charged with an aryl chloride or bromide (neat, 1.0 equiv), potassium carbonate (typically, 3.0 equiv), boronic acid (typically, 2.0 equiv) placed under a positive pressure of argon, and subjected to three evacuation/backfilling cycles under high vacuum. Ethanol (typically, 0.5 M) containing Pd-NHC catalyst (typically, 0.25 mol %) was added with vigorous stirring at indicated temperature, the reaction mixture was placed in a preheated oil bath and stirred for the indicated time. After the indicated time, the reaction mixture was cooled down to room temperature, diluted with CH_2Cl_2 (10 mL), filtered, and concentrated. A sample was analyzed by ^{1}H NMR ($CDCl_3$, 500 MHz) and GC-MS to obtain conversion, selectivity and yield using internal standard and comparison with authentic samples. Purification by chromatography on silica gel (EtOAc/hexanes) afforded the title product.

Determination of Kinetic Profiles Aryl Chlorides

General Procedure. An oven-dried vial equipped with a stir bar was charged with 4-chlorotoluene (neat, 0.20 mmol, 1.0 equiv), KO*t*Bu (1.1 equiv) or K_2CO_3 (2.2 equiv), phenylboronic acid (1.05 equiv) placed under a positive pressure of argon, and subjected to three evacuation/backfilling cycles under high vacuum. EtOH (0.5 M) containing NHC-Pd (0.5 mol% for [(IPr)Pd(μ-Cl)Cl]$_2$, 1 mol% for (IPr)Pd(1-*t*Bu-ind)Cl) was added with vigorous stirring and the reaction mixture was stirred at 23 °C for the indicated time. After the indicated time, the reaction mixture was diluted with CH_2Cl_2 (10 mL), filtered, and concentrated. The sample was analyzed by ^{1}H NMR ($CDCl_3$, 500 MHz) and/or GC- MS to obtain conversion, selectivity and yield using internal standard and comparison with authentic samples.

[(IPr)Pd(μ-Cl)Cl]$_2$ Catalyzed Suzuki-Miyaura Cross-Coupling at Low Palladium Loading

General Procedure. An oven-dried vial equipped with a stir bar was charged with an aryl chloride or bromide (neat, 1.0 equiv), potassium carbonate (typically, 3.0 equiv), boronic acid (typically, 2.0 equiv) placed under a positive pressure of argon, and subjected to three evacuation/backfilling cycles under high vacuum. Ethanol (typically, 0.5 M) containing [(IPr)Pd(μ-Cl)Cl]$_2$ (typically, 0.0025 mol %) was added with vigorous stirring at indicated temperature, the reaction mixture was placed in a preheated oil bath and stirred for the indicated time. After the indicated time, the reaction mixture was cooled down to room temperature, diluted with CH_2Cl_2 (10 mL), filtered, and concentrated. A sample was analyzed by ^{1}H NMR ($CDCl_3$, 500 MHz) and GC-MS to obtain conversion, selectivity and yield using internal standard and comparison with authentic samples.

Experiments on Activation of Pd(II) to Pd(0)

Rates of Activation of [(IPr)Pd(allyl)Cl], [(IPr)Pd(cin)Cl] and [(IPr)Pd(μ-Cl)Cl]$_2$ in the presence of dvds were determined according to the previous report (Melvin et al., 2015).

General Procedure. KO*t*Bu (9.8 mg, 0.087 mmol) was dissolved in 300 μL of d_4-MeOH along with 100 μL of a 0.87 M solution of dvds in d_4-MeOH. [(IPr)Pd(allyl)Cl] (5.0 mg, 0.0087 mmol), [(IPr)Pd(cin)Cl] (5.6 mg, 0.0087 mmol), or [(IPr)Pd(μ-Cl)Cl]$_2$ (4.9 mg, 0.00435 mmol) was dissolved in 100 μL of d_4-MeOH. These solutions were combined in a J. Young NMR tube at −78 °C. The reaction mixture was degassed on a Schlenk line, after which dinitrogen was introduced into the NMR tube. An array of ^{1}H NMR spectra was taken at 25 °C over the course of 3 h. During this time, the growth of the methyl protons of the (IPr)Pd(dvds) product were monitored.

4-Methylbiphenyl (Table S2)

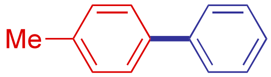

According to the general procedure, the reaction of 4-chlorotoluene (0.20 mmol), phenylboronic acid (1.05 equiv), K_2CO_3 (2.2 equiv) and [(IPr)Pd(μ-Cl)Cl]$_2$ (0.05 mol%) in EtOH (0.5 M) for 12 h at 23 °C, afforded after work-up and chromatography the title compound in 98 % yield (33.1 mg). White solid. ^{1}H NMR (500 MHz, $CDCl_3$) δ 7.58 (d, *J* = 7.9 Hz, 2H), 7.50 (d, *J* = 7.8 Hz, 2H), 7.43 (t, *J* = 7.7 Hz, 2H), 7.34 (t, 1H), 7.26 (d, *J* = 7.7 Hz, 3H), 2.40 (s, 3H). ^{13}C NMR (125 MHz, $CDCl_3$) δ 141.30, 138.50, 137.16, 129.62, 128.85, 127.14, 127.12, 21.25. NMR spectroscopic data agreed with literature values (Liu et al., 2018).

4-Methoxybiphenyl (Table S2)

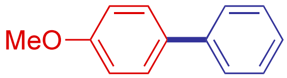

According to the general procedure, the reaction of 4-chloroanisole (0.20 mmol), phenylboronic acid (1.05 equiv), K_2CO_3 (2.2 equiv) and [(IPr)Pd(μ-Cl)Cl]$_2$ (0.05 mol%) in EtOH (0.5 M) for 12 h at 23 °C, afforded after work-up and chromatography the title compound in 98 % yield (36.2 mg). White solid. ^{1}H NMR (500 MHz, $CDCl_3$) δ 7.59 – 7.49 (m, 4H), 7.42 (t, *J* = 7.8 Hz, 2H), 7.31 (t, *J* = 7.3 Hz, 1H), 6.98 (d, *J* = 8.7 Hz, 2H), 3.86 (s, 3H). ^{13}C NMR (125 MHz, $CDCl_3$) δ 159.39, 141.08, 134.04, 128.96, 128.40, 126.98, 126.90, 114.45, 55.60. NMR spectroscopic data agreed with literature values (Monguchi et al., 2012).

4'-Methoxy-2,6-dimethylbiphenyl (Table S2)

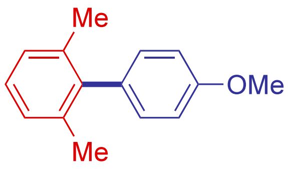

According to the general procedure, the reaction of 2-chloro-*m*-xylene (0.20 mmol), (4-methoxyl)phenylboronic acid (1.05 equiv), K_2CO_3 (2.2 equiv) and [(IPr)Pd(μ-Cl)Cl]$_2$ (0.05 mol%) in EtOH (0.5 M) for 12 h at 23 °C, afforded after work-up and chromatography the

title compound in 98 % yield (41.7 mg). ^{1}H NMR (500 MHz, $CDCl_3$) δ 7.18 – 7.13 (m, 1H), 7.10 (d, J = 8.0 Hz, 2H), 7.06 (d, J = 8.7 Hz, 2H), 6.97 (d, J = 8.6 Hz, 2H), 3.86 (s, 3H), 2.04 (s, 6H). ^{13}C NMR (125 MHz, $CDCl_3$) δ 158.40, 141.66, 136.67, 133.47, 130.21, 127.38, 127.02, 113.95, 55.37, 21.05. NMR spectroscopic data agreed with literature values (Lipshutz et al, 2008).

4-Cyano-4'-methylbiphenyl (Scheme 2)

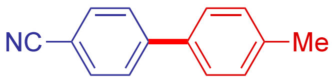

According to the general procedure, the reaction of 4-chlorobenzonitrile (0.20 mmol), 4-methylphenylboronic acid (2 equiv), K_2CO_3 (3 equiv) and $[(IPr)Pd(\mu\text{-}Cl)Cl]_2$ (0.25 mol%) in EtOH (0.5 M) for 12 h at 23 °C, afforded after work-up and chromatography the title compound in 97 % yield (37.5 mg). White solid. ^{1}H NMR (500 MHz, $CDCl_3$) δ 7.74 – 7.62 (m, 4H), 7.50 (d, J = 8.2 Hz, 2H), 7.29 (d, J = 7.9 Hz, 2H), 2.42 (s, 3H). ^{13}C NMR (125 MHz, $CDCl_3$) δ 145.71, 138.87, 136.37, 132.67, 129.95, 127.57, 127.17, 119.16, 110.65, 21.31. NMR spectroscopic data agreed with literature values (Liu et al., 2011).

4-Hydroxy-4'-methylbiphenyl (Scheme 2)

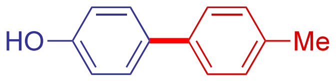

According to the general procedure, the reaction of 4-bromophenol (0.20 mmol), 4-methylphenylboronic acid (2 equiv), K_2CO_3 (3 equiv) and $[(IPr)Pd(\mu\text{-}Cl)Cl]_2$ (0.25 mol%) in EtOH (0.5 M) for 12 h at 60 °C, afforded after work-up and chromatography the title compound in 95 % yield (35.1 mg). White solid. ^{1}H NMR (500 MHz, $CDCl_3$) δ 7.46 (d, J = 8.4 Hz, 2H), 7.44 (d, J = 7.9 Hz, 2H), 7.22 (d, J = 7.8 Hz, 2H), 6.89 (d, J = 8.5 Hz, 2H), 4.80 (s, 1H), 2.38 (s, 3H). ^{13}C NMR (125 MHz, $CDCl_3$) δ 154.97, 138.03, 136.56, 134.14, 129.58, 128.33, 126.71, 115.72, 21.20. NMR spectroscopic data agreed with literature values (Edwards et al., 2014).

4-Amino-4'-methylbiphenyl (Scheme 2)

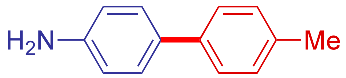

According to the general procedure, the reaction of 4-chloroaniline (0.20 mmol), 4-methylphenylboronic acid (2 equiv), K_2CO_3 (3 equiv) and [(IPr)Pd(μ-Cl)Cl]$_2$ (0.25 mol%) in EtOH (0.5 M) for 12 h at 60 °C, afforded after work-up and chromatography the title compound in 78 % yield (28.6 mg). Yellow solid. ^{1}H NMR (500 MHz, $CDCl_3$) δ 7.43 (d, *J* = 8.2 Hz, 2H), 7.40 (d, *J* = 8.5 Hz, 2H), 7.21 (d, *J* = 7.9 Hz, 2H), 6.75 (d, *J* = 8.5 Hz, 2H), 3.70 (s, 2H), 2.37 (s, 3H). ^{13}C NMR (125 MHz, $CDCl_3$) δ 145.70, 138.46, 136.04, 131.76, 129.50, 127.96, 126.41, 115.53, 21.18. NMR spectroscopic data agreed with literature values (Kamio et al., 2019).

2-Amino-4'-methylbiphenyl (Scheme 2)

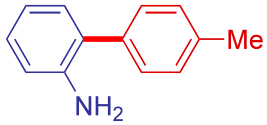

According to the general procedure, the reaction of 2-chloroaniline (0.20 mmol), 4-methylphenylboronic acid (2 equiv), K_2CO_3 (3 equiv) and [(IPr)Pd(μ-Cl)Cl]$_2$ (0.25 mol%) in EtOH (0.5 M) for 12 h at 23 °C, afforded after work-up and chromatography the title compound in 92 % yield (33.7 mg). Yellow oil. ^{1}H NMR (500 MHz, $CDCl_3$) δ 7.35 (d, *J* = 8.1 Hz, 2H), 7.25 (d, *J* = 7.2 Hz, 2H), 7.17 – 7.09 (m, 2H), 6.82 (dt, *J* = 7.4, 1.2 Hz, 1H), 6.76 (dd, *J* = 8.0, 1.2 Hz, 1H), 3.73 (s, 2H), 2.40 (s, 3H). ^{13}C NMR (125 MHz, $CDCl_3$) δ 143.66, 136.96, 136.64, 130.58, 129.62, 129.07, 128.42, 127.77, 118.76, 115.68, 21.33. NMR spectroscopic data agreed with literature values (Ke et al., 2014).

2-(*p*-Tolyl)pyridine (Scheme 2)

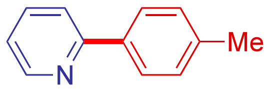

According to the general procedure, the reaction of 2-chloropyridine (0.20 mmol), 4-methylphenylboronic acid (2 equiv), K_2CO_3 (3 equiv) and [(IPr)Pd(μ-Cl)Cl]$_2$ (0.25 mol%) in EtOH (0.5 M) for 12 h at 60 °C, afforded after work-up and chromatography the title compound in 82 % yield (27.7 mg). Yellow oil. ^{1}H NMR (500 MHz, $CDCl_3$) δ 8.68 (d, *J* = 4.7

Hz, 1H), 7.89 (d, J = 7.9 Hz, 2H), 7.76 – 7.68 (m, 2H), 7.28 (d, J = 7.9 Hz, 2H), 7.20 (t, J = 4.7 Hz, 1H), 2.41 (s, 3H). ^{13}C NMR (125 MHz, $CDCl_3$) δ 157.49, 149.61, 138.94, 136.66, 136.63, 129.48, 126.77, 121.79, 120.26, 21.28. NMR spectroscopic data agreed with literature values (Iglesias et al., 2012).

3-(6-Methoxynaphthalen-2-yl)pyridine (Scheme 2)

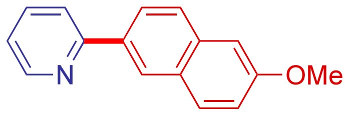

According to the general procedure, the reaction of 2-chloropyridine (0.20 mmol), 6-methoxy-2-naphthaleneboronic acid (2 equiv), K_2CO_3 (3 equiv) and [(IPr)Pd(μ-Cl)Cl]$_2$ (0.25 mol%) in EtOH (0.5 M) for 12 h at 60 °C, afforded after work-up and chromatography the title compound in 97 % yield (45.6 mg). Yellow solid. ^{1}H NMR (500 MHz, $CDCl_3$) δ 8.73 (d, J = 4.4 Hz, 1H), 8.42 (s, 1H), 8.11 (dd, J = 8.6, 1.6 Hz, 1H), 7.87 – 7.82 (m, 3H), 7.78 (td, J = 7.7, 1.6 Hz, 1H), 7.25 – 7.22 (m, 1H), 7.21 – 7.15 (m, 2H), 3.95 (s, 3H). ^{13}C NMR (125 MHz, $CDCl_3$) δ 158.38, 157.61, 149.86, 136.90, 135.07, 134.74, 130.39, 129.15, 127.41, 126.28, 125.20, 122.00, 120.63, 119.33, 105.78, 55.49. NMR spectroscopic data agreed with literature values (Zhang et al., 2015).

Methyl 2-(*p*-tolyl)nicotinate (Scheme 2)

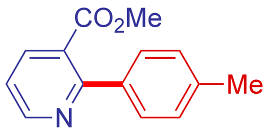

According to the general procedure, the reaction of methyl 2-chloronicotinate (0.20 mmol), 4-methylphenylboronic acid (2 equiv), K_2CO_3 (3 equiv) and [(IPr)Pd(μ-Cl)Cl]$_2$ (0.25 mol%) in THF (0.5 M) for 12 h at 60 °C, afforded after work-up and chromatography the title compound in 75 % yield (34.1 mg). Colorless solid. ^{1}H NMR (500 MHz, $CDCl_3$) δ 8.76 (dd, J = 4.7, 1.5 Hz, 1H), 8.06 (dd, J = 7.8, 1.6 Hz, 1H), 7.45 (d, J = 8.0 Hz, 2H), 7.30 (dd, J = 7.8, 4.8 Hz, 1H), 7.24 (d, J = 7.8 Hz, 2H), 3.72 (s, 3H), 2.40 (s, 3H). ^{13}C NMR (125 MHz, $CDCl_3$) δ 168.92, 158.86, 151.40, 138.83, 137.92, 137.21, 129.07, 128.56, 126.96, 121.38, 52.52, 21.49. **HRMS** calcd for

$C_{14}H_{14}NO_2$ (M^+ + H) 228.0986, found 228.1019. NMR spectroscopic data agreed with literature values (Galenko et al., 2017).

3-(*p*-Tolyl)pyridine (Scheme 2)

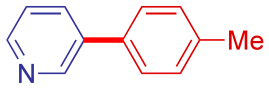

According to the general procedure, the reaction of 3-chloropyridine (0.20 mmol), 4-methylphenylboronic acid (2 equiv), K_2CO_3 (3 equiv) and [(IPr)Pd(μ-Cl)Cl]$_2$ (0.25 mol%) in EtOH (0.5 M) for 12 h at 60 °C, afforded after work-up and chromatography the title compound in 89 % yield (30.1 mg). Yellow oil. ^{1}H NMR (500 MHz, $CDCl_3$) δ 8.84 (s, 1H), 8.57 (d, *J* = 4.9 Hz, 1H), 7.86 (d, *J* = 7.9 Hz, 1H), 7.49 (d, *J* = 8.1 Hz, 2H), 7.35 (dd, *J* = 7.9, 4.8 Hz, 1H), 7.29 (d, *J* = 7.8 Hz, 2H), 2.41 (s, 3H). ^{13}C NMR (125 MHz, $CDCl_3$) δ 148.36, 148.34, 138.18, 136.71, 135.09, 134.27, 129.94, 127.13, 123.65, 21.31. NMR spectroscopic data agreed with literature values (Iglesias et al., 2012).

3-(6-Methoxynaphthalen-2-yl)pyridine (Scheme 2)

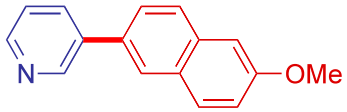

According to the general procedure, the reaction of 3-chloropyridine (0.20 mmol), 6-methoxy-2-naphthaleneboronic acid (2 equiv), K_2CO_3 (3 equiv) and [(IPr)Pd(μ-Cl)Cl]$_2$ (0.25 mol%) in EtOH (0.5 M) for 12 h at 60 °C, afforded after work-up and chromatography the title compound in 96 % yield (45.1 mg). White solid. ^{1}H NMR (500 MHz, $CDCl_3$) δ 8.97 (d, *J* = 2.2 Hz, 1H), 8.61 (dd, *J* = 4.9, 1.6 Hz, 1H), 8.02 – 7.95 (m, 2H), 7.85 (d, *J* = 8.6 Hz, 1H), 7.81 (d, *J* = 8.9 Hz, 1H), 7.68 (dd, *J* = 8.5, 1.8 Hz, 1H), 7.43 – 7.37 (m, 1H), 7.20 (dd, *J* = 8.9, 2.5 Hz, 1H), 7.18 (s, 1H), 3.95 (s, 3H). ^{13}C NMR (125 MHz, $CDCl_3$) δ 158.27, 148.44, 148.24, 136.87, 134.59, 134.29, 133.00, 129.90, 129.23, 127.85, 126.11, 125.62, 123.77, 119.67, 105.73, 55.52. NMR spectroscopic data agreed with literature values (Voets et al., 2005).

5-(3,4-Dimethoxyphenyl)-1*H*-indole (Scheme 2)

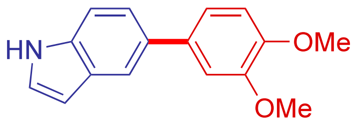

According to the general procedure, the reaction of 5-chloro-1*H*-indole (0.20 mmol), 3,4-dimethoxyphenylboronic acid (2 equiv), K_2CO_3 (3 equiv) and [(IPr)Pd(μ-Cl)Cl]$_2$ (0.25 mol%) in EtOH (0.5 M) for 12 h at 60 °C, afforded after work-up and chromatography the title compound in 89 % yield (45.1 mg). White solid. ^{1}H NMR (500 MHz, $CDCl_3$) δ 8.18 (brs, 1H), 7.82 (s, 1H), 7.43 (q, J = 8.4 Hz, 2H), 7.26 – 7.23 (m, 1H), 7.22 – 7.15 (m, 2H), 6.96 (d, J = 8.0 Hz, 1H), 6.61 (s, 1H), 3.97 (s, 3H), 3.93 (s, 3H). ^{13}C NMR (125 MHz, $CDCl_3$) δ 149.20, 148.09, 135.89, 135.25, 133.50, 128.54, 124.97, 121.94, 119.61, 119.01, 111.68, 111.30, 111.07, 103.10, 56.17, 56.09. NMR spectroscopic data agreed with literature values (Jakab et al., 2015).

2-(4-Fluorophenyl)quinoline (Scheme 2)

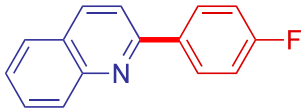

According to the general procedure, the reaction of 2-chloroquinoline (0.20 mmol), 4-fluorophenylboronic acid (2 equiv), K_2CO_3 (3 equiv) and [(IPr)Pd(μ-Cl)Cl]$_2$ (0.25 mol%) in EtOH (0.5 M) for 12 h at 60 °C, afforded after work-up and chromatography the title compound in 97 % yield (43.3 mg).White solid. ^{1}H NMR (500 MHz, $CDCl_3$) δ 8.22 (d, J = 8.6 Hz, 1H), 8.20 – 8.10 (m, 3H), 7.83 (d, J = 8.6 Hz, 2H), 7.74 (t, J = 7.8 Hz, 1H), 7.53 (t, J = 7.6 Hz, 1H), 7.21 (t, J = 8.1 Hz, 2H). ^{13}C NMR (125 MHz, $CDCl_3$) δ 163.94 (d, J^F = 249.1 Hz), 156.37, 148.37, 137.04, 135.96 (d, J^F = 3.1 Hz), 129.92, 129.79, 129.54 (d, J^F = 8.4 Hz), 127.61, 127.22, 126.48, 118.76, 115.91 (d, J^F = 21.5 Hz). ^{19}F NMR (471 MHz, $CDCl_3$) δ -112.52. NMR spectroscopic data agreed with literature values (Wu et al., 2015).

2,4-Dimethoxy-6-(*p*-tolyl)-1,3,5-triazine (Scheme 2)

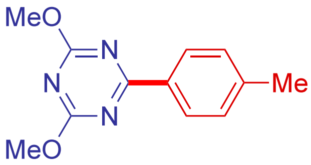

According to the general procedure, the reaction of 2-chloro-4,6-dimethoxy-1,3,5-triazine (0.20 mmol), 4-methylphenylboronic acid (2 equiv), K_2CO_3 (3 equiv) and [(IPr)Pd(μ-Cl)Cl]$_2$ (0.25 mol%) in EtOH (0.5 M) for 12 h at 23 °C, afforded after work-up and chromatography the title compound in 98 % yield (45.3 mg). White solid. ^{1}H NMR (500 MHz, $CDCl_3$) δ 8.39 (d, J = 8.2 Hz, 2H), 7.29 (d, J = 8.0 Hz, 2H), 4.12 (s, 6H), 2.43 (s, 3H). ^{13}C NMR (125 MHz, $CDCl_3$) δ 175.05, 172.98, 143.69, 132.49, 129.40, 129.18, 55.29, 21.84. NMR spectroscopic data agreed with literature values (Li et al., 2013).

4-(4-(*tert*-Butyl)phenyl)-7-nitrobenzo[*c*][1,2,5]oxadiazole (Scheme 2)

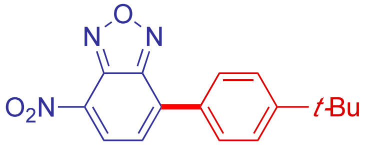

According to the general procedure, the reaction of 4-chloro-7-nitrobenzofurazan (0.20 mmol), 4-*tert*-butylphenylboronic acid (2 equiv), K_2CO_3 (3 equiv) and [(IPr)Pd(μ-Cl)Cl]$_2$ (0.25 mol%) in EtOH (0.5 M) for 12 h at 23 °C, afforded after work-up and chromatography the title compound in 57 % yield (33.9 mg). Yellow solid. ^{1}H NMR (500 MHz, $CDCl_3$) δ 8.59 (d, J = 7.7 Hz, 1H), 8.02 (d, J = 8.6 Hz, 2H), 7.74 (d, J = 7.7 Hz, 1H), 7.62 (d, J = 8.6 Hz, 2H), 1.40 (s, 9H). ^{13}C NMR (125 MHz, $CDCl_3$) δ 155.27, 149.83, 143.52, 139.00, 135.01, 131.18, 130.95, 129.09, 126.61, 125.42, 35.21, 31.29. NMR spectroscopic data agreed with literature values (Singh et al., 2008).

3',4'-Dimethoxy-4-formylbiphenyl (Scheme 2)

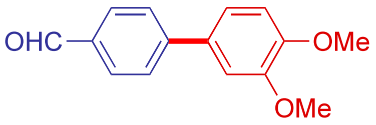

According to the general procedure, the reaction of 4-chlorobenzaldehyde (0.20 mmol), 3,4-dimethoxyphenylboronic acid (2 equiv), K_2CO_3 (3 equiv) and [(IPr)Pd(μ-Cl)Cl]$_2$ (0.25 mol%) in EtOH (0.5 M) for 12 h at 60 °C, afforded after work-up and chromatography the title compound in 97 % yield (46.9 mg). Yellow solid. ^{1}H NMR (500 MHz, $CDCl_3$) δ 10.04 (s, 1H), 7.93 (d, J = 8.2 Hz, 2H), 7.72 (d, J = 8.2 Hz, 2H), 7.22 (d, J = 9.0 Hz, 1H), 7.15 (s, 1H), 6.98 (d, J = 8.4 Hz, 1H), 3.97 (s, 3H), 3.95 (s, 3H). ^{13}C NMR (125 MHz, $CDCl_3$) δ 192.00, 149.78, 149.51, 147.13,

134.95, 132.65, 130.44, 127.37, 120.13, 111.68, 110.54, 56.18. NMR spectroscopic data agreed with literature values (Wang et al., 2015).

4'-Methyl-[1,1'-biphenyl]-4-carboxylic acid (Scheme 2)

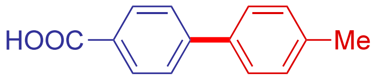

According to the general procedure, the reaction of 4-chlorobenzoic acid (0.20 mmol), 4-methylphenylboronic acid (2 equiv), KOH (3 equiv) and $[(IPr)Pd(\mu\text{-}Cl)Cl]_2$ (0.25 mol%) in EtOH (0.5 M) for 12 h at 100 °C, afforded after work-up and chromatography the title compound in 90 % yield (38.2 mg). White solid. ^{1}H NMR (500 MHz, DMSO-d_6) δ 12.93 (s, 1H), 8.00 (d, J = 8.6 Hz, 2H), 7.77 (d, J = 8.4 Hz, 2H), 7.63 (d, J = 8.2 Hz, 2H), 7.30 (d, J = 7.9 Hz, 2H), 2.35 (s, 3H). ^{13}C NMR (125 MHz, DMSO-d_6) δ 167.15, 144.22, 137.78, 136.10, 129.93, 129.67, 129.29, 126.77, 126.47, 20.71. NMR spectroscopic data agreed with literature values (Edwards et al., 2014).

5-(*p*-Tolyl)benzo[*d*][1,3]dioxole (Scheme 2)

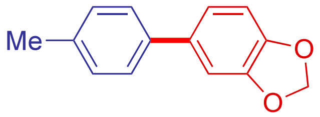

According to the general procedure, the reaction of 4-chlorotoluene (0.20 mmol), 3,4-(methylenedioxy)phenylboronic acid (2 equiv), K_2CO_3 (3 equiv) and $[(IPr)Pd(\mu\text{-}Cl)Cl]_2$ (0.25 mol%) in EtOH (0.5 M) for 12 h at 60 °C, afforded after work-up and chromatography the title compound in 86 % yield (36.5 mg). White solid. ^{1}H NMR (500 MHz, $CDCl_3$) δ 7.41 (d, J = 8.1 Hz, 2H), 7.22 (d, J = 7.8 Hz, 2H), 7.10 – 7.02 (m, 2H), 6.87 (d, J = 7.9 Hz, 1H), 5.99 (s, 2H), 2.38 (s, 3H). ^{13}C NMR (125 MHz, $CDCl_3$) δ 148.19, 146.95, 138.22, 136.81, 135.73, 129.58, 126.87, 120.50, 108.67, 107.69, 101.22, 21.20. NMR spectroscopic data agreed with literature values (Kamio et al, 2019).

4-Hydroxy-4'-methylbiphenyl (Scheme 2)

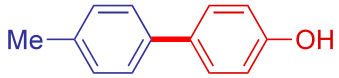

According to the general procedure, the reaction of 4-chlorotoluene (0.20 mmol), 4-hydroxyphenylboronic acid (2 equiv), K_2CO_3 (3 equiv) and [(IPr)Pd(μ-Cl)Cl]$_2$ (0.25 mol%) in EtOH (0.5 M) for 12 h at 60 °C, afforded after work-up and chromatography the title compound in 89 % yield (32.8 mg). White solid. ^{1}H NMR (500 MHz, $CDCl_3$) δ 7.46 (d, J = 8.4 Hz, 2H), 7.44 (d, J = 7.9 Hz, 2H), 7.22 (d, J = 7.8 Hz, 2H), 6.89 (d, J = 8.5 Hz, 2H), 4.80 (s, 1H), 2.38 (s, 3H). ^{13}C NMR (125 MHz, $CDCl_3$) δ 154.97, 138.03, 136.56, 134.14, 129.58, 128.33, 126.71, 115.72, 21.20. NMR spectroscopic data agreed with literature values (Edwards et al., 2014).

2-(2,4-Difluorophenyl)pyridine (Scheme 2)

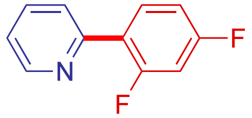

According to the general procedure, the reaction of 2-chloropyridine (0.20 mmol), 2,4-difluorophenylboronic acid (2 equiv), K_2CO_3 (3 equiv) and [(IPr)Pd(μ-Cl)Cl]$_2$ (0.25 mol%) in EtOH (0.5 M) for 12 h at 60 °C, afforded after work-up and chromatography the title compound in 99 % yield (37.8 mg). Yellow oil. ^{1}H NMR (500 MHz, $CDCl_3$) δ 8.76 – 8.66 (m, 1H), 8.06 – 7.95 (m, 1H), 7.84 – 7.69 (m, 2H), 7.24 – 7.21 (m, 1H), 7.07 – 6.86 (m, 2H). ^{13}C NMR (125 MHz, $CDCl_3$) δ 163.34 (dd, J^F = 251.2, 11.6 Hz), 160.72 (dd, J^F = 252.2, 12.0 Hz), 152.70, 149.93, 136.61, 132.27 (dd, J^F = 9.6, 4.4 Hz), 124.36 (d, J^F = 9.1 Hz), 123.95 (d, J^F = 12.8 Hz), 122.57, 112.03 (dd, J^F = 21.3, 2.5 Hz), 104.50 (t, J^F = 26.5 Hz). ^{19}F NMR (471 MHz, $CDCl_3$) δ -109.36, -112.97. NMR spectroscopic data agreed with literature values (Bergmann et al, 2018).

2,4-Dimethoxy-6-(thiophen-3-yl)-1,3,5-triazine (Scheme 2)

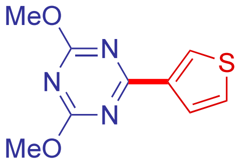

According to the general procedure, the reaction of 2-chloro-4,6-dimethoxy-1,3,5-triazine (0.20 mmol), 3-thienylboronic acid (2 equiv), K_2CO_3 (3 equiv) and [(IPr)Pd(μ-Cl)Cl]$_2$ (0.25 mol%) in MeOH (0.5 M) for 12 h at 60 °C, afforded after work-up and chromatography the title compound in 87 % yield (38.8 mg). ^{1}H NMR (500 MHz, $CDCl_3$) δ 8.45 (dd, J = 3.0, 1.0 Hz, 1H), 7.87 (dd,

J = 5.1, 1.0 Hz, 1H), 7.34 (dd, *J* = 5.1, 3.1 Hz, 1H), 4.07 (s, 6H). ^{13}C NMR (126 MHz, $CDCl_3$) δ 172.89, 171.14, 139.44, 131.72, 127.64, 126.26, 55.22. NMR spectroscopic data agreed with literature values (Li et al., 2019).

3-(4,6-Dimethoxy-1,3,5-triazin-2-yl)benzonitrile (Scheme 2)

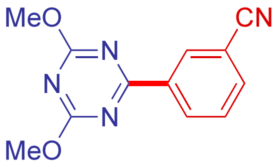

According to the general procedure, the reaction of 2-chloro-4,6-dimethoxy-1,3,5-triazine (0.20 mmol), 3-cyanophenylboronic acid (2 equiv), K_2CO_3 (3 equiv) and [(IPr)Pd(μ-Cl)Cl]$_2$ (0.25 mol%) in EtOH (0.5 M) for 12 h at 60 °C, afforded after work-up and chromatography the title compound in 84 % yield (40.7 mg). White solid. ^{1}H NMR (500 MHz, $CDCl_3$) δ 8.80 (t, *J* = 1.4 Hz, 1H), 8.72 (dt, *J* = 8.0, 1.4 Hz, 1H), 7.84 (dt, *J* = 7.7, 1.4 Hz, 1H), 7.62 (t, *J* = 7.9 Hz, 1H), 4.15 (s, 6H). ^{13}C NMR (125 MHz, $CDCl_3$) δ 173.18, 173.04, 136.49, 135.79, 133.09, 132.84, 129.60, 118.40, 113.14, 55.66. HRMS calcd for $C_{12}H_{11}N_4O_2$ (M^+ + H) 243.0877, found 243.0851.

2-Methoxy-5-(*p*-tolyl)pyridine (Scheme 3)

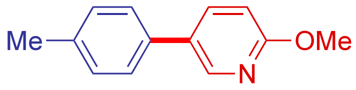

According to the general procedure, the reaction of 4-chlorotoluene (0.20 mmol), 6-methoxy-3-pyridinylboronic acid (2 equiv), K_2CO_3 (3 equiv) and [(IPr)Pd(μ-Cl)Cl]$_2$ (0.25 mol%) in EtOH (0.5 M) for 12 h at 60 °C, afforded after work-up and chromatography the title compound in 97 % yield (38.6 mg). Colorless oil. ^{1}H NMR (500 MHz, $CDCl_3$) δ 8.37 (d, *J* = 2.5 Hz, 1H), 7.77 (dd, *J* = 8.6, 2.6 Hz, 1H), 7.42 (d, *J* = 8.1 Hz, 2H), 7.26 – 7.23 (m, 2H), 6.81 (d, *J* = 8.6 Hz, 1H), 3.98 (s, 3H), 2.40 (s, 3H). ^{13}C NMR (125 MHz, $CDCl_3$) δ 163.58, 144.92, 137.49, 137.27, 135.18, 130.21, 129.82, 126.68, 110.88, 53.66, 21.24. NMR spectroscopic data agreed with literature values (Liu et al., 2011).

***tert*-Butyl (4-(quinolin-2-yl)phenyl)carbamate (Scheme 3)**

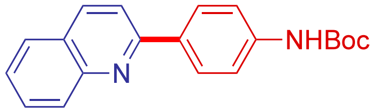

According to the general procedure, the reaction of 2-chloroquinoline (0.20 mmol), 4-(*N*-Boc-amino)phenylboronic acid (2 equiv), K_2CO_3 (3 equiv) and [(IPr)Pd(μ-Cl)Cl]$_2$ (0.25 mol%) in EtOH (0.5 M) for 12 h at 60 °C, afforded after work-up and chromatography the title compound in 98 % yield (62.8 mg).White solid. ^{1}H NMR (500 MHz, $CDCl_3$) δ 8.19 (d, *J* = 8.6 Hz, 1H), 8.14 (d, *J* = 8.8 Hz, 3H), 7.85 (d, *J* = 8.6 Hz, 1H), 7.81 (d, *J* = 8.1 Hz, 1H), 7.71 (td, *J* = 7.3, 1.5 Hz, 1H), 7.57 – 7.47 (m, 3H), 6.61 (brs, 1H), 1.55 (s, 10H). ^{13}C NMR (125 MHz, $CDCl_3$) δ 156.84, 152.65, 148.44, 139.73, 136.81, 134.37, 129.74, 128.44, 127.58, 127.37, 127.19, 126.18, 118.75, 118.56, 29.86, 28.52. NMR spectroscopic data agreed with literature values (Cashion et al., 2011).

4'-Methyl-*N*-phenyl-[1,1'-biphenyl]-4-carboxamide (Scheme 3)

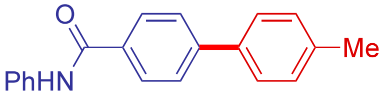

According to the general procedure, the reaction of 4-chloro-*N*-phenylbenzamide (0.20 mmol), 4-methylphenylboronic acid (2 equiv), K_2CO_3 (3 equiv) and [(IPr)Pd(μ-Cl)Cl]$_2$ (0.25 mol%) in EtOH (0.5 M) for 12 h at 100 °C, afforded after work-up and chromatography the title compound in 93 % yield (53.4 mg). White solid. ^{1}H NMR (500 MHz, DMSO-d_6) δ 10.27 (s, 1H), 8.05 (d, *J* = 8.4 Hz, 2H), 7.84 – 7.78 (m, 4H), 7.66 (d, *J* = 8.2 Hz, 2H), 7.36 (t, *J* = 7.4 Hz, 2H), 7.32 (d, *J* = 7.9 Hz, 2H), 7.11 (t, *J* = 7.3 Hz, 1H), 2.36 (s, 3H). ^{13}C NMR (126 MHz, DMSO-d_6) δ 165.13, 142.98, 139.19, 137.58, 136.17, 133.37, 129.63, 128.58, 128.31, 126.71, 126.22, 123.61, 120.34, 20.70. HRMS calcd for $C_{20}H_{17}ON$ (M^+ + H) 288.1383, found 288.1378.

3-Methoxy-6-phenylpyridazine (Scheme 3)

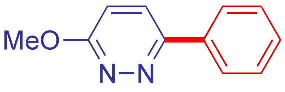

According to the general procedure, the reaction of 3-chloro-6-methoxypyridazine (0.20 mmol), phenylboronic acid (2 equiv), K_2CO_3 (3 equiv) and [(IPr)Pd(μ-Cl)Cl]$_2$ (0.25 mol%) in EtOH (0.5 M) for 12 h at 60 °C, afforded after work-up and chromatography the title compound in 89 % yield (33.1 mg). White solid. ^{1}H NMR (500 MHz, $CDCl_3$) δ 8.01 (d, *J* = 8.3 Hz, 2H), 7.79 (d, *J* = 9.3 Hz, 1H), 7.54 – 7.43 (m, 3H), 7.05 (d, *J* = 9.2 Hz, 1H), 4.19 (s, 3H). ^{13}C NMR (125 MHz, $CDCl_3$) δ 164.44, 155.38, 136.39, 129.56, 129.07, 127.25, 126.67, 117.83, 55.04. NMR spectroscopic data agreed with literature values (Clapham et al., 2008).

4'-Methyl-[1,1'-biphenyl]-4-carboxamide (Scheme 3)

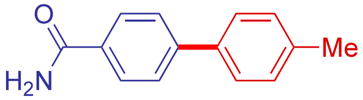

According to the general procedure, the reaction of 4-chlorobenzamide (0.20 mmol), 4-methylphenylboronic acid (2 equiv), KOH (3 equiv) and [(IPr)Pd(μ-Cl)Cl]$_2$ (0.25 mol%) in EtOH (0.5 M) for 12 h at 100 °C, afforded after work-up and chromatography the title compound in 95 % yield (40.1 mg). White solid. ^{1}H NMR (500 MHz, DMSO-d_6) δ 8.00 (s, 1H), 7.95 (d, *J* = 8.4 Hz, 2H), 7.72 (d, *J* = 8.4 Hz, 2H), 7.62 (d, *J* = 8.2 Hz, 2H), 7.37 (s, 1H), 7.29 (d, *J* = 7.9 Hz, 2H), 2.35 (s, 3H). ^{13}C NMR (126 MHz, $CDCl_3$) δ 167.53, 142.64, 137.41, 136.30, 132.76, 129.58 (d, *J* = 9.3 Hz), 128.11, 126.66 (d, *J* = 8.5 Hz), 126.09, 20.68. NMR spectroscopic data agreed with literature values (Asghar et al., 2017).

***N*-Methyl-[1,1'-biphenyl]-4-carboxamide (Scheme 3)**

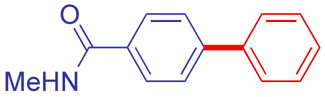

According to the general procedure, the reaction of 4-chloro-*N*-methylbenzamide (0.20 mmol), phenylboronic acid (2 equiv), K_2CO_3 (3 equiv) and [(IPr)Pd(μ-Cl)Cl]$_2$ (0.25 mol%) in EtOH (0.5 M) for 12 h at 60 °C, afforded after work-up and chromatography the title compound in 93 % yield (39.2 mg). White solid. ^{1}H NMR (500 MHz, $CDCl_3$) δ 7.84 (d, *J* = 8.4 Hz, 2H), 7.63 (d, *J* = 8.3 Hz, 2H), 7.59 (d, *J* = 7.0 Hz, 2H), 7.45 (t, *J* = 7.6 Hz, 2H), 7.38 (t, *J* = 7.3 Hz, 1H), 6.38 (s, 1H), 3.03 (d, *J* = 4.8 Hz, 3H). ^{13}C NMR (125 MHz, $CDCl_3$) δ 168.10, 144.24,

140.13, 133.41, 129.02, 128.07, 127.50, 127.31, 127.29, 27.00. NMR spectroscopic data agreed with literature values (Rao et al., 2017).

_N,N_-Dimethyl-[1,1'-biphenyl]-4-carboxamide (Scheme 3)

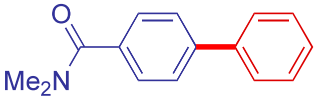

According to the general procedure, the reaction of 4-chloro-*N,N*-dimethylbenzamide (0.20 mmol), phenylboronic acid (2 equiv), K_2CO_3 (3 equiv) and [(IPr)Pd(μ-Cl)Cl]$_2$ (0.25 mol%) in EtOH (0.5 M) for 12 h at 60 °C, afforded after work-up and chromatography the title compound in 88 % yield (39.6 mg). White solid.^{1}H NMR (500 MHz, $CDCl_3$) δ 7.62 (d, *J* = 8.2 Hz, 2H), 7.60 (d, *J* = 7.4 Hz, 2H), 7.50 (d, *J* = 8.2 Hz, 2H), 7.45 (t, *J* = 7.7 Hz, 2H), 7.37 (t, *J* = 7.3 Hz, 1H), 3.14 (s, 3H), 3.04 (s, 3H). ^{13}C NMR (125 MHz, $CDCl_3$) δ 171.59, 142.56, 140.46, 135.19, 128.99, 127.85, 127.78, 127.27, 127.17, 39.79, 35.56. NMR spectroscopic data agreed with literature values (Asghar et al., 2017).

4-Hydroxy-4'-methylbiphenyl (Scheme 3)

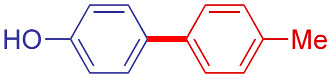

According to the general procedure, the reaction of 4-chlorophenol (0.20 mmol), 4-methylphenylboronic acid (2 equiv), K_2CO_3 (3 equiv) and [(IPr)Pd(μ-Cl)Cl]$_2$ (0.25 mol%) in EtOH (0.5 M) for 12 h at 60 °C, afforded after work-up and chromatography the title compound in 82 % yield (30.2 mg). White solid. ^{1}H NMR (500 MHz, $CDCl_3$) δ 7.46 (d, *J* = 8.4 Hz, 2H), 7.44 (d, *J* = 7.9 Hz, 2H), 7.22 (d, *J* = 7.8 Hz, 2H), 6.89 (d, *J* = 8.5 Hz, 2H), 4.80 (s, 1H), 2.38 (s, 3H). ^{13}C NMR (125 MHz, $CDCl_3$) δ 154.97, 138.03, 136.56, 134.14, 129.58, 128.33, 126.71, 115.72, 21.20. NMR spectroscopic data agreed with literature values (Edwards et al., 2014).

_N_-Methyl-[1,1'-biphenyl]-4-sulfonamide (Scheme 3)

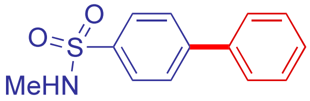

According to the general procedure, the reaction of 4-chloro-*N*-methylbenzenesulfonamide (0.20 mmol), phenylboronic acid (2 equiv), K_2CO_3 (3 equiv) and [(IPr)Pd(μ-Cl)Cl]$_2$ (0.05 mol%) in EtOH (0.5 M) for 12 h at 100 °C, afforded after work-up and chromatography the title compound in 98 % yield (48.4 mg). White solid.^{1}H NMR (500 MHz, $CDCl_3$) δ 7.94 (d, *J* = 8.5 Hz, 2H), 7.73 (d, *J* = 8.4 Hz, 2H), 7.61 (d, *J* = 7.4 Hz, 2H), 7.48 (t, *J* = 7.6 Hz, 2H), 7.42 (t, *J* = 7.3 Hz, 1H), 4.64 (q, *J* = 5.4 Hz, 1H), 2.71 (d, *J* = 5.4 Hz, 3H). ^{13}C NMR (125 MHz, $CDCl_3$) δ 145.81, 139.42, 137.51, 129.19, 128.62, 127.89, 127.45, 29.52. NMR spectroscopic data agreed with literature values (Nordvall et al., 2007).

4,7-Di(thiophen-2-yl)benzo[*c*][1,2,5]thiadiazole (Scheme 3)

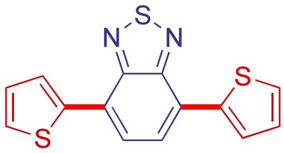

According to the general procedure, the reaction of 4,7-dibromobenzo[c][1,2,5]thiadiazole (0.20 mmol), 2-thienylboronic acid (3 equiv), K_2CO_3 (3 equiv) and [(IPr)Pd(μ-Cl)Cl]$_2$ (0.025 mol%) in EtOH (0.5 M) for 12 h at 100 °C, afforded after work-up and chromatography the title compound in 98 % yield (58.9 mg). Red solid. ^{1}H NMR (500 MHz, $CDCl_3$) δ 8.10 (dd, *J* = 3.7, 1.2 Hz, 2H), 7.84 (s, 2H), 7.45 (dd, *J* = 5.0, 1.2 Hz, 2H), 7.21 (dd, *J* = 5.1, 3.7 Hz, 2H). ^{13}C NMR (125 MHz, $CDCl_3$) δ 152.82, 139.57, 128.23, 127.72, 127.01, 126.18, 125.96. NMR spectroscopic data agreed with literature values (Chen et al., 2015).

2-(3,4,5-Trifluorophenyl)pyrazine (Scheme 3)

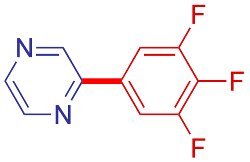

According to the general procedure, the reaction of 2-chloropyrazine (0.20 mmol), (3,4,5-trifluorophenyl)boronic acid (2 equiv), K_2CO_3 (3 equiv) and [(IPr)Pd(μ-Cl)Cl]$_2$ (0.0025 mol%) in EtOH (0.5 M) for 12 h at 100 °C, afforded after work-up and chromatography the title compound in 84 % yield (35.3 mg). White solid. ^{1}H NMR (500 MHz, $CDCl_3$) δ 8.97 (d, *J* =

1.1 Hz, 1H), 8.63 (t, J = 2.0 Hz, 1H), 8.56 (d, J = 2.3 Hz, 1H), 7.75 – 7.65 (m, 2H). ^{13}C NMR (125 MHz, $CDCl_3$) δ 151.88 (ddd, J = 250.7, 10.3, 4.0 Hz), 149.62 (q, J = 2.5 Hz), 144.40, 144.09, 141.72, 141.09 (dt, J = 255.6, 15.3 Hz), 132.47 (td, J = 7.5, 4.5 Hz), 111.19 (dd, J = 17.2, 5.6 Hz). ^{19}F NMR (471 MHz, $CDCl_3$) δ -132.84 (d, J = 23.6 Hz, 2F), -158.00 (t, J = 21.2 Hz, 1F). NMR spectroscopic data agreed with literature values (Chen et al., 2015).

2-(6-Methoxypyridin-3-yl)pyrazine (Scheme 3)

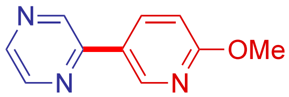

According to the general procedure, the reaction of 2-chloropyrazine (0.20 mmol), 6-methoxy-3-pyridinylboronic acid (2 equiv), K_2CO_3 (3 equiv) and $[(IPr)Pd(\mu\text{-}Cl)Cl]_2$ (0.05 mol%) in EtOH (0.5 M) for 12 h at 100 °C, afforded after work-up and chromatography the title compound in 98 % yield (36.7 mg). White solid. ^{1}H NMR (500 MHz, $CDCl_3$) δ 8.99 (s, 1H), 8.81 (s, 1H), 8.61 (s, 1H), 8.50 (s, 1H), 8.25 (d, J = 8.7 Hz, 1H), 6.88 (d, J = 8.1 Hz, 1H), 4.01 (s, 3H). ^{13}C NMR (125 MHz, $CDCl_3$) δ 165.38, 150.87, 145.88, 144.42, 142.96, 141.54, 137.30, 125.81, 111.55, 53.96. NMR spectroscopic data agreed with literature values (Chen et al., 2015).

3-(Pyridin-2-yl)quinoline (Scheme 3)

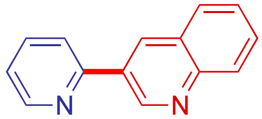

According to the general procedure, the reaction of 2-chloropyridine (0.20 mmol), 3-quinolineboronic acid (2 equiv), K_2CO_3 (3 equiv) and $[(IPr)Pd(\mu\text{-}Cl)Cl]_2$ (0.25 mol%) in EtOH (0.5 M) for 12 h at 60 °C, afforded after work-up and chromatography the title compound in 72 % yield (29.7 mg). White solid. ^{1}H NMR (500 MHz, $CDCl_3$) δ 9.55 (d, J = 2.2 Hz, 1H), 8.78 (d, J = 2.7 Hz, 2H), 8.16 (d, J = 8.4 Hz, 1H), 7.97 – 7.89 (m, 2H), 7.85 (td, J = 7.7, 1.8 Hz, 1H), 7.75 (t, J = 7.7 Hz, 1H), 7.59 (t, J = 7.5 Hz, 1H), 7.36 – 7.30 (m, 1H). ^{13}C NMR (125 MHz, $CDCl_3$) δ 155.04, 150.35, 149.45, 148.40, 137.22, 134.04, 132.07, 130.13, 129.45,

128.67, 128.03, 127.19, 122.97, 120.97. NMR spectroscopic data agreed with literature values (Chen et al., 2015).

4,6-Dimethoxy-2-(naphthalen-1-yl)pyrimidine (Scheme 3)

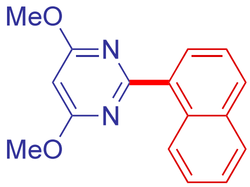

According to the general procedure, the reaction of 2-chloro-4,6-dimethoxypyrimidine (0.20 mmol), 1-naphthylboronic acid (2 equiv), K_2CO_3 (3 equiv) and [(IPr)Pd(μ-Cl)Cl]$_2$ (0.025 mol%) in EtOH (0.5 M) for 12 h at 100 °C, afforded after work-up and chromatography the title compound in 95 % yield (50.6 mg). White solid. ^{1}H NMR (500 MHz, $CDCl_3$) δ 8.92 (d, *J* = 8.3 Hz, 1H), 8.19 (d, *J* = 6.5 Hz, 1H), 7.96 (d, *J* = 8.2 Hz, 1H), 7.91 (d, *J* = 7.5 Hz, 1H), 7.60 – 7.49 (m, 3H), 6.09 (s, 1H), 4.06 (s, 6H). ^{13}C NMR (125 MHz, $CDCl_3$) δ 171.44, 165.80, 135.66, 134.29, 131.25, 130.73, 129.46, 128.59, 126.58, 126.38, 125.82, 125.20, 88.10, 54.28. NMR spectroscopic data agreed with literature values (Chen et al., 2015).

Ethyl 1-methyl-5-phenyl-1*H*-indole-2-carboxylate (Scheme 3)

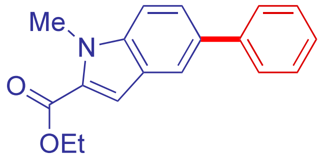

According to the general procedure, the reaction of ethyl 5-chloro-1-methyl-1H-indole-2-carboxylate (0.20 mmol), phenylboronic acid (2 equiv), K_2CO_3 (3 equiv) and [(IPr)Pd(μ-Cl)Cl]$_2$ (0.25 mol%) in EtOH (0.5 M) for 12 h at 60 °C, afforded after work-up and chromatography the title compound in 92 % yield (51.4 mg). White solid. ^{1}H NMR (500 MHz, $CDCl_3$) δ 7.87 (d, *J* = 1.8 Hz, 1H), 7.67 – 7.59 (m, 3H), 7.48 – 7.42 (m, 3H), 7.37 – 7.31 (m, 2H), 4.39 (q, *J* = 7.1 Hz, 2H), 4.11 (s, 3H), 1.43 (t, *J* = 7.1 Hz, 3H). ^{13}C NMR (125 MHz, $CDCl_3$) δ 162.33, 142.02, 139.32, 134.19, 128.88, 128.82, 127.44, 126.77, 126.49, 125.07,

120.89, 110.67, 110.55, 60.74, 31.95, 14.54. NMR spectroscopic data agreed with literature values (Chikvaidze et al., 2012).

2,4-di-*tert*-Butyl-6-(5-(4-(trifluoromethyl)phenyl)-2*H*-benzo[*d*][1,2,3]triazol-2-yl)phenol (Scheme 3)

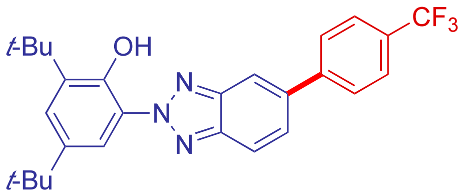

According to the general procedure, the reaction of 2,4-di-*tert*-butyl-6-(5-chloro-2H-benzo[d][1,2,3]triazol-2-yl)phenol (0.20 mmol), 4-(trifluoromethyl)phenylboronic acid (2 equiv), K_2CO_3 (3 equiv) and $[(IPr)Pd(\mu\text{-}Cl)Cl]_2$ (0.25 mol%) in EtOH (0.5 M) for 12 h at 60 °C, afforded after work-up and chromatography the title compound in 95 % yield (88.5 mg). White solid. ^1H NMR (500 MHz, $CDCl_3$) δ 11.71 (s, 1H), 8.32 (d, *J* = 2.3 Hz, 1H), 8.13 (s, 1H), 8.04 (d, *J* = 8.9 Hz, 1H), 7.83 – 7.75 (m, 4H), 7.73 (dd, *J* = 8.9, 1.6 Hz, 1H), 7.45 (d, *J* = 2.3 Hz, 1H), 1.53 (s, 9H), 1.41 (s, 9H). ^{13}C NMR (125 MHz, $CDCl_3$) δ 146.93, 144.29, 143.26, 142.53, 141.98, 139.42, 138.87, 130.07 (q, *J* = 32.8 Hz), 127.96, 127.94, 126.10 (q, *J* = 3.8 Hz), 125.55, 125.31, 124.34 (q, *J* = 272.0 Hz), 118.35, 116.32, 115.84, 35.88, 34.77, 31.66, 29.74. ^{19}F NMR (471 MHz, $CDCl_3$) δ -62.47. NMR spectroscopic data agreed with literature values (Chen et al., 2015).

4'-Methoxy-3,5-bis(trifluoromethyl)biphenyl (Scheme 3)

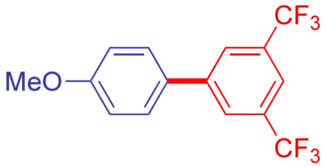

According to the general procedure, the reaction of 4-chloroanisole (0.20 mmol), (3,5-bis(trifluoromethyl)phenyl)boronic acid (2 equiv), K_2CO_3 (3 equiv) and $[(IPr)Pd(\mu\text{-}Cl)Cl]_2$ (0.05 mol%) in EtOH (0.5 M) for 12 h at 100 °C, afforded after work-up and chromatography the title compound in 76 % yield (48.7 mg). Colorless oil. ^1H NMR (500 MHz, $CDCl_3$) δ 7.97 (s, 2H), 7.80 (s, 1H), 7.55 (d, *J* = 8.5 Hz, 2H), 7.03 (d, *J* = 8.6 Hz, 2H), 3.88

(s, 3H). ^{13}C NMR (125 MHz, $CDCl_3$) δ 160.49, 143.04, 132.18 (q, J = 33.2 Hz), 130.77, 128.52, 126.79 (q, J = 3.9 Hz), 123.59 (q, J = 272.6 Hz), 120.34 (q, J = 3.7 Hz), 114.85, 55.59. ^{19}F NMR (471 MHz, $CDCl_3$) δ -62.87. NMR spectroscopic data agreed with literature values (Chen et al., 2015).

***tert*-Butyl 2-(4,6-dimethoxypyrimidin-2-yl)-1*H*-pyrrole-1-carboxylate (Scheme 3)**

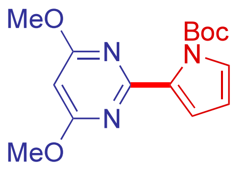

According to the general procedure, the reaction of 2-chloro-4,6-dimethoxypyrimidine (0.20 mmol), (1-(*tert*-butoxycarbonyl)-1H-pyrrol-2-yl)boronic acid (2 equiv), K_2CO_3 (3 equiv) and [(IPr)Pd(μ-Cl)Cl]$_2$ (0.25 mol%) in EtOH (0.5 M) for 12 h at 60 °C, afforded after work-up and chromatography the title compound in 87 % yield (53.1 mg). Colorless oil. ^{1}H NMR (500 MHz, $CDCl_3$) δ 7.34 – 7.28 (m, 1H), 6.78 – 6.72 (m, 1H), 6.23 (t, J = 3.0 Hz, 1H), 5.93 (s, 1H), 3.95 (s, 6H), 1.46 (s, 9H). ^{13}C NMR (125 MHz, $CDCl_3$) δ 171.13, 159.50, 149.12, 133.17, 124.96, 118.01, 110.60, 87.73, 83.68, 54.09, 27.84. NMR spectroscopic data agreed with literature values (Chen et al., 2015).

Isopropyl 2-(4-([1,1'-biphenyl]-4-carbonyl)phenoxy)-2-methylpropanoate (Scheme 4)

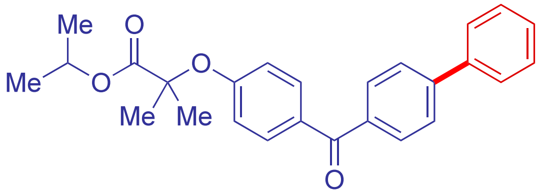

According to the general procedure, the reaction of Fenofibrate (0.20 mmol), phenylboronic acid (2 equiv), K_2CO_3 (3 equiv) and [(IPr)Pd(μ-Cl)Cl]$_2$ (0.25 mol%) in *i*-PrOH (0.5 M) for 12 h at 60 °C, afforded after work-up and chromatography the title compound in 90 % yield (72.4 mg). White solid. ^{1}H NMR (500 MHz, $CDCl_3$) δ 7.84 (d, J = 8.2 Hz, 2H), 7.80 (d, J = 8.8 Hz, 2H), 7.69 (d, J = 8.4 Hz, 2H), 7.65 (d, J = 7.5 Hz, 2H), 7.48 (t, J = 7.7 Hz, 2H), 7.40 (t, J = 7.3 Hz, 1H), 6.89 (d, J = 8.8 Hz, 2H), 5.10 (hept, J = 6.3 Hz, 1H), 1.67 (s, 6H), 1.21

(d, J = 6.3 Hz, 6H). ^{13}C NMR (125 MHz, $CDCl_3$) δ 195.29, 173.32, 159.66, 144.93, 140.20, 136.92, 132.13, 130.88, 130.55, 129.08, 128.22, 127.41, 127.03, 117.34, 79.51, 69.46, 25.52, 21.67. HRMS calcd for $C_{26}H_{27}O_4$ (M^+ + H) 403.1904, found 403.1922.

1-(4-Fluorophenyl)-4-(4-hydroxy-4-(4'-methoxy-[1,1'-biphenyl]-4-yl)piperidin-1-yl)butan-1-one (Scheme 4)

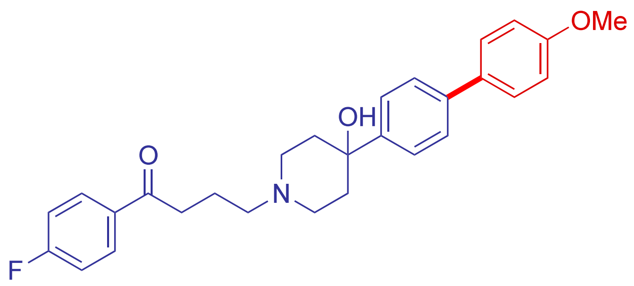

According to the general procedure, the reaction of Haloperidol (0.20 mmol), 4-methoxyphenylboronic acid (2 equiv), K_2CO_3 (5 equiv) and [(IPr)Pd(μ-Cl)Cl]$_2$ (0.25 mol%) in EtOH (0.5 M) for 12 h at 60 °C, afforded after work-up and chromatography the title compound in 93 % yield (83.2 mg). Green solid. ^{1}H NMR (500 MHz, $CDCl_3$) δ 8.08 – 7.98 (m, 2H), 7.59 – 7.46 (m, 6H), 7.13 (t, J = 8.4 Hz, 2H), 6.96 (d, J = 8.3 Hz, 2H), 3.84 (s, 3H), 2.99 (t, J = 7.1 Hz, 2H), 2.83 (d, J = 11.2 Hz, 2H), 2.60 – 2.45 (m, 4H), 2.10 (t, J = 11.0 Hz, 2H), 2.01 (p, J = 6.1 Hz, 2H), 1.75 (d, J = 13.5 Hz, 2H). ^{13}C NMR (125 MHz, $CDCl_3$) δ 198.48, 165.75 (d, J = 254.4 Hz), 159.27, 146.82, 139.61, 133.78 (d, J = 3.0 Hz), 133.36, 130.82 (d, J = 9.2 Hz), 128.16, 126.70, 125.10, 115.73 (d, J = 21.8 Hz), 114.34, 71.23, 57.96, 55.46, 49.55, 38.42, 36.41, 21.93. ^{19}F NMR (471 MHz, $CDCl_3$) δ -105.68. HRMS calcd for $C_{28}H_{31}FNO_3$ (M^+ + H) 448.2282, found 448.2313.

2-(5-Methoxy-2-methyl-1-(4'-methyl-[1,1'-biphenyl]-4-carbonyl)-1H-indol-3-yl)acetic acid (Scheme 4)

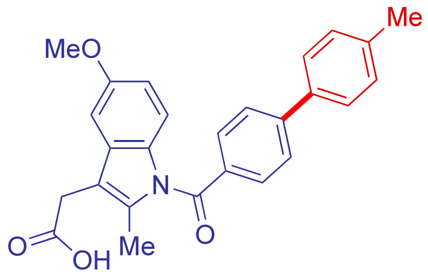

According to the general procedure, the reaction of Indomethacin (0.20 mmol), 4-methylphenylboronic acid (2 equiv), K_2CO_3 (5 equiv) and $[(IPr)Pd(\mu\text{-}Cl)Cl]_2$ (0.025 mol%) in EtOH (0.5 M) for 12 h at 100 °C, afforded after work-up and chromatography the title compound in 83 % yield (68.6 mg). White solid. ^{1}H NMR (500 MHz, DMSO-d_6) δ 12.37 (s, 1H), 7.86 (d, J = 8.3 Hz, 2H), 7.76 – 7.66 (m, 4H), 7.33 (d, J = 7.9 Hz, 2H), 7.05 (d, J = 2.5 Hz, 1H), 6.97 (d, J = 8.9 Hz, 1H), 6.70 (dd, J = 9.0, 2.5 Hz, 1H), 3.76 (s, 3H), 3.68 (s, 2H), 2.37 (s, 3H), 2.26 (s, 3H). ^{13}C NMR (125 MHz, DMSO-d_6) δ 172.62, 169.10, 155.88, 144.67, 138.52, 136.18, 135.68, 134.18, 131.06, 130.82, 130.57, 130.21, 127.30, 127.11, 114.89, 113.53, 111.70, 102.07, 55.87, 30.05, 21.21, 13.60. HRMS calcd for $C_{26}H_{24}NO_4$ (M^+ + H) 414.1700, found 414.1726.

_N_,_N_-Dimethyl-3-(2-phenyl-10_H_-phenothiazin-10-yl)propan-1-amine (Scheme 4)

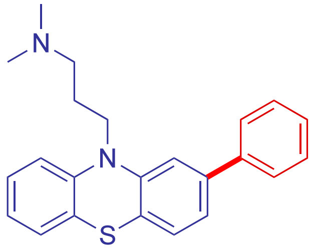

According to the general procedure, the reaction of Chlorpromazine hydrochloride (0.20 mmol), phenylboronic acid (2 equiv), K_2CO_3 (4 equiv) and $[(IPr)Pd(\mu\text{-}Cl)Cl]_2$ (0.25 mol%) in EtOH (0.5 M) for 12 h at 100 °C, afforded after work-up and chromatography the title compound in 95 % yield (68.5 mg). White solid. ^{1}H NMR (500 MHz, $CDCl_3$) δ 7.54 (d, J = 6.9 Hz, 1H), 7.43 (t, J = 7.7 Hz, 2H), 7.35 (t, J = 7.3 Hz, 1H), 7.23 – 7.11 (m, 4H), 7.09 (d, J = 1.8 Hz, 1H), 6.93 (t, J = 7.6 Hz, 2H), 4.00 (t, J = 6.9 Hz, 2H), 2.49 (t, J = 7.1 Hz, 2H), 2.24 (s, 6H), 2.03 (p, J = 7.0 Hz, 2H). ^{13}C NMR (125 MHz, $CDCl_3$) δ 145.78, 145.23, 141.07, 140.87, 128.90,

127.77, 127.64, 127.52, 127.44, 127.14, 125.32, 124.58, 122.72, 121.58, 115.86, 114.66, 57.24, 45.55, 45.51, 25.13. HRMS calcd for $C_{23}H_{25}N_2S$ (M^+ + H) 361.1738, found 361.1752.

_N_-(4-(_N_-(Cyclohexylcarbamoyl)sulfamoyl)phenethyl)-4-methoxy-4'-methyl-[1,1'-biphenyl]-3-carboxamide (Scheme 4)

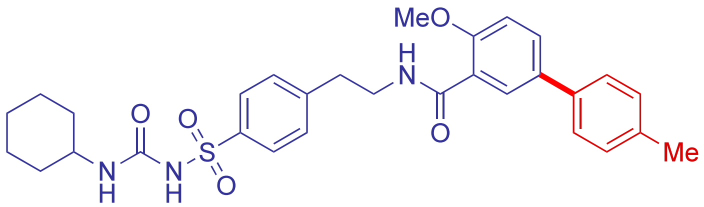

According to the general procedure, the reaction of Glibenclamide (0.20 mmol), 4-methylphenylboronic acid (2 equiv), K_2CO_3 (3 equiv) and [(IPr)Pd(μ-Cl)Cl]$_2$ (0.25 mol%) in EtOH (0.5 M) for 12 h at 100 °C, afforded after work-up and chromatography the title compound in 76 % yield (83.6 mg). White solid. ^{1}H NMR (500 MHz, $CDCl_3$) δ 8.41 (d, *J* = 2.6 Hz, 1H), 7.99 (t, *J* = 5.8 Hz, 1H), 7.88 (d, *J* = 8.1 Hz, 2H), 7.65 (dd, *J* = 8.6, 2.6 Hz, 1H), 7.48 (d, *J* = 8.1 Hz, 2H), 7.40 (d, *J* = 8.1 Hz, 2H), 7.22 (d, *J* = 7.8 Hz, 2H), 6.98 (d, *J* = 8.6 Hz, 1H), 6.44 (d, *J* = 8.0 Hz, 1H), 3.80 (s, 3H), 3.79 – 3.74 (m, 2H), 3.62 – 3.52 (m, 1H), 3.03 (t, *J* = 6.9 Hz, 2H), 2.37 (s, 3H), 1.80 (d, *J* = 8.7 Hz, 2H), 1.65 – 1.58 (m, 2H), 1.57 – 1.50 (m, 1H), 1.28 – 1.24 (m, 2H), 1.17 – 1.08 (m, 3H). ^{13}C NMR (125 MHz, $CDCl_3$) δ 165.75, 156.79, 151.05, 145.89, 138.14, 137.08, 136.88, 134.46, 131.23, 130.47, 129.82, 129.63, 127.53, 126.71, 121.25, 112.02, 56.17, 49.17, 40.74, 35.71, 33.04, 25.43, 24.66, 21.18. HRMS calcd for $C_{30}H_{36}N_3O_5S$ (M^+ + H) 550.2370, found 550.2418.

(2*S*,6'*R*)-2',4,6-Trimethoxy-6'-methyl-7-(*p*-tolyl)-3*H*-spiro[benzofuran-2,1'-cyclohexan]-2'-ene-3,4'-dione (Scheme 4)

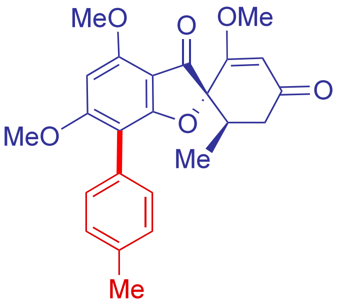

According to the general procedure, the reaction of (+)-Griseofulvin (0.20 mmol), 4-methylphenylboronic acid (2 equiv), K_2CO_3 (3 equiv) and $[(IPr)Pd(\mu\text{-}Cl)Cl]_2$ (1 mol%) in *t*-BuOH (0.5 M) for 12 h at 100 °C, afforded after work-up and chromatography the title compound in 62 % yield (50.6 mg). White solid. ^{1}H NMR (500 MHz, $CDCl_3$) δ 7.37 (d, *J* = 8.0 Hz, 2H), 7.24 (d, 2H), 6.16 (s, 1H), 5.49 (s, 1H), 4.01 (s, 3H), 3.90 (s, 3H), 3.60 (s, 3H), 3.10 – 3.03 (m, 1H), 2.77 – 2.68 (m, 1H), 2.39 (s, 3H), 2.39 – 2.33 (m, 1H), 0.97 (d, *J* = 6.7 Hz, 3H). ^{13}C NMR (125 MHz, $CDCl_3$) δ 197.53, 193.51, 171.95, 171.83, 166.46, 158.62, 137.34, 130.65, 129.00, 127.93, 107.56, 104.72, 104.47, 89.87, 88.92, 56.67, 56.57, 56.28, 40.24, 36.55, 21.54, 14.53. HRMS calcd for $C_{24}H_{25}O_6$ (M^+ + H) 409.1646, found 409.1668.

N^4-(7-(3,4-Dimethoxyphenyl)quinolin-4-yl)-N^1,N^1-diethylpentane-1,4-diamine (Scheme 4)

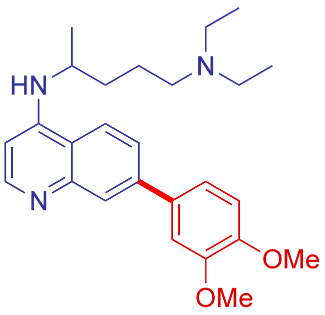

According to the general procedure, the reaction of Chloroquine diphosphate salt (0.20 mmol), 3,4-dimethoxyphenylboronic acid (2 equiv), K_2CO_3 (5 equiv) and $[(IPr)Pd(\mu\text{-}Cl)Cl]_2$ (0.025 mol%) in EtOH (0.5 M) for 12 h at 100 °C, afforded after work-up and chromatography the title compound in 89 % yield (75.1 mg). White solid. ^{1}H NMR (500 MHz, $CDCl_3$) δ 8.53 (d, *J* = 5.4 Hz, 1H), 8.16 (d, *J* = 1.9 Hz, 1H), 7.81 (d, *J* = 8.7 Hz, 1H), 7.64 (dd, *J* = 8.7, 1.9 Hz, 1H), 7.32 – 7.26 (m, 2H), 6.97 (d, *J* = 8.1 Hz, 1H), 6.41 (d, *J* = 5.4 Hz, 1H), 5.27 (d, *J* = 6.3 Hz, 1H), 3.95 (s, 3H), 3.93 (s, 3H), 3.74 (p, *J* = 6.3 Hz, 1H), 2.55 (q, *J* = 7.1 Hz, 4H), 2.47 (t, *J* = 6.8 Hz, 2H), 1.82 – 1.71 (m, 1H), 1.71 – 1.56 (m, 3H), 1.32 (d, *J* = 6.4 Hz, 3H), 1.02 (t, *J* = 7.1 Hz, 6H). ^{13}C NMR (125 MHz, $CDCl_3$) δ 151.46, 149.41, 149.11, 149.09, 149.03, 141.39, 133.21, 126.91, 123.61, 120.24, 119.67, 117.73, 111.63, 110.52, 98.97, 56.11, 56.08, 52.67, 48.36, 46.89, 34.66, 23.84, 20.41, 11.42. HRMS calcd for $C_{26}H_{36}N_3O_2$ (M^+ + H) 422.2802, found 422.2821.

Supplemental References

Frisch, M. J. et al. (2016). Gaussian 16, Revision C 01. (Gaussian Inc).

Becke, A. (1988). Density-functional exchange-energy approximation with correct asymptotic behavior. Phys. Rev. A *38*, 3098-3100.

Perdew, J. P. (1986). Density-functional approximation for the correlation energy of the inhomogeneous electron gas. Phys. Rev. B *33*, 8822-8824.

Perdew, J. P. (1986). Erratum: Density-functional approximation for the correlation energy of the inhomogeneous electron gas. Phys. Rev. B *34*, 7406.

Schäfer, A., Huber, C., and Ahlrichs, R. (1994). Fully optimized contracted Gaussian basis sets of triple zeta valence quality for atoms Li to Kr. J. Chem. Phys. *100*, 5829-5835.

Weigend, F. (2006). Accurate Coulomb-fitting basis sets for H to Rn. Phys. Chem. Chem. Phys. *8*, 1057-1065.

Kechle, W., Dolg, W., Stoll, M., and Preuss, H. (1994). Energy-adjusted pseudopotentials for the actinides. Parameter sets and test calculations for thorium and thorium monoxide. J. Chem. Phys. *100*, 7535-7542.

Leininger, T., Nicklass, A., Stoll, H., Dolg, M., and Schwerdtfeger, P. (1996). The accuracy of the pseudopotential approximation. II. A comparison of various core sizes for indium pseudopotentials in calculations for spectroscopic constants of InH, InF, and InCl. J. Chem. Phys. *105*, 1052-1059.

Grimme, S., Antony, J., Ehrlich, S., and Krieg, H. (2010). A consistent and accurate ab initio parametrization of density functional dispersion correction (DFT-D) for the 94 elements H-Pu. J. Chem. Phys. *132*, 154104-154119.

Zhao, Y., and Truhlar, D. G. (2008). Density functionals with broad applicability in chemistry. Acc. Chem. Res. *41*, 157-167.

Weigend, F., and Ahlrichs, R. (2005). Balanced basis sets of split valence, triple zeta valence and quadruple zeta valence quality for H to Rn: Design and assessment of accuracy. Phys. Chem. Chem. Phys. *7*, 3297–3305.

Barone, V., and Cossi, M. J. (1998). Quantum calculation of molecular energies and energy gradients in solution by a conductor solvent model. Phys. Chem. A *102*, 1995-2001.

Tomasi, J., and Persico, M. (1994). Molecular interactions in solution: an overview of methods based on continuous distributions of the solvent. Chem. Rev. *94*, 2027-2094.

Zhou, T., Li, G., Nolan, S. P., and Szostak, M. (2019). [Pd(NHC)(acac)Cl]: Well-Defined, Air-Stable, and Readily Available Precatalysts for Suzuki and Buchwald–Hartwig Cross-coupling (Transamidation) of Amides and Esters by N–C/O–C Activation. Org. Lett. *21*, 3304-3309.

Lei, P., Meng, G., Ling, Y., An, J., and Szostak, M. (2017). Pd-PEPPSI: Pd-NHC Precatalyst for Suzuki−Miyaura Cross-Coupling Reactions of Amides. J. Org. Chem. *82*, 6638-6646.

Liu, C., Li, G., Shi, S., Meng, G., Lalancette, R., Szostak, R., and Szostak, M. (2018). Acyl and Decarbonylative Suzuki Coupling of N-Acetyl Amides: Electronic Tuning of Twisted, Acyclic Amides in Catalytic Carbon-Nitrogen Bond Cleavage. ACS Catal. *8*, 9131-9139

Monguchi, Y., Hattori, T., Miyamoto, Y., Yanase, T., Sawama, Y., and Sajiki, H. (2012). Palladium on Carbon-Catalyzed Cross-Coupling using Triarylbismuths. Adv. Synth. Catal. *354*, 2561-2567.

Lipshutz, B. H., Petersen, T. B., and Abela, A. R. (2008). Room Temperature Suzuki-Miyaura Couplings in Water Facilitated by Nonionic Amphiphiles. Org. Lett. *10*, 1333-336.

Al-Huniti, M. H., Rivera-Chávez, J., Colón, K. L., Stanley, J. L., Burdette, J. E., Pearce, C. J., Oberlies, N. H., and Croatt, M. P. (2018). Development and Utilization of a Palladium-Catalyzed Dehydration of Primary Amides To Form Nitriles. Org. Lett. *20*, 6046-6050.

Ackermann, L., Lygin, A. V., and Hofmann, N. (2011). Ruthenium-catalyzed oxidative annulation by cleavage of C-H/N-H bonds. Angew. Chem. Int. Ed. *50*, 6379-6382.

Patel, B., Firkin, C. R., Snape, E. W., Jenkin, S. L., Brown, D., Chaffey, J. G. K., Hopes, P. A., Reens, C. D., Butters, M., and Moseley, J. D. (2012). Process Development and Scale-Up of AZD7545, a PDK Inhibitor. Org. Process Res. Dev. *16*, 447-460.

Sun, Y.-H., Sun, T.-Y., Wu, Y.-D., Zhang, X., and Rao, Y. (2016). A diversity-oriented synthesis of bioactive benzanilides via a regioselective C(sp^2)–H hydroxylation strategy. Chem. Sci. *7*, 2229-2238.

Wang, H., Sun, S., and Cheng, J. (2017). Copper-Catalyzed Arylsulfonylation and Cyclizative Carbonation of N-(Arylsulfonyl)acrylamides Involving Desulfonative Arrangement toward Sulfonated Oxindoles. Org. Lett. *19*, 5844-5847.

Zhang, K., Xu, X., Zheng, J., Yao, H., Huang, Y., and Lin, A. (2017). [3 + 3] Cycloaddition of in Situ Formed Azaoxyallyl Cations with 2-Alkenylindoles: An Approach to Tetrahydro-β-carbolinones. Org. Lett. *19*, 2596-2599.

Deska, J., del Pozo Ochoa, C., and Backvall, J.-E. (2010). Chemoenzymatic Dynamic Kinetic Resolution of Axially Chiral Allenes. Chem. Eur. J. *16*, 4447-4451.

Flahaut, A., Toutah, K., Mangeney, P., Roland, S. (2009). Diethylzinc−Mediated Allylation of Carbonyl Compounds Catalyzed by [(NHC)(PR_3)PdX_2] and [(NHC)Pd(η^3−allyl)Cl] Complexes. Eur. J. Inorg. Chem. *35*, 5422-5432.

Liu, N., Wang, L., and Wang, Z.-X. (2011). Room-temperature Nickel-Catalysed Cross-couplings of Aryl Chlorides with Arylzincs. Chem. Commun. *47*, 1598-1600.

Edwards, G. A., Trafford, M. A., Hamilton, A. E., Buxton, A. M., Bardeaux, M. C., and Chalker, J. M. (2014). Melamine and Melamine-Formaldehyde Polymers as Ligands for Palladium and Application to Suzuki-Miyaura Cross-Coupling Reactions in Sustainable Solvents. J. Org. Chem. *79*, 2094-2104.

Kamio, S., Kageyuki, I., Osaka, I., and Yoshida, H. (2019). Anthranilamide (aam)-Substituted Arylboranes in Direct Carbon–Carbon Bond Forming Reactions. Chem. Commun. *55*, 2624-2627.

Ke, H., Chen, X., and Zou, G. (2014). N-Heterocyclic Carbene-Assisted, Bis(phosphine)nickel-Catalyzed Cross-Couplings of Diarylborinic Acids with Aryl Chlorides, Tosylates, and Sulfamates. J. Org. Chem. *79*, 7132-7140.

Iglesias, M. J., Prieto, A., and Nicasio, M. C. (2012). Kumada-Tamao-Corriu Coupling of Heteroaromatic Chlorides and Aryl Ethers Catalyzed by (IPr)Ni(allyl)Cl. Org. Lett. *14*, 4318-4321.

Zhang, S.-S., Jiang, C.-Y., Wu, J.-Q., Liu, X.-G., Li, Q., Huang, Z.-S., Li, D., and Wang, H. (2015). Cp*Rh(iii) and Cp*Ir(iii)-catalysed Redox-neutral C–H Arylation with Quinone Diazides: Quick and Facile Synthesis of Arylated Phenols. Chem. Commun. *51*, 10240-10243.

Galenko, A. V., Shakirova, F. M., Galenko, E. E., Novikov, M. S., and Khlebnikov, A. F. (2017). Fe(II)/Au(I) Relay Catalyzed Propargylisoxazole to Pyridine Isomerization: Access to 6-Halonicotinates. J. Org. Chem. *82*, 5367-5379.

Voets, M., Antes, I., Scherer, C., Muller-Vieira, U., Biemel, K., Barassin, C., Marchais-Oberwinkler, S., and Hartmann, R. W. (2005). Heteroaryl-substituted Naphthalenes and Structurally Modified Derivatives: Selective Inhibitors of CYP11B2 for the Treatment of Congestive Heart Failure and Myocardial Fibrosis. J. Med. Chem. *48*, 6632-6642.

Jakab, A., Dalicsek, Z., Holczbauer, T., Hamza, A., Papai, I., Finta, Z., Timari, G., and Soos, T. ´(2015). Superstable Palladium(0) Complex as an Air- and Thermostable Catalyst for Suzuki Coupling Reactions. Eur. J. Org. Chem. 60-66.

Wu, K., Huang, Z., Liu, C., Zhang, H., and Lei, A. (2015). Aerobic C–N bond activation: a simple strategy to construct pyridines and quinolines. Chem. Commun. *51*, 2286-2289.

Li, X., Zhang, J., Geng, Y., and Jin, Z. (2013). Nickel-Catalyzed Suzuki-Miyaura Coupling of Heteroaryl Ethers with Arylboronic Acids. J. Org. Chem. *78*, 5078-5084.

Singh, R., Ramesh, U., Huang, J., Issakani, S., Tsvetkov, L., and Petroski, M. (2008). Ubiquitin Ligase Inhibitors. WO 2008115259, Sep 25, 2008.

Wang, M., Yuan, X., Li, H., Ren, L., Sun, Z., Hou, Y., and Chu, W. (2015). Nickel-catalysed Suzuki–Miyaura Cross-coupling Reactions of Aryl Halides with Arylboronic Acids in Ionic Liquids. Catal. Commun. *58*, 154-157.

Bergmann, A. M., Oldham, A. M., You, W., and Brown, M. K. (2018). Copper-catalyzed Cross-coupling of Aryl-, Primary Alkyl-, and Secondary Alkylboranes with Heteroaryl Bromides. Chem. Commun. *54*, 5381-5384.

Li, D., He, X., Xu, C., Huang, F., Liu, N., Shen, D., and Liu, F. (2019). N-Heterocarbene Palladium Complexes with Dianisole Backbones: Synthesis, Structure, and Catalysis. Organometallics *38*, 2539-2552.

Liu, C., Ni, Q., Bao, F., and Qiu, J. (2011). A Simple and Efficient Protocol for a Palladium-Catalyzed Ligand-Free Suzuki Reaction at Room Temperature in Aqueous DMF. Green Chem. *13*, 1260-1266.

Cashion, D. K., Chen, G., Kasi, D., Kolb, C., Liu, C., Sinha, A., Szardenings, A. K., Wang, E., Yu, C., Zhang, W., Gangadharmath, U. B., and Walsh, J. C. (2011). Imaging Agents for Detecting Neurological Disorders. WO 2011119565, Sep 29, 2011.

Clapham, K. M., Batsanov, A. S., Greenwood, R. D. R., Bryce, M. R., Smith, A. E., and Tarbit, B. (2008). Functionalized Heteroarylpyridazines and Pyridazin-3(2H)-one Derivatives via Palladium-Catalyzed Cross-Coupling Methodology. J. Org. Chem. *73*, 2176-2181.

Asghar, S., Tailor, S. B., Elorriaga, D., and Bedford, R. B. (2017). Cobalt-Catalyzed Suzuki Biaryl Coupling of Aryl Halides. Angew. Chem., Int. Ed. *56*, 16367-16370.

Rao, S. N., Reddy, N. N. K., Samanta, S., and Adimurthy, S. (2017). I_2-Catalyzed Oxidative Amidation of Benzyl Amines and Benzyl Cyanides under Mild Conditions. J. Org. Chem. *82*, 13632-13642.

Nordvall, G., and Yngve, U. (2007). Novel quinazolines as 5-ht6 modulators. WO 2007108744, Sep 27, 2007.

Chen, L., Ren, P., and Carrow, B. P. (2016). Tri(1-adamantyl)-phosphine: Expanding the Boundary of Electron-Releasing Character Available to Organophosphorus Compounds. J. Am. Chem. Soc. *138*, 6392-6395.

Chikvaidze, I., Barbakadze, N. N., and Samsoniya, S. A. (2012). Some New Derivatives of 5-Aryl-, 2,5-Diaryl- and 2-Ethoxycarbonyl-5-aryl-indoles. Arkivoc *6*, 143-154.

Melvin, P. R., Nova, A., Balcells, D., Dai, W., Hazari, N., Hruszkewycz, D. P., Shah, H. P., and Tudge, M. T. (2015). Design of a Versatile and Improved Precatalyst Scaffold for Palladium Catalyzed Cross-Coupling: (η^3-1-*t*Bu-indenyl)$_2$(μ-Cl)$_2$Pd$_2$. ACS Catal. *5*, 3680-3688.
